# Synthesis and Characterization of Tritium‐Labeled Neuropeptide Y Y_5_ Receptor Ligands Derived From CGP71683A

**DOI:** 10.1002/ardp.70314

**Published:** 2026-08-17

**Authors:** Franziska Schettler, Pierre Koch, Max Keller

**Affiliations:** ^1^ Institute of Pharmacy, Faculty of Chemistry and Pharmacy University of Regensburg Regensburg Germany

**Keywords:** binding assay, neuropeptide Y, radioligand, Y_5_ receptor

## Abstract

Among the human neuropeptide Y (NPY) receptors, the Y_5_ receptor (Y_5_R) represents a potential therapeutic target for the treatment of obesity and stress‐related diseases. As the Y_5_R is expressed in various malignant tumors, it is also considered a potential target for cancer diagnosis and therapy. Although numerous non‐peptidic Y_5_R antagonists with high binding affinity have been reported, only labeled peptidic agonists are used in routine Y_5_R competition binding assays, which are needed for the development and characterization of Y_5_R ligands. In the present study, three radiolabeled derivatives of the high‐affinity Y_5_R antagonist CGP71683A (**1**) were synthesized ([^3^H]**26**, [^3^H]**32**, [^3^H]**42**) and studied in saturation binding assays using adherent or suspended HEC‐1B‐hY_5_R cells or membrane preparations thereof. Their *K*_d_ values (1.5–13 nM) were consistent with the *K*_i_ values (1.3–11 nM) of the non‐labeled analogs. However, all synthesized radioligands exhibited very high non‐specific binding, primarily caused by binding of the radioligands to cellular membranes, which limits their use as tool compounds in radiochemical Y_5_R binding assays. Nevertheless, a competition binding assay with [^3^H]**32** and the endogenous Y_5_R agonist hNPY afforded a *K*_i_ that was in agreement with literature data.

## Graphical abstract

From a synthesized series of 2,4‐diaminoquinazoline‐type Y_5_R ligands, three were prepared as tritium‐labeled compounds and characterized by saturation equilibrium binding at HEC‐1B‐hY5R cells. [^3^H]**32** was further applied in a competition binding assay. High non‐specific binding of the radioligands prompted investigation of material‐dependent adsorption.

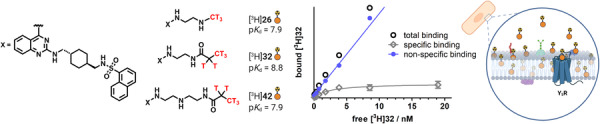

**Abbreviations**
Aib: 2‐aminoisobutyric acid
BSA: bovine serum albumin
CHO: Chinese hamster ovary
CV: column volume
DIPEA: *N*,*N*‐diisopropylethylamine
dpm: disintegration per minute
EtOAc: ethylacetate
EtOH: ethanol
FBS: fetal bovine serum
HATU: *O*‐(7‐azabenzotriazol‐1‐yl)‐*N*,*N*,*N′*,*N′*‐tetramethyluronium
HEC: human endometrial cancer
HEPES: 4‐(2‐hydroxyethyl)‐1‐piperazineethanesulfonic acid
His‐hPYY: N‐terminally histidinylated analog of hPYY
hNPY: human neuropeptide Y
hPP: human pancreatic polypeptide
hPYY: human peptide YY
*k*
: retention (or capacity) factor (HPLC)
MeOH: methanol
PBS: phosphate‐buffered saline
p*K*_i_
: negative decadic logarithm of the dissociation constant *K*_i_ (in M) obtained from a competition binding experiment
pNPY: porcine neuropeptide Y
PYY: peptide YY
RP: reversed phase
SEM: standard error of the mean
TFA: trifluoroacetic acid
*t*_R_
: retention time
Y_*x*_R: NPY Y_*x*_ receptor

## 1 Introduction

**Figure 1:**
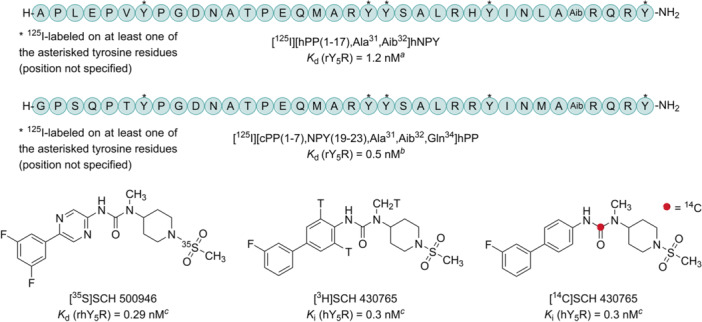
Structures and binding affinities of reported Y_5_R‐selective radioligands labeled with ^125^I, ^35^S, ^3^H or ^14^C. ^a^Dumont et al. [17], ^b^Dumont et al. [18], ^c^Mullins et al. [19]. The stated *K*_i_ value corresponds to the non‐labeled analog of the radioligand. Note: the depicted structure of [^35^S]SCH 500946 corresponds to the structure in the original report [19]. In Hesk et al. 2018 [21], a different structure of [^35^S]SCH 500946 is presented (phenyl moiety instead of the pyrazine ring).

The human genome comprises five genes encoding neuropeptide Y (NPY) receptors, namely Y_1_, Y_2_, Y_4_, Y_5_, and y_6_ receptors. However, as the latter represents a pseudogene encoding a nonfunctional receptor, only the Y_1_, Y_2_, Y_4_, and Y_5_ receptor subtypes are of physiological relevance in humans. The protein initially identified as the Y_3_ receptor was later shown to be the CXCR4 chemokine receptor [1]. NPY receptors (YR) are activated by the homologous neuropeptides NPY, peptide YY (PYY), and pancreatic polypeptide (PP) [2]. The Y_5_R is expressed in the central nervous system in various brain regions (e.g., hippocampus, hypothalamus, olfactory bulb) [3, 4] and in peripheral organs such as the colon (jejunal epithelial crypt cells and nonepithelial colon) [5], kidney [6], and in adipose tissue [7]. Primarily activated by NPY, the Y_5_R is involved in the regulation of food intake [8, 9], energy homeostasis [10], and mood disorders [11, 12]. Since the Y_5_R is expressed in different types of tumors, for example, neuroblastoma, breast cancer, and hepatic cancer, it represents a potential target for tumor imaging and cancer therapy [13, 14, 15, 16]. Determinations of Y_5_R ligand binding affinities have been performed using radiolabeled derivatives of NPY, PYY, and PP. In view of Y_5_R‐selective radioligands, two peptidic probes, [^125^I][hPP(1–17), Ala^31^, Aib^32^]hNPY and [^125^I][cPP(1–7), NPY(19–23), Ala^31^, Aib^32^, Gln^34^]hPP, and radiolabeled derivatives of the Y_5_R antagonists SCH 500946 and SCH 430765 were reported (Figure 1) [17, 18, 19]. Compared to labeled receptor agonists, labeled antagonists offer several advantages as they usually do not induce receptor internalization and their receptor binding affinities are less dependent on G protein recruitment to the receptor [20].

**Figure 2:**
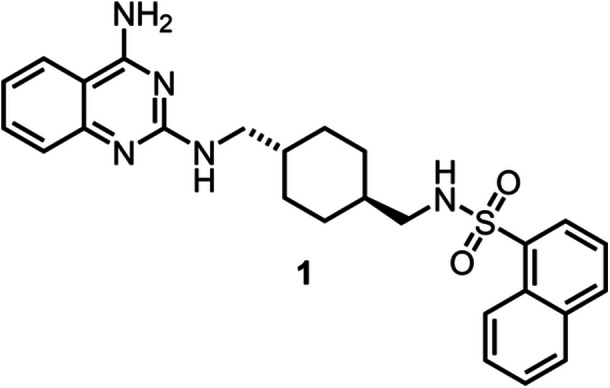
Structure of CGP71683A (**1**), a Y_5_R antagonist, displaying high selectivity over Y_1_R, Y_2_R, and Y_4_R. The following IC_50_ values (radioligand competition binding assays) are reported: hY_5_R = 2.9 nM, hY_1_R = 8370 nM, hY_2_R = 1890 nM, hY_4_R = 5740 nM [22].

Considering the reported ^35^S‐labeled derivative of the Y_5_R antagonist SCH 500946, this radioligand does not represent an ideal molecular tool for the determination of Y_5_R ligand binding affinities, because it showed a pseudo‐irreversible binding to the Y_5_R and its binding site at Y_5_R was suggested to be distinct from that of the endogenous receptor ligands [19]. There is only one report in the literature describing the use of [^35^S]SCH 500946 for determining Y_5_R binding affinities of structurally related analogs in competition binding assays [19]. The radioligands [^3^H]SCH 430765 and [^14^C]SCH 430765 were developed for the purpose of ADME studies [21]. However, in vivo studies as well as Y_5_R competition binding assays with these radioligands have not been reported to the best of our knowledge. The naphthalene sulfonamide CGP71683A (**1**) represents another selective Y_5_R antagonist with high binding affinity (Figure 2) [22]. The binding of **1** to Y_5_R has been investigated using radiolabeled NPY and PYY derivatives [23, 24], indicating a competitive interaction between the physiological Y_5_R agonists and antagonist **1**. Aiming for a radiolabeled Y_5_R antagonist that can serve as a tool compound for Y_5_R competition binding assays, three tritium‐labeled derivatives of **1** were prepared and characterized in saturation binding experiments using different formats of radiochemical binding assays.

## 2 Results and Discussion

### 2.1 Ligand Design, Synthesis, and Chemical Stability

**Scheme 1:**
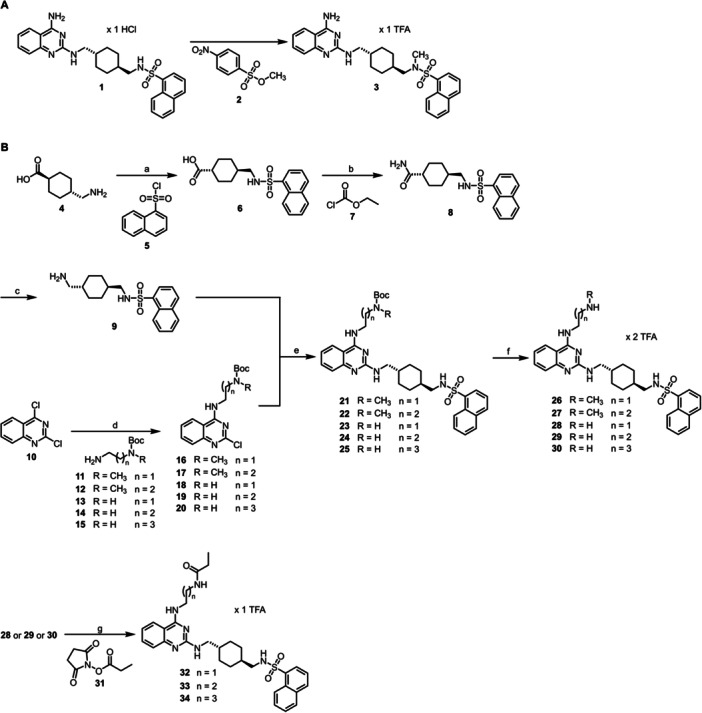
Synthesis of derivatives of **1** representing either precursors for radiolabeling (**28**–**30**) or the non‐labeled forms of potential radioligands (**26**, **27**, **32**–**34**). Reagents and conditions: (A) 2 M NaHMDS in THF, MeOH, rt, 1 h, 45%. (B) (a) 1 N NaOH, CH_2_Cl_2_, rt, overnight, 100%; (b) (i) triethylamine, THF, −5°C, 45 min; (ii) 25% aq. NH_3_, THF, −5°C to rt, 5 h, 73%; (c) BH_3_‐THF, THF, reflux, 3 h, 59%; (d) DIPEA, 2‐propanol, rt, 1–5 h, 78%–100%; (e) 1‐butanol, microwave (150°C–160°C, 1 h), 47%–64%; (f) 2 N HCl in dioxane or MeOH, rt, 3–15 h, 39%–83%; (g) DIPEA, MeOH or DMF, rt, 2 h–1.5 days, 14%–29%.

The design of the tritium‐labeled Y_5_R ligands was driven by two major considerations: first, structural modification of parent compound **1** should affect Y_5_R binding as little as possible, and second, the radiolabel should be introduced in the final step of the synthesis. Common reactions for the introduction of a tritiated group include, for example, methylation using [^3^H]methyl iodide or [^3^H]methyl nosylate, as well as propionylation using succinimidyl [^3^H]propionate. In a first approach, compound **1** was directly methylated using methyl iodide or methyl nosylate (**2**) as a methylating agent. Several reaction conditions were tested and monitored by RP‐HPLC (Table 1). Methylation of **1** occurred only under strongly basic conditions, yielding one monomethylated derivative of **1**, compound **3**, without detectable formation of side products. For scale‐up, **2** was used due to its more convenient handling compared to methyl iodide (Scheme 1A). However, methylation occurred at the sulfonamide nitrogen (as identified via HSQC and HMBC), which is known to be unfavorable for substitution, as this NH is assumed to act as a hydrogen bond donor in terms of Y_5_R binding [22, 25].

**Table 1:** Summary of reagents and reaction conditions for the preparation of **3** from **1** a.

Methylation agent		Base		Temperature	Product/Side product formation
Methyl nosylate (**2**)	1 equiv.	K_2_CO_3_	2 equiv.	rt	None
Methyl iodide	1 equiv.	K_2_CO_3_	2 equiv.	rt	None
Methyl nosylate (**2**)	1 equiv.	DIPEA	2 equiv.	rt	None
Methyl iodide	5 equiv.	DIPEA	2 equiv.	rt	None
Methyl iodide	5 equiv.	DIPEA	2 equiv.	50°C	None
Methyl iodide	3 equiv.	NaH	4 equiv.	rt	None
Methyl iodide	3 equiv.	NaH	4 equiv.	50°C	None
Methyl iodide	5 equiv.	NaHMDS	3 equiv.	rt	Product
Methyl nosylate (**2**)	2 equiv.	NaHMDS	3 equiv.	rt	Product
Methyl nosylate (**2**)	1 equiv.	NaHMDS	2 equiv.	rt	Product

^a^ One equivalent of **1** was used throughout. Reaction times (15 min–16 h) are not given as the time was not the decisive parameter for product formation.

As substituents at the amino group in position 4 of the 2,4‐diaminoquinazoline moiety have been reported to be tolerated with respect to Y_5_R binding [26], the naphthalene sulfonamides **26**–**30** and **32**–**34**, bearing different side chains in this position, were synthesized (Scheme 1B). The synthesis was guided by reported procedures [22, 27]. Sulfonylation of *trans*‐4‐(aminomethyl)cyclohexane‐1‐carboxylic acid (**4**) with 1‐naphthalenesulfonyl chloride (**5**) gave carboxylic acid **6**. Conversion of **6** via ethyl chloroformate (**7**) into an anhydride followed by ammonolysis afforded amide **8**, which was reduced with BH_3_‐THF to amine **9**. Reaction of 2,4‐dichloroquinazoline (**10**) with the primary amines **11–15** afforded the quinazoline derivatives **16–20** by regioselective substitution of the chlorine atom in position 4. Subsequent substitution of the chlorine atom in position 2 by amine **9** under harsher conditions gave the Boc‐protected intermediates **21–25**, which were treated with hydrochloric acid to obtain the amines **26**–**30**.

**Scheme 2:**
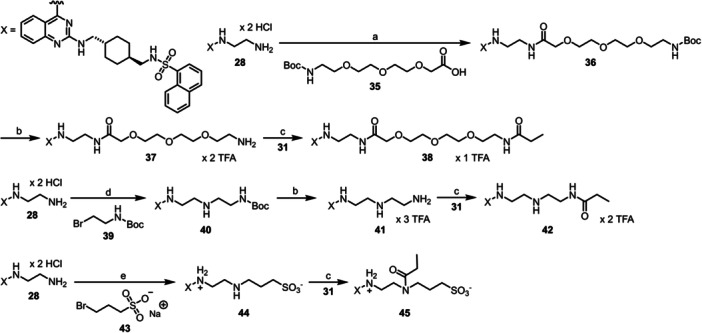
Synthesis of derivatives of **1** representing either precursors for radiolabeling (**37**, **41**, **44**) or the non‐labeled forms of potential radioligands (**38**, **42**, **45**). Reagents and conditions: (a) HATU, DIPEA, DMF, rt, overnight, 30%; (b) 2 N HCl in MeOH, rt, 3 h, 53%–63%; (c) DIPEA, DMF, rt, 1.5‐16 h, 46%–83%; (d) K_2_CO_3_, acetonitrile, microwave (110°C, 15 min), 10%; (e) DIPEA, DMF, 30°C, 18 h, 18%.

The secondary amines **26** and **27** represent the non‐labeled analogs of potential radioligands, which can be obtained by [^3^H]methylation of **28** and **29**, respectively. In contrast, propionylation of primary amines **28–30** using succinimidyl propionate (**31**) afforded the propionamides **32–34**, also representing the non‐radioactive analogs of potential radioligands, which are accessible by [^3^H]propionylation of **28–30**. Generally, the synthesis of radioligands via methylation of primary or secondary amines with tritiated methylating agents is less convenient than the preparation of radioligands by [^3^H]propionylation, as alkylation yields not only the monomethylated product but also the di‐, and in the case of primary amines, also the trimethylated amines or ammonium ions. The separation of the different alkylation products is challenging due to their structural similarity. These aspects prompted us towards the synthesis of [^3^H]**32**. However, as [^3^H]**32** showed very high non‐specific binding in the radiochemical binding assay (see ‘Characterization of [^3^H]**26**, [^3^H]**32**, and [^3^H]**42** by Saturation Binding’), further derivatives of **1** with increased polarity or altered net charge at pH 7.4 were synthesized. These approaches resulted in the target compounds **38** (introduction of a PEG linker), **42** (insertion of a secondary amine structure in the side chain of **32**), and **45** (incorporation of a negatively charged sulfonate group) (Scheme 2). For the syntheses of **38** and **42**, primary amine **28** was acylated with 2,2‐dimethyl‐4‐oxo‐3,8,11,14‐tetraoxa‐5‐azahexadecan‐16‐oic acid (**35**) in the presence of HATU, or alkylated by treatment with *tert*‐butyl (2‐bromoethyl)carbamate (**39**) to afford the Boc‐protected intermediates **36** and **40**, respectively. Subsequent deprotection with hydrochloric acid gave **37** and **41**, which were treated with **31** to afford compounds **38** and **42** (Scheme 2). Notably, propionylation of **41**, containing a secondary and primary amino group in the linker, proceeded regioselectively at the sterically readily accessible terminal primary amino group (only traces of side products were formed). The moderate yield of **42** of 61% can be attributed to partial hydrolysis of the succinimidyl ester **31** and an incomplete recovery during purification. The structure of **42** was confirmed by 2D NMR spectroscopy (see Supporting Information). The key signal, proving the correct constitution, is the coupling of the C‐atom of the propionyl carbonyl group to a hydrogen atom bound to a nitrogen atom (identified by HSQC) in the HMBC spectrum (see Supporting Information). In the case of a propionylation of the secondary amino group, such a coupling cannot be observed since a propionylated secondary amino group does not contain a hydrogen atom. Concerning the synthesis of **45**, treatment of **28** with sodium 3‐bromopropane‐1‐sulfonate (**43**) resulted in a mixture of intermediate **44** and an unidentified side product, which could not be separated by repeated purification using preparative RP‐HPLC. Propionylation of impure **44** with **31** gave **45**.

Since **42** exhibited promising Y_5_R binding affinity (*K*_i_ = 11 nM, cf. Table 2), its tritiated analog [^3^H]**42** was synthesized (see below). In contrast, due to the low Y_5_R binding affinity of **38** (*K*_i_ = 120 nM, cf. Table 2), its tritiated analog was not prepared. Instead, the radioligand [^3^H]**26**, representing a truncated analog of [^3^H]**42** with respect to the length of the side chain that still contains the basic secondary amine structure, was synthesized.

**Table 2:** Y receptor binding data of **1**, **3**, **26**–**30**, **32**–**34**, **37**, **38**, **41**, **42**, **45**, and His‐hPYY.

Compd.	p*K*_i_ ± SEM/*K*_i_ [nM]			
	hY_5_R^a^	hY_1_R^b^	hY_2_R^c^	hY_4_R^d^
**1**	9.2 ± 0.2/0.7	n.d.	n.d.	n.d.
**3**	6.8 ± 0.1/181	n.d.	n.d.	n.d.
**26**	8.9 ± 0.1/1.3	< 5/>10,000	< 6/>1000	< 5/>10,000
**27**	8.40 ± 0.02/4.0	n.d.	n.d.	n.d.
**28**	8.6 ± 0.1/2.5	< 5/>10,000	< 5/>10,000	< 5/>10,000
**29**	8.6 ± 0.1/2.6	n.d.	n.d.	n.d.
**30**	7.7 ± 0.1/23	n.d.	n.d.	n.d.
**32**	8.8 ± 0.1/1.9	< 5/>10,000	< 5/>10,000	< 5/>10,000
**33**	8.6 ± 0.1/2.3	n.d.	n.d.	n.d.
**34**	8.7 ± 0.1/2.2	n.d.	n.d.	n.d.
**37**	8.1 ± 0.1/7.8	< 5/>10,000	< 5/>10,000	< 5/>10,000
**38**	7.0 ± 0.1/117	n.d.	n.d.	n.d.
**41**	7.55 ± 0.03/28.5	< 5/>10,000	< 5/>10,000	< 5/>10,000
**42**	8.0 ± 0.1/10.6	< 5/>10,000	< 5/>10,000	< 5/>10,000
**45**	< 6/>1000	< 5/>10,000	< 5/>10,000	< 5/>10,000
His‐hPYY	7.7 ± 0.1/21.3	n.d.	n.d.	n.d.

*Note:* Data represent mean values ± SEM (p*K*_i_) or mean values (*K*_i_) from 3 to 4 independent experiments performed in triplicate. n.d. not determined.

^a^ Determined by competition binding at HEC‐1B‐hY_5_R cells using [Lys^4^‐[^3^H]propionyl]pNPY (*K*_d_ = 11 nM [24], *c* = 5 nM) as radioligand.

^b^ Determined by competition binding at SK‐N‐MC neuroblastoma cells using [^3^H]UR‐MK299 (*K*_d_ = 0.058 nM [31], *c* = 0.075 nM) as radioligand.

^c^ Determined by competition binding with [Lys^4^‐[^3^H]propionyl]hPYY (*K*_d_ = 0.16 nM [31], *c* = 0.4 nM) at CHO‐hY_2_R cells.

^d^ Determined by competition binding at CHO‐hY_4_R‐G_qi5_‐mtAEQ cells using [^3^H]UR‐JG102 (*K*_d_ = 0.11 nM [29], *c* = 0.25 nM) as radioligand.

The chemical stability of **26**, **32**, and **42** was investigated in PBS (pH 7.4) at rt over 24 h. No decomposition was observed for any of the compounds studied (Supporting Information, Figures S1–S3).

### 2.2 Competitive Binding Studies at Subtypes of the Human YR Family

Y_5_R binding affinities of **3, 26–30, 32–34, 37, 38, 41, 42**, and **45** were determined in a competition binding assay using intact HEC‐1B‐hY_5_R cells and [Lys^4^‐[^3^H]propionyl]pNPY as the radioligand. The obtained radioligand displacement curves are depicted in Figure S4 (Supporting Information) and p*K*_i_ and *K*_i_ values are presented in Table 2. The determined *K*_i_ value of the parent compound **1** (*K*_i_ = 0.7 nM) was in agreement with reported Y_5_R binding data (IC_50_ = 2.9 nM [22], *K*_i_ = 0.3 nM [17], *K*_i_ = 1.3 nM [28]). Compound **3**, bearing a methyl group at the sulfonamide nitrogen, showed low affinity for the Y_5_R (*K*_i_ = 181 nM), consistent with literature data [22]. This confirms that the NH group of the sulfonamide moiety is likely involved in hydrogen bonding with Y_5_R [22, 25]. Apart from **3**, the first series of derivatives of **1** (**26–30, 32–34)** exhibited high Y_5_R binding affinities (*K*_i_ ≤ 4 nM), comparable to **1**, with the exception of **30**, which showed a reduced affinity (*K*_i_ = 23 nM). This shows that the length of the alkyl spacer between the NH group in position 4 of the diaminoquinazoline moiety and the basic amino group in the side chain is critical for Y_5_R binding: while an ethylene (**28**) and a trimethylene spacer (**29**) are well tolerated, extension to a tetramethylene spacer (**30**) results in a 10‐fold decrease in binding affinity (cf. Table 2). Compound **41**, which bears a side chain of similar length to that of **30** but contains an additional basic group (secondary amine), exhibited a Y_5_R binding affinity (*K*_i_ = 29 nM) comparable to that of **30**. Interestingly, propionylation of **30** and **41**, yielding **34** and **42**, respectively, resulted in a ca. 10‐fold (**30** → **34**) and ca. 2.5‐fold (**41** → **42**) increase in Y_5_R binding affinity, indicating that the linker length at position 4 of the 2,4‐diaminoquinazoline moiety is not the only factor contributing to reduced Y_5_R affinity. The decrease in Y_5_R binding affinity may be attributed to the positively charged terminal basic group, whose presence is unfavorable when its distance from the 2,4‐diaminoquinazoline moiety exceeds a spacer length of three heavy atoms. A different trend is observed when the side chain at the 2,4‐diaminoquinazoline scaffold is further extended through incorporation of a PEG‐linker: while the compound bearing a free primary amino group (**37**) exhibited high Y_5_R binding affinity (*K*_i_ = 7.8 nM), its propionylated congener (**38**) showed markedly reduced binding (*K*_i_ = 120 nM). These findings suggest that the positively charged terminal amino group in **37** contributes to favorable interactions with Y_5_R (e.g., an ionic interaction with an acidic amino acid), which compensates for repulsive interactions caused by the PEG‐extended side chain. Finally, the introduction of a negatively charged sulfonate group into the side chain, combined with propionylation of the secondary amine in the linker (**45**), resulted in a marked decrease in Y_5_R binding affinity (*K*_i_ >1000 nM). This may be attributed to steric hindrance in the binding pocket caused by the presence of the propionyl group or by a repulsive electrostatic interaction of the sulfonate group with an acidic amino acid of Y_5_R, or both. For selected compounds (**26**, **28**, **32**, **37**, **41**, **42**, and **45**), Y_5_R selectivity was assessed by investigating their binding to Y_1_R, Y_2_R, and Y_4_R in previously established radiochemical binding assays [29, 30, 31]. As these compounds showed very low binding affinity for these YR subtypes, p*K*_i_ values could not be determined (Table 2). The results of the binding studies demonstrate that the introduction of linkers or side chains into **1** via alkylation of the amino group at position 4 of the quinazoline scaffold is tolerated to a limited extent, depending on the actual linker structure, and does not impair the high Y_5_R selectivity of **1**.

### 2.3 Synthesis of [^3^H]26, [^3^H]32, and [^3^H]42

**Scheme 3:**
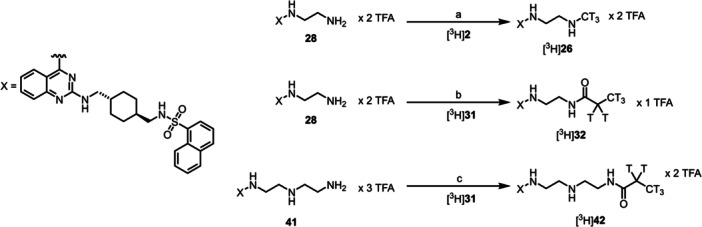
Synthesis of the radioligands [^3^H]**26**, [^3^H]**32**, and [^3^H]**42**. Reagents and conditions: (a) K_2_CO_3_, acetonitrile, rt, overnight, radiochemical yield: 15%; (b) DIPEA, DMF, rt, 2 h, radiochemical yield: 62%; (c) DIPEA, DMF, rt, 1.5 h, radiochemical yield: 47%. Note that the tritium atoms in the [^3^H]propionyl and [^3^H]methyl group do not represent a stoichiometric quantity of tritium isotopes; they only indicate that tritium is present in one of the respective positions.

The radioligands [^3^H]**32** and [^3^H]**42** were obtained in satisfactory radiochemical yield by treatment of the amine precursors **28** and **41** with succinimidyl [^3^H]propionate ([^3^H]**31**) (Scheme 3). [^3^H]**26** was synthesized by treating a 5.8‐fold excess of precursor **28** with [^3^H]methyl nosylate ([^3^H]**2**) at rt overnight, affording [^3^H]**26** in 15% radiochemical yield. The reason for the lower yield compared to [^3^H]**32** and [^3^H]**42** was the formation of a double [^3^H]methylated side product and an incomplete conversion of the labeling reagent [^3^H]**2**. The deviating synthetic route towards **26** depicted in Scheme 1 was employed to obtain **26** in higher yield by a more practical method for analytical characterization.

**Figure 3:**
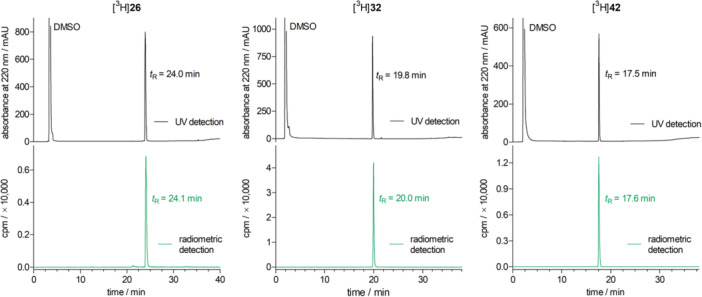
Radiochemical purities of [^3^H]**26** (97%), [^3^H]**32** (>99%), and [^3^H]**42** (>99%) determined by RP‐HPLC analysis.

Purification by RP‐HPLC gave all radioligands in excellent radiochemical purities (Figure 3). The molar activities of the prepared radioligands, which are specified by the molar activities of the used labeling reagents, amounted to 3.0 TBq/mmol ([^3^H]**26**) or 3.7 TBq/mmol ([^3^H]**32**, [^3^H]**42**).

The determination of the distribution coefficient logD_7.4_ of [^3^H]**26**, [^3^H]**32**, and [^3^H]**42** in 1‐octanol/PBS 1:1 yielded values of 1.40, 1.24, and 1.56, respectively, indicating moderate lipophilicity. Unexpectedly, the logD_7.4_ of [^3^H]**42**, which contains an additional basic group (secondary amine) in the linker compared to [^3^H]**32**, was not lower than that of [^3^H]**32**. This could be explained by reduced p*K*_a_ values of the 2,4‐diaminoquinazoline moiety or the proximal secondary amine group compared to the p*K*_a_ of an isolated 2,4‐diaminoquinazoline scaffold (p*K*_a_ ca. 8 [32]) and diethylamine (p*K*_a_ ≈ 11 [33]), resulting in a comparatively low protonation state at pH 7.4.

### 2.4 Characterization of [^3^H]26, [^3^H]32, and [^3^H]42 by Saturation Binding

**Figure 4:**
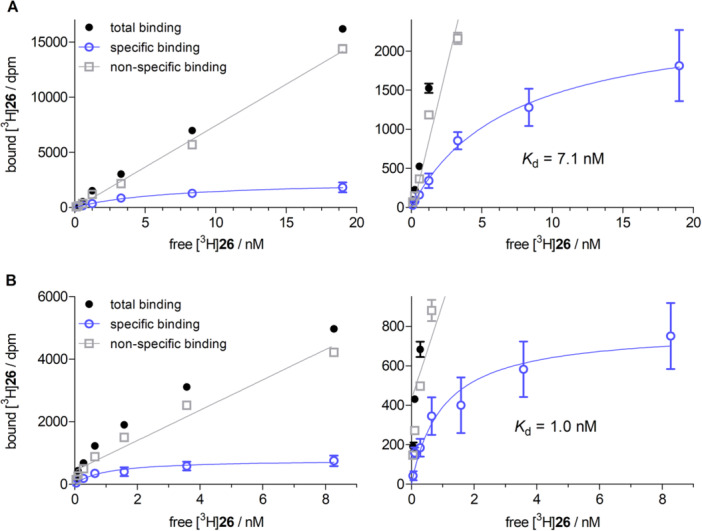
Representative Y_5_R binding isotherms (specific binding) of [^3^H]**26** obtained from saturation binding experiments performed in triplicate at adherent (A) or suspended (B) HEC‐1B‐hY_5_R cells at 22 ± 2°C. The respective illustrations show total, specific, and non‐specific binding on the left and a zoomed‐in view, capturing specific binding, on the right. Non‐specific binding was determined in the presence of 25 µM (A) or 10 µM (B) His‐hPYY. Total and non‐specific binding data represent mean values ± SEM. Specific binding data represent calculated values ± propagated error.

**Figure 5:**
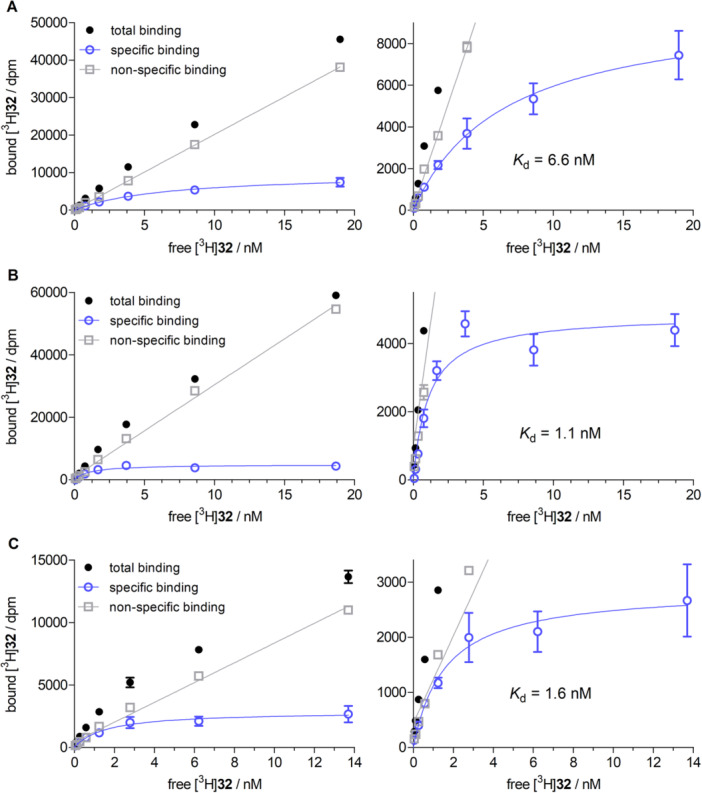
Representative Y_5_R binding isotherms (specific binding) of [^3^H]**32** obtained from saturation binding experiments performed in triplicate at adherent (A) or suspended (B) HEC‐1B‐hY_5_R cells or with membranes of HEC‐1B‐hY_5_R cells (C) at 22 ± 2°C. The respective illustrations show total, specific, and non‐specific binding on the left and a zoomed‐in view, capturing specific binding, on the right. Non‐specific binding was determined in the presence of 10 µM (A, B) or 7 µM (C) His‐hPYY. Total and non‐specific binding data represent mean values ± SEM. Specific binding data represent calculated values ± propagated error.

**Figure 6:**
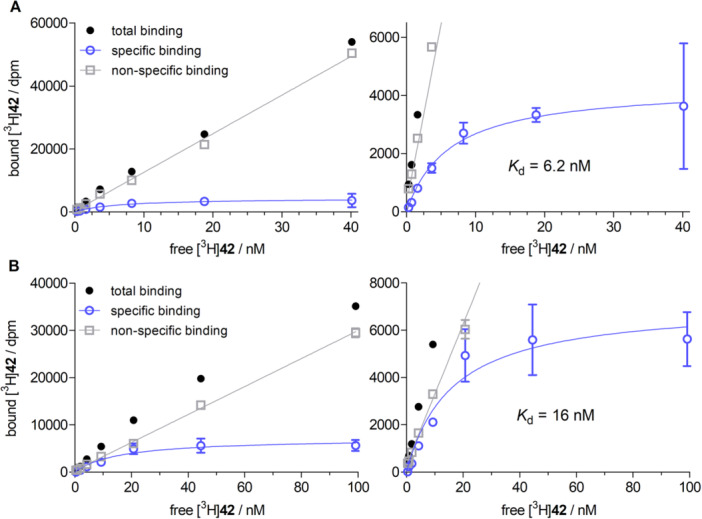
Representative Y_5_R binding isotherms (specific binding) of [^3^H]**42** obtained from saturation binding experiments performed in triplicate at adherent (A) or suspended (B) HEC‐1B‐hY_5_R cells at 22 ± 2°C. The respective illustrations show total, specific, and non‐specific binding on the left and a zoomed‐in view, capturing specific binding, on the right. Non‐specific binding was determined in the presence of 10 µM His‐hPYY. Total and non‐specific binding data represent mean values ± SEM. Specific binding data represent calculated values ± propagated error.

All synthesized radioligands were investigated in saturation binding assays using adherent and suspended HEC‐1B‐hY_5_R cells [34], which have previously been used for Y_5_R binding studies using [Lys^4^‐[^3^H]propionyl]pNPY [29, 35, 36]. Additionally, saturation binding of [^3^H]**32** was studied at membrane preparations of HEC‐1B‐hY_5_R cells and in a 24‐well assay format. For the determination of non‐specific binding, an *N*‐terminally histidinylated analog of hPYY (His‐hPYY) was used. It was available in our lab due to an unintended purchase of His‐hPYY instead of hPYY. As His‐hPYY exhibits Y_5_R binding affinity comparable to that of hPYY (cf. Table 2), it was used for the determination of non‐specific binding. Unfortunately, non‐specific binding proved to be very high in all assay types (cf. Figures 4, 5, 6 and Supporting Information: Figure S5). However, fitting of specific binding data, indicating saturable binding, afforded *K*_d_ values, which were in good agreement with the *K*_i_ values of the non‐radioactive analogs **26**, **32**, and **42** (Tables 2 and 3).

**Table 3:** Overview of p*K*_d_ values of [^3^H]**26**, [^3^H]**32**, and [^3^H]**42** obtained from saturation binding experiments at intact HEC‐1B‐hY_5_R cells or cell membranes.

Radioligand	Adherent cells		Suspended cells		Cell membranes	
	p*K*_d_/*K*_d_ [nM]^a^	Non‐specific binding at *K*_d_ (%)^b^	p*K*_d_/*K*_d_ [nM]^a^	Non‐specific binding at *K*_d_ (%)^b^	p*K*_d_/*K*_d_ [nM]^a^	Non‐specific binding at *K*_d_ (%)^b^
[^3^H]**26**	8.2 ± 0.1/6.4#	84 ± 1	8.8 ± 0.1/1.5#	73 ± 1	n.d.	n.d.
[^3^H]**32**	8.4 ± 0.1/4.5#	70 ± 1	8.9 ± 0.1/1.5#	66 ± 4	8.8 ± 0.1/1.6#	56 ± 4
	8.1 ± 0.1/7.6##					
[^3^H]**42**	8.2 ± 0.1/7.4#	76 ± 2	7.9 ± 0.1/13#	69 ± 5	n.d.	n.d.

^a^ Equilibrium dissociation constant expressed as p*K*_d_ (mean values ± SEM) and *K*_d_ (mean value) obtained from three to five independent experiments performed in triplicate at 22 ± 2°C.

^b^ Mean value of the non‐specific binding at the concentration corresponding to the *K*_d_ value, obtained from three to five independent experiments.

^#^ p*K*_d_ value determined in 96‐well plates.

^##^ p*K*_d_ value determined in 24‐well plates.

The extent of non‐specific binding at *K*_d_ ranged from 56% to 84% of total binding (Table 3), which exceeds the generally recommended limit of 50% non‐specific binding at *K*_d_ for receptor‐ligand binding assays [37]. Associated with high non‐specific binding is an increase in inaccuracy of specific binding, which forms the basis for data analysis. To explore the origin of the high non‐specific binding, the binding of [^3^H]**32** to a cell line not expressing the Y_5_R was investigated. For this purpose, [^3^H]**32** was incubated with CHO‐hY_2_R cells at concentrations and conditions identical to those used in the saturation binding assays. Binding of [^3^H]**32** to the reference cells, representing only non‐specific binding, was comparable to the non‐specific binding in HEC‐1B‐hY_5_R cells (cf. Figure 5 and Supporting Information: Figure S6), indicating that the high non‐specific binding of the radioligands is independent of the cell line. As an additional control, [^3^H]**32** was studied in a mock saturation binding experiment performed in the absence of cells, complying with the conditions of the saturation binding experiments using suspended cells. This setup exclusively measures the binding of the radioligand to the filter used for cell collection in the actual saturation binding assay. Binding of [^3^H]**32** to the filter was markedly lower (ca. 14% of non‐specific binding in the assay with cells), clearly indicating that the radioligands strongly attach to cells in a non‐specific manner. Since non‐specific binding was also high when using membrane preparations in the saturation binding experiments, it can most likely be attributed to incorporation of the radioligands into the plasma membrane rather than to cellular uptake. The high attraction by lipid bilayers might be caused by the combination of lipophilic moieties (cyclohexyl, naphthyl) and positively charged groups in the radioligand structure.

**Figure 7:**
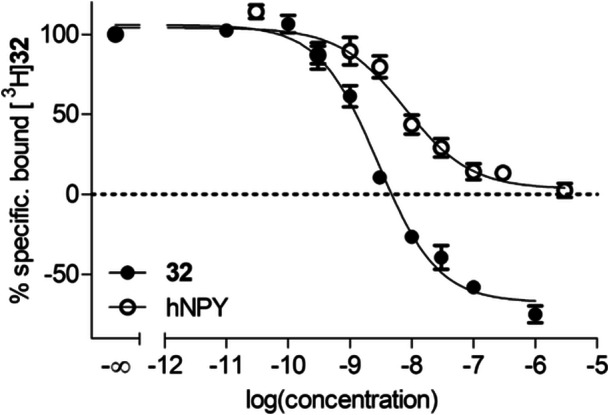
Radioligand displacement curves from competition binding experiments conducted with [^3^H]**32** (*K*_d_ = 1.6 nM, *c* = 3 nM) and **32** or hNPY at HEC‐1B‐hY_5_R cell membranes. Data represent mean values ± SEM from three independent experiments (performed in triplicate).

Despite non‐specific binding exceeding 50% at *K*_d_ in all studied assay types, competition binding experiments were performed as proof of concept. For this purpose, the *K*_i_ values of **32** and the endogenous Y_5_R ligand hNPY were determined using the system with the lowest non‐specific binding, that is, binding of [^3^H]**32** to membrane preparations of HEC‐1B‐hY_5_R cells (cf. Table 3). Whereas the lower plateau of the radioligand displacement curve of **32** reached approximately −70%, the displacement curve of hNPY showed a plateau around 0% specifically bound [^3^H]**32** (Figure 7). The strongly negative curve plateau observed for **32** can be explained by the displacement of non‐specifically bound [^3^H]**32** by its non‐radioactive analog, which is most pronounced at the highest concentrations of **32**. In contrast, hNPY, addressing different non‐specific binding sites due to its distinct structure, does not displace non‐specifically bound [^3^H]**32**. In an assay with low non‐specific binding, the phenomenon of a plateau below 0% specifically bound radioligand would be much less pronounced. These results demonstrate that high non‐specific binding, as mentioned before, impairs the robustness, reliability, and usefulness of a binding assay. Nonetheless, the *K*_i_ value obtained for **32** (*K*_i_ = 1.1 ± 0.2 nM; mean value ± SEM from three individual experiments) was in good agreement with the *K*_d_ value of [^3^H]**32** (*K*_d_ = 1.6 nM, Table 3) and the *K*_i_ value of **32** determined with [Lys^4^‐[^3^H]propionyl]pNPY (*K*_i_ = 1.9 nM, Table 2). Likewise, the obtained *K*_i_ value of hNPY, amounting to 2.2 ± 1.1 nM (mean value ± SEM from three individual experiments), was in accordance with reported Y_5_R binding data of hNPY (IC_50_ = 3 [38], *K*_i_ = 0.30 nM [39], *K*_i_ = 0.73 nM [40]).

### 2.5 Radioligand Adsorption Studies

**Figure 8:**
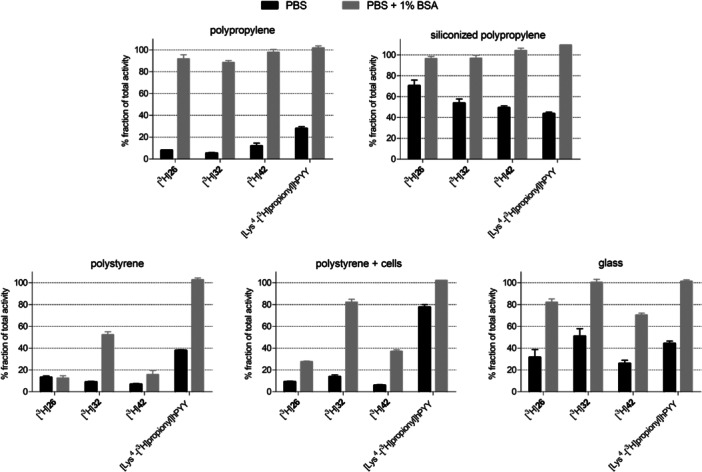
Adsorption of [^3^H]**26**, [^3^H]**32**, [^3^H]**42**, and [Lys^4^‐[^3^H]propionyl]hPYY to various materials studied for 2 nM ([^3^H]**26**, [^3^H]**32**), 13 nM ([^3^H]**42**) or 1 nM ([Lys^4^‐[^3^H]propionyl]hPYY) solutions in PBS and PBS supplemented with 1% BSA. Presented are the relative amounts of free (non‐adsorbed) radioligand after 2 h of incubation at rt in the respective tube or vessel. “Polystyrene” stands for a tissue culture‐treated white/transparent 96‐well plate (VWR, catalog no. 732‐3740). “Polystyrene + cells” stands for the same 96‐well plate containing a confluent layer of adherent CHO‐K1 cells on the bottom. The test solutions of the radioligands were prepared in polypropylene reaction vessels immediately followed (< 1 min) by the removal and transfer of an aliquot (200 µL) into the vessel under study and the withdrawal of an aliquot (10 µL) to determine the value of 100% activity. Data represent mean values ± SEM from three to six individual experiments performed in a singlet (radioactivity was measured in triplicate for each experiment).

Prompted by the high non‐specific binding observed in the radioligand binding assays, the adsorption of [^3^H]**26**, [^3^H]**32**, and [^3^H]**42** to various materials in PBS without BSA and PBS supplemented with 1% BSA was investigated at rt. Additionally, the recently reported peptidic radioligand [Lys^4^‐[^3^H]propionyl]hPYY [31] was included in these studies. The concentrations of the radioligands used corresponded approximately to their *K*_d_ values, as determined in saturation binding experiments. Incubations were carried out for 2 h, consistent with typical conditions of radiochemical binding assays. The tested materials comprised polypropylene tubes, siliconized polypropylene tubes (coated with SigmaCote, Sigma Aldrich), tissue culture‐treated white 96‐well plates with transparent bottom, the same plates with a confluent cell layer, and borosilicate glass. Results are presented as the recovered radioactivity after 2 h incubation (Figure 8). All studied radioligands showed strong adsorption to polypropylene in the absence of BSA. In the case of siliconized polypropylene tubes, adsorption in the absence of BSA was markedly lower. In PBS containing BSA, the radioligands displayed very weak adsorption to both, non‐siliconized and siliconized polypropylene tubes. The effect of BSA can be attributed to the surface coating of the material with BSA, preventing the adsorption of the radioligand. Furthermore, as the adsorption of a compound is connected to its solubility in the respective solvent, reversible binding of the radioligands to dissolved BSA, resulting in increased solubility, may also contribute to decreased radioligand adsorption.

In white/clear 96‐well plates, [^3^H]**26**, [^3^H]**32**, and [^3^H]**42** displayed strong adsorption in the absence of BSA (>80%), whereas [Lys^4^‐[^3^H]propionyl]hPYY showed a moderate adsorption (ca. 60%). Interestingly, in the presence of BSA, only [^3^H]**32** and [Lys^4^‐[^3^H]propionyl]hPYY showed a considerably lower adsorption compared to BSA‐free PBS. In white/clear 96‐well plates with a confluent cell layer, a similar picture was obtained with minor differences: adsorption of [Lys^4^‐[^3^H]propionyl]hPYY was lower in the absence of BSA (ca. 20%), and the effect of BSA was more pronounced for [^3^H]**26**, [^3^H]**32**, and [^3^H]**42**. For borosilicate glass, the adsorption profile resembled that of siliconized polypropylene tubes: moderate adsorption in PBS without BSA and low adsorption in the presence of BSA. Summing up, the adsorption studies confirmed that BSA is an effective buffer additive to minimize adsorption of bioactive compounds when studied in biological assays [41, 42]. However, it should be kept in mind that reversible binding of the radioligand to BSA can result in a decreased concentration of free radioligand, which might also affect the results of binding assays.

## 3 Conclusion

Aiming for a small‐molecule radioligand for the Y_5_R, three radiolabeled derivatives ([^3^H]**26**, [^3^H]**32**, [^3^H]**42**) of CGP71683A (**1**) were prepared by modification of **1** in position 4 of the 2,4‐diaminoquinazoline scaffold. With *K*_d_ values of 1.5–13 nM, the radioligands exhibit high Y_5_R binding affinities. Due to very high non‐specific binding in radiochemical binding assays, their use as tool compounds for Y_5_R binding studies is limited. However, Y_5_R binding assays involving these radioligands as probes might be implementable with cells exhibiting very high Y_5_R expression. As demonstrated in the present study, a modification of the radioligand structure by attachment of polar moieties, potentially leading to reduced non‐specific binding, is challenging in terms of preserving high Y_5_R binding affinity. Moreover, it is uncertain whether such a strategy would lead to substantially decreased non‐specific binding since the scaffold of **1** shows high affinity to lipid bilayers. The synthesis of radiolabeled analogs of Y_5_R antagonists with structures different from **1** and the aryl‐piperidinyl urea derivatives (cf. Figure 1) could provide access to useful Y_5_R radioligands. The presented radioligands also show pronounced adsorption to polystyrene, a standard material of microtiter plates. These findings recapitulate that non‐labeled therapeutics or drug candidates can potentially display high non‐specific binding to biomembranes and other materials even at low concentrations. Such a feature might result in overestimated free ligand concentrations and consequently in underestimated bioactivities.

## 4 Experimental

### 4.1 Chemistry

#### 4.1.1 General

##### 4.1.1.1 Materials

*Tert*‐butyl (2‐bromoethyl)carbamate, 1‐naphthalenesulfonyl chloride, 2,2‐dimethyl‐4‐oxo‐3,8,11,14‐tetraoxa‐5‐azahexadecan‐16‐oic acid, 2,4‐dichloroquinazoline, CGP71683A, sodium 3‐bromopropane‐1‐sulfonate, *tert*‐butyl (2‐aminoethyl)(methyl)carbamate, *tert*‐butyl (2‐aminoethyl)carbamate, *tert*‐butyl (3‐aminopropyl)(methyl)carbamate, *tert*‐butyl (3‐aminopropyl)carbamate, *tert*‐butyl (4‐aminobutyl)carbamate, *trans*‐4‐(aminomethyl)cyclohexane‐1‐carboxylic acid, and HATU were obtained from BLDpharm (Reinbek, Germany). Anhydrous potassium carbonate, 2 M NaHMDS in THF, TFA, extra dry dichloromethane, and extra dry THF (both over molecular sieve, stabilized) were ordered from ACROS/Fisher Scientific (Schwerte, Germany). Potassium carbonate pellets, methyl iodide, and triethylamine were purchased from Merck (Darmstadt, Germany). 1 N NaOH, 2‐propanol, and Na_2_SO_4_ were from Carl Roth (Karlsruhe, Germany). 1‐Butanol, 4 N HCl in dioxane, borane THF complex solution, di‐*tert*‐butyl dicarbonate, ethyl chloroformate, methyl 4‐nitrobenzenesulfonate, 1‐octanol, DIPEA, Triton X‐100, Sigmacote, geneticin (G418), minimum essential medium (EMEM), and Ham's F‐12 medium were obtained from Sigma Aldrich (Taufkirchen, Germany). Human peptide YY (hPYY), human neuropeptide Y (hNPY), porcine neuropeptide Y (pNPY), human pancreatic polypeptide (hPP), and histidyl hPYY (His‐hPYY) were purchased from Synpeptide (Shanghai, China). Bacitracin, HEPES, and bovine serum albumin (BSA) were obtained from Serva (Heidelberg). Zeocin and hygromycin B were obtained from InvivoGen (Toulouse, France). Fetal bovine serum (FBS) was purchased from Pan Biotech (Aidenbach, Germany). [^3^H]**2** (molar activity: 3 TBq/mmol) and [^3^H]**31** (molar activity: 3.7 TBq/mmol) were obtained from Novandi (Södertälje, Sweden). The Y_2_R antagonist BIIE0246 was kindly provided by Boehringer Ingelheim via its open innovation platform opnMe (Ingelheim, Germany), and JNJ31020028 was purchased from Cayman Chemical (Ann Arbor, USA). The syntheses of [^3^H]UR‐JG102 [29] (molar activity: 3.44 TBq/mmol), [^3^H]UR‐MK299 [30] (molar activity: 3.885 TBq/mmol), and [Lys^4^‐[^3^H]propionyl]hPYY [31] (molar activity: 3.44 TBq/mmol) were described previously. [Lys^4^‐[^3^H]propionyl]pNPY (molar activity: 1.39 TBq/mmol) was prepared according to a previously reported procedure [30] with minor modifications that were described elsewhere [36]. Succinimidyl propionate (**31**) was prepared according to a reported procedure [30]. Gradient grade (HPLC) acetonitrile and MeOH were purchased from VWR (Darmstadt, Germany). Technical‐grade solvents were used without further purification. Millipore water was consistently used for the preparation of stock solutions, buffers, and eluents for HPLC. Polypropylene reaction vessels (1.5 and 2 mL) were obtained from Sarstedt (Nümbrecht, Germany) and were used to prepare and keep stock solutions, for stability studies, and as reaction vessels. Reactions using microwave irradiation were performed on the Microwave Synthesizer Initiator from Biotage (Uppsala, Sweden).

##### 4.1.1.2 Elemental Analysis

Elemental analysis was performed with a Vario MICRO Cube elemental analyzer (Elementar Analysensysteme, Hanau, Germany).

##### 4.1.1.3 Mass Spectrometry

High‐resolution mass spectrometry (HRMS) was performed with an Agilent 6540 UHD accurate‐mass Q‐TOF LC/MS system coupled to an Agilent 1290 analytical HPLC system (Agilent Technologies, Santa Clara, CA) using an ESI source and the following LC method: column: Zorbax Eclipse Plus C18, 1.8 μm, 50 × 2.1 mm (Agilent), column temperature: 40°C, solvent/linear gradient: 0 − 4 min: 0.1% aqueous HCOOH/acetonitrile supplemented with 0.1% HCOOH 95:5−2:98, 4−5 min: 2:98, flow: 0.6 mL/min. Low‐resolution mass spectrometry (ESI‐MS) was either performed on the same device as HRMS or on an Advion expression CMS ESI‐MS system (Interchim, Montluçon, France) operated in ESI+ mode (capillary temperature 200°C, capillary voltage 160 V, source gas temperature 250°C, ESI voltage 3500 V) and ESI‐ mode (capillary temperature 200°C, capillary voltage 160 V, source gas temperature 250°C, ESI voltage 2500 V). For sample administration, the probe was diluted in MeOH and directly injected into the ESI source via a syringe. MeOH was used as carrier fluid.

##### 4.1.1.4 NMR Spectroscopy

NMR spectra were recorded on an AVANCE 600 instrument with cryogenic probe (^1^H: 600 MHz; ^13^C: 151 MHz), an AVANCE 500 (^1^H: 500 MHz; ^13^C: 126 MHz), or an AVANCE 400 (^1^H: 400 MHz; ^13^C: 101 MHz) (Bruker, Karlsruhe, Germany). NMR spectra were calibrated based on the solvent residual peaks (^1^H‐NMR, DMSO‐*d*_6_: δ = 2.50 ppm; ^13^C‐NMR, DMSO‐*d*_6_: δ = 39.52 ppm). Data are reported as follows: ^1^H‐NMR: chemical shift δ in ppm (multiplicity [s = singlet, d = doublet, t = triplet, m = multiplet, and br s = broad singlet], integral, coupling constant *J* in Hz); ^13^C NMR: chemical shift δ in ppm. Annotation concerning the ^13^C NMR spectra of the target compounds: The carbon atom signals were assigned to their respective C‐atoms using the following abbreviations: carbonyl^x^, C‐atom of the carbonyl group of residue x; Cq, quaternary carbon atom; CH_x_, carbon atom bearing x hydrogen atoms; qz, 2,4‐diaminoquinazoline moiety; naph, naphthalene moiety; cy, cyclohexane; cy‐exo, exocyclic CH_2_ group at the cyclohexane ring; linker, linker that is attached to the nitrogen in 4‐position of the 2,4‐diaminoquinazoline ring; prop, propionamide; PEG‐linker, carbon atoms of the PEG‐linker; CF_3_, CF_3_‐group of TFA.

##### 4.1.1.5 Preparative HPLC

Preparative HPLC was performed with a system from Knauer (Berlin, Germany) consisting of two K‐1800 pumps and a K‐2001 detector (in the following referred to as “system 1”) or with a Prep 150 LC system from Waters (Eschborn, Germany) comprising a Waters 2545 binary gradient module, a Waters 2489 UV/vis detector, and a Waters fraction collector III (in the following referred to as “system 2”). A Gemini NX‐C18, 5 μm, 250 mm × 21 mm (Phenomenex) was used as a reversed‐phase (RP) column at a flow rate of 20 mL/min using mixtures of 0.1% aq. TFA and acetonitrile as the mobile phase. A detection wavelength of 220 nm was used throughout. Collected fractions were lyophilized using a Scanvac CoolSafe 100‐9 freeze dryer (Labogene, Allerød, Denmark) equipped with an RZ 6 rotary vane vacuum pump (Vacuubrand, Wertheim, Germany).

##### 4.1.1.6 Analytical HPLC

Analytical HPLC analysis was performed with an 1100 series system from Agilent Technologies composed of a degasser (G1379A), a binary pump (G1312A), a variable wavelength detector (G1314A), a thermostated column compartment (G1316A), and an autosampler (G1329A). Detection was performed at 220 nm, and the oven temperature was 30°C. For the analysis of reaction mixtures and purity controls, a Kinetex‐XB C18 100 A, 5 μm, 250 × 4.6 mm (Phenomenex) served as the stationary phase at a flow rate of 0.8 mL/min. Mixtures of 0.05% aq. TFA (A) and acetonitrile (B) were used as the mobile phase. The following linear gradient was applied: 0–30 min: A/B 90:10–5:95, 30–40 min: 5:95 (isocratic). The injection volume was 50 μL. Retention (capacity) factors *k* were calculated from the retention times *t*_R_ according to *k* = (*t*_R_‐*t*_0_)/*t*_0_ (*t*_0_ = dead time, 2.6 min for the system, column, and flow rate mentioned above).

#### 4.1.2 Synthesis Protocols and Analytical Data

##### 4.1.2.1 General Procedures

A. Nucleophilic aromatic substitution of 2,4‐dichloroquinazoline (**10**): The corresponding primary amine (1.0–1.1 equiv.) and DIPEA (2.0 equiv.) were added to a suspension of **10** (1.0 equiv.) in 2‐propanol. The mixture was stirred at rt for 1–5 h. The solvent was removed by rotary evaporation, the residue was taken up with water, and the aqueous phase was washed with CH_2_Cl_2_ (3–4 ×). The combined organic phases were dried over Na_2_SO_4,_ and the volatiles were removed under reduced pressure. The product was obtained after purification via flash chromatography.
B. Nucleophilic aromatic substitution of 4‐*N*‐alkylated 2‐chloroquinazoline derivatives: The respective 4‐*N*‐alkylated 2‐chloroquinazoline derivative (1.0 equiv.) and **9** (1.0 equiv.) were suspended in 1‐butanol. The mixture was stirred under microwave irradiation (150°C–160°C, 1 h) in a sealed tube. The solvent was removed under reduced pressure, and the residue was subjected to flash chromatography to obtain the pure product.
C. Cleavage of the Boc‐protecting group: The respective Boc‐protected amine was dissolved in 2 N HCl in MeOH or dioxane, and the mixture was stirred at rt for 3 h if not stated otherwise. The volatiles were removed *in vacuo*, and the residue was purified by preparative HPLC or used without further purification in the next step.
D. Propionylation with succinimidyl propionate (**31**): A solution of the corresponding amine (1.0 equiv.) and DIPEA (2.0–7.3 equiv.) in MeOH or DMF was stirred at rt for 15–40 min. **31** (1.5–2.2 equiv.) was dissolved in MeOH or DMF and added in two equal portions to the solution of the amine with a time gap of 2 min. When the reaction control by HPLC indicated sufficient conversion, the mixture was diluted with 10% aq. TFA (in an amount corresponding to the used amount of DIPEA) and subjected to preparative HPLC.

##### 4.1.2.2 Detailed Synthesis Protocols

Annotation concerning the determination of the number of trifluoroacetate counter ions for compounds isolated as hydrotrifluoroacetates: due to the presence of at least one basic moiety in the 2,4‐diaminoquinazoline derivatives, target compounds purified by preparative HPLC using 0.1% TFA in water as aqueous eluent were obtained as hydrotrifluoroacetates. The number of trifluoroacetate counter ions was exemplarily determined for **28** by elemental analysis, revealing the presence of two trifluoroacetate counter ions (bound to the protonated basic 2,4‐diaminoquinazoline moiety and the protonated basic primary amino group). Based on this knowledge, the number of trifluoroacetate counter ions was assigned to the other target compounds.

*N*‐{[(1*r*,4*r*)‐4‐{[(4‐Aminoquinazolin‐2‐yl)amino]methyl}cyclohexyl]methyl}‐*N*‐methylnaphthalene‐1‐sulfonamide hydrotrifluoroacetate (**3**) [27]: 2 M NaHMDS in THF (15 µL) was added to a solution of **1** hydrochloride (4.9 mg, 9.57 µmol) in MeOH (100 µL). After stirring at rt for 30 min, **2** (4.2 mg, 19.14 µmol) in DMF (10.2 µL) was added, and stirring was continued at rt. The reaction was stopped by the addition of water (1 mL) after 1 h, giving a cloudy mixture. DMF was added dropwise until the solution was limpid and was then subjected to preparative HPLC (system 1, gradient: 0–23 min: 0.1% aq. TFA/acetonitrile: 90:10–25:75, *t*_R_ **=** 18 min), yielding a white fluffy solid (2.6 mg, 45%). ^1^H NMR (600 MHz, DMSO‐*d*_*6*_) (two major rotamers with a ratio of approximately 2.3:1 were evident in the ^1^H and ^13^C NMR spectra) δ 12.69 (br s, 0.7H), 11.58 (br s, 0.3H), 9.08 (s, 0.7H), 8.96 (s, 0.7H), 8.84 (br s, 0.3H), 8.59 (d, *J* **=** 8.7 Hz, 1.5H), 8.38 (br s, 0.5H), 8.25 (d, *J* **=** 8.6 Hz, 1H), 8.19 (d, *J* **=** 8.2 Hz, 1H), 8.10 (d, *J* **=** 8.2 Hz, 1H), 8.06 (dd, *J* **=** 7.4, 1.2 Hz, 1H), 7.79 (t, *J* **=** 7.7 Hz, 1H), 7.75–7.69 (m, 1.1H), 7.68–7.60 (m, 2.2H), 7.51–7.26 (m, 1.7H), 3.29 (br s, 1.6H), 3.22 (br s, 0.6H), 3.00 (d, *J* **=** 7.3 Hz, 2H), 2.78 (s, 3H), 1.85–1.72 (m, 2H), 1.69 (d, *J* **=** 12.3 Hz, 2H), 1.63–1.45 (m, 2H), 1.00–0.89 (m, 2H), 0.85–0.74 (m, 2H). ^1^H NMR (600 MHz, DMSO‐*d*_6_/D_2_O 9:1 v/v) δ 8.51 (d, *J* **=** 8.8 Hz, 1H), 8.20 (d, *J* **=** 8.3 Hz, 1H), 8.13–8.08 (m, 1H), 8.05 (d, *J* **=** 8.1 Hz, 1H), 8.02 (dd, *J* **=** 7.4, 1.2 Hz, 1H), 7.78 (t, *J* **=** 7.9 Hz, 1H), 7.72–7.66 (m, 1H), 7.65–7.58 (m, 2.3H), 7.45–7.27 (m, 1.6H), 3.26 (br s, 1.3H), 3.18 (br s, 0.7H), 2.96 (d, *J* **=** 7.3 Hz, 2H), 2.74 (s, 3H), 1.78–1.59 (m, 4H), 1.51 (s, 2H), 0.90 (q, *J* **=** 12.3, 11.8 Hz, 2H), 0.75 (q, *J* **=** 12.4 Hz, 2H). ^13^C NMR (150 MHz, DMSO‐*d*_*6*_) δ 162.85 (Cq^qz^), 161.98 (Cq^qz^), 158.56 (carbonyl^TFA^), 153.42 (Cq^qz^), 139.50 (Cq^qz^), 135.56 (CH^qz^), 135.16 (CH^qz^), 134.04 (Cq^naph^), 134.02 (CH^naph^), 133.86 (Cq^naph^), 129.04 (CH^naph^), 128.83 (CH^naph^), 128.06 (CH^naph^), 128.02 (Cq^naph^), 126.93 (CH^naph^), 124.83 (CH^qz^), 124.63 (2 × CH^naph^), 124.01 (CH^qz^), 117.16 (CH^qz^), 116.66 (q, *J* **=** 297.7 Hz, CF_3_), 116.53 (CH^qz^), 109.73 (Cq^qz^), 109.07 (Cq^qz^), 55.43 (CH_2_^cy‐exo^), 46.83 (CH_2_^cy‐exo^), 46.41 (CH_2_^cy‐exo^), 37.31 (CH^cy^), 36.75 (CH^cy^), 35.35 (CH^cy^), 34.64 (CH_3_), 29.51 (CH_2_^cy^), 29.46 (CH_2_^cy^). HRMS (ESI): *m/z* [M**+**H]^+^ calcd. for [C_27_H_32_N_5_O_2_S]^+^ 490.2271, found: 490.2278. RP‐HPLC (220 nm): >99% (*t*_R_ **=** 18.3 min, *k* **=** 6.0). C_27_H_31_N_5_O_2_S** ∙** C_2_HF_3_O_2_ (489.64** +** 114.02).

(1*r*,4*r*)‐4‐[(Naphthalene‐1‐sulfonamido)methyl]cyclohexane‐1‐carboxylic acid (**6**) [27]: 1‐Naphthalenesulfonyl chloride (**5**) (5.2 g, 22.90 mmol) in dry CH_2_Cl_2_ (21 mL) was added dropwise to a solution of *trans*‐4‐(aminomethyl)cyclohexane‐1‐carboxylic acid (**4**) (3.0 g, 19.08 mmol) in 1 N NaOH (45 mL) over a period of 10 min at rt. The mixture was stirred at rt overnight. 4 N HCl (10 mL) and water (50 mL) were added, and the white precipitate was filtered off and washed with water. Residual solvent was removed by lyophilization, yielding **6** as a white solid (6.7 g, 100%; note: the yield calculated based on the product mass was >100%, which is due to residual CH_2_Cl_2_). ^1^H NMR (400 MHz, DMSO‐*d*_*6*_) δ 11.66 (br s, 1H), 8.66 (d, *J* **=** 8.5 Hz, 1H), 8.21 (d, *J* **=** 8.1 Hz, 1H), 8.14‐8.02 (m, 2H), 7.95 (t, *J* **=** 6.0 Hz, 1H), 7.78–7.56 (m, 3H), 2.61 (t, *J* **=** 6.4 Hz, 2H), 2.00 (tt, *J* **=** 12.1, 3.6 Hz, 1H), 1.76 (dd, *J* **=** 13.6, 3.5 Hz, 2H), 1.60 (dd, *J* **=** 13.4, 3.4 Hz, 2H), 1.27–1.14 (m, 1H), 1.13–0.99 (m, 2H), 0.81–0.64 (m, 2H). ^13^C NMR (101 MHz, DMSO‐*d*_*6*_) δ 176.59, 135.88, 133.85, 133.57, 128.89, 128.27, 127.74, 127.55, 126.81, 124.76, 124.51, 48.46, 42.30, 36.78, 29.06 (2 carbon atoms), 28.08 (2 carbon atoms). MS (ESI): *m/z* (%) 348.1 (100) [M**+**H]^+^, 717.2 (27) [2 M+Na]^+^, 370.1 (24) [M+Na]^+^, 365.1 (16) [M**+**NH_4_]^+^. C_18_H_21_NO_4_S (347.43).

(1*r*,4*r*)‐4‐[(Naphthalene‐1‐sulfonamido)methyl]cyclohexane‐1‐carboxamide (**8**) [27]: **6** was dissolved in dry THF (20 mL) and triethylamine (2.8 mL, 20.25 mmol) was added. The mixture was cooled to −5°C (salt cooling bath). Ethyl chloroformate (**7**) (1.9 mL, 20.25 mmol) was dissolved in dry THF (2 mL) and added to the mixture in proportions (4 × 500 µL, 1 min time gap in between). Stirring was continued at −5°C for 45 min, followed by the addition of a 25% aqueous ammonia solution (20 mL) over a time period of 5 min. After stirring the formed suspension at rt for 5 h, water (100 mL) was added, and the precipitate was filtered off and washed with water. Residual solvent was removed by lyophilization to yield **8** as a white solid (4.9 g, 73%). ^1^H NMR (400 MHz, DMSO‐*d*_*6*_) δ 8.67 (d, *J* **=** 9.1 Hz, 1H), 8.21 (d, *J* **=** 8.2 Hz, 1H), 8.14–8.05 (m, 2H), 7.94 (t, *J* **=** 5.9 Hz, 1H), 7.74–7.59 (m, 3H), 7.10 (s, 1H), 6.60 (s, 1H), 2.61 (t, *J* **=** 6.4 Hz, 2H), 1.89 (tt, *J* **=** 12.1, 3.4 Hz, 1H), 1.67–1.49 (m, 4H), 1.25–1.00 (m, 3H), 0.78–0.58 (m, 2H). ^13^C NMR (101 MHz, DMSO‐*d*_*6*_) δ 177.10, 135.88, 133.84, 133.56, 128.89, 128.26, 127.73, 127.56, 126.80, 124.76, 124.50, 48.60, 43.54, 36.85, 29.34 (2 carbon atoms), 28.52 (2 carbon atoms). MS (ESI): *m/z* (%) 347.1 (100) [M**+**H]^+^, 369.1 (18) [M+Na]^+^, 364.2 (15) [M**+**NH_4_]^+^. C_18_H_22_N_2_O_3_S (346.45).

*N*‐{[(1*r*,4*r*)‐4‐(Aminomethyl)cyclohexyl]methyl}naphthalene‐1 sulfonamide (**9**) [27]: To a stirred suspension of **8** (4.7 g, 13.57 mmol) in dry THF (36 mL) was added dropwise to a 1 M borane THF complex solution (33.8 mL). The mixture was heated to reflux, and stirring was continued for 3 h. After cooling the mixture to 0°C (ice bath), water (8 mL) and 4 N HCl (34 mL) were added. MeOH (70 mL) was added and removed by rotary evaporation. The addition of MeOH (68 mL) followed by rotary evaporation of the organic solvent was repeated two more times. The aqueous phase was adjusted to an alkaline pH using KOH pellets. The mixture was transferred into a separating funnel, and **9** was extracted with CH_2_Cl_2_ (3 × 100 mL). The organic phases were combined, dried over Na_2_SO_4,_ and the volatiles were removed *in vacuo*. Purification by flash chromatography (gradient 0.0–2.0 CV: CH_2_Cl_2_/MeOH 90:10 v/v (isocratic), 2.0–6.0 CV: 90:10–60:40 v/v, 6.0–7.0 CV: 60:40 v/v (isocratic), 7.0–7.5 CV: 60:40–0:100 v/v) yielded **9** (2.6 g, 59%) as a pale‐yellow solid. ^1^H NMR (400 MHz, DMSO‐*d*_*6*_) δ 8.73–8.60 (m, 1H), 8.21 (d, *J* **=** 8.2 Hz, 1H), 8.14–8.02 (m, 2H), 7.77–7.54 (m, 3H), 3.55 (br s, 3H), 2.61 (d, *J* **=** 6.9 Hz, 2H), 2.28 (d, *J* **=** 6.4 Hz, 2H), 1.76–1.46 (m, 4H), 1.17 (br s, 1H), 1.01 (br s, 1H), 0.78–0.45 (m, 4H). ^13^C NMR (101 MHz, DMSO‐*d*_*6*_) δ 135.89, 133.83, 133.52, 128.87, 128.25, 127.70, 127.57, 126.78, 124.78, 124.48, 48.75, 48.16, 40.63, 37.68, 29.79 (2 carbon atoms), 29.72 (2 carbon atoms). MS (ESI): *m/z* (%) 333.2 (100) [M**+**H]^+^. C_18_H_24_N_2_O_2_S (332.46).

*tert*‐Butyl {2‐[(2‐chloroquinazolin‐4‐yl)amino]ethyl}(methyl)carbamate (**16**): **16** was prepared from *tert*‐butyl (2‐aminoethyl)(methyl)carbamate (225 mg, 1.29 mmol) and **10** (257 mg, 1.29 mmol) in 2‐propanol (3 mL) according to general procedure A, applying a reaction time of 4 h (DIPEA: 439 µL, 2.58 mmol). Purification by flash chromatography (gradient: 0.0–2.0 CV: CH_2_Cl_2_/MeOH 99:1 v/v (isocratic), 2.0–3.5 CV: 99:1–96.5:3.5 v/v, 3.5–6.0 CV: 96.5:3.5 v/v (isocratic), 6.0–7.0 CV: 96.5:3.5–98:2 v/v, 7.0–8.3 CV: 98:2 v/v (isocratic)) yielded **16** as a pale orange oil (420 mg, 97%). ^1^H NMR (400 MHz, DMSO‐*d*_*6*_) (two major rotamers with a ratio of approximately 4:1 were evident in the ^1^H and ^13^C NMR spectra) δ 8.84 (br s, 0.7H), 8.76 (br s, 0.3H), 8.28–8.12 (m, 1H), 7.82–7.70 (m, 1H), 7.67–7.57 (m, 1H), 7.57–7.45 (m, 1H), 3.71–3.55 (m, 2H), 3.55–3.40 (m, 2H), 2.92–2.74 (m, 3H), 1.22 (s, 3H, interfering with the signals of EtOAc), 1.01 (s, 6H). ^13^C NMR (101 MHz, DMSO‐*d*_*6*_) δ 161.31, 156.95, 154.74, 150.27, 133.60, 126.65, 125.98, 123.03, 113.70, 78.34, 77.97, 46.91, 46.32, 38.55, 34.42, 33.49, 27.54 (3 carbon atoms). MS (ESI): *m/z* (%) 337.1, 339.1 (100) [M**+**H]^+^. C_16_H_21_ClN_4_O_2_ (336.82).

*tert*‐Butyl {3‐[(2‐chloroquinazolin‐4‐yl)amino]propyl}(methyl)carbamate (**17**): **17** was prepared from *tert*‐butyl (3‐aminopropyl)(methyl)carbamate (312 mg, 1.66 mmol) and **10** (300 mg, 1.51 mmol) in 2‐propanol (3 mL) according to general procedure A applying a reaction time of 5 h (DIPEA: 513 µL, 3.01 mmol). Purification by flash chromatography (gradient: 0.0–2.0 CV: petroleum ether/EtOAc 90:10 v/v (isocratic), 2.0–5.0 CV: 90:10–40:60 v/v, 5.0–6.0 CV: 40:60 v/v (isocratic), 6.0–8.0 CV: 40:60–0:100 v/v) yielded **17** as a colorless oil (410 mg, 78%). ^1^H NMR (400 MHz, DMSO‐*d*_*6*_) (two major rotamers with a ratio of approximately 1.5:1 were evident in the ^1^H NMR spectrum) δ 8.69 (t, *J* **=** 5.3 Hz, 1H), 8.24 (d, *J* **=** 8.2 Hz, 1H), 7.83–7.74 (m, 1H), 7.61 (dd, *J* **=** 8.4, 1.2 Hz, 1H), 7.56–7.48 (m, 1H), 3.48 (q, *J* **=** 6.7 Hz, 2H), 3.26 (t, *J* **=** 7.0 Hz, 2H), 2.80 (s, 3H), 1.85 (br s, 2H), 1.37 (s, 3.8H), 1.28 (s, 5.2H). ^13^C NMR (101 MHz, DMSO‐*d*_*6*_) δ 161.06, 157.00, 154.93, 150.21, 133.53, 126.62, 125.99, 123.02, 113.57, 78.34, 46.10, 38.57, 33.71, 27.92 (3 carbon atoms), 26.45. MS (ESI): *m/z* (%) 351.2, 535.2 (100) [M**+**H]^+^. C_17_H_23_ClN_4_O_2_ (350.85).

*tert*‐Butyl {2‐[(2‐chloroquinazolin‐4‐yl)amino]ethyl}carbamate (**18**) [27]: **18** was prepared from *tert*‐butyl (2‐aminoethyl)carbamate (402 mg, 2.51 mmol) and **10** (508 mg, 2.55 mmol) in 2‐propanol (3 mL) according to general procedure A applying a reaction time of 2 h (DIPEA: 868 µL, 5.10 mmol). Purification by flash chromatography (gradient: 0.0–1.0 CV: petroleum ether/EtOAc 95:5 v/v (isocratic), 1.0–4.0 CV: 95:5–80:20 v/v, 4.0–6.0 CV: 80:20 v/v (isocratic), 6.0–8.0 CV: 80:20–50:50 v/v, 8.0–10.0 CV: 50:50 v/v (isocratic), 10.0–11.0 CV: 50:50–0:100 v/v) yielded **18** as a pale orange oil (687 mg, 83%). ^1^H NMR (400 MHz, DMSO‐*d*_*6*_) δ 8.72 (t, *J* **=** 4.9 Hz, 1H), 8.22 (d, *J* **=** 8.2 Hz, 1H), 7.82–7.71 (m, 1H), 7.66–7.58 (m, 1H), 7.58–7.47 (m, 1H), 6.97 (t, *J* **=** 5.9 Hz, 1H), 3.58–3.49 (m, 2H), 3.24 (q, *J* **=** 6.2 Hz, 2H), 1.35 (s, 9H). ^13^C NMR (101 MHz, DMSO‐*d*_*6*_) δ 161.30, 156.92, 155.80, 150.23, 133.54, 126.57, 125.94, 123.19, 113.65, 77.69, 41.07, 38.72, 28.18 (3 carbon atoms). MS ESI): *m/z* (%) 323.1, 325.1 (100) [M**+**H]^+^. C_15_H_19_ClN_4_O_2_ (322.79).

*tert*‐Butyl {3‐[(2‐chloroquinazolin‐4‐yl)amino]propyl}carbamate (**19**) [43]: **19** was prepared from *tert*‐butyl (3‐aminopropyl)carbamate (190 mg, 1.09 mmol) and **10** (200 mg, 1.00 mmol) in 2‐propanol (2 mL) according to general procedure A applying a reaction time of 2 h (DIPEA: 342 µL, 2.01 mmol). Purification by flash chromatography (gradient: 0.0–1.0 CV: petroleum ether/EtOAc 95:5 v/v (isocratic), 1.0–4.0 CV: 95:5–80:20 v/v, 4.0–6.0 CV: 80:20 v/v (isocratic), 6.0–10.7 CV: 80:20–50:50, 10.7–14.0 CV: 50:50–0:100 v/v) yielded **19** as a colorless oil (355 mg, 105%; note: the yield calculated based on the product mass was >100%, which is due to residual solvent and **10**). ^1^H NMR (400 MHz, DMSO‐*d*_6_) δ 8.66 (t, *J* **=** 5.5 Hz, 1H), 8.24 (d, *J* **=** 7.9 Hz, 1H), 7.83–7.73 (m, 1H), 7.61 (dd, *J* **=** 8.3, 1.1 Hz, 1H), 7.57–7.48 (m, 1H), 6.86 (t, *J* **=** 5.9 Hz, 1H), 3.50 (q, *J* **=** 6.6 Hz, 2H), 3.02 (q, *J* **=** 6.6 Hz, 2H), 1.76 (p, *J* **=** 7.0 Hz, 2H), 1.36 (s, 9H). ^13^C NMR (101 MHz, DMSO‐*d*_6_) δ 161.05, 156.97, 155.59, 150.19, 133.53, 126.61, 126.00, 123.03, 113.55, 77.50, 38.60, 37.66, 28.65, 28.23 (3 carbon atoms). MS (ESI): *m/z* (%) 337.1, 339.1 (100) [M**+**H]^+^, 281.1, 283.3 (6) [M–C_4_H_8_ **+**H]^+^. C_16_H_21_ClN_4_O_2_ (336.82).

*tert*‐Butyl {4‐[(2‐chloroquinazolin‐4‐yl)amino]butyl}carbamate (**20**) [44]: **20** was prepared from *tert*‐butyl (4‐aminobutyl)carbamate (238 mg, 1.27 mmol) and **10** (252 mg, 1.27 mmol) in 2‐propanol (3 mL) according to general procedure A applying a reaction time of 1 h (DIPEA: 431 µL, 2.53 mmol). Purification by flash chromatography (gradient: 0.0–1.5 CV: petroleum ether/EtOAc 90:10 v/v (isocratic), 1.5–5.5 CV: 90:10–30:70 v/v, 5.5–7.6 CV: 30:70 v/v (isocratic), 7.6–8.6 CV: 30:70–0:100 v/v) yielded **20** as a white resin (360 mg, 81%). ^1^H NMR (400 MHz, DMSO‐*d*_*6*_) δ 8.70 (t, *J* **=** 5.6 Hz, 1H), 8.26 (d, *J* **=** 8.0 Hz, 1H), 7.83–7.72 (m, 1H), 7.63–7.57 (m, 1H), 7.56–7.46 (m, 1H), 6.79 (t, *J* **=** 5.8 Hz, 1H), 3.50 (q, *J* **=** 6.6 Hz, 2H), 2.96 (q, *J* **=** 6.6 Hz, 2H), 1.69–1.55 (m, 2H), 1.54–1.40 (m, 2H), 1.36 (s, 9H). ^13^C NMR (101 MHz, DMSO‐*d*_*6*_) δ 161.04, 157.05, 155.59, 150.23, 133.50, 126.60, 125.95, 123.07, 113.54, 77.34, 40.58, 28.24 (3 carbon atoms), 27.04, 25.62. Note: the carbon signal of one CH_2_‐group was not apparent due to interference with the solvent residual peak. MS (ESI): *m/z* (%) 351.2, 353.2 (100) [M**+**H]^+^. C_17_H_23_ClN_4_O_2_ (350.85).

*tert*‐Butyl methyl[2‐({2‐[({(1*r*,4*r*)‐4‐[(naphthalene‐1‐sulfonamido)methyl]cyclohexyl}methyl)amino]quinazolin‐4‐yl}amino)ethyl]carbamate (**21**): **21** was prepared from **16** (210 mg, 623.48 µmol) and **9** (207 mg, 623.48 µmol) in 1‐butanol (4 mL) according to general procedure B. Purification by flash chromatography (gradient: 0.0–1.2 CV: CH_2_Cl_2_/MeOH 96:4 v/v (isocratic), 1.2–2.0 CV: 96:4–93.5:6.5 v/v, 2.0–3.3 CV: 93.5:6.5 v/v (isocratic), 3.3–3.8 CV: 93.5:6.5–90:10 v/v, 3.8–6.2 CV: 90:10 v/v (isocratic)) gave **21** as a white solid (187 mg, 47%). ^1^H NMR (400 MHz, DMSO‐*d*_6_) (two major rotamers with a ratio of approximately 1.5:1 were evident in the ^1^H NMR spectrum) δ 12.45 (br s, 0.7H), 9.58 (br s, 0.7H), 9.07 (br s, 0.1H), 8.66 (d, *J* **=** 8.5 Hz, 1H), 8.20 (d, *J* **=** 8.2 Hz, 2H), 8.12–8.03 (m, 2.4H), 7.95 (t, *J* **=** 5.9 Hz, 1H), 7.82–7.57 (m, 4.3H), 7.46–7.28 (m, 1.8H), 3.65 (br s, 1.7H), 3.57–3.38 (m, 2.3H), 3.32 (s, 2H, interfering with the water signal), 2.84 (s, 3H), 2.62 (t, *J* **=** 6.3 Hz, 2H), 1.76–1.55 (m, 4H), 1.44 (br s, 1H), 1.35–1.13 (m, 5H), 1.02 (s, 5H), 0.88–0.58 (m, 4H). MS (ESI): *m/z* (%) 633.3 (100) [M**+**H]^+^. C_34_H_44_N_6_O_4_S (632.82).

*tert*‐Butyl methyl[3‐({2‐[({(1*r*,4*r*)‐4‐[(naphthalene‐1‐sulfonamido)methyl]cyclohexyl}methyl)amino]quinazolin‐4‐yl}amino)propyl]carbamate (**22**): **22** was prepared from **17** (205 mg, 584.30 µmol) and **9** (196 mg, 589.54 µmol) in 1‐butanol (4 mL) according to general procedure B. Purification by flash chromatography (gradient: 0.0–2.0 CV: CH_2_Cl_2_/MeOH 98.5:1.5 v/v (isocratic), 2.0–3.5 CV: 98.5–96:4 v/v, 3.5–6.8 96:4 v/v (isocratic), 6.8–8.3 CV: 96:4–92:8 v/v, 8.3–9.0 CV: 92:8 v/v (isocratic), 9.0–10.0 CV: 92:8–90:10 v/v) gave **22** as a white solid (240 mg, 64%). ^1^H NMR (400 MHz, DMSO‐*d*_*6*_) (two major rotamers with a ratio of approximately 1.5:1 were evident in the ^1^H NMR spectrum) δ 12.38 (br s, 0.8H), 9.55 (br s, 0.7H), 9.00 (br s, 0.1H), 8.66 (d, *J* **=** 8.5 Hz, 1H), 8.24 (br s, 0.8H), 8.20 (d, *J* **=** 8.3 Hz, 1.2H), 8.12–7.98 (m, 2.4H), 7.94 (t, *J* **=** 5.6 Hz, 1H), 7.82–7.53 (m, 4.2H), 7.37 (d, *J* **=** 10.3 Hz, 1.8H), 3.60–3.48 (m, 2H), 3.24 (t, *J* **=** 7.0 Hz, 4H), 2.77 (s, 3H), 2.61 (t, *J* **=** 6.3 Hz, 2H), 1.92–1.77 (m, 2H), 1.75–1.55 (m, 4H), 1.44 (s, 1H), 1.37–1.18 (m, 10H), 0.84–0.62 (m, 4H). MS (ESI): *m/z* (%) 647.3 (100) [M**+**H]^+^. C_35_H_46_N_6_O_4_S (646.85).

*tert*‐Butyl [2‐({2‐[({(1*r*,4*r*)‐4‐[(naphthalene‐1‐sulfonamido)methyl]cyclohexyl}methyl)amino]quinazolin‐4‐yl}amino)ethyl]carbamate (**23**) [27]: **23** was prepared from **18** (244 mg, 755.90 µmol) and **9** (243 mg, 730.91 µmol) in 1‐butanol (6 mL) according to general procedure B. Purification by flash chromatography (gradient: 0.0–3.0 CV: CH_2_Cl_2_/MeOH 98:2 v/v (isocratic), 3.0–5.0 CV: 98:2–96:4 v/v, 5.0–6.0 CV: 96:4 v/v (isocratic), 6.0–7.3 CV: 96:4–90:10) gave **23** as a white solid (270 mg, 60%). ^1^H NMR (400 MHz, DMSO‐*d*_6_) δ 12.66 (br s, 1H), 9.61 (s, 1H), 8.66 (d, *J* **=** 8.5 Hz, 1H), 8.38–8.00 (m, 4H, interfering with the water signal), 7.96 (t, *J* **=** 5.9 Hz, 1H), 7.83–7.56 (m, 4H), 7.45–7.25 (m, 2H), 7.00 (br s, 1H), 3.61–3.49 (m, 2H), 3.44–3.14 (m, 4H, interfering with the water signal), 2.61 (t, *J* **=** 6.3 Hz, 2H), 1.74–1.51 (m, 4H), 1.41 (br s, 1H), 1.37–1.23 (m, 9H), 1.21–1.14 (m, 1H), 0.92–0.53 (m, 4H). ^1^H NMR (500 MHz, DMSO‐*d*_6_/D_2_O 9:1 v/v) δ 8.61 (d, *J* **=** 8.6 Hz, 1H), 8.23–7.98 (m, 4H), 7.80–7.55 (m, 4H), 7.42–7.25 (m, 2H), 3.57–3.42 (m, 2H), 3.30–3.08 (m, 4H), 2.58 (d, *J* **=** 6.8 Hz, 2H), 1.73–1.51 (m, 4H), 1.42 (s, 1H), 1.32–1.14 (m, 9H), 1.09 (br s, 1H), 0.84–0.50 (m, 4H). ^13^C NMR (101 MHz, DMSO‐*d*_6_) δ 160.01, 155.67, 153.41, 139.22, 135.87, 134.97, 133.83, 133.52, 128.86, 128.25, 127.70, 127.57, 126.78, 124.78, 124.48, 124.34, 123.69, 116.72, 109.64, 77.73, 48.63, 46.35, 41.46, 38.33, 37.65, 37.36, 29.55 (2 carbon atoms), 29.48 (2 carbon atoms), 28.17 (3 carbon atoms). MS (ESI): *m/z* (%) 619.3 (100) [M**+**H]^+^. C_33_H_42_N_6_O_4_S (618.80).

*tert*‐Butyl [3‐({2‐[({(1*r*,4*r*)‐4‐[(naphthalene‐1‐sulfonamido)methyl]cyclohexyl}methyl)amino]quinazolin‐4‐yl}amino)propyl]carbamate (**24**): **24** was prepared from **19** (151 mg, 448.31 µmol) and **9** (149 mg, 448.31 µmol) in 1‐butanol (6–7 mL) according to general procedure B applying a reaction time of 25 min instead of 1 h. As HPLC analysis revealed incomplete conversion of the starting material, microwave irradiation was repeated (20 min, 150°C). Purification by flash chromatography (gradient: 0.0–3.0 CV: CH_2_Cl_2_/MeOH 98:2 v/v (isocratic), 3.0–5.0 CV: 98:2–95:5 v/v, 5.0–8.0 CV: 95:5 v/v (isocratic), 8.0–9.5 CV: 95:5–92:8 v/v, 9.5–10.0 CV: 92:8 v/v (isocratic), 10.0–10.5 CV: 92:8–90:10 v/v) gave the title compound as a white solid (136 mg, 48%). MS (ESI): *m/z* (%) 633.2 (100) [M**+**H]^+^. C_34_H_44_N_6_O_4_S (632.82).

*tert*‐Butyl [4‐({2–[({(1*r*,4*r*)‐4‐[(naphthalene‐1‐sulfonamido)methyl]cyclohexyl}methyl)amino]quinazolin‐4‐yl}amino)butyl]carbamate (**25**) [45]: **25** was prepared from **20** (103 mg, 293.58 µmol) and **9** (98 mg, 294.77 µmol) in 1‐butanol (4 mL) according to general procedure B. Purification by flash chromatography (gradient: 0.0–3.0 CV: CH_2_Cl_2_/MeOH 98:2 v/v (isocratic), 3.0–5.0 CV: 98:2–95:5 v/v, 5.0–8.0 CV: 95:5 (isocratic), 8.0–9.5 CV: 95:5–92:8 v/v, 9.5–11.0 CV: 92:8 v/v (isocratic), 11.0–12.0 CV: 92:8–90:10 v/v, 12.0–14.0 CV: 90:10 v/v (isocratic)) gave **25** as a white solid (110 mg, 58%). ^1^H NMR (500 MHz, DMSO‐*d*_6_) (two major rotamers with a ratio of approximately 2.3:1 were evident in the ^1^H spectrum) δ 12.57 (br s, 1H), 9.63 (s, 0.7H), 9.19 (br s, 0.2H), 8.66 (d, *J* **=** 8.7 Hz, 1H), 8.29 (d, *J* **=** 8.2 Hz, 1H), 8.20 (d, *J* **=** 8.2 Hz, 1H), 8.14–8.03 (m, 2.8H), 7.95 (t, *J* **=** 5.8 Hz, 1H), 7.79–7.67 (m, 2H), 7.67–7.59 (m, 2H), 7.49–7.22 (m, 1.8H), 6.80 (br s, 0.8H), 3.62–3.48 (m, 2H), 3.25 (t, *J* **=** 6.3 Hz, 2H), 3.00–2.86 (m, 1.7H), 2.61 (t, *J* **=** 6.3 Hz, 2H), 1.77–1.52 (m, 6H), 1.50–1.38 (m, 3H), 1.34 (s, 9H), 1.22 (br s, 1H), 0.87–0.75 (m, 2H), 0.74–0.60 (m, 2H). ^13^C NMR (126 MHz, DMSO‐*d*_6_) δ 159.65, 155.58, 153.24, 139.03, 135.85, 134.98, 133.84, 133.54, 128.87, 128.27, 127.71, 127.57, 126.79, 124.78, 124.49, 124.19, 123.86, 116.72, 109.58, 77.37, 48.62, 46.49, 40.90, 37.51 (2 carbon atoms), 37.39, 29.62 (2 carbon atoms), 29.53 (2 carbon atoms), 28.24 (3 carbon atoms), 27.05, 25.53. MS (ESI): *m/z* (%) 647.3 (100) [M**+**H]^+^. C_35_H_46_N_6_O_4_S (646.85).

*N*‐[((1*r*,4*r*)‐4‐{[(4‐{[2‐(Methylamino)ethyl]amino}quinazolin‐2‐yl)amino]methyl}cyclohexyl)methyl]naphthalene‐1‐sulfonamide bis(hydrotrifluoroacetate) (**26**): To synthesize **26** on a larger scale for analytical purposes, **21** (186 mg, 293.92 µmol) was dissolved in 2 N HCl in MeOH (2.8 mL) according to general procedure C giving a colorless oil, which was subjected to preparative HPLC (system 2, gradient: 0–30 min: 0.1% aq. TFA/acetonitrile 73:27–55:45, *t*_R_ **=** 10 min) yielding **26** as a white fluffy solid (120 mg, 54%). To find the optimal conditions for the synthesis of the radioligand [^3^H]**26** (by [^3^H]methylation of **28**), several test reactions (methylation of **28**) were performed. The following condition proved to be the most convenient: **28** (4.9 mg, 6.56 µmol) and K_2_CO_3_ (2.8 mg, 20.26 µmol) were suspended in acetonitrile (100 µL) and the mixture was stirred at rt for 25 min. **2** (11 mg, 50.6 µmol) was dissolved in acetonitrile (50 µL), 1.3 µL (1.31 µmol) of this solution were added to the solution of **28**, and stirring was continued at rt. The reaction was monitored via HPLC. ^1^H NMR (500 MHz, DMSO‐*d*_*6*_) (two major rotamers with a ratio of approximately 4:1 were evident in the ^1^H and ^13^C NMR spectra) δ 13.09 (s, 0.8H), 11.87 (s, 0.2H), 9.63 (t, *J* **=** 5.6 Hz, 0.8H), 9.49 (br s, 0.2H), 8.87–8.69 (m, 3H), 8.66 (d, *J* **=** 8.5 Hz, 1H), 8.24–8.16 (m, 2H), 8.08 (t, *J* **=** 7.8 Hz, 2H), 7.95 (t, *J* **=** 5.9 Hz, 1H), 7.80 (t, *J* **=** 7.7 Hz, 1H), 7.76–7.68 (m, 1.2H), 7.68–7.59 (m, 2H), 7.48–7.34 (m, 1.8H), 3.94–3.83 (m, 1.6H), 3.82 (br s, 0.4H, interfering with the water signal), 3.35–3.12 (m, 4H), 2.61 (br s, 5H), 1.78–1.57 (m, 4H), 1.54–1.35 (m, 1H), 1.30–1.17 (m, 1H), 0.91–0.77 (m, 2H), 0.77–0.66 (m, 2H). ^1^H NMR (500 MHz, DMSO‐*d*_*6*_/D_2_O 9:1 v/v) δ 8.62 (d, *J* **=** 8.6 Hz, 1H), 8.19 (d, *J* **=** 8.2 Hz, 1H), 8.12 (d, *J* **=** 8.3 Hz, 1H), 8.06 (t, *J* **=** 8.5 Hz, 2H), 7.80 (t, *J* **=** 7.4 Hz, 1H), 7.72–7.66 (m, 1.3H), 7.66–7.58 (m, 2H), 7.49–7.33 (m, 1.7H), 3.85 (br s, 1.4H), 3.80 (br s, 0.6H), 3.33–3.10 (m, 4H), 2.63–2.55 (m, 5H), 1.73–1.50 (m, 4H), 1.41 (br s, 1H), 1.22 (br s, 1H), 0.88–0.63 (m, 4H). ^13^C NMR (126 MHz, DMSO‐*d*_*6*_) δ 160.59 (Cq^qz^), 158.93 (q, *J* **=** 31.9 Hz, carbonyl^TFA^), 153.15 (Cq^qz^), 139.14 (Cq^qz^), 135.85 (Cq^naph^), 135.37 (CH^qz^), 133.85 (Cq^naph^), 133.55 (CH^naph^), 128.89 (CH^naph^), 128.24 (CH^naph^), 127.73 (CH^naph^), 127.56 (Cq^naph^), 126.81 (CH^naph^), 124.77 (CH^naph^), 124.50 (CH^naph^), 124.39 (CH^qz^), 123.94 (CH^qz^), 116.97 (q, *J* **=** 298.4 Hz, CF_3_), 116.49 (CH^qz^), 109.67 (Cq^qz^), 48.61 (CH_2_^cy‐exo^), 47.47 (CH_2_^linker^), 46.68 (CH_2_^linker^), 46.50 (CH_2_^cy‐exo^), 37.59 (CH_2_^linker^), 37.49 (CH^cy^), 37.43 (CH^cy^), 36.69 (CH_2_^linker^), 32.56 (CH_3_), 29.55 (CH_2_^cy^), 29.40 (CH_2_^cy^). HRMS (ESI): *m/z* [M**+**H]^+^ calcd. for [C_29_H_37_N_6_O_2_S]^+^ 533.2694, found: 533.2699. RP‐HPLC (220 nm): 98% (*t*_R_ **=** 14.8 min, *k* **=** 4.7). C_29_H_36_N_6_O_2_S** ∙** C_4_H_2_F_6_O_4_ (532.71** +** 228.04).

*N*‐[((1*r*,4*r*)‐4‐{[(4‐{[3‐(Methylamino)propyl]amino}quinazolin‐2‐yl)amino]methyl}cyclohexyl)methyl]naphthalene‐1‐sulfonamide bis(hydrotrifluoroacetate) (**27**): **22** (230 mg, 355.57 µmol) was dissolved in 2 N HCl in MeOH (3.6 mL) according to general procedure C giving a yellowish foam, which was subjected to preparative HPLC (system 2, gradient: 0–30 min: 0.1% aq. TFA/acetonitrile 85:25–58:42, *t*_R_ **=** 15 min) yielding **27** as a white fluffy solid (123 mg, 45%). ^1^H NMR (500 MHz, DMSO‐*d*_*6*_) (two major rotamers with a ratio of approximately 4:1 were evident in the ^1^H NMR spectrum) δ 13.03 (s, 0.8H), 11.74 (s, 0.2H), 9.55 (s, 0.8H), 9.34 (br s, 0.2H), 8.71 (br s, 0.9H), 8.66 (d, *J* **=** 8.5 Hz, 1.1H), 8.60 (s, 2H), 8.24–8.16 (m, 2H), 8.08 (t, *J* **=** 7.4 Hz, 2H), 7.95 (t, *J* **=** 5.9 Hz, 1H), 7.79 (t, *J* **=** 7.5 Hz, 1H), 7.73–7.67 (m, 1.1H), 7.67–7.60 (m, 2.1H), 7.45–7.35 (m, 1.8H), 3.63 (br s, 2H), 3.28 (t, *J* **=** 6.2 Hz, 1.5H), 3.19 (br s, 0.5H), 2.98 (br s, 2H), 2.62 (t, *J* **=** 6.3 Hz, 2H), 2.56 (s, 3H), 2.02–1.92 (m, 2H), 1.78–1.56 (m, 4H), 1.45 (br s, 1H), 1.31–1.16 (m, 1H), 0.92–0.62 (m, 4H). ^1^H NMR (500 MHz, DMSO‐*d*_*6*_/D_2_O 9:1 v/v) δ 8.61 (d, *J* **=** 8.5, 1H), 8.18 (d, *J* **=** 8.2 Hz, 1H), 8.12 (d, *J* **=** 8.2 Hz, 1H), 8.06 (t, *J* **=** 8.4 Hz, 2H), 7.78 (t, *J* **=** 7.8 Hz, 1H), 7.72–7.66 (m, 1H), 7.66–7.58 (m, 2.3H), 7.44–7.32 (m, 1.7H), 3.65–3.52 (m, 2H), 3.25 (d, *J* **=** 6.5 Hz, 1.4H), 3.20–3.10 (m, 0.6H), 2.99–2.87 (m, 2H), 2.59 (d, *J* **=** 6.7 Hz, 2H), 2.53 (s, 3H), 2.01–1.88 (m, 2H), 1.75–1.56 (m, 4H), 1.43 (br s, 1H), 1.21 (br s, 1H), 0.86–0.64 (m, 4H). ^13^C NMR (126 MHz, DMSO‐*d*_*6*_) δ 160.04 (Cq^qz^), 158.83 (q, *J* **=** 31.2 Hz, carbonyl^TFA^), 153.18 (Cq^qz^), 139.22 (Cq^qz^), 135.85 (Cq^naph^), 135.23 (CH^qz^), 133.85 (Cq^naph^), 133.55 (CH^naph^), 128.88 (CH^naph^), 128.23 (CH^naph^), 127.73 (CH^naph^), 127.56 (Cq^naph^), 126.81 (CH^naph^), 124.76 (CH^naph^), 124.50 (CH^naph^), 124.04 (CH^qz^), 123.97 (CH^qz^), 117.13 (q, *J* **=** 299.6 Hz, CF_3_), 116.60 (CH^qz^), 109.54 (Cq^qz^), 48.60 (CH_2_^cy‐exo^), 46.58 (CH_2_^cy‐exo^), 46.20 (CH_2_^linker^), 38.46 (CH_2_^linker^), 37.52 (CH^cy^), 37.45 (CH^cy^), 32.44 (CH_3_), 29.63 (CH_2_^cy^), 29.53 (CH_2_^cy^), 24.83 (CH_2_^linker^). HRMS (ESI): *m/z* [M**+**H]^+^ calcd. for [C_30_H_39_N_6_O_2_S]^+^ 547.2850, found: 547.2860. RP‐HPLC (220 nm): 97% (*t*_R_ **=** 15.0 min, *k* **=** 4.8). C_30_H_38_N_6_O_2_S** ∙** C_4_H_2_F_6_O_4_ (546.73** +** 228.04).

*N*‐({(1*r*,4*r*)‐4‐[({4‐[(2‐Aminoethyl)amino]quinazolin‐2‐yl}amino)methyl]cyclohexyl}methyl)naphthalene‐1‐sulfonamide bis(hydrotrifluoroacetate) (**28**) [27]: **23** (270 mg, 436.33 µmol) was dissolved in 2 N HCl in MeOH (2 mL) according to general procedure C. Stirring was continued for 15 h. Evaporation of the volatiles yielded **28** as a brown foam (198 mg, 77%). For the use of **28** as a starting material for other Y_5_R ligands, it was used without further purification. For the use as a labeling precursor for radioligand synthesis, elemental analysis, and pharmacological characterization, a small portion (ca. 50 mg) was purified by preparative HPLC (system 1, gradient: 0–30 min: 0.1% aq. TFA/acetonitrile 80:20–50:50, *t*_R_ **=** 16 min), giving a white fluffy solid. ^1^H NMR (500 MHz, DMSO‐*d*_6_) (two major rotamers with a ratio of approximately 4:1 were evident in the ^1^H and ^13^C NMR spectra) δ 13.16 (s, 0.8H), 11.87 (s, 0.2H), 9.58 (t, *J* **=** 5.6 Hz, 0.8H), 9.43 (br s, 0.2H), 8.79 (t, *J* **=** 5.8 Hz, 0.8H), 8.73 (br s, 0.2H), 8.66 (d, *J* **=** 8.5 Hz, 1H), 8.23–8.15 (m, 2H), 8.13–7.97 (m, 5H), 7.96 (t, *J* **=** 5.9 Hz, 1H), 7.79 (t, *J* **=** 7.7 Hz, 1H), 7.74‐7.68 (m, 1.2H), 7.67–7.60 (m, 2H), 7.51–7.32 (m, 1.8H), 3.86–3.73 (m, 2H, interfering with the water signal), 3.31 (t, *J* **=** 6.2 Hz, 1.6H), 3.23–3.11 (m, 2.4H), 2.62 (t, *J* **=** 6.2 Hz, 2H), 1.78–1.57 (m, 4H), 1.42 (br s, 1H), 1.31–1.17 (m, 1H), 0.91–0.64 (m, 4H). ^1^H NMR (500 MHz, DMSO‐*d*_6_/D_2_O 9:1 v/v) δ 8.62 (d, *J* **=** 8.6 Hz, 1H), 8.18 (d, *J* **=** 8.2 Hz, 1H), 8.11 (d, *J* **=** 7.9 Hz, 1H), 8.09–8.00 (m, 2H), 7.79 (t, *J* **=** 7.5 Hz, 1H), 7.71–7.56 (m, 3.3H), 7.50–7.33 (m, 1.7H), 3.80 (t, *J* **=** 5.6, 1.5H), 3.75 (br s, 0.5H), 3.28 (d, *J* **=** 6.5 Hz, 1.4H), 3.21–3.12 (m, 2.6H), 2.59 (d, *J* **=** 6.6 Hz, 2H), 1.75–1.53 (m, 4H), 1.40 (br s, 1H), 1.22 (br s, 1H), 0.86–0.75 (m, 2H), 0.75–0.62 (m, 2H). ^13^C NMR (126 MHz, DMSO‐*d*_6_) δ 160.62 (Cq^qz^), 160.11 (Cq^qz^), 159.02 (q, *J* **=** 32.0 Hz, carbonyl^TFA^), 153.19 (Cq^qz^), 139.19 (Cq^qz^), 135.86 (Cq^naph^), 135.35 (CH^qz^), 133.86 (Cq^naph^), 133.56 (CH^naph^), 128.90 (CH^naph^), 128.25 (CH^naph^), 127.74 (CH^naph^), 127.58 (Cq^naph^), 126.82 (CH^naph^), 124.78 (CH^naph^), 124.51 (CH^naph^), 124.34 (CH^qz^), 124.08 (CH^qz^), 123.93 (CH^qz^), 116.95 (q, *J* **=** 297.7 Hz, CF_3_), 116.48 (CH^qz^), 109.67 (Cq^qz^), 48.63 (CH_2_^cy‐exo^), 46.46 (CH_2_^cy‐exo^), 38.92 (CH_2_^linker^), 38.17 (CH_2_^linker^), 37.66 (CH^cy^), 37.59 (CH_2_^linker^), 37.44 (CH^cy^), 36.68 (CH^cy^), 29.57 (CH_2_^cy^), 29.54 (CH_2_^cy^), 29.40 (CH_2_^cy^). Note: the carbon signal of one CH_2_‐group was not apparent due to interference with the solvent residual peak. HRMS (ESI): *m/z* [M**+**H]^+^ calcd. for [C_28_H_35_N_6_O_2_S]^+^ 519.2537, found: 519.2538. Anal. calcd. for C_28_H_34_N_6_O_2_ **∙** H_4_O_2_ **∙** C_4_H_2_F_6_O_4_: C 49.10, H 5.15, N 10.74, S 4.10. Found: C 49.45, H 5.12, N 10.53, S 4.38. UV‐Vis (0.1% aq. TFA/acetonitrile 1.3:1 v/v): λ_max_ **=** 225 nm. RP‐HPLC (220 nm): 97% (*t*_R_ **=** 14.6 min, *k* **=** 4.6). C_28_H_34_N_6_O_2_S** ∙** C_4_H_2_F_6_O_4_ (518.68** +** 228.04).

*N*‐({(1*r*,4*r*)‐4‐[({4‐[(3‐Aminopropyl)amino]quinazolin‐2‐yl}amino)methyl]cyclohexyl}methyl)naphthalene‐1‐sulfonamide bis(hydrotrifluoroacetate) (**29**): **24** (136 mg, 214.91 µmol) was dissolved in 2 N HCl in dioxane (2.2 mL) according to general procedure C. Stirring was continued overnight. Evaporation of the volatiles yielded **29** as a white foam (167 mg, 128%, note: the isolated foam contained residual solvent). For analytical data and pharmacological characterization, a small portion (ca. 110 mg) was purified by preparative HPLC (system 1, gradient: 0–30 min: 0.1% aq. TFA/acetonitrile 90:10–50:50, *t*_R_ **=** 22 min), yielding **29** as a white fluffy solid (64.0 mg, 46%). ^1^H NMR (600 MHz, DMSO‐*d*_*6*_) (two major rotamers with a ratio of approximately 2.3:1 were evident in the ^1^H and ^13^C NMR spectra) δ 12.90 (br s, 0.7H), 11.66 (s, 0.2H), 9.53 (s, 0.7H), 9.33 (s, 0.2H), 8.80–8.48 (m, 2H), 8.20 (t, *J* **=** 7.8 Hz, 2H), 8.08 (t, *J* **=** 7.4 Hz, 2H), 7.94 (t, *J* **=** 5.9 Hz, 1H), 7.88–7.75 (m, 4H), 7.73–7.68 (m, 1.2H), 7.68–7.59 (m, 2H), 7.47–7.37 (m, 1.7H), 3.69–3.57 (m, 2H), 3.28, (t, *J* **=** 5.6 Hz, 1.5H), 3.18 (s, 0.5H), 2.88 (br s, 2H), 2.62 (t, *J* **=** 6.5 Hz, 2H), 1.98–1.87 (m, 2H), 1.81–1.57 (m, 4H), 1.45 (br s, 1H), 1.31–1.19 (m, 1H), 0.89–0.64 (m, 4H). ^1^H NMR (600 MHz, DMSO‐*d*_*6*_/D_2_O 9:1 v/v) δ 8.61 (d, *J* **=** 8.6 Hz, 1H), 8.18 (d, *J* **=** 8.2 Hz, 1H), 8.12 (d, *J* **=** 8.2 Hz, 1H), 8.09–8.02 (m, 2H), 7.78 (t, *J* **=** 7.7 Hz, 1H), 7.71–7.67 (m, 1H), 7.67–7.58 (m, 2.3H), 7.45–7.35 (m, 1.7H), 3.64–3.56 (m, 2H), 3.25 (d, *J* **=** 6.6 Hz, 1.5H), 3.14 (br s, 0.5H), 2.85 (t, *J* **=** 7.8 Hz, 2H), 2.59 (d, *J* **=** 6.7 Hz, 2H), 1.96–1.87 (m, 2H), 1.73–1.54 (m, 4H), 1.44 (br s, 1H), 1.21 (s, 1H), 0.85–0.74 (m, 2H), 0.74–0.63 (m, 2H). ^13^C NMR (151 MHz, DMSO‐*d*_*6*_) δ 160.00 (Cq^qz^), 159.41 (Cq^qz^), 158.71 (carbonyl^TFA^), 153.10 (Cq^qz^), 139.15 (Cq^qz^), 135.85 (Cq^naph^), 135.23 (CH^qz^), 133.85 (Cq^naph^), 133.55 (CH^naph^), 128.89 (CH^naph^), 128.23 (CH^naph^), 127.73 (CH^naph^), 127.56 (Cq^naph^), 126.81 (CH^naph^), 124.76 (CH^naph^), 124.50 (CH^naph^), 124.01 (CH^qz^), 123.74 (CH^qz^), 117.33 (CH^qz^), 117.16 (q, *J* **=** 298.7 Hz), 116.65 (CH^qz^), 110.24 (Cq^qz^), 109.53 (Cq^qz^), 48.61 (CH_2_^cy‐exo^), 46.94 (CH_2_^cy‐exo^), 46.61 (CH_2_^cy‐exo^), 38.57 (CH_2_^linker^), 38.07 (CH_2_^linker^), 37.50 (CH^cy^), 37.45 (CH^cy^), 37.00 (CH_2_^linker^), 36.81 (CH_2_^linker^), 36.64 (CH_2_^linker^), 29.64 (CH_2_^cy^), 29.52 (CH_2_^cy^), 26.56 (CH_2_^linker^), 26.35 (CH_2_^linker^). HRMS (ESI): *m/z* [M**+**H]^+^ calcd. for [C_29_H_37_N_6_O_2_S]^+^ 533.2693, found: 533.2697. RP‐HPLC (220 nm): >99% (*t*_R_ **=** 13.4 min, *k* **=** 4.2). C_29_H_36_N_6_O_2_S** ∙** C_4_H_2_F_6_O_4_ (532.71** +** 228.04).

*N*‐({(1*r*,4*r*)‐4‐[({4‐[(4‐Aminobutyl)amino]quinazolin‐2‐yl}amino)methyl]cyclohexyl}methyl)naphthalene‐1‐sulfonamide bis(hydrotrifluoroacetate) (**30**) [45]: **25** (110 mg, 170.05 µmol) was dissolved in 2 N HCl in MeOH (1.7 mL) according to general procedure C. Evaporation of the volatiles yielded **30** as a white foam (110 mg, 104%; the isolated foam contained residual solvent). For analytical data and pharmacological characterization, a small portion (ca. 60 mg) was purified by preparative HPLC (system 1, gradient: 0–30 min: 0.1% aq. TFA/acetonitrile 80:20–50:50, *t*_R_ **=** 17 min), yielding a white fluffy solid (62.0 mg, 83%). ^1^H NMR (500 MHz, DMSO‐*d*_6_) (two major rotamers with a ratio of approximately 4:1 were evident in the ^1^H NMR spectrum) δ 12.89 (s, 0.8H), 11.64 (s, 0.2H), 9.55 (br s, 0.8H), 9.29 (br s, 0.2H), 8.75–8.57 (m, 2H), 8.26–8.17 (m, 2H), 8.08 (t, *J* **=** 7.6 Hz, 2H), 7.95 (t, *J* **=** 5.9 Hz, 1H), 7.89–7.73 (m, 4H), 7.72–7.60 (m, 3.3H), 7.49–7.31 (m, 1.7H), 3.66–3.50 (m, 2H, interfering with the water signal), 3.26 (t, *J* **=** 6.2 Hz, 1.6H), 3.18 (br s, 0.4H), 2.87–2.75 (m, 2H), 2.62 (t, *J* **=** 6.3 Hz, 2H), 1.86–1.54 (m, 8H), 1.46 (br s, 1H), 1.31–1.17 (m, 1H), 0.90–0.61 (m, 4H). ^1^H NMR (500 MHz, DMSO‐*d*_6_/D_2_O 9:1 v/v) δ 8.61 (d, *J* **=** 8.5 Hz, 1H), 8.18 (d, *J* **=** 8.2 Hz, 1H), 8.14 (d, *J* **=** 8.2 Hz, 1H), 8.09–8.02 (m, 2H), 7.77 (t, *J* **=** 7.8 Hz, 1H), 7.72–7.66 (m, 1H), 7.66–7.58 (m, 2.3H), 7.44–7.35 (m, 1.7H), 3.56 (t, *J* **=** 6.7 Hz, 2H), 3.23 (d, *J* **=** 6.5 Hz, 1.5H), 3.14 (br s, 0.5H), 2.83–2.74 (m, 2H), 2.59 (d, *J* **=** 6.7 Hz, 2H), 1.76–1.52 (m, 8H), 1.44 (br s, 1H), 1.25–1.13 (m, 1H), 0.83–0.62 (m, 4H). ^13^C NMR (126 MHz, DMSO‐*d*_*6*_) δ 159.86 (Cq^qz^), 158.62 (q, *J* **=** 31.5 Hz, carbonyl^TFA^), 153.11 (Cq^qz^), 139.14 (Cq^qz^), 135.85 (Cq^naph^), 135.19 (CH^qz^), 133.85 (Cq^naph^), 133.55 (CH^naph^), 128.89 (CH^naph^), 128.23 (CH^naph^), 127.73 (CH^naph^), 127.56 (Cq^naph^), 126.81 (CH^naph^), 124.76 (CH^naph^), 124.50 (CH^naph^), 124.02 (CH^qz^), 123.95 (CH^qz^), 118.22 (CF_3_), 116.63 (CH^qz^), 115.85 (CF_3_), 109.49 (Cq^qz^), 48.60 (CH_2_^cy‐exo^), 46.61 (CH_2_^cy‐exo^), 40.60 (CH_2_^linker^), 38.58 (CH_2_^linker^), 37.52 (CH^cy^), 37.45 (CH^cy^), 29.68 (CH_2_^cy^), 29.55 (CH_2_^cy^), 25.05 (CH_2_^linker^), 24.61 (CH_2_^linker^). HRMS (ESI): *m/z* [M**+**H]^+^ calcd. for [C_30_H_39_N_6_O_2_S]^+^ 547.2850, found: 547.2859. RP‐HPLC (220 nm): 97% (*t*_R_ **=** 15.0 min, *k* **=** 4.8). C_30_H_38_N_6_O_2_S** ∙** C_4_H_2_F_6_O_4_ (546.73** +** 228.04).

*N*‐[2‐({2‐[({(1*r*,4*r*)‐4‐[(Naphthalene‐1‐sulfonamido)methyl]cyclohexyl}methyl)amino]quinazolin‐4‐yl}amino)ethyl]propionamide hydrotrifluoroacetate (**32**): **32** was prepared from **28** dihydrochloride (121 mg, 204.53 µmol) and **31** (52.5 mg, 306.80 µmol) in a mixture of MeOH/DMF 4:3 v/v (700 µL) according to general procedure D (DIPEA: 104 µL, 613.59 µmol; reaction time: 1.5 h). Purification by preparative HPLC (system 1, gradient: 0–20 min: 0.1% aq. TFA/acetonitrile 80:20–30:70, *t*_R_ **=** 18 min) yielded **32** as a white fluffy solid (19.8 mg, 14%). ^1^H NMR (500 MHz, DMSO‐*d*_*6*_) (two major rotamers with a ratio of approximately 4:1 were evident in the ^1^H NMR spectrum) δ 12.68 (s, 0.8H), 11.57 (s, 0.2H), 9.53 (br s, 0.8H), 9.26 (br s, 0.2H), 8.72 (br s, 0.2H), 8.67 (d, *J* **=** 8.6 Hz, 1H), 8.45 (t, *J* **=** 5.8 Hz, 0.8H), 8.21 (d, *J* **=** 8.2 Hz, 1H), 8.17 (d, *J* **=** 8.1 Hz, 1H), 8.12–8.05 (m, 2H), 7.94 (br s, 2H), 7.78 (t, *J* **=** 7.8 Hz, 1H), 7.72–7.59 (m, 3.2H), 7.46–7.36 (m, 1.8H), 3.62 (q, *J* **=** 6.1 Hz, 2H), 3.40–3.32 (m, 2H), 3.29 (t, *J* **=** 6.3 Hz, 1.6H), 3.17 (br s, 0.4H), 2.61 (t, *J* **=** 6.0 Hz, 2H), 2.06 (q, *J* **=** 7.6 Hz, 2H), 1.78–1.56 (m, 4H), 1.43 (br s, 1H), 1.30–1.15 (m, 1H), 0.97 (t, *J* **=** 7.6 Hz, 3H), 0.89–0.76 (m, 2H), 0.76–0.62 (m, 2H). ^1^H NMR (500 MHz, DMSO‐*d*_*6*_/D_2_O 9:1 v/v) δ 8.61 (d, *J* **=** 8.6 Hz, 1H), 8.17 (d, *J* **=** 8.2 Hz, 1H), 8.14‐8.00 (m, 3H), 7.76 (t, *J* **=** 7.7 Hz, 1H), 7.72–7.56 (m, 3.3H), 7.45–7.31 (m, 1.7H), 3.60 (t, *J* **=** 6.1 Hz, 2H), 3.33 (t, *J* **=** 6.1 Hz, 2H), 3.25 (d, *J* **=** 6.6 Hz, 1.5H), 3.14 (br s, 0.5H), 2.63–2.55 (m, 2H), 2.03 (q, *J* **=** 7.6 Hz, 2H), 1.75–1.52 (m, 4H), 11.41 (br s, 1H), 1.28–1.11 (m, 1H), 0.93 (t, *J* **=** 7.6 Hz, 3H), 0.84‐0.57 (m, 4H). ^13^C NMR (126 MHz, DMSO‐*d*_*6*_) δ 173.32 (carbonyl^prop^), 160.10 (Cq^qz^), 158.75 (q, *J* **=** 33.1 Hz, carbonyl^TFA^), 153.02 (Cq^qz^), 139.08 (Cq^qz^), 135.86 (Cq^naph^), 135.13 (CH^qz^), 133.84 (Cq^naph^), 133.54 (CH^naph^), 128.88 (CH^naph^), 128.27 (CH^naph^), 127.72 (CH^naph^), 127.57 (Cq^naph^), 126.79 (CH^naph^), 124.78 (CH^naph^), 124.50 (CH^naph^), 124.05 (CH^qz^), 124.01 (CH^qz^), 117.86 (TFA, CF_3_), 116.62 (CH^qz^), 115.49 (TFA, CF_3_), 109.61 (Cq^qz^), 48.61 (CH_2_^cy‐exo^), 46.47 (CH_2_^cy‐exo^), 41.20 (CH_2_^linker^), 37.61 (CH^cy^), 37.40 (CH^cy^), 37.20 (CH_2_^linker^), 29.55 (2 CH_2_^cy^), 28.50 (CH_2_^prop^), 9.83 (CH_3_^prop^). HRMS (ESI): *m/z* [M**+**H]^+^ calcd. for [C_31_H_39_N_6_O_3_S]^+^ 575.2799, found: 575.2808. RP‐HPLC (220 nm): >99% (*t*_R_ **=** 17.2 min, *k* **=** 5.6). C_31_H_38_N_6_O_3_S** ∙** C_2_HF_3_O_2_ (574.74** +** 114.02).

*N*‐[3‐({2‐[({(1*r*,4*r*)‐4‐[(Naphthalene‐1‐sulfonamido)methyl]cyclohexyl}methyl)amino]quinazolin‐4‐yl}amino)propyl]propionamide hydrotrifluoroacetate (**33**): **33** was prepared from **29** hydrochloride (50.0 mg, 87.85 µmol) and **31** (23.0 mg, 131.77 µmol) in MeOH (200 µL) according to general procedure D (DIPEA: 30 µL, 175.70 µmol). After 3 h, an additional portion of **31** (15 mg, 87.85 µmol) was added, and stirring was continued for 1.5 d. The reaction was stopped by the addition of 10% aq. TFA (300 µL). Purification by preparative HPLC (system 1, gradient: 0–30 min: 0.1% aq. TFA/acetonitrile 80:20–30:70, *t*_R_ **=** 19 min) yielded **33** as a white fluffy solid (15 mg, 29%). ^1^H NMR (600 MHz, DMSO‐*d*_*6*_) (two major rotamers with a ratio of approximately 2.3:1 were evident in the ^1^H and ^13^C NMR spectra) δ 12.30 (s, 0.7H), 11.46 (s, 0.2H), 9.47 (br s, 0.7H), 9.19 (s, 0.2H), 8.71 (br s, 0.2H), 8.66 (d, *J* **=** 8.6 Hz, 1H), 8.26–8.13 (m, 2.8H), 8.08 (t, *J* **=** 8.6 Hz, 2H), 7.93 (t, *J* **=** 5.8 Hz, 1H), 7.85–7.75 (m, 2H), 7.73–7.68 (m, 1H), 7.68–7.59 (m, 2.2H), 7.48–7.36 (m, 1.8H), 3.56 (q, *J* **=** 6.6 Hz, 2H, interfering with the water signal), 3.25 (t, *J* **=** 6.2 Hz, 1.4H), 3.19–3.09 (m, 2.6H), 2.66–2.57 (m, 2H), 2.11–2.00 (m, 2H), 1.77 (p, *J* **=** 7.0 Hz, 2H), 1.73–1.58 (m, 4H), 1.46 (br s, 1H), 1.23 (br s, 1H), 0.97 (t, *J* **=** 7.6 Hz, 3H), 0.86–0.76 (m, 2H), 0.76–0.64 (m, 2H). ^1^H NMR (600 MHz, DMSO‐*d*_*6*_/D_2_O 9:1 v/v) δ 8.61 (d, *J* **=** 8.6 Hz, 1H), 8.18 (d, *J* **=** 8.2 Hz, 1H), 8.12 (d, *J* **=** 8.4 Hz, 1H), 8.09–8.01 (m, 2H), 7.76 (t, *J* **=** 7.8 Hz, 1H), 7.71–7.66 (m, 1H), 7.65–7.59 (m, 2.3H), 7.44–7.35 (m, 1.7H), 3.53 (t, *J* **=** 7.0 Hz, 2H), 3.23 (d, *J* **=** 6.2 Hz, 1.5H), 3.13 (br s, 0.5H), 3.10 (t, *J* **=** 7.0 Hz, 2H), 2.63–2.55 (m, 2H), 2.10–1.98 (m, 2H), 1.75 (p, *J* **=** 7.0 Hz, 2H), 1.69–1.54 (m, 4H), 1.43 (br s, 1H), 1.19 (br s, 1H), 0.94 (t, *J* **=** 7.6 Hz, 3H), 0.82–0.72 (m, 2H), 0.71–0.60 (m, 2H). ^13^C NMR (151 MHz, DMSO‐*d*_*6*_) δ 172.97 (carbonyl^prop^), 159.72 (Cq^qz^), 158.31 (q, *J* **=** 33.0 Hz, carbonyl^TFA^), 152.91 (Cq^qz^), 138.99 (Cq^qz^), 135.85 (Cq^naph^), 135.10 (CH^qz^), 133.84 (Cq^naph^), 133.54 (CH^naph^), 128.88 (CH^naph^), 128.26 (CH^naph^), 127.72 (CH^naph^), 127.56 (Cq^naph^), 126.80 (CH^naph^), 124.76 (CH^naph^), 124.50 (CH^naph^), 124.09 (CH^qz^), 123.88 (CH^qz^), 117.29 (CH^qz^), 116.77 (CH^qz^), 116.59 (q, *J* **=** 296.9 Hz, CF_3_), 109.57 (Cq^qz^), 48.60 (CH_2_^cy‐exo^), 46.96 (CH_2_^cy‐exo^), 46.59 (CH_2_^cy‐exo^), 37.53 (CH^cy^), 37.41 (CH^cy^), 36.60 (CH_2_^linker^), 36.35 (CH_2_^linker^), 29.63 (CH_2_^cy^), 29.53 (CH_2_^cy^), 28.51 (CH_2_^prop^), 28.36 (CH_2_^prop^), 9.98 (CH_3_^prop^). Note: one CH_2_^linker^ signal interferes with the DMSO residual peak. HRMS (ESI): *m/z* [M**+**H]^+^ calcd. for [C_32_H_41_N_6_O_3_S]^+^ 589.2955, found: 589.2964. RP‐HPLC (220 nm): >99% (*t*_R_ **=** 17.5 min, *k* **=** 5.7). C_32_H_40_N_6_O_3_S** ∙** C_2_HF_3_O_2_ (588.77** +** 114.02).

*N*‐[4‐({2‐[({(1*r*,4*r*)‐4‐[(Naphthalene‐1‐sulfonamido)methyl]cyclohexyl}methyl)amino]quinazolin‐4‐yl}amino)butyl]propionamide hydrotrifluoroacetate (**34**): **34** was prepared from **30** dihydrochloride (50.0 mg, 80.69 µmol) and **31** (20.7 mg, 121.04 µmol) in DMF (300 mL) according to general procedure D (DIPEA: 41 µL, 242.07 µmol; reaction time: overnight). Purification by preparative HPLC (system 1, gradient: 0–30 min: 0.1% aq. TFA/acetonitrile 80:20–30:70, *t*_R_ **=** 19 min) yielded **34** as a white fluffy solid (9 mg, 16%). ^1^H NMR (600 MHz, DMSO‐*d*_*6*_) (two major rotamers with a ratio of approximately 4:1 were evident in the ^1^H and ^13^C NMR spectra) δ 12.56 (s, 0.8H), 11.51 (s, 0.2H), 9.50 (br s, 0.8H), 9.23 (s, 0.2H), 8.69 (s, 0.2H), 8.66 (d, *J* **=** 8.6 Hz, 1H), 8.38 (br s, 0.8H), 8.27–8.16 (m, 2H), 8.14–8.03 (m, 2H), 7.93 (t, *J* **=** 5.9 Hz, 1H), 7.82–7.72 (m, 2H), 7.70 (t, *J* **=** 7.6 Hz, 1.1H), 7.67–7.60 (m, 2.1H), 7.46–7.34 (m, 1.8H), 3.63–3.49 (m, 2H), 3.25 (t, *J* **=** 6.2 Hz, 1.5H), 3.16 (br s, 0.5H), 3.11–3.03 (m, 2H), 2.61 (t, *J* **=** 6.3 Hz, 2H), 2.03 (q, *J* **=** 7.6 Hz, 2H), 1.75–1.56 (m, 6H), 1.53–1.39 (m, 3H), 1.22 (br s, 1H), 0.96 (t, *J* **=** 7.6 Hz, 3H), 0.85–0.75 (m, 2H), 0.75–0.63 (m, 2H). ^1^H NMR (600 MHz, DMSO‐*d*_*6*_/D_2_O 9:1 v/v) δ 8.61 (d, *J* **=** 8.6 Hz, 1H), 8.17 (d, *J* **=** 8.2 Hz, 1H), 8.13 (d, *J* **=** 8.2 Hz, 1H), 8.07 (d, *J* **=** 7.1 Hz, 1H), 8.03 (d, *J* **=** 8.1 Hz, 1H), 7.76 (t, *J* **=** 7.7 Hz, 1H), 7.71–7.66 (m, 1H), 7.65–7.58 (m, 2.3H), 7.43–7.34 (m, 1.7H), 3.54 (t, *J* **=** 7.0 Hz, 2H), 3.22 (d, *J* **=** 6.6 Hz, 1.5H), 3.13 (br s, 0.5H), 3.08–3.00 (m, 2H), 2.59 (d, *J* **=** 6.8 Hz, 2H), 2.02 (q, *J* **=** 7.6 Hz, 2H), 1.76–1.53 (m, 6H), 1.51–1.34 (m, 3H), 1.19 (br s, 1H), 0.94 (t, *J* **=** 7.6 Hz, 3H), 0.83–0.56 (m, 4H). ^13^C NMR (151 MHz, DMSO‐*d*_*6*_) δ 172.73 (carbonyl^prop^), 159.72 (Cq^qz^), 159.06 (Cq^qz^), 158.61 (q, *J* **=** 33.3 Hz, carbonyl^TFA^), 153.21 (Cq^qz^), 152.99 (Cq^qz^), 139.07 (Cq^qz^), 138.79 (Cq^qz^), 135.85 (Cq^naph^), 135.09 (CH^qz^), 134.71 (CH^qz^), 133.84 (Cq^naph^), 133.54 (CH^naph^), 128.87 (CH^naph^), 128.26 (CH^naph^), 127.71 (CH^naph^), 127.57 (Cq^naph^), 126.79 (CH^naph^), 124.77 (CH^naph^), 124.49 (CH^naph^), 124.02 (CH^qz^), 123.94 (CH^qz^), 123.73 (CH^qz^), 117.29 (CH^qz^), 116.69 (CH^qz^), 116.55 (q, *J* **=** 295.8 Hz, CF_3_), 110.22 (Cq^qz^), 109.54 (Cq^qz^), 48.59 (CH_2_^cy‐exo^), 46.96 (CH_2_^cy‐exo^), 46.60 (CH_2_^cy‐exo^), 41.00 (CH_2_^linker^), 40.80 (CH_2_^linker^), 38.04 (CH_2_^linker^), 37.52 (CH^cy^), 37.41 (CH^cy^), 36.60 (CH^cy^), 29.66 (CH_2_^cy^), 29.53 (CH_2_^cy^), 28.52 (CH_2_^prop^), 26.81 (CH_2_^linker^), 25.60 (CH_2_^linker^), 10.01 (CH_3_^prop^). HRMS (ESI): *m/z* [M**+**H]^+^ calcd. for [C_33_H_43_N_6_O_3_S]^+^ 603.3112, found: 603.3120. RP‐HPLC (220 nm): >99% (*t*_R_ **=** 17.7 min, *k* **=** 5.8). C_33_H_42_N_6_O_3_S** ∙** C_2_HF_3_O_2_ (602.80** +** 114.02).

*tert*‐Butyl [1‐({2‐[({(1*r*,4*r*)‐4‐[(naphthalene‐1‐sulfonamido)methyl]cyclohexyl}methyl)amino]quinazolin‐4‐yl}amino)‐4‐oxo‐6,9,12‐trioxa‐3‐azatetradecan‐14‐yl]carbamate (**36**): **28** dihydrochloride (58 mg, 98.04 µmol) and DIPEA (78 µL, 447.79 µmol) were dissolved in DMF (200 µL) and the mixture was stirred at rt for 20 min. HATU (43 mg, 113.09 µmol) and 2,2‐dimethyl‐4‐oxo‐3,8,11,14‐tetraoxa‐5‐azahexadecan‐16‐oic acid (34 mg, 113.09 µmol) were added and stirring was continued at rt overnight. The solvents were removed under vacuum. The product was obtained after purification via flash chromatography (gradient: 0.0–3.0 CV: CH_2_Cl_2_/MeOH 100:0 v/v (isocratic), 3.0–4.0 CV: 100:0–98:2 v/v, 4.0–8.6 CV: 98:2–92.5:7.5 v/v, 8.6–10.0 CV: 92.5:7.5–90:10 v/v, 10.0–13.0 CV: 90:10 v/v (isocratic)) as a colorless resin (24 mg, 30%). MS (ESI): *m/z* (%) 808.3 [M**+**H]^+^. C_41_H_57_N_7_O_8_S (808.01).

2‐{2‐[2‐(2‐Aminoethoxy)ethoxy]ethoxy}‐*N*‐[2‐({2‐[({(1*r*,4*r*)‐4‐[(naphthalene‐1‐sulfonamido)methyl]cyclohexyl}methyl)amino]quinazolin‐4‐yl}amino)ethyl]acetamide bis(hydrotrifluoroacetate) (**37**): **36** (39.4 mg, 48.76 µmol) was dissolved in 2 N HCl in MeOH (480 µL) according to general procedure C. Evaporation of the volatiles gave **37** as a colorless oil (41.5 mg, 109%). Half of the residue was used without further purification, the other half was subjected to preparative HPLC (system 2, gradient: 0–23 min: 0.1% aq. TFA/acetonitrile 75:25–55:45, *t*_R_ **=** 12 min) yielding **37** as a white fluffy solid (13.2 mg, 53%). ^1^H NMR (600 MHz, DMSO‐*d*_*6*_) (two major rotamers with a ratio of approximately 4:1 were evident in the ^1^H and ^13^C NMR spectra) δ 13.01 (s, 0.8H), 11.73 (0.2H), 9.54 (t, *J* **=** 5.7 Hz, 0.8H), 9.29 (br s, 0.2H), 8.88–8.55 (m, 2H), 8.19 (dd, *J* **=** 14.6, 8.2 Hz, 2H), 8.08 (dd, *J* **=** 12.2, 7.7 Hz, 2H), 7.96 (dt, *J* **=** 19.2, 5.9 Hz, 2H), 7.85 (s, 3H), 7.78 (t, *J* **=** 7.8 Hz, 1H), 7.73–7.68 (m, 1.2H), 7.68–7.60 (m, 2H), 7.47–7.31 (m, 1.8H), 3.88 (s, 2H), 3.69–3.62 (m, 2H), 3.61–3.35 (m, 12H, interfering with the water signal), 3.30 (t, *J* **=** 6.2 Hz, 1.5H), 3.18 (br s, 0.5H), 3.00–2.91 (m, 2H), 2.61 (t, *J* **=** 6.3 Hz, 2H), 1.75–1.58 (m, 4H), 1.43 (br s, 1H), 1.22 (br s, 1H), 0.88–0.76 (m, 2H), 0.76–0.65 (m, 2H). ^1^H NMR (600 MHz, DMSO‐*d*_*6*_/D_2_O 9:1 v/v) δ 8.61 (d, *J* **=** 8.6 Hz, 1H), 8.17 (d, *J* **=** 8.2 Hz, 1H), 8.10 (d, *J* **=** 8.1 Hz, 1H), 8.05 (dd, *J* **=** 15.3, 7.7 Hz, 2H), 7.77 (t, *J* **=** 7.8 Hz, 1H), 7.68 (t, *J* **=** 7.6 Hz, 1H), 7.66–7.58 (m, 2.3H), 7.44‐7.35 (m, 1.7H), 3.85 (s, 2H), 3.66–3.60 (m, 2H), 3.55 (t, *J* **=** 5.2 Hz, 2H), 3.52–3.46 (m, 8H), 3.42 (t, *J* **=** 6.1 Hz, 2H), 3.26 (d, *J* **=** 6.6 Hz, 1.5H), 3.15 (br s, 0.5H), 2.93 (t, *J* **=** 5.1 Hz, 2H), 2.59 (d, *J* **=** 6.8 Hz, 2H), 1.72–1.53 (m, 4H), 1.41 (br s, 1H), 1.19 (br s, 1H), 0.82–0.73 (m, 2H), 0.72–0.61 (m, 2H). ^13^C NMR (151 MHz, DMSO‐*d*_*6*_) δ 169.88 (carbonyl^PEG‐linker^), 169.77 (carbonyl^PEG‐linker^), 160.17 (Cq^qz^), 159.46 (Cq^qz^), 158.73 (q, *J* **=** 31.7 Hz, carbonyl^TFA^), 153.14 (Cq^qz^), 139.16 (Cq^qz^), 138.84 (Cq^qz^), 135.87 (Cq^naph^), 135.17 (CH^qz^), 134.75 (CH^qz^), 133.85 (Cq^naph^), 133.55 (CH^naph^), 128.88 (CH^naph^), 128.25 (CH^naph^), 127.73 (CH^naph^), 127.57 (Cq^naph^), 126.80 (CH^naph^), 124.78 (CH^naph^), 124.50 (CH^naph^), 124.08 (CH^qz^), 123.97 (CH^qz^), 123.83 (CH^qz^), 117.30 (CH^qz^), 117.05 (q, *J* **=** 298.7 Hz, CF_3_), 116.54 (CH^qz^), 110.31 (Cq^qz^), 109.58 (Cq^qz^), 70.06 (CH_2_^PEG‐linker^), 70.01 (2 × CH_2_^PEG‐linker^), 69.62 (CH_2_^PEG‐linker^), 69.55 (CH_2_^PEG‐linker^), 66.67 (CH_2_^PEG‐linker^), 48.62 (CH_2_^cy‐exo^), 46.93 (CH_2_^cy‐exo^), 46.45 (CH_2_^cy‐exo^), 41.05 (CH_2_^linker^), 38.56 (CH_2_^PEG‐linker^), 37.62 (CH^cy^), 37.42 (CH^cy^), 37.11 (CH_2_^linker^), 36.97 (CH_2_^linker^), 36.64 (CH_2_^linker^), 29.56 (CH_2_^cy^), 29.44 (CH_2_^cy^). HRMS (ESI): *m/z* [M**+**2H]^2+^ calcd. for [C_36_H_51_N_7_O_6_S]^2+^ 354.6806, found: 354.6812. RP‐HPLC (220 nm): 97% (*t*_R_ **=** 14.9 min, *k* **=** 4.7). C_36_H_49_N_7_O_6_S** ∙** C_4_H_2_F_6_O_4_ (707.89** +** 228.04).

*N*‐[1‐({2‐[({(1*r*,4*r*)‐4‐[(Naphthalene‐1‐sulfonamido)methyl]cyclohexyl}methyl)amino]quinazolin‐4‐yl}amino)‐4‐oxo‐6,9,12‐trioxa‐3‐azatetradecan‐14‐yl]propionamide hydrotrifluoroacetate (**38**): **38** was prepared from **37** dihydrochloride (20.8 mg, 26.58 µmol) and **31** (7.5 mg, 43.82 µmol) in DMF (300 µL) according to general procedure D applying a reaction time of 2 h (DIPEA: 19 µL, 106.30 µmol). Purification by preparative HPLC (system 2, gradient: 0–23 min: 0.1% aq. TFA/acetonitrile 70:30–50:50, *t*_R_ **=** 14 min) yielded **38** as a white fluffy solid (12 mg, 46%). ^1^H NMR (600 MHz, DMSO‐*d*_*6*_) δ 12.70–12.40 (m, 0.8H), 11.54 (s, 0.2H), 9.51 (br s, 0.8H), 9.25 (s, 0.2H), 8.73 (s, 0.2H), 8.66 (d, *J* **=** 8.6 Hz, 1H), 8.43–8.29 (m, 0.8H), 8.20 (d, *J* **=** 8.2 Hz, 1H), 8.16 (d, *J* **=** 8.2 Hz, 1H), 8.12–8.04 (m, 2H), 7.98–7.88 (m, 2H), 7.82–7.74 (m, 2H), 7.74‐7.57 (m, 3.2H), 7.48–7.33 (m, 1.8H), 3.87 (s, 2H), 3.70–3.60 (m, 2H), 3.57–3.52 (m, 2H), 3.52–3.48 (m, 2H), 3.46–3.42 (m, 6H), 3.40–3.32 (m, 2H, interfering with the water signal), 3.29 (t, *J* **=** 6.2 Hz, 1.5H, interfering with the water signal), 3.16 (q, *J* **=** 5.9 Hz, 2.5H), 2.65–2.58 (m, 2H), 2.05 (q, *J* **=** 7.6 Hz, 2H), 1.75–1.56 (m, 4H), 1.44 (br s, 1H), 1.27–1.15 (m, 1H), 0.96 (t, *J* **=** 7.6 Hz, 3H), 0.86–0.75 (m, 2H), 0.75–0.65 (m, 2H). ^1^H NMR (600 MHz, DMSO‐*d*_*6*_/D_2_O 9:1 v/v) δ 8.61 (d, *J* **=** 8.6 Hz, 1H), 8.17 (d, *J* **=** 8.2 Hz, 1H), 8.12–8.01 (m, 3H), 7.76 (t, *J* **=** 7.8 Hz, 1H), 7.71–7.66 (m, 1H), 7.66–7.58 (m, 2.3H), 7.44–7.34 (m, 1.7H), 3.84 (s, 2H), 3.67–3.60 (m, 2H), 3.55–3.48 (m, 2H), 3.48–3.44 (m, 2H), 3.44–3.38 (m, 6H), 3.34 (t, *J* **=** 5.9 Hz, 2H), 3.26 (d, *J* **=** 6.6 Hz, 1.5H), 3.14 (t, *J* **=** 5.9 Hz, 2.5H), 2.63–2.56 (m, 2H), 2.03 (q, *J* **=** 7.6 Hz, 2H), 1.72–1.53 (m, 4H), 1.41 (br s, 1H), 1.19 (br s, 1H), 0.94 (t, *J* **=** 7.6 Hz, 3H), 0.82–0.73 (m, 2H), 0.73–0.60 (m, 2H). ^13^C NMR (151 MHz, DMSO‐*d*_*6*_) δ 172.99 (carbonyl^prop^), 169.78 (Carbonyl^PEG‐linker^), 160.14 (Cq^qz^), 159.45 (Cq^qz^), 158.65 (carbonyl^TFA^), 153.16 (Cq^qz^), 152.98 (Cq^qz^), 139.06 (Cq^qz^), 138.76 (Cq^qz^), 135.86 (Cq^naph^), 135.15 (CH^qz^), 134.78 (CH^qz^), 133.85 (Cq^naph^), 133.55 (CH^naph^), 128.88 (CH^naph^), 128.27 (CH^naph^), 127.73 (CH^naph^), 127.57 (Cq^naph^), 126.80 (CH^naph^), 124.77 (CH^naph^), 124.50 (CH^naph^), 124.04 (CH^qz^), 123.81 (CH^qz^), 117.26 (CH^qz^), 117.10 (q, *J* **=** 298.5 Hz, CF_3_), 116.66 (CH^qz^), 110.33 (Cq^qz^), 109.61 (Cq^qz^), 70.12 (CH_2_^PEG‐linker^), 70.01 (CH_2_^PEG‐linker^), 69.67 (CH_2_^PEG‐linker^), 69.53 (CH_2_^PEG‐linker^), 69.50 (CH_2_^PEG‐linker^), 69.12 (CH_2_^PEG‐linker^), 48.61 (CH_2_^cy‐exo^), 46.96 (CH_2_^cy‐exo^), 46.48 (CH_2_^cy‐exo^), 41.10 (CH_2_^linker^), 38.42 (CH_2_^PEG‐linker^), 37.61 (CH^cy^), 37.41 (CH^cy^), 37.07 (CH_2_^linker^), 36.93 (CH_2_^linker^), 36.61 (CH_2_^linker^), 29.55 (CH_2_^cy^), 28.39 (CH_2_^prop^), 9.90 (CH_3_^prop^). HRMS (ESI): *m/z* [M**+**H]^+^ calcd. for [C_39_H_54_N_7_O_7_S]^+^ 764.3800, found: 764.3814. RP‐HPLC (220 nm): 98% (*t*_R_ **=** 17.4 min, *k* **=** 5.7). C_39_H_53_N_7_O_7_S** ∙** C_2_HF_3_O_2_ (763.95** +** 114.02).

*tert*‐Butyl (2‐{[2‐({2‐[({(1*r*,4*r*)‐4‐[(naphthalene‐1‐sulfonamido)methyl]cyclohexyl}methyl)amino]quinazolin‐4‐yl}amino)ethyl]amino}ethyl)carbamate bis(hydrotrifluoroacetate) (**40**): **28** dihydrochloride (150 mg, 254.06 µmol), *tert*‐butyl (2‐bromoethyl)carbamate (39.9 mg, 177.83 µmol), and anhydrous K_2_CO_3_ (234.0 mg, 1.69 mmol) were suspended in acetonitrile (3 mL). The mixture was stirred under microwave irradiation (110°C, 15 min) in a sealed tube. The liquid was transferred to a round‐bottom flask, the solvent was removed under reduced pressure, the residue was mixed with water, and the aqueous solution was treated three times with CH_2_Cl_2_. The organic phases were combined, dried over Na_2_SO_4_, and the volatiles were removed by rotary evaporation. Purification by preparative HPLC (system 2, gradient: 0–25 min: 0.1% aq. TFA/acetonitrile 75:25–35:65, *t*_R_ **=** 13 min) yielded **40** as a white fluffy solid (22.2 mg, 10%). HRMS (ESI): *m/z* [M**+**H]^+^ calcd. for [C_35_H_48_N_7_O_4_S]^+^ 662.3483, found: 662.3485. C_35_H_47_N_7_O_4_S** ∙** C_4_H_2_F_6_O_4_ (661.87** +** 228.04).

*N*‐{[(1*r*,4*r*)‐4‐({[4‐({2‐[(2‐Aminoethyl)amino]ethyl}amino)quinazolin‐2‐yl]amino}methyl)cyclohexyl]methyl}naphthalene‐1‐sulfonamide tris(hydrotrifluoroacetate) (**41**): **40** (16.5 mg, 24.93 µmol) was dissolved in 2 N HCl in MeOH (500 µL) followed by processing according to general procedure C. The residue was subjected to preparative HPLC (system 2, gradient: 0–25 min: 0.1% aq. TFA/acetonitrile 75:25–50:50. *t*_R_ **=** 10 min) yielding **41** as a white fluffy solid (14.3 mg, 63%). ^1^H NMR (600 MHz, DMSO‐*d*_*6*_) (two major rotamers with a ratio of approximately 3:1 were evident in the ^1^H and ^13^C NMR spectra) δ 13.12 (s, 0.7H), 11.85 (s, 0.2H), 9.58 (s, 0.7H), 9.44 (s, 0.3H), 9.19 (br s, 2H), 8.85–8.68 (m, 1H), 8.66 (d, *J* **=** 8.6 Hz, 1H), 8.23–8.00 (m, 7H), 7.95 (t, *J* **=** 6.0 Hz, 1H), 7.80 (t, *J* **=** 7.7 Hz, 1H), 7.72–7.67 (m, 1.2H), 7.67–7.59 (m, 2H), 7.47–7.34 (m, 1.8H), 3.88 (br s, 1.5H), 3.82 (br s, 0.5H), 3.53–3.18 (m, 6H, interfering with the water signal), 3.14 (br s, 2H), 2.62 (t, *J* **=** 6.4 Hz, 2H), 1.77–1.58 (m, 4H), 1.53–1.38 (m, 1H), 1.29–1.19 (m, 1H), 0.89–0.78 (m, 2H), 0.78–0.68 (m, 2H). ^1^H NMR (600 MHz, DMSO‐*d*_*6*_/D_2_O 9:1 v/v) δ 8.57 (d, *J* **=** 8.6 Hz, 1H), 8.20–8.12 (m, 1H), 8.09–7.98 (m, 3H), 7.78 (t, *J* **=** 7.8 Hz, 1H), 7.71–7.65 (m, 1H), 7.65–7.57 (m, 2.4H), 7.46–7.33 (m, 1.6H), 3.84 (br s, 1.3H), 3.80 (br s, 0.7H), 3.38–3.00 (m, 8H), 2.58 (d, *J* **=** 6.8 Hz, 2H), 1.72–1.50 (m, 4H), 1.40 (br s, 1H), 1.19 (br s, 1H), 0.83–0.73 (m, 2H), 0.73–0.61 (m, 2H). ^13^C NMR (151 MHz, DMSO‐*d*_*6*_) δ 160.55 (Cq^qz^), 160.11 (Cq^qz^), 159.19 (carbonyl^TFA^), 158.97 (carbonyl^TFA^), 153.16 (Cq^qz^), 139.22 (Cq^qz^), 135.85 (Cq^naph^), 135.42 (CH^qz^), 134.99 (CH^qz^), 133.85 (Cq^naph^), 133.55 (CH^naph^), 128.89 (CH^naph^), 128.22 (CH^naph^), 127.73 (CH^naph^), 127.57 (Cq^naph^), 126.81 (CH^naph^), 124.76 (CH^naph^), 124.50 (CH^naph^), 124.27 (CH^qz^), 123.95 (CH^qz^), 117.33 (CH^qz^), 116.99 (q, *J* **=** 298.5 Hz, CF_3_), 116.54 (CH^qz^), 110.31 (Cq^qz^), 109.57 (Cq^qz^), 48.60 (CH_2_^cy‐exo^), 46.93 (CH_2_^cy‐exo^), 46.55 (CH_2_^cy‐exo^), 46.25 (CH_2_^cy‐exo^), 45.41 (CH_2_^linker^), 44.05 (CH_2_^linker^), 37.92 (CH_2_^linker^), 37.54 (CH^cy^), 37.45 (CH^cy^), 36.68 (CH^cy^), 35.18 (CH_2_^linker^), 29.55 (CH_2_^cy^), 29.40 (CH_2_^cy^). HRMS (ESI): *m/z* [M**+**H]^+^ calcd. for [C_30_H_40_N_7_O_2_S]^+^ 562.2959, found: 562.2961. RP‐HPLC (220 nm): 97% (*t*_R_ **=** 13.5 min, *k* **=** 4.2). C_30_H_39_N_7_O_2_S** ∙** C_6_H_3_F_9_O_6_ (561.75** +** 342.07).

*N*‐(2‐{[2‐({2‐[({(1*r*,4*r*)‐4‐[(Naphthalene‐1‐sulfonamido)methyl]cyclohexyl}methyl)amino]quinazolin‐4‐yl}amino)ethyl]amino}ethyl)propionamide bis(hydrotrifluoroacetate) (**42**): **42** was prepared from **41** (6.0 mg, 6.64 µmol) and **31** (1.7 mg, 9.96 µmol) in DMF (100 µL) according to general procedure D applying a reaction time of 1.5 h (DIPEA: 6.9 µL, 39.83 µmol). Purification by preparative HPLC (system 2, gradient: 0–20 min: 0.1% aq. TFA/acetonitrile 75:25–40:60, *t*_R_ **=** 11 min) yielded **42** as a white fluffy solid (3.4 mg, 61%). ^1^H NMR (600 MHz, DMSO‐*d*_*6*_) (two major rotamers with a ratio of approximately 2.3:1 were evident in the ^1^H and ^13^C NMR spectra) δ 12.87 (br s, 0.7H), 11.75 (br s, 0.3H), 9.52 (br s, 0.7H), 9.39 (br s, 0.3H), 8.82–8.48 (m, 4H), 8.20 (d, *J* **=** 8.2 Hz, 1H), 8.14 (d, *J* **=** 8.2 Hz, 1H), 8.07 (t, *J* **=** 7.4 Hz, 3H), 7.94 (t, *J* **=** 6.0 Hz, 1H), 7.85–7.77 (m, 1H), 7.72–7.67 (m, 1.3H), 7.67–7.59 (m, 2H), 7.49–7.37 (m, 1.7H), 3.86 (br s, 1.4H), 3.81 (br s, 0.6H), 3.33–3.24 (m, 5.2H, interference with the water signal), 3.20 (br s, 0.8H), 3.04 (br s, 2H), 2.61 (t, *J* **=** 6.4 Hz, 2H), 2.14‐2.03 (m, 2H), 1.76–1.56 (m, 4H), 1.43 (br s, 1H), 1.24 (br s, 1H), 0.99–0.91 (t, *J* **=** 7.4 Hz, 3H), 0.86–0.77 (m, 2H), 0.75–0.65 (m, 2H). ^1^H NMR (600 MHz, DMSO‐*d*_*6*_/D_2_O 9:1 v/v) δ 8.61–8.56 (m, 1H), 8.17 (d, *J* **=** 8.2 Hz, 1H), 8.08–8.01 (m, 3H), 7.79 (t, *J* **=** 7.6 Hz, 1H), 7.71–7.66 (m, 1H), 7.65–7.57 (m, 2.3H), 7.48–7.35 (m, 1.5H), 3.38–3.20 (m, 5.2H), 3.16 (br s, 0.8H), 3.09–2.95 (m, 2H), 2.58 (d, *J* **=** 6.7 Hz, 2H), 2.13–2.01 (m, 2H), 1.74‐1.52 (m, 4H), 1.40 (br s, 1H), 1.20 (br s, 1H), 0.94‐0.85 (m, 3H), 0.82–0.73 (m, 2H), 0.73–0.58 (m, 2H). Note: One CH_2_ signal is missing due to interference with the HDO peak. ^13^C NMR (151 MHz, DMSO‐*d*_*6*_) δ 174.41 (carbonyl^prop^), 174.30 (carbonyl^prop^), 160.66 (Cq^qz^), 160.22 (Cq^qz^), 158.65 (carbonyl^TFA^), 153.24 (Cq^qz^), 153.10 (Cq^qz^), 139.17 (Cq^qz^), 138.90 (Cq^qz^), 135.88 (Cq^naph^), 135.50 (CH^qz^), 135.15 (CH^qz^), 133.92 (Cq^naph^), 133.64 (CH^naph^), 128.97 (CH^naph^), 128.31 (CH^naph^), 127.82 (CH^naph^), 127.62 (Cq^naph^), 126.90 (CH^naph^), 124.80 (CH^naph^), 124.58 (CH^naph^), 124.32 (CH^qz^), 124.11 (CH^qz^), 117.33 (CH^qz^), 116.83 (q, *J* **=** 297.1 Hz, CF_3_), 116.68 (CH^qz^), 110.41 (Cq^qz^), 109.71 (Cq^qz^), 48.66 (CH_2_^cy‐exo^), 47.02 (CH_2_^linker^), 46.74 (CH_2_^linker^), 46.61 (CH_2_^cy‐exo^), 46.17 (CH_2_^cy‐exo^), 45.30 (CH_2_^linker^), 37.69 (CH^cy^), 37.61 (CH^cy^), 37.51 (CH_2_^linker^), 36.78 (CH_2_^linker^), 35.29 (CH_2_^linker^), 29.63 (CH_2_^cy^), 29.60 (CH_2_^cy^), 29.46 (CH_2_^cyg^), 28.35 (CH_2_^prop^), 9.55 (CH_3_^prop^). HRMS (ESI): *m/z* [M**+**H]^+^ calcd. for [C_33_H_44_N_7_O_3_S]^+^ 618.3221, found: 618.3227. RP‐HPLC (220 nm): 95% (*t*_R_ **=** 15.2 min, *k* **=** 4.8). C_33_H_43_N_7_O_3_S** ∙** C_4_H_2_F_6_O_4_ (617.81** +** 228.05).

3‐{[2‐({2‐[({(1*r*,4*r*)‐4‐[(Naphthalene‐1‐sulfonamido)methyl]cyclohexyl}methyl)amino]quinazolin‐4‐yl}amino)ethyl]ammonio}propane‐1‐sulfonate (**44**): **28** dihydrochloride (181 mg, 305.95 µmol) and DIPEA (320 µL, 1.83 mmol) were dissolved in DMF (2 mL) and the mixture was stirred at rt for 20 min. Sodium 3‐bromopropane‐1‐sulfonate (52 mg, 231.08 µmol) was added to the mixture in two portions at 4 min intervals. After 2 h, the temperature was increased to 30°C and stirring was continued overnight. 10% aq. TFA (2 mL) was added, and the mixture was subjected to preparative HPLC. The separation via preparative HPLC was performed with system 2 as described under *Preparative HPLC* with the following modifications: the column was replaced by a YMC‐Actus Triart C8, S‐5 μm, 250 mm × 20 mm (YMC Europe, Dinslaken, Germany), the flow was reduced to 15 mL/min, and MeOH was used instead of acetonitrile. The following gradient was applied: 0–40 min: 0.1% aq. TFA/MeOH 55:45–25:75, *t*_R_ **=** 18 min. **44** was obtained as a white fluffy solid (35 mg, 18%). Note: The product obtained was still contaminated with an unknown side product that could not be separated from **44**. HRMS (ESI): *m/z* [M**+**H]^+^ calcd. for [C_31_H_41_N_6_O_5_S_2_]^+^ 641.2575, found: 641.2582. C_31_H_40_N_6_O_5_S_2_ (640.82).

3‐{*N*‐[2‐({2‐[({(1*r*,4*r*)‐4‐[(Naphthalene‐1‐sulfonamido)methyl]cyclohexyl}methyl)amino]quinazolin‐4‐yl}ammonio)ethyl]propionamido}propane‐1‐sulfonate (**45**): **45** was prepared from impure **44** (30 mg, 46.82 µmol) and **31** (17 mg, 99.33 µmol) in DMF (1 mL) according to general procedure D applying a reaction time of 16 h (DIPEA: 58 µL, 330.36 µmol). Purification by preparative HPLC (system 2, gradient: 0–70 min: 0.1% aq. TFA/acetonitrile 65:35–35:65, *t*_R_ **=** 6 min) yielded **45** as a white fluffy solid (27 mg, 83%). ^1^H NMR (600 MHz, DMSO‐*d*_*6*_) (four major rotamers with a ratio of approximately 2:4:1:2 were evident in the ^1^H and ^13^C NMR spectra) δ 12.23 (s, 0.2H), 12.14 (s, 0.4H), 11.42 (s, 0.1H), 11.37 (s, 0.2H), 9.69–9.50 (m, 0.6H), 9.38 (br s, 0.4H), 8.95 (br s, 0.3H), 8.65 (d, *J* **=** 8.2 Hz, 1H), 8.20 (d, *J* **=** 8.2 Hz, 1.2H), 8.16–8.01 (m, 3.3H), 7.94 (br s, 0.4H), 7.88 (br s, 0.6H), 7.82–7.73 (m, 0.4H), 7.73–7.57 (m, 3.9H), 7.46–7.34 (m, 1H), 7.33–7.24 (m, 0.6H), 3.74‐3.57 (m, 2.4H), 3.56–3.32 (m, 4H), 3.30–3.12 (m, 2H), 2.61 (br s, 0.7H), 2.57–2.44 (m, 4H, interfering with the solvent residual signal), 2.36–2.23 (m, 2H), 1.94 (br s, 0.5H), 1.85 (br s, 1.5H), 1.77–1.44 (m, 4.3H), 1.37 (br s, 0.7H), 1.22 (br s, 0.4H), 1.12 (br s, 0.7H), 0.98–0.86 (m, 3H), 0.86–0.46 (m, 4H). ^13^C NMR (151 MHz, DMSO‐*d*_*6*_) δ 173.19 (carbonyl^prop^), 173.05 (carbonyl^prop^), 172.56 (carbonyl^prop^), 172.42 (carbonyl^prop^), 160.08 (Cq^qz^), 160.01 (Cq^qz^), 159.31 (Cq^qz^), 158.51 (carbonyl^TFA^), 158.27 (carbonyl^TFA^), 153.06 (Cq^qz^), 152.85 (Cq^qz^), 152.80 (Cq^qz^), 138.94 (Cq^qz^), 138.79 (Cq^qz^), 135.87 (Cq^naph^), 135.29 (CH^qz^), 135.10 (CH^qz^), 134.86 (CH^qz^), 134.73 (CH^qz^), 133.83 (Cq^naph^), 133.52 (CH^naph^), 128.85 (CH^naph^), 128.25 (CH^naph^), 127.72 (CH^naph^), 127.55 (Cq^naph^), 126.79 (CH^naph^), 124.81 (CH^naph^), 124.49 (CH^naph^), 124.18 (CH^qz^), 124.03 (CH^qz^), 123.91 (CH^qz^), 123.75 (CH^qz^), 123.66 (CH^qz^), 117.24 (CH^qz^), 116.61 (CH^qz^), 116.54 (CH^qz^), 110.15 (Cq^qz^), 110.02 (Cq^qz^), 109.42 (Cq^qz^), 109.27 (Cq^qz^), 48.87 (CH_2_^cy‐exo^), 48.62 (CH_2_^cy‐exo^), 48.48 (CH_2_^cy‐exo^), 48.42 (CH_2_^cy‐exo^), 48.19 (CH_2_^cy‐exo^), 47.33 (CH_2_^cy‐exo^), 46.94 (CH_2_^cy‐exo^), 46.75 (CH_2_^cy‐exo^), 46.67 (CH_2_^cy‐exo^), 45.43 (CH_2_^linker^), 45.04 (CH_2_^linker^), 44.87 (CH_2_^linker^), 44.16 (CH_2_^linker^), 43.88 (CH_2_^linker^), 43.52 (CH_2_^linker^), 37.41 (CH^cy^), 37.27 (CH^cy^), 37.24 (CH^cy^), 37.17 (CH^cy^), 37.14 (CH^cy^), 36.40 (CH^cy^), 29.61 (CH_2_^linker^), 29.55 (CH_2_^linker^), 29.49 (CH_2_^linker^), 29.44 (CH_2_^linker^), 29.38 (CH_2_^linker^), 25.44 (CH_2_^prop^), 25.28 (CH_2_^prop^), 24.99 (CH_2_^linker^), 24.86 (CH_2_^linker^), 23.74, 9.53 (CH_3_^propionyl^). Note: the carbon signal of one CH_2_^linker^‐group was not apparent due to interference with the solvent residual peak. HRMS (ESI): *m/z* [M–H]^–^ calcd. for [C_34_H_43_N_6_O_6_S_2_]^–^ 695.2691, found: 695.2702. RP‐HPLC (220 nm): >97% (*t*_R_ **=** 16.4 min, *k* **=** 5.3). C_34_H_44_N_6_O_6_S_2_ (696.88).

##### 4.1.2.3 Syntheses of [^3^H]**26**, [^3^H]**32**, and [^3^H]**42**

The radioligands [^3^H]**26**, [^3^H]**32**, and [^3^H]**42** were prepared following a reported procedure for the syntheses of [^3^H]propionylated neurotensin receptor ligands, which was modified as required [46]. For reaction control, purification and purity control runs of the radioligands, an HPLC system from Waters (Eschborn, Germany) consisting of two pumps 510, a pump control module, a manual injector (loop size: 200 µL), a 486 UV/vis detector, and an LB 514 Flowstar radiodetector (Berthold Technologies, Bad Wildbad, Germany) was used (the latter was disconnected during the purification process, that is, fractions containing the respective radioligand were collected based on UV detection). A Luna C18(2) column (3 μm, 150 mm × 4.6 mm; Phenomenex, Aschaffenburg, Germany) served as the stationary phase at a flow rate of 0.8 mL/min. The labeling reagents [^3^H]**2** (molar activity: 3.0 TBq/mmol, activity concentration: 10 mCi/mL; solvent: acetonitrile) and [^3^H]**31** (molar activity: 3.7 TBq/mmol; activity concentration: 3.1 mCi/mL ([^3^H]**32**), 2 mCi/mL ([^3^H]**42**); solvent: n‐heptane/EtOAc 3:2 v/v) were purchased from Novandi (Södertälje, Sweden). [^3^H]**2** was used for the synthesis of [^3^H]**26,** and [^3^H]**31** was used for the syntheses of [^3^H]**32** and [^3^H]**42**. First, the respective radioactive labeling reagent was transferred from the delivered ampule ([^3^H]**26**: 55.5 MBq, 0.15 mL, 18.5 nmol; [^3^H]**32**: 258 MBq, 2.25 mL, 70.0 nmol; [^3^H]**42**: 48.1 MBq, 0.65 mL, 13.0 nmol) into a 2 mL polypropylene reaction vessel with screw cap followed by removal of the solvents in a vacuum concentrator (rt, 15–75 min). Due to the high volume of [^3^H]**31** solution used for [^3^H]**32**, the liquid was initially split into two reaction vessels, and after 15 min of evaporation, the aliquots were recombined in one of the vessels. A suspension of the precursor **28** (0.08 mg, 107 nmol) and K_2_CO_3_ (13.0 mg, 94.1 µmol) in acetonitrile (30 µL), a solution of **28** (0.28 mg, 375 nmol) and DIPEA (1 μL, 5.74 µmol) in dry DMF (49 µL) or of **41** (0.08 mg, 90.7 nmol) and DIPEA (0.08 µL, 483 nmol) in dry DMF (30 µL) was added, immediately followed by vortexing. Subsequently, the vessels were shaken at rt overnight ([^3^H]**26**), for 2 h ([^3^H]**32**), or for 1.5 h ([^3^H]**42**). The mixtures were acidified by the addition of 2% aq. TFA (470 µL ([^3^H]**26**), 40 µL ([^3^H]**32**), or 22 µL ([^3^H]**42**)), followed by the addition of neat acetonitrile (30 µL, [^3^H]**26**) or a mixture of acetonitrile/0.05% aq. TFA 1:9 v/v (240 µL, [^3^H]**32**), 15:85 v/v (180 μL, [^3^H]**42**). For the purification by analytical HPLC, these solutions were directly injected into the HPLC system without filtration. [^3^H]**26**, [^3^H]**32**, and [^3^H]**42** were isolated using the aforementioned HPLC system. For [^3^H]**26**, mixtures of acetonitrile supplemented with 0.15% TFA (A) and 0.2% aq. TFA (B) was used as the mobile phase. The following linear gradient was applied: 0–23 min: A/B 15:85–34.5:65.5, 23–40 min: 34.5:65.5–37.2:62.8, 40–42 min: 37.2:62.8–95:5, 42–47 min: 95:5 (isocratic). For [^3^H]**32** and [^3^H]**42**, mixtures of acetonitrile supplemented with 0.035% TFA (A) and 0.05% aq. TFA (B) was used as the mobile phase. The following linear gradients were applied: [^3^H]**32**, 0–20 min: A/B 15:85–60:40, 20–22 min: 60:40–95:5, 22–30 min: 95:5 (isocratic); [^3^H]**42**, 0–20 min: A/B 20:80–48:52, 20–22 min: 48:52–95:5, 22–30 min: 95:5 (isocratic). Four ([^3^H]**26**), three ([^3^H]**32**), or two ([^3^H]**42**) runs were conducted. The fractions containing [^3^H]**26** (*t*_R_ = 40 min), [^3^H]**32** (*t*_R_ = 20 min), or [^3^H]**42** (*t*_R_ = 17 min) were collected and combined in a 2 mL polypropylene reaction vessel with a screw cap. The volume of the combined eluates was reduced by evaporation in a vacuum concentrator to approximately 170 µL ([^3^H]**26**), 220 µL ([^3^H]**32**), or 60 µL ([^3^H]**42**), followed by the addition of EtOH ([^3^H]**26**: 397 μL, [^3^H]**32**: 330 μL, [^3^H]**42**: 440 μL). In the case of [^3^H]**32**, the resulting solution was split equally into two 2‐mL polypropylene reaction vessels with screw caps, followed by the addition of EtOH/water 3:2 v/v (525 µL) to each vessel to obtain the preliminary stock solutions (2 × 800 µL). The solutions of [^3^H]**26** and [^3^H]**42** were not further diluted, that is, they represented the preliminary stock solutions. To determine the radiochemical purity and to prove the identity of the radioligands, the aforementioned HPLC system, column, and solvents were used. The injection volume was 100 µL throughout. For the analysis of [^3^H]**26**, 4 µL of the preliminary stock solution were added to 100 µL of a 20 µM solution of **26** in acetonitrile supplemented with 0.15% TFA/0.2% aq. TFA 15:85 v/v and the following linear gradient was applied: 0–25 min: A/B 15:85–50:50, 25–33 min: 50:50–95:5, 33–40 min: 95:5 (isocratic). The radiochemical purity was 97% (*t*_R_ = 24 min) (cf. Figure 3). For the analysis of [^3^H]**32**, 3 µL of the preliminary stock solution were added to 100 µL of a 20 µM solution of **32** in acetonitrile/0.05% aq. TFA 15:85 v/v. The following linear gradient was applied: 0–20 min: A/B 15:85–60:40, 20–30 min: 60:40–95:5, 30–38 min: 95:5 (isocratic). The radiochemical purity was >99% (*t*_R_ = 20 min) (cf. Figure 3). For the analysis of [^3^H]**42**, 2 µL of the preliminary stock solution were added to 100 µL of a 10 µM solution of **42** in acetonitrile supplemented with 0.035% TFA/0.05% aq. TFA 20:80 v/v. The following linear gradient was applied: 0–20 min: A/B 20:80–48:52, 20–30 min: 48:52–95:5, 30–38 min: 95:5 (isocratic). The radiochemical purity was >99% (*t*_R_ = 18 min) (cf. Figure 3). Prior to the quantification of the activity of [^3^H]**32**, each of the preliminary stock solutions of this radioligand (2 × 800 µL) was equally split into two 2‐mL vessels, followed by the addition of EtOH/water 3:2 v/v (600 µL) to each vessel, resulting in four preliminary stock solutions with a volume of 1000 µL each. To quantify the activity of the radioligands and to determine the molar concentration, 2 × 2 µL of the preliminary stock solution were added to 998 μL of DMSO/H_2_O 1:1 v/v ([^3^H]**32**) or DMSO/H_2_O 4:1 v/v ([^3^H]**26**, [^3^H]**42**), and 4 × 2 µL ([^3^H]**32**) or 4 × 10 µL ([^3^H]**26**, [^3^H]**42**) of these dilutions were added to 3 mL of liquid scintillator (Rotiszint Routine; Carl Roth, Karlsruhe, Germany) in a 5‐mL scintillation vial followed by measurement with a Tri‐Carb 3100TR liquid scintillation counter (PerkinElmer). The activity concentration of [^3^H]**26** was adjusted to 15 MBq/mL by the addition of EtOH/H_2_O 7:3 v/v (9.2 µL), resulting in a final stock with a concentration of 5 μM (volume: 568 µL). The activity concentration of [^3^H]**32** was adjusted to 27.75 MBq/mL by the addition of EtOH/H_2_O 3:2 v/v to the preliminary stock solutions, resulting in a final stock with a concentration of 7.5 μM and a total volume of 1854, 1249, 1425, and 1209 µL. The activity concentration of [^3^H]**42** was adjusted to 37 MBq/mL by the addition of EtOH/H_2_O 8:2 v/v (113.3 µL), resulting in a final stock with a concentration of 10 μM (volume: 607 µL). Radiochemical yields: [^3^H]**26**, 8.52 MBq (0.23 mCi), 15.4%; [^3^H]**32**, 159.2 MBq (4.3 mCi), 61.7%; [^3^H]**42**, 22.47 MBq (0.607 mCi), 46.7%. Molar activities: as the supplier of the labeling reagents [^3^H]**31** and [^3^H]**2** (Novandi, Södertälje, Sweden) provides a precisely determined molar activity and due to the fact that the radioligands bear exactly one tritiated propionyl residue or one tritiated methyl group originating from the labeling reagent, the molar activities of [^3^H]**26**, [^3^H]**32** and [^3^H]**42** were defined to be equal to the molar activity of the labeling reagents, amounting to 3.7 TBq/mmol or 3.0 TBq/mmol, respectively (it is assumed that under the mild reaction conditions the carbon‐tritium bonds remained intact). The molar activities of the labeling reagents were determined by LC‐MS analysis. Coinjection of non‐radioactive succinimidyl propionate or non‐radioactive methyl nosylate allowed the quantification of the incorporated tritium (Novandi, Södertälje, Sweden).

##### 4.1.2.4 Determination of LogD_7.4_

The determination of the distribution coefficients logD_7.4_ of the radioligands [^3^H]**26**, [^3^H]**32**, and [^3^H]**42** was accomplished according to a reported procedure that was modified as required [47]. Initially, PBS (pH 7.4) presaturated with 1‐octanol and 1‐octanol presaturated with PBS were prepared. 125 µL of a 8 nM ([^3^H]**26**, [^3^H]**32**) or 500 µL of a 14 nM ([^3^H]**42**) radioligand solution in PBS were added to a mixture of 1‐octanol (500 µL) and PBS (375 µL) or neat 1‐octanol (500 µL) in a 2 mL HPLC vial (Agilent, article number 5182‐0714) with a screw cap with septum (Agilent, article number 5182‐0717). After vortexing (2 min) and centrifuging (5 min, 1000 rpm, rt; centrifuge 5804 R, Eppendorf, Hamburg, Germany; note: for centrifugation the HPLC vial was placed in a 5 mL polypropylene tube), 3 × 10 µL of the upper phase (1‐octanol) were transferred into a scintillation vial with 3 mL scintillation liquid (Rotiszint Routine, Carl Roth, Karlsruhe, Germany). To obtain the sample of the lower phase (PBS), the HPLC vial was turned upside down, and approximately 350 µL of the lower phase was carefully removed using a syringe with a cannula and transferred into a 1.5‐mL reaction vessel. 3 × 100 µL of the aqueous phase were subjected to measurement using a Beckman LS 6500 beta‐counter (Beckman Coulter, Krefeld, Germany). The experiments were performed in triplicate. The disintegration per minute (dpm) values were averaged and transformed to a distribution coefficient logD_7.4_ according to logD_7.4_ = log((*A*_octanol_ × 10)/*A*_aqueous_), where *A*_octanol_ is the mean of the dpm values obtained from the samples of the 1‐octanol phase, and *A*_aqueous_ is the mean of the dpm values obtained for the samples of the aqueous phase.

##### 4.1.2.5 Chemical Stability

The chemical stability of compounds **32**, **26**, and **42** was investigated in PBS (adjusted to pH 7.4) at 22°C using 1.5‐mL polypropylene reaction vessels. The incubation was started by the addition of 1.5 µL of a 10 mM stock solution (in DMSO) of each investigated compound to 148.5 µL of PBS (final concentration: 100 µM). After time periods of 0, 6, and 24 h, an aliquot (30 µL) was withdrawn and added to 30 µL of acetonitrile/1% aq. TFA 1:1 v/v to obtain a compound solution with a concentration of 50 µM. 50 µL of this solution was subjected to analytical RP‐HPLC analysis. Under the same conditions, neat PBS was kept and treated in a separate vessel and was diluted and analyzed as the samples containing the investigated compounds to obtain blank chromatograms (vehicle controls) for 0 and 24 h.

### 4.2 Radioligand Adsorption Test

The adsorption of the radioligands [^3^H]**26**, [^3^H]**32**, [^3^H]**42**, and [Lys^4^‐[^3^H]propionyl]hPYY was studied using five different materials: 1.5‐mL polypropylene reaction vessels (Sarstedt, product number 72.690.001), siliconized (SigmaCote, Sigma Aldrich) 1.5‐mL polypropylene reaction vessels, glass inserts for HPLC vials (Agilent, product number 5181–1270), white/transparent polystyrene 96‐well plates (VWR 732‐3740), and the same 96‐well plates with a confluent cell layer (CHO‐K1 cells) at the bottom of the wells. To investigate the adsorption, 2 nM solutions of [^3^H]**26** or [^3^H]**32**, 13 nM solutions of [^3^H]**42**, and 1 nM solutions of [Lys^4^‐[^3^H]propionyl]hPYY in neat PBS (pH 7.4) and PBS supplemented with 1% BSA were prepared in 5‐mL (Eppendorf, product number 0030119401) or 1.5‐mL polypropylene reaction vessels (Sarstedt). 200 µL of these solutions were immediately (within 1 min) transferred to the vessels under study, followed by transferring 3 × 10 µL of each solution to a 1450‐401 96‐well sample plate (PerkinElmer, Rodgau, Germany) to determine the initial radioligand activity (100% values) (note: the 1450‐401 sample plate was used for the measurement of radioactivity). The vessels under study were incubated at rt for 2 h under gentle shaking, followed by transferring 3 × 10 µL of each sample to the 1450‐401 sample plate. Rotiszint Routine (Carl Roth) (200 µL) was added to each well and the plate was sealed with a transparent sealing tape (Greiner Bio‐One EASYseal, part no. 676001; Greiner Bio‐One, Frickenhausen, Germany) and subjected to measurement of the radioactivity (dpm) with a MicroBeta2 plate counter (PerkinElmer, Rodgau, Germany). The dpm values of the triplicates were averaged, and the mean values of the 2 h incubation samples were normalized based on the dpm values of the 100% reference samples. Each experiment was repeated at least three times. The relative activities of individual experiments were converted to mean values ± SEM.

### 4.3 Pharmacological Assays

#### 4.3.1 Cell Culture

Cells were cultured in T25, T75, or T175 flasks (Sarstedt, Nümbrecht, Germany) in a humidified atmosphere (95% air, 5% CO_2_) at 37°C. SK‐N‐MC neuroblastoma cells (obtained from the American Type Culture Collection, ATCC HTB‐10) were maintained in EMEM supplemented with 7.5% FBS. CHO‐hY_2_R cells (obtained from Perkin Elmer, Rodgau, Germany) were cultured in Ham's F‐12 supplemented with 7.5% FBS and G418 (400 μg/mL). CHO‐hY_4_‐G_qi5_‐mtAEQ cells [48] were cultured in Ham's F‐12 supplemented with 7.5% FBS, hygromycin (400 µg/mL), zeocin (250 µg/mL), and G418 (400 µg/mL). HEC‐1B‐hY_5_ cells [34] were maintained in EMEM supplemented with 7.5% FBS and G418 (400 µg/mL). CHO‐K1 cells were cultured in Ham's F‐12 supplemented with 7.5% FBS.

#### 4.3.2 HEC‐1B‐hY_5_R Cell Membrane Preparations

HEC‐1B‐hY_5_R cells were grown in 175 cm^2^ cell culture dishes (ref 83.3903, Sarstedt) to ca. 90% confluency. After washing two times with cold PBS (4°C), the cells were scraped off in Tris buffer (50 mM Tris HCl, 1 mM EDTA, pH 7.4) and centrifuged at 200*g* at 4°C for 5 min. The cell pellet was resuspended in the aforementioned Tris buffer supplemented with SIGMAFAST protein inhibitor cocktail (Sigma Aldrich) (1 tablet in 100 mL buffer). Under cooling in ice‐water, cells were disrupted with an Ultraturrax (IKA, Staufen, Germany) applying six 5 s treatments with breaks of 30 s in between. The resulting homogenate was centrifuged at 1500*g* at 4°C for 15 min. The supernatant was then centrifuged with an Optima XPN‐80 ultracentrifuge (Beckman Coulter, Brea, CA) at 119,000*g* at 4°C for 30 min. The pellet was resuspended in Tris buffer (50 mM Tris HCl, 1 mM EDTA, pH 7.4), followed by aliquoting and storing at −80°C. The protein concentration was determined according to the Bradford method [49] in transparent 96‐well plates (Greiner Cellstar 655180, Greiner Bio‐One, Frickenhausen, Germany) using BSA as a standard. The protein concentration amounted to 1.05 ± 0.03 mg/mL (mean value ± SEM from three different sample dilutions).

#### 4.3.3 Buffers Used for Binding Assays

*Buffer II* (used for binding experiments at the Y_1_R, Y_2_R, and Y_5_R): an isotonic HEPES buffer (10 mM HEPES, 150 mM NaCl, 25 mM NaHCO_3_, 2.5 mM CaCl_2_, 5 mM KCl, pH 7.4) supplemented with 1% BSA and 0.1 mg/mL bacitracin (Serva).

*DPBS* (used for binding experiments at Y_4_R): Dulbecco's phosphate‐buffered saline with calcium and magnesium (1.8 mM CaCl_2_, 2.68 mM KCl, 1.47 mM KH_2_PO_4_, 3.98 mM MgSO_4_, 137 mM NaCl, 8.06 mM Na_2_HPO_4_, pH 7.4) supplemented with 1% BSA and 0.1 mg/mL bacitracin.

#### 4.3.4 Radioligand Binding Assays

To determine Y_5_R binding affinities of **1**, **3**, **26**–**30**, **32**–**34**, **37**, **38**, **41**, **42, 45**, and His‐hPYY, and Y_1_R, Y_2_R, and Y_4_R binding affinities of **26**, **28**, **32**, **37**, **41**, **42**, and **45**, previously described radioligand competition binding assays were performed with intact YR‐expressing cells at 22 ± 2°C. The binding assays were previously validated by the determination of binding affinities of the reference ligands pNPY (Y_1_R, Y_2_R, and Y_5_R) and hPP (Y_4_R), which were in good agreement with previously reported binding affinities (for determined and reference p*K*_i_ or *K*_i_ values of pNPY (Y_1_R, Y_2_R, Y_5_R) see Konieczny et al. [50]; for determined and reference p*K*_i_ values of hPP (hY_4_R) see Gleixner et al. [29]).

##### 4.3.4.1 Y_1_R Binding

Competition binding assays at Y_1_R‐expressing SK‐N‐MC neuroblastoma cells were performed as previously described using [^3^H]UR‐MK299 (*K*_d_ = 0.058 nM [31]) as radioligand, but the used concentration of the radioligand was 0.075 nM instead of 0.15 nM [30]. Due to low radioligand displacement, no curve fitting was performed for the investigated compounds **26**, **28**, **32**, **37**, **41**, **42**, and **45** (studied at concentrations up to 10 µM).

##### 4.3.4.2 Y_2_R Binding

Competition binding assays at CHO‐hY_2_R cells were performed as previously described using [Lys^4^‐[^3^H]propionyl]hPYY (*K*_d_ = 0.16 nM, concentration: 0.4 nM) as radioligand [31]. Due to low radioligand displacement, no curve fitting was performed for the investigated compounds **26**, **28**, **32**, **37**, **41**, **42**, and **45** (studied at concentrations up to 10 µM).

##### 4.3.4.3 Y_4_R Binding

Competition binding experiments at CHO‐hY_4_R‐G_qi5_‐mtAEQ cells using [^3^H]UR‐JG102 as radioligand (*K*_d_ = 0.11 nM, concentration: 0.25 nM) were performed as previously described [29]. Due to low radioligand displacement, no curve fitting was performed for the investigated compounds **26**, **28**, **32**, **37**, **41**, **42**, and **45** (studied at concentrations up to 10 µM).

##### 4.3.4.4 Y_5_R Binding

Competition binding studies at HEC‐1B‐hY_5_R cells using [Lys^4^‐[^3^H]propionyl]pNPY (*K*_d_ = 11 nM [24], concentration: 5 nM) as radioligand were performed as previously reported [51]. Total binding data (dpm, including total binding in the absence of competitor) were plotted against log(concentration competitor) and analyzed by a four‐parameter logistic equation (log(inhibitor) vs. response‐variable slope, GraphPad Prism 5, GraphPad Software, San Diego, CA, USA) to obtain pIC_50_ and IC_50_ values (in the case of His‐hPYY the following constraint was applied: bottom (lower curve plateau) >0). To plot average data from individual binding experiments, data were normalized (100% = specifically bound radioligand in the absence of competitor, 0% = non‐specifically bound radioligand). pIC_50_ and IC_50_ values from individual experiments were converted to p*K*_i_ and *K*_i_ values according to the Cheng‐Prusoff equation [52] (logarithmic form in the case of p*K*_i_ values).

#### 4.3.5 Y_5_R Binding Assays With [^3^H]**26**, [^3^H]**32**, and [^3^H]**42**

##### 4.3.5.1 Saturation Binding Experiments

Saturation binding of the radioligands [^3^H]**26**, [^3^H]**32**, and [^3^H]**42** was studied at adherent and suspended intact HEC‐1B‐hY_5_R cells. Y_5_R binding of [^3^H]**32** was additionally studied using membrane preparations of HEC‐1B‐hY_5_R cells. All experiments were performed in triplicate. Except for the saturation binding experiments with [^3^H]**32** at adherent cells in 24‐well plates (performed twice), at least three individual experiments were carried out. To reduce radioligand consumption, [^3^H]**26** was diluted 1:2 with **26,** and [^3^H]**42** was diluted 1:3 with **42** (for the purpose of easier reading, these mixtures are denoted [^3^H]**26d** and [^3^H]**42d** in the following). All samples were incubated at 22 ± 2°C in the dark under gentle shaking for 60 min ([^3^H]**32**, cell membranes), 90 min ([^3^H]**32**, adherent cells and cell suspensions), or 120 min ([^3^H]**26d**, [^3^H]**42d**). *Buffer II* served as a binding buffer throughout. In the case of experiments performed with cell suspensions, HEC‐1B‐hY_5_R cells were grown in T75 culture flasks to reach a confluency of 80%–90% on the day of the experiment. The culture medium was removed, and the adherent cells were washed twice with *buffer II* (for this purpose, the buffer was not supplemented with BSA and bacitracin). The cells were scraped off the flask using a cell scraper, and centrifuged at 300*g* at rt for 5 min. The supernatant was discarded, and the cells were resuspended in *buffer II* at 800,000 cells/mL. Samples involving suspended cells or membrane preparations were prepared and incubated in 96‐well polypropylene plates (Brand 701330 Wertheim, Germany). The same filtration procedure was applied to experiments with suspended cells and membrane preparations to separate free from cell‐ or membrane‐bound radioligand and to measure radioactivity: after completed incubation, cells or membranes were collected on GF/C filter mats (0.26 mm; Whatman, Maidstone, UK) (pretreated with 0.3% polyethylenimine for 30 min) using an in‐house manufactured harvester for 96‐well plates (precision engineering workshop of the University of Regensburg, Regensburg, Germany), and the wells of the plate and the cells or membranes on the filter mat were immediately washed twice with ice‐cold PBS (2 × 200 µL). Filter pieces for each well were punched out and transferred into a 1450‐401 96‐well sample plate (PerkinElmer, Rodgau, Germany) followed by the addition of Rotiszint Routine (Carl Roth) (200 μL). The plates were sealed with a transparent sealing tape (Greiner Bio‐One EASYseal, part no. 676001; Greiner Bio‐One) and shaken in the dark overnight before measurement. Radioactivity (dpm) was measured with a MicroBeta2 plate counter equipped with six pairs of photomultiplier tubes (PerkinElmer). For experiments involving adherent cells, HEC‐1B‐hY_5_R cells were seeded in white 96‐well plates with clear bottom (VWR 732‐3740) or transparent 24‐well plates (supplier no. 662160, Greiner‐Bio One) at a density that gave 80%–90% confluency on the day of the experiment. The culture medium was aspirated and the cells were washed with *buffer II* (without BSA and bacitracin) (200 µL for 96‐well plates, 500 µL for 24‐well plates) followed by covering with *buffer II* and the addition of the radioligand. After completed incubation, the liquid was aspirated and the wells were washed twice with ice‐cold PBS (2 × 200 µL for 96‐well plates, 2 × 500 µL for 24‐well plates) followed by the addition of lysis solution (urea (8 M), acetic acid (3 M) and Triton‐X (1%) in water) (25 µL for 96‐well plates, 200 µL for 24‐well plates). After shaking for at least 20 min, liquid scintillator (UltimaGold, PerkinElmer) (200 µL) was added to the 96‐well plates, which were then sealed with transparent sealing tape (permanent seal for microplates, Perkin Elmer, prod. No. 1450‐461). The sealed plates were turned upside down several times to achieve complete mixing of scintillator and lysis solution and were kept in the dark for at least 2 h before measurement of the radioactivity (dpm) with the MicroBeta2 plate counter. For 24‐well plates, the lysates were transferred into scintillation vials filled with liquid scintillator (Rotiszint Routine, Carl Roth) (3 mL) and measured in a Beckman LS 6500 beta‐counter (Beckman Coulter, Krefeld; Germany).

##### 4.3.5.2 Saturation Binding Experiments With [^3^H]**26d**

A 96‐well polypropylene plate was prefilled with cell suspension (80 µL) or a white/clear 96‐well plate with adherent cells was prefilled with *buffer II* (80 µL) after initial washing of the cells. For the determination of total binding, *buffer II* (10 µL) and a 10‐fold concentrated (relative to the final concentration) solution of [^3^H]**26d** in *buffer II* (10 µL) were added. To determine non‐specific binding, a 100 µM solution of His‐hPYY (for suspended cells) or a 250 µM solution of His‐hPYY (for adherent cells) (final concentration: 10 µM or 25 µM) in *buffer II* (10 µL) and a 10‐fold concentrated solution of [^3^H]**26d** in *buffer II* (10 µL) were added. The samples were incubated under shaking for 120 min, followed by cell harvesting (cell suspensions) or washing and cell lysis (adherent cells), and further processing as described above.

##### 4.3.5.3 Saturation Binding Experiments With [^3^H]**32**

For saturation binding experiments in 96‐well assay format, the polypropylene plate was prefilled with cell suspension (160 µL), or a white/clear 96‐well plate with adherent cells was prefilled with *buffer II* (160 µL) after initial washing of the cells. For the determination of total binding, *buffer II* (20 µL) and a 10‐fold concentrated (relative to the final concentration) solution of [^3^H]**32** in *buffer II* (20 µL) were added. To determine non‐specific binding, a 100 µM solution of His‐hPYY (final concentration: 10 µM) in *buffer II* (20 µL) and a 10‐fold concentrated solution of [^3^H]**32** in *buffer II* (20 µL) were added. The samples were incubated under shaking for 90 min, followed by cell harvesting (cell suspensions) or washing and cell lysis (adherent cells), and further processing as described above.

For saturation binding experiments with [^3^H]**32** at HEC‐1B‐hY_5_R cell membranes, membrane aliquots (2 × 200 µL) were thawed on the day of the experiment. The suspension was centrifuged at 9000*g* at 4°C for 5 min, followed by discarding the supernatant and resuspension of the pellet in *buffer II* (2,2 mL). The membrane dilution was homogenized using a 0.4 mm diameter needle (Sterican, B. Braun, Melsungen, Germany). Experiments were performed as saturation binding experiments with suspended HEC‐1B‐hY_5_R cells with minor modifications, using 20 μL of the membrane dilution in *buffer II* (soluble protein content: 191 μg/mL) instead of 160 μL of the cell suspension. To keep the total sample volume at 200 μL, the difference in volume (140 µL) was compensated for by the addition of *buffer II*. The samples were prepared as follows: 160 μL of *buffer II* (determination of total binding) or 140 µL of *buffer II* (determination of non‐specific binding) were added to the empty wells of the polypropylene 96‐well plate, followed by the addition of 20 µL of a 70 µM solution of His‐hPYY (final concentration: 7 µM) in *buffer II* to the wells assigned for the determination of non‐specific binding, and the addition of a 10‐fold concentrated solution of [^3^H]**32** in *buffer II* (20 µL) to all wells. Samples were incubated for 60 min, followed by processing as described for the assay with suspended HEC‐1B‐hY_5_R cells.

For saturation binding experiments with [^3^H]**32** at adherent cells in 24‐well plates, 200 µL of *buffer II* were added after initial washing, followed by the addition of 25 µL of *buffer II* (determination of total binding) or 25 µL of a 70 µM solution of His‐hPYY (final concentration: 7 µM) in *buffer II* (determination of non‐specific binding), and the addition of 25 µL of a 10‐fold concentrated (relative to the final concentration) solution of [^3^H]**32** in *buffer II*. The samples were incubated under shaking for 90 min, followed by washing, cell lysis, and further processing as described above.

##### 4.3.5.4 Saturation Binding Experiments With [^3^H]**42d**

A 96‐well polypropylene plate was prefilled with cell suspension (160 µL) or a white/clear 96‐well plate with adherent cells was prefilled with *buffer II* (160 µL) after initial washing of the cells. For the determination of total binding, *buffer II* (20 µL) and a 10‐fold concentrated (relative to the final concentration) solution of [^3^H]**42d** in *buffer II* (20 µL) were added. To determine non‐specific binding, a 100 µM solution of His‐hPYY (final concentration: 10 µM) in *buffer II* (20 µL) and a 10‐fold concentrated solution of [^3^H]**42d** in *buffer II* (20 µL) were added. The samples were incubated under shaking for 120 min, followed by cell harvesting (cell suspensions) or washing and cell lysis (adherent cells), and further processing as described above.

##### 4.3.5.5 Processing of Saturation Binding Data

Specific binding data, obtained by subtracting triplicate dpm mean values of non‐specific binding from triplicate dpm mean values of total binding, were plotted against the free radioligand concentration and analyzed using a two‐parameter equation describing hyperbolic single‐site binding (one‐site, specific binding, GraphPad Prism 5) to obtain *K*_d_ values. The free concentrations of the radioligands (nM) were calculated by subtracting the amount of non‐specifically and specifically bound radioligand (nM) (calculated from non‐specifically or specifically bound radioligand in dpm, the molar activity, and the volume per well) from the total radioligand concentration. To obtain p*K*_d_ values, individual *K*_d_ values were converted to p*K*_d_ values, followed by the calculation of mean p*K*_d_ values ± SEM.

##### 4.3.5.6 Competition Binding Experiments With [^3^H]**32**

Competition binding experiments at HEC‐1B‐hY_5_R cell membranes with [^3^H]**32** (*K*_d_ = 1.6 nM) were performed according to the procedure described for saturation binding experiments with [^3^H]**32** at cell membranes (see *Saturation Binding Experiments With* [^3^H]**32**, but a constant radioligand concentration of 3 nM was used and the compound of interest (competitor) was added to the samples at varying concentrations (sample preparation: 140 µL of *buffer II* + 20 µL of a 10‐fold concentrated solution of competitor in *buffer II* + 20 µL of a 30 nM solution of [^3^H]**32** in *buffer II* + 20 µL of membrane suspension). For the determination of total binding in the absence of competitor, 20 µL of *buffer II* were added instead of the competitor solution. For the determination of non‐specific binding, 20 µL of a 100 µM solution of His‐hPYY (final concentration: 10 µM) in *buffer II* were added instead of the competitor solution. Samples were incubated for 60 min, followed by processing as described for the saturation binding assays using cell suspensions. Data were processed as described before (*4.3.4 Radioligand Binding Assays – Y*_*5*_ *R Binding*).

#### 4.3.6 Calculation of Propagated Errors

Propagated errors (applying to specifically bound radioligand determined in saturation binding assays) were calculated as described elsewhere [29].

## Funding

The authors have nothing to report.

## Conflicts of Interest

The authors declare no conflicts of interest.

## Supporting information


Supporting File 1

Supporting File 2

## Data Availability

Data that support the findings of this study are available from the corresponding author upon reasonable request.
