# Supplementary material for: Synthesis and Characterization of Tritium‐Labeled Neuropeptide Y Y5 Receptor Ligands Derived From CGP71683A
Source: Arch Pharm (Weinheim). 2026 Aug 17;359(8):e70314. doi: 10.1002/ardp.70314 (PMC13479786; doi:10.1002/ardp.70314)
Supplement: Supplementary file 1 [file ARDP-359-e70314-s002.pdf]

Supporting Information

# Synthesis and Characterization of Tritium-Labeled Neuropeptide Y $Y_5$ Receptor Ligands Derived from CGP71683A

*Franziska Schettler,*[1] *Pierre Koch*[1] *and Max Keller*[1,*]

[1]Institute of Pharmacy, Faculty of Chemistry and Pharmacy, University of Regensburg, Regensburg, Germany

*Correspondence:

Prof. Dr. Max Keller, Institute of Pharmacy, Faculty of Chemistry and Pharmacy, University of Regensburg, Universitätsstraße 31, D-93053, Germany

Email: max.keller@chemie.uni-regensburg.de

**Content**

1. Figures S1-S7 ........ S2

2. RP-HPLC chromatograms of compounds **3**, **26-30**, **32-34**, **37**, **38**, **41**, **42**, and **45** (purity controls) ........ S7

3. $^{1}$H- and $^{13}$C-NMR spectra of compounds **3**, **26-30**, **32-34**, **37**, **38**, **41**, **42**, and **45**, and HSQC and HMBC spectrum of **42** ........ S12

4. HRMS spectra of compounds **3**, **26-30**, **32-34**, **37**, **38**, **41**, **42**, and **45** ........ S35

## 1. Figures S1-S7

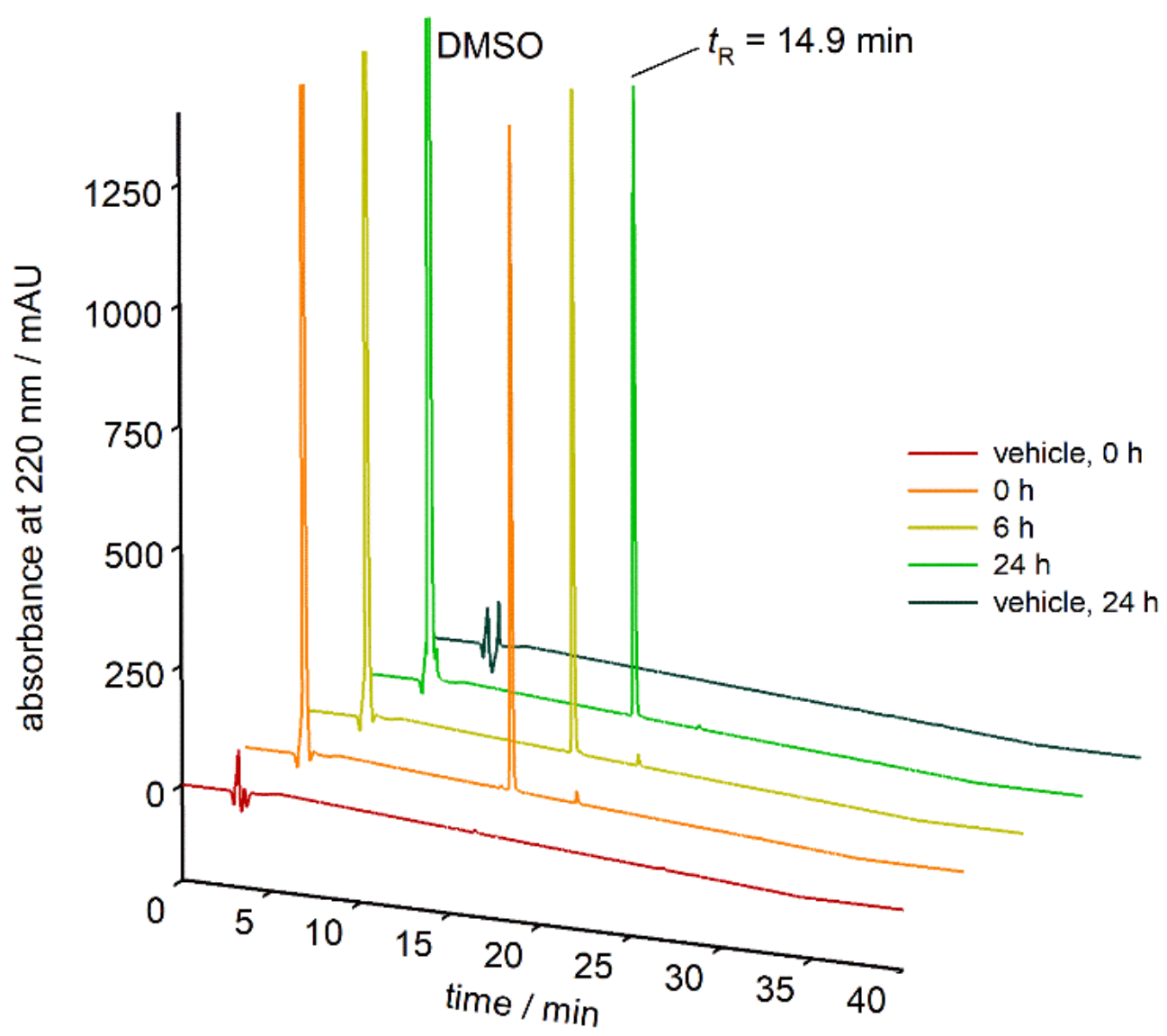


**Figure S1.** Chromatograms of the RP-HPLC analysis of **26** after incubation in PBS pH 7.4 at 22 °C for up to 24 h. **26** showed no decomposition.

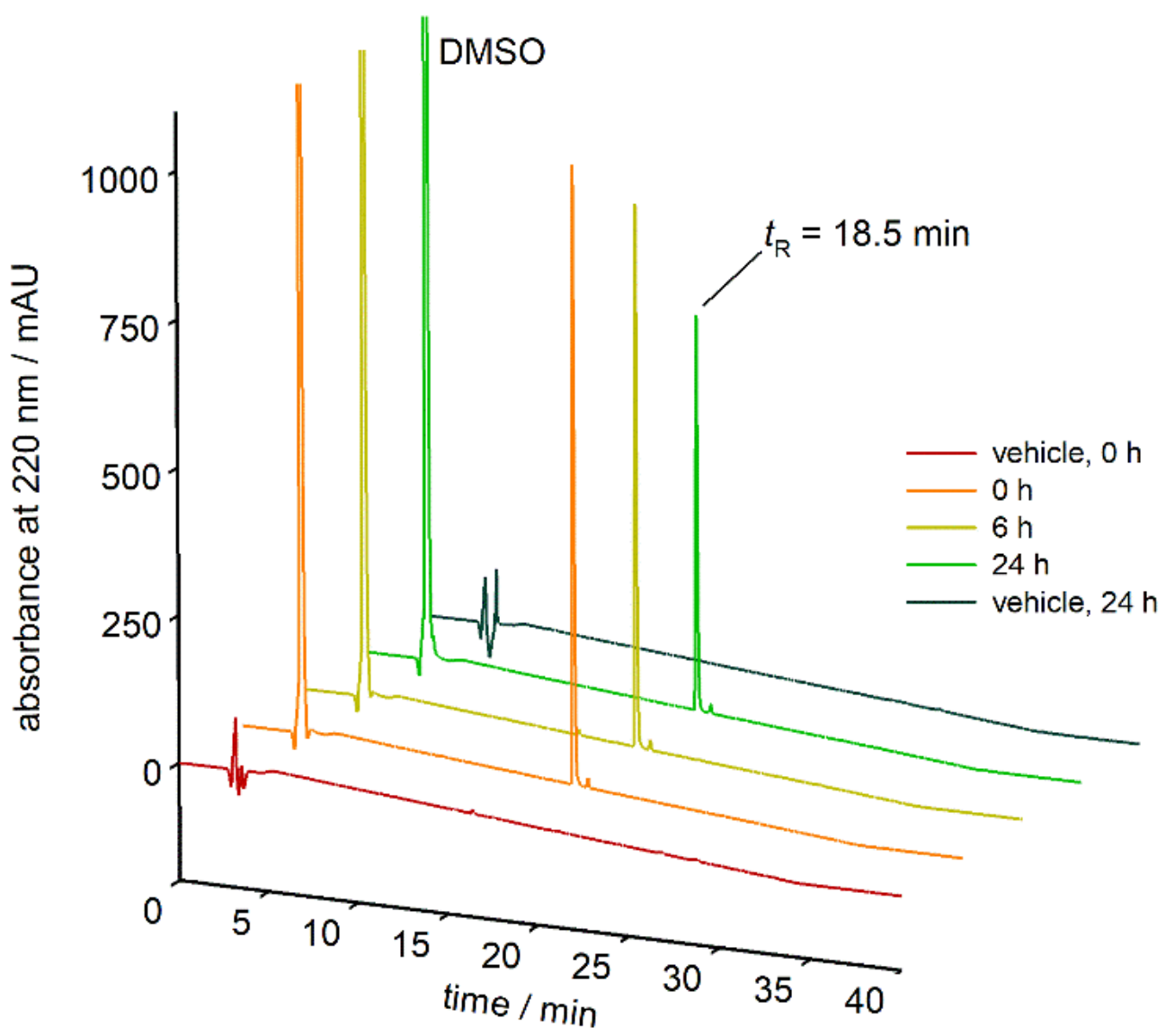


**Figure S2.** Chromatograms of the RP-HPLC analysis of **32** after incubation in PBS pH 7.4 at 22 °C for up to 24 h. **32** showed no decomposition. The decrease in peak height over time is caused by adsorption of **32** to the vessel.

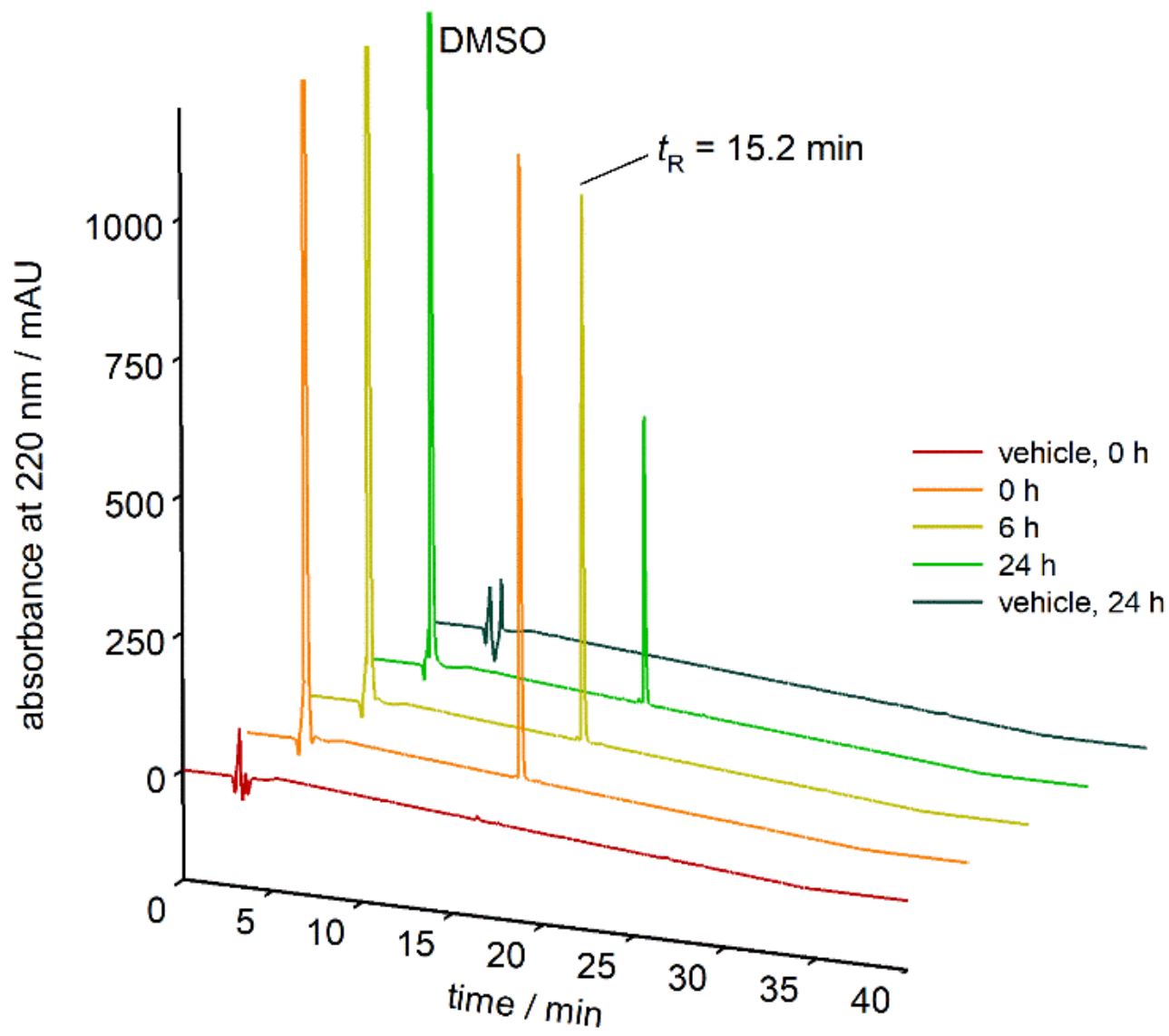


**Figure S3.** Chromatograms of the RP-HPLC analysis of **42** after incubation in PBS pH 7.4 at 22 °C for up to 24 h. **42** showed no decomposition. The decrease in peak height over time is caused by adsorption of **42** to the vessel.

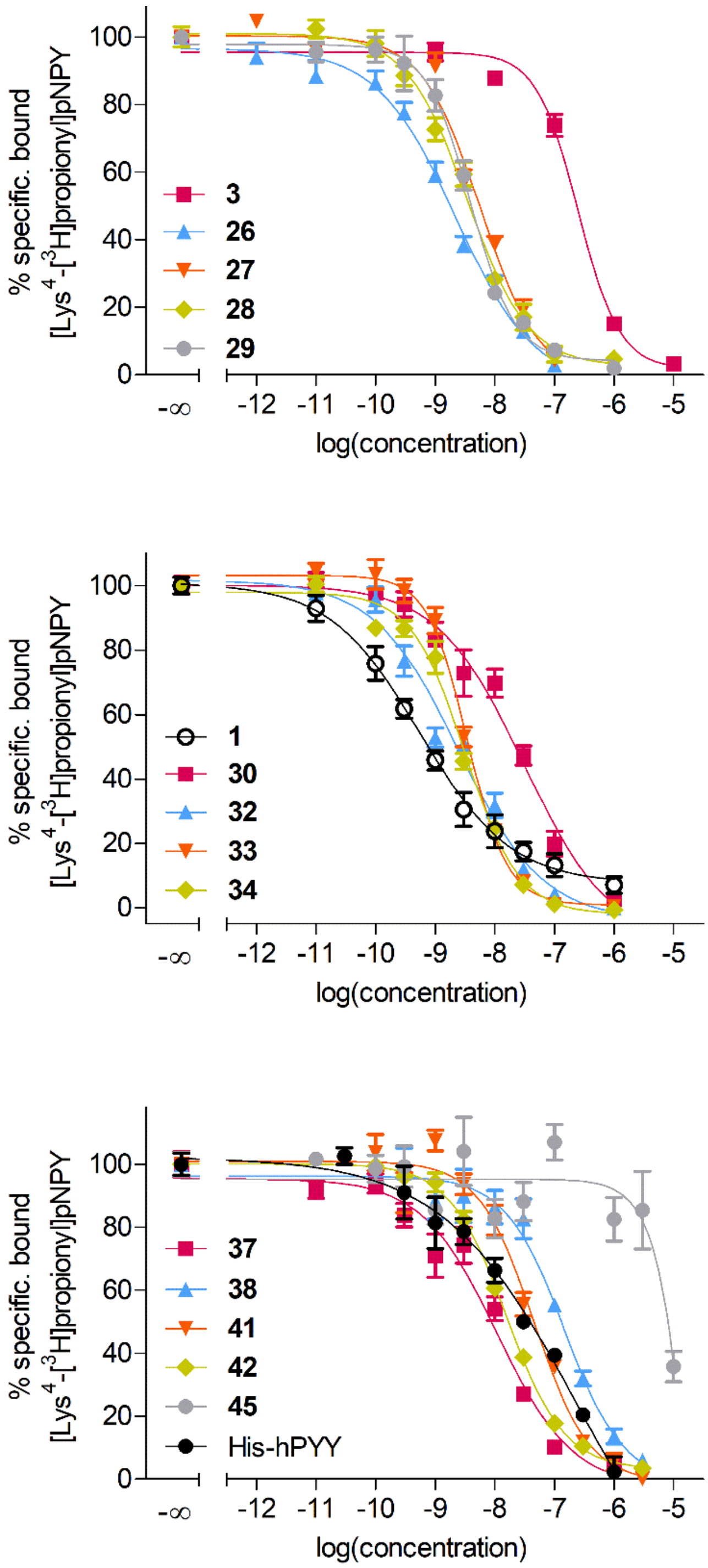


**Figure S4.** Radioligand displacement curves from competition binding experiments with [Lys$^{4}$-[$^{3}$H]propionyl]pNPY ($K_d$ = 11 nM, c = 5 nM) and **1**, **3**, **26**-**30**, **32**-**34**, **37**, **38**, **41**, **42**, **45**, and His-hPYY at intact HEC-1B-hY$_5$R cells. Data represent mean values ± SEM from 3-5 independent experiments (performed in triplicate). For p$K_i$ values, see Table 2 (main article).

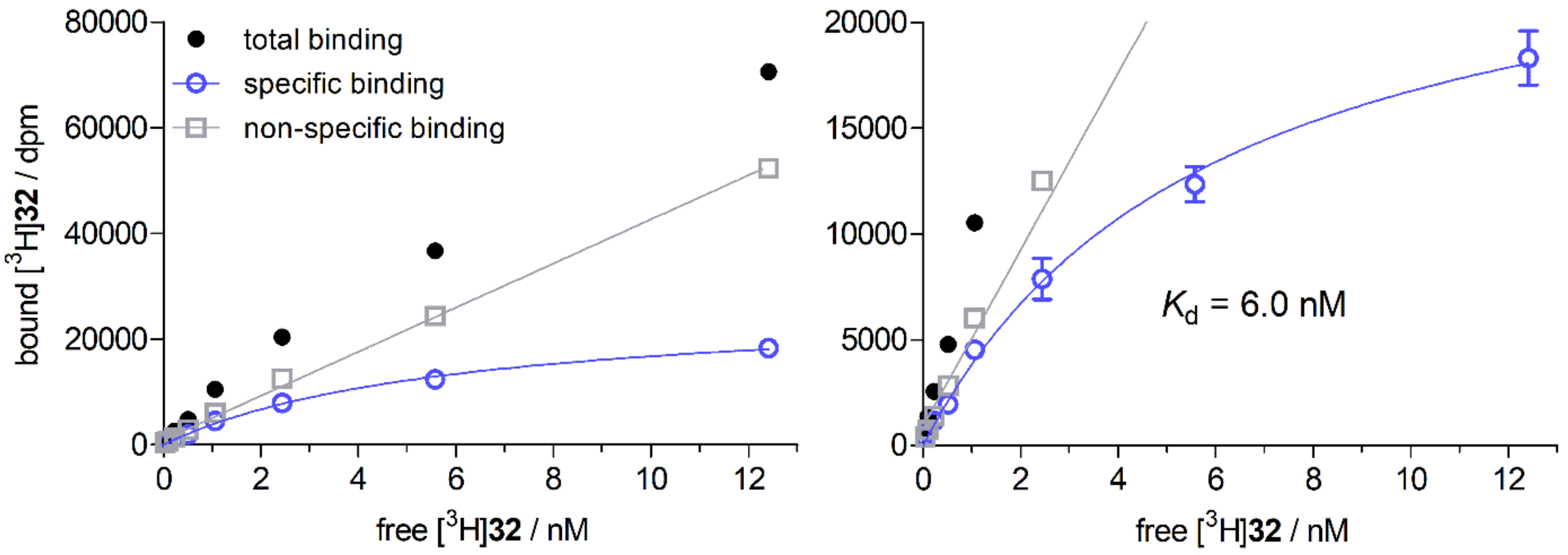


**Figure S5.** Data of a representative binding experiment of [$^3$H]**32** in a 24-well assay format in *buffer II* at 22 ± 2 °C performed at adherent HEC-1B-hY$_5$R cells. The illustration shows total, specific, and non-specific binding on the left and a zoomed-in view, capturing specific binding, on the right. Non-specific binding was determined in the presence of 70 µM His-hPYY. Total and non-specific binding data represent mean values ± SEM. Specific binding data represent calculated values ± propagated error.

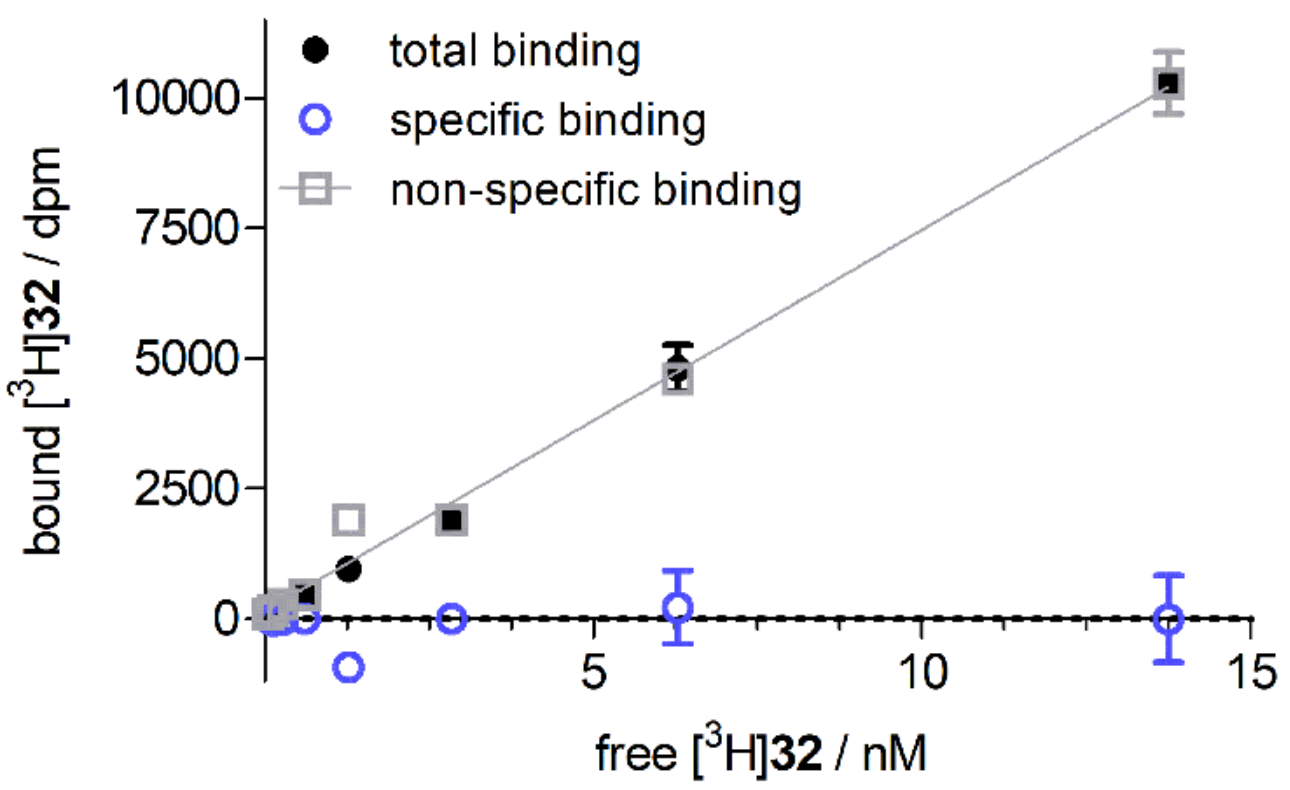


**Figure S6.** Data of a representative binding experiment of [$^3$H]**32** in *buffer II* at 22 ± 2 °C performed at suspended CHO-hY$_2$R cells to demonstrate the high non-specific binding also to cells not expressing Y$_5$R. Total and non-specific binding data represent mean values ± SEM. Specific binding data represent calculated values ± propagated error.

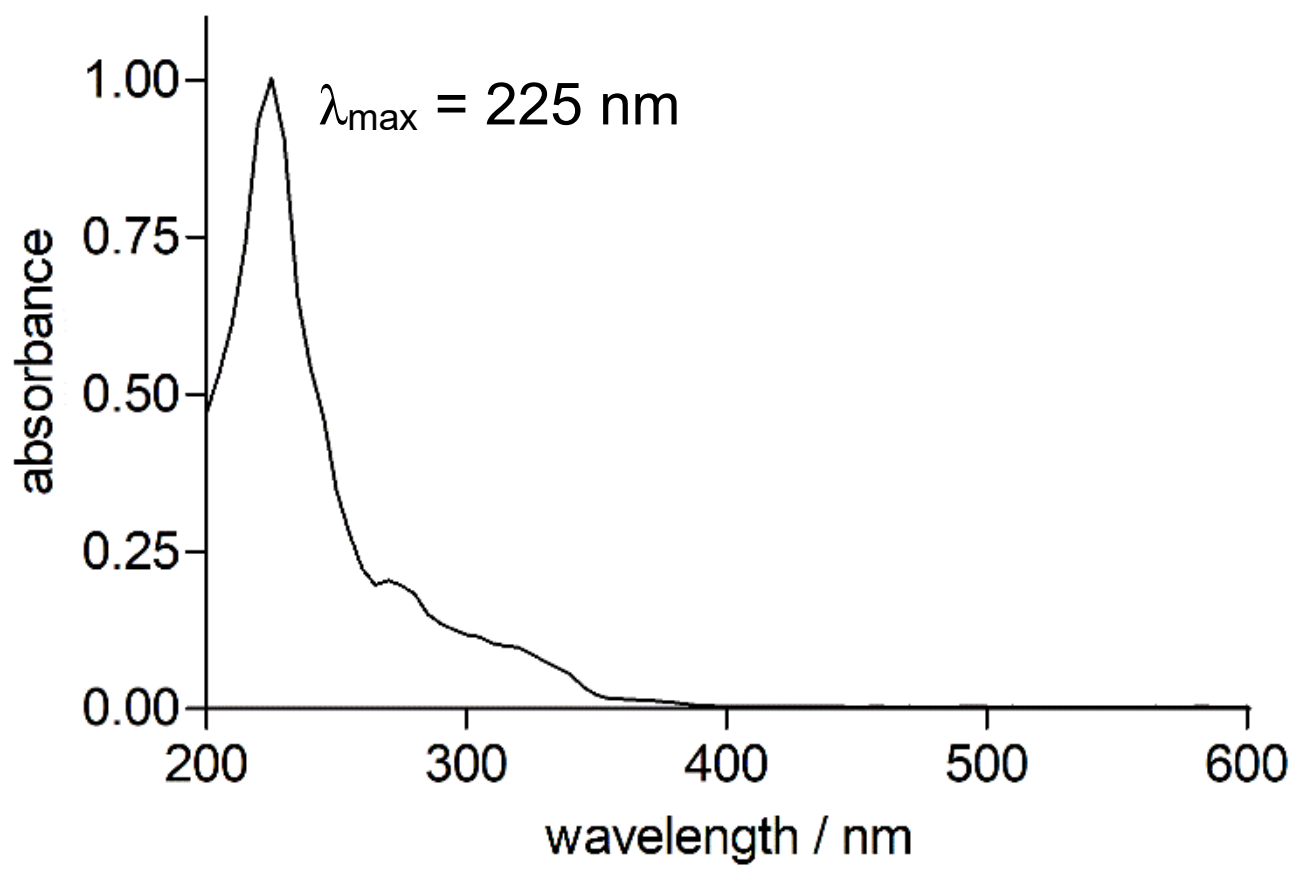


**Figure S7.** UV-Vis absorbance spectrum of **28** (25 µM) in 0.1% aq. TFA/acetonitrile 1.3:1 v/v acquired with a Cary 60 spectrophotometer (Agilent Technologies, Santa Clara, CA).

**2. RP-HPLC chromatograms of compounds 3, 26-30, 32-34, 37, 38, 41, 42, and 45 (purity controls)**

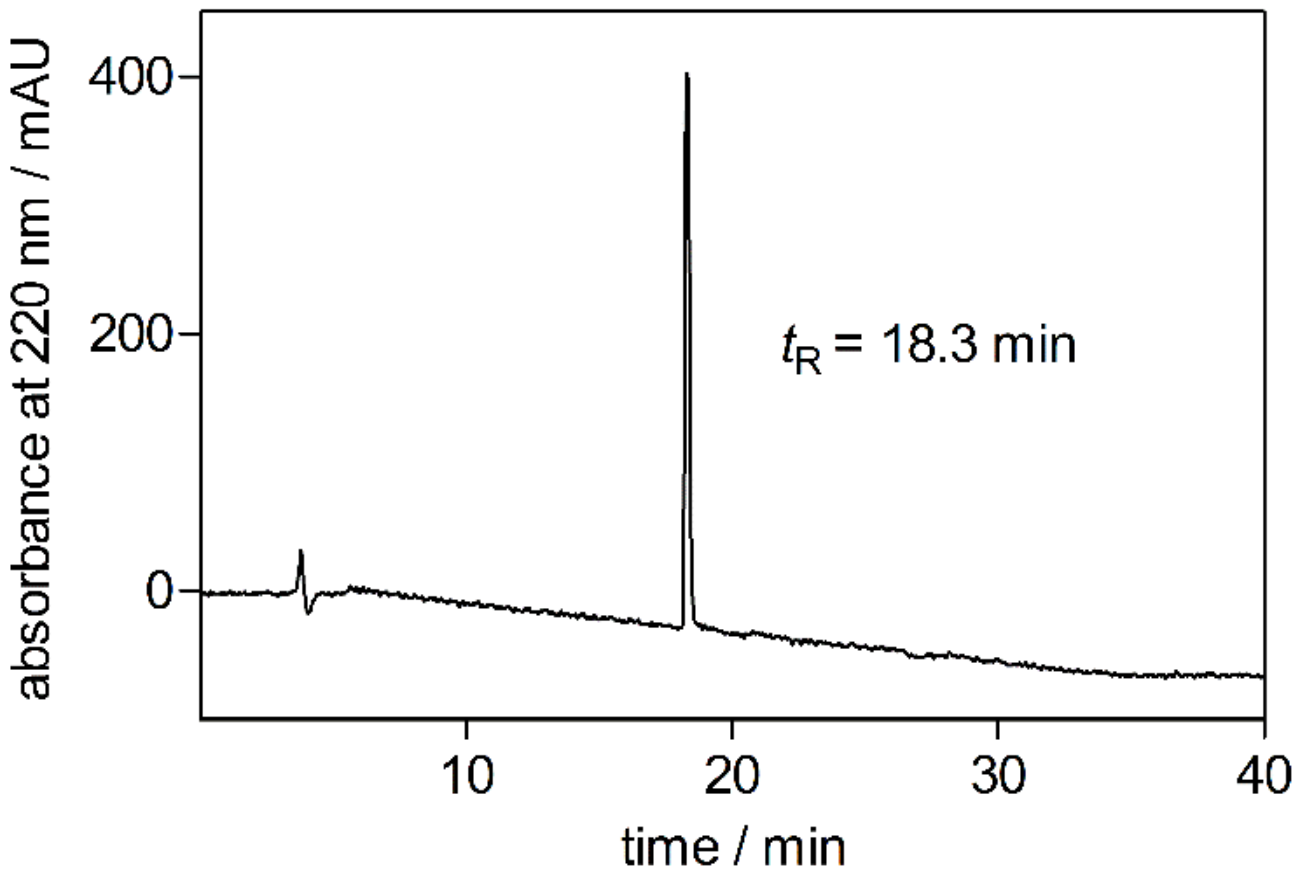


RP-HPLC analysis of compound **3**.

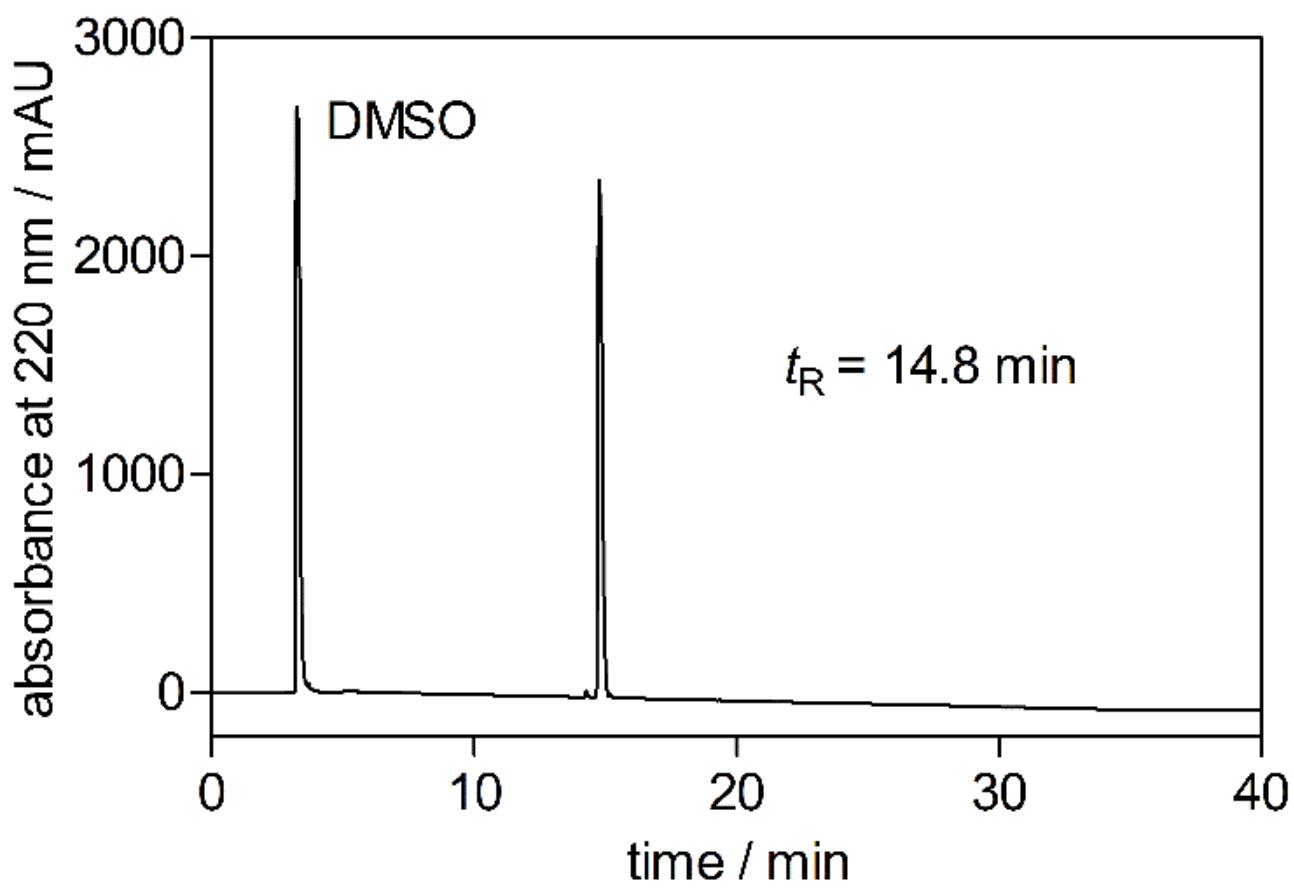


RP-HPLC analysis of compound **26**.

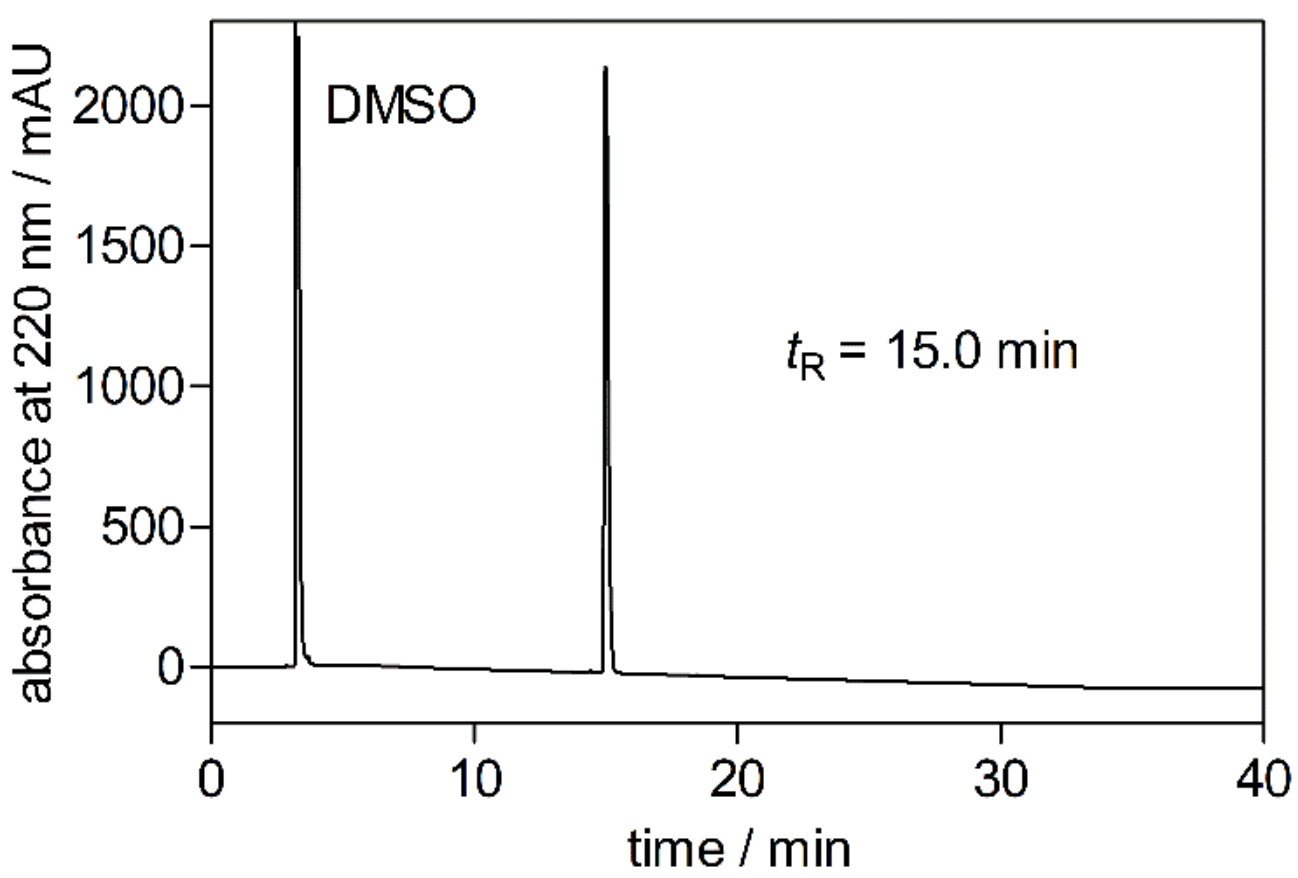


RP-HPLC analysis of compound **27**.

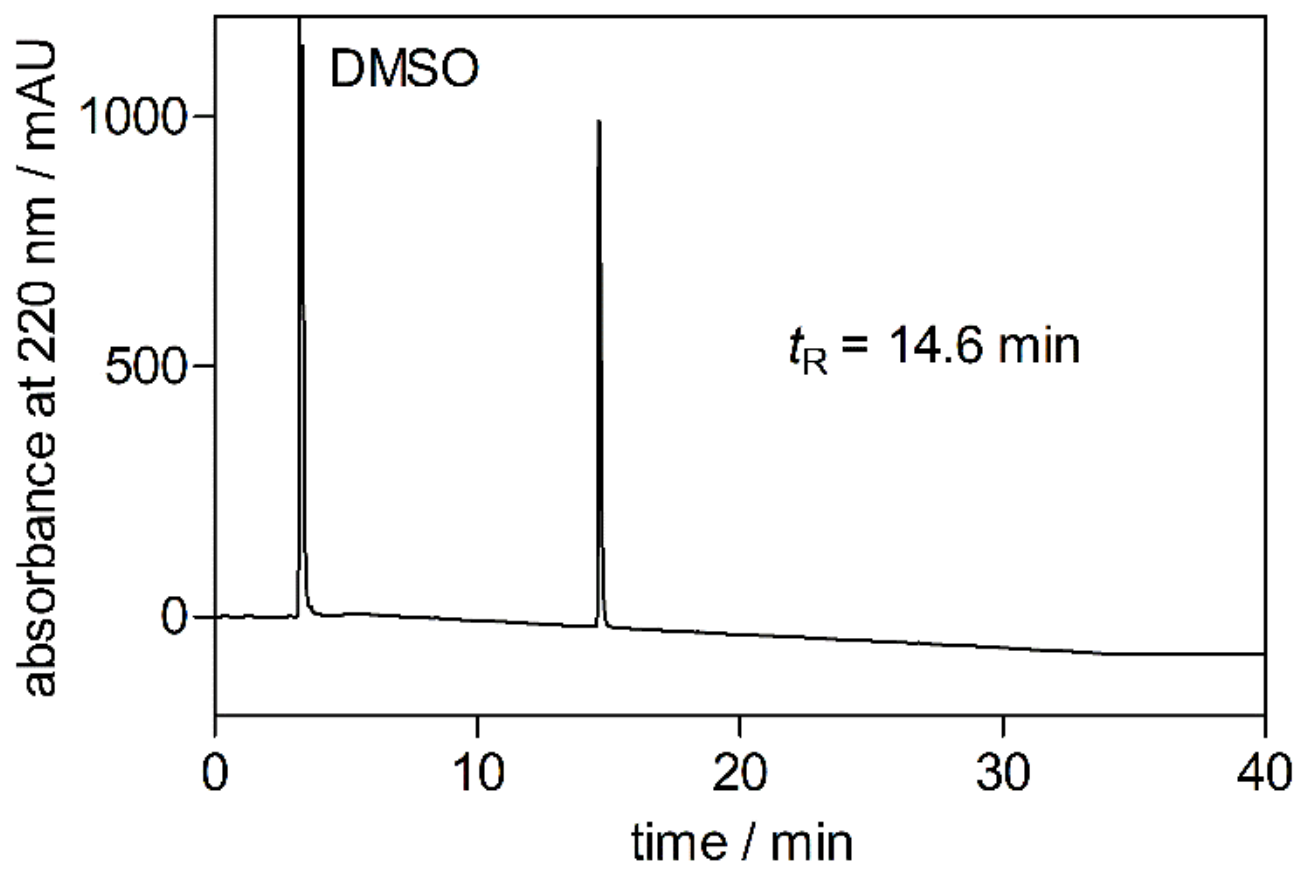


RP-HPLC analysis of compound **28**.

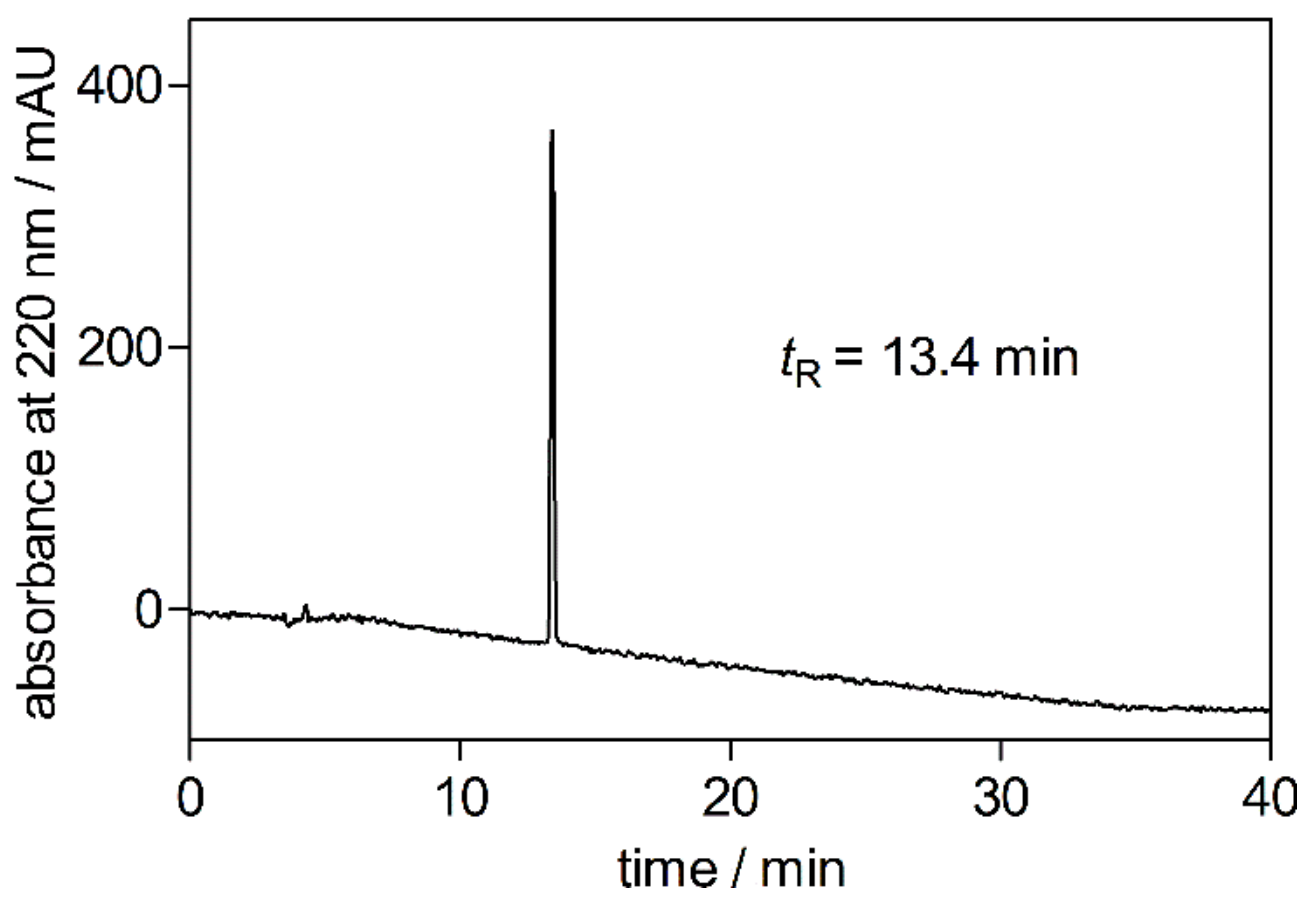


RP-HPLC analysis of compound **29**.

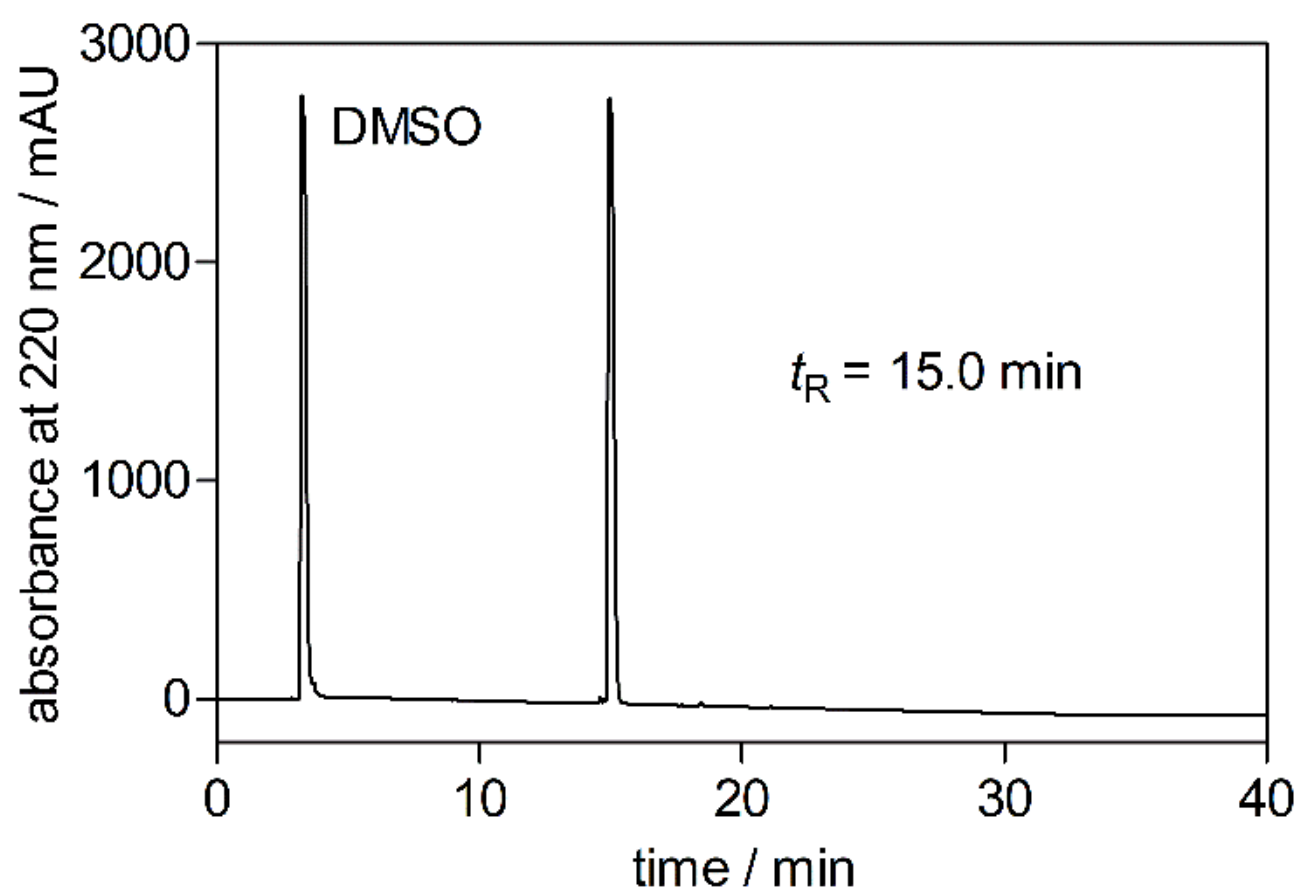


RP-HPLC analysis of compound **30**.

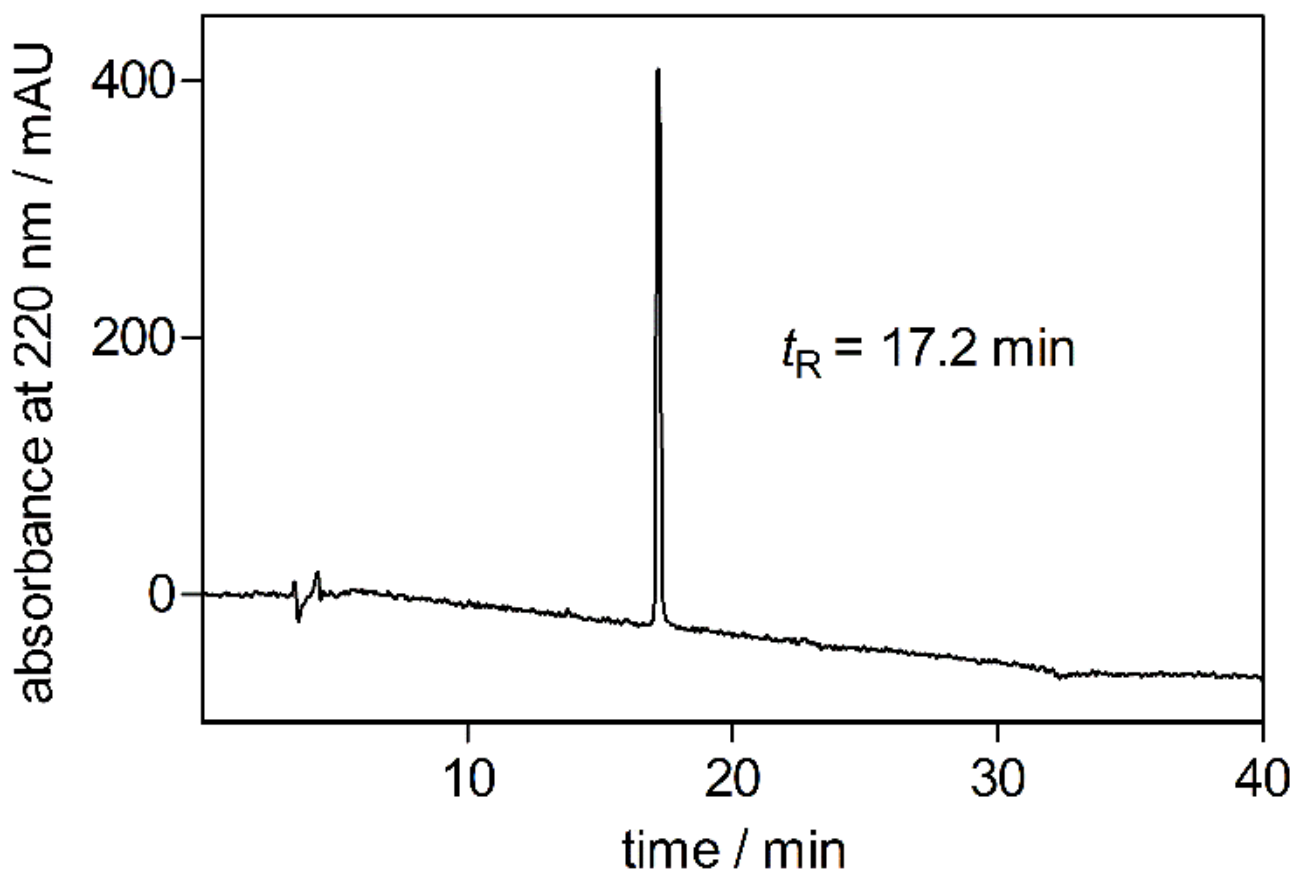


RP-HPLC analysis of compound **32**.

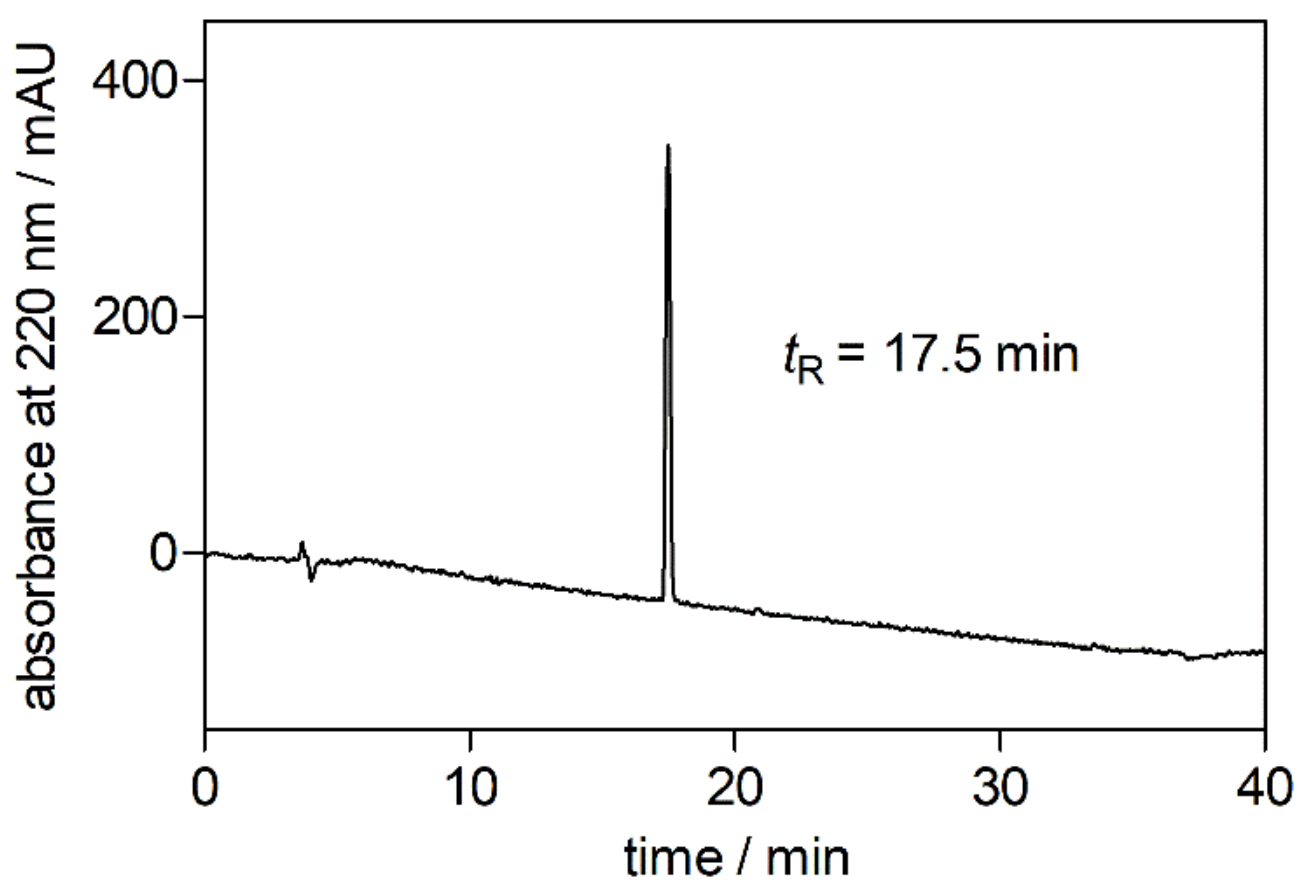


RP-HPLC analysis of compound **33**.

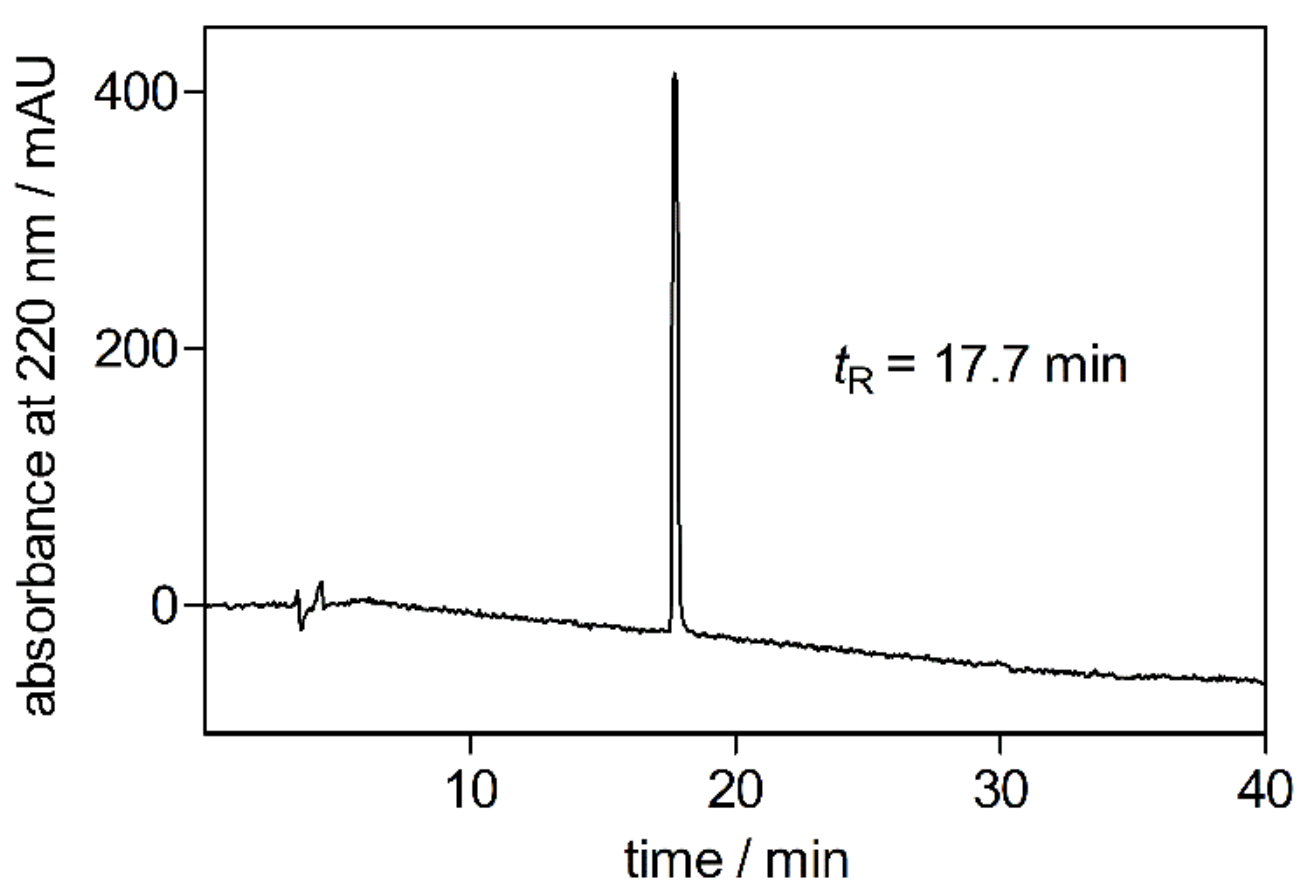


RP-HPLC analysis of compound **34**.

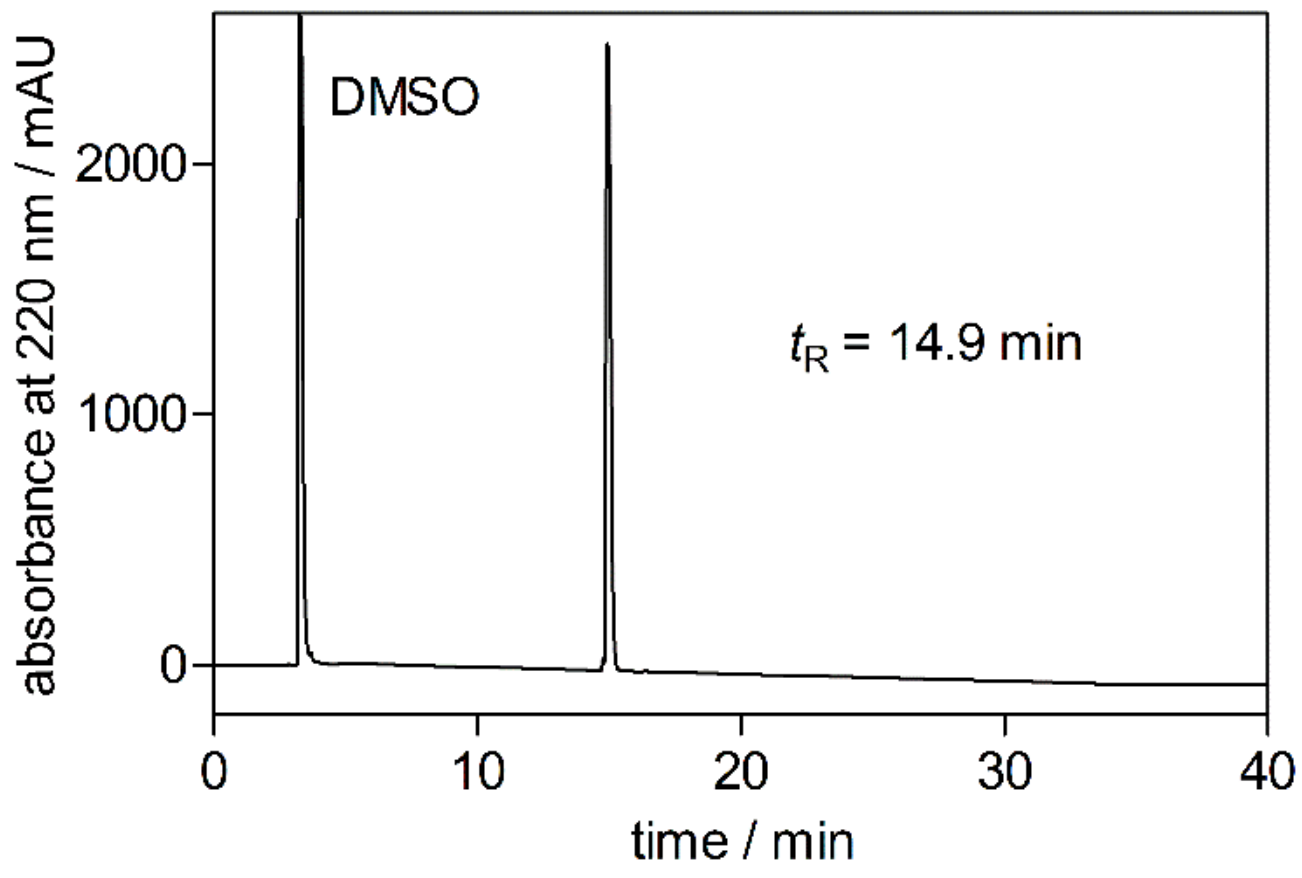


RP-HPLC analysis of compound **37**.

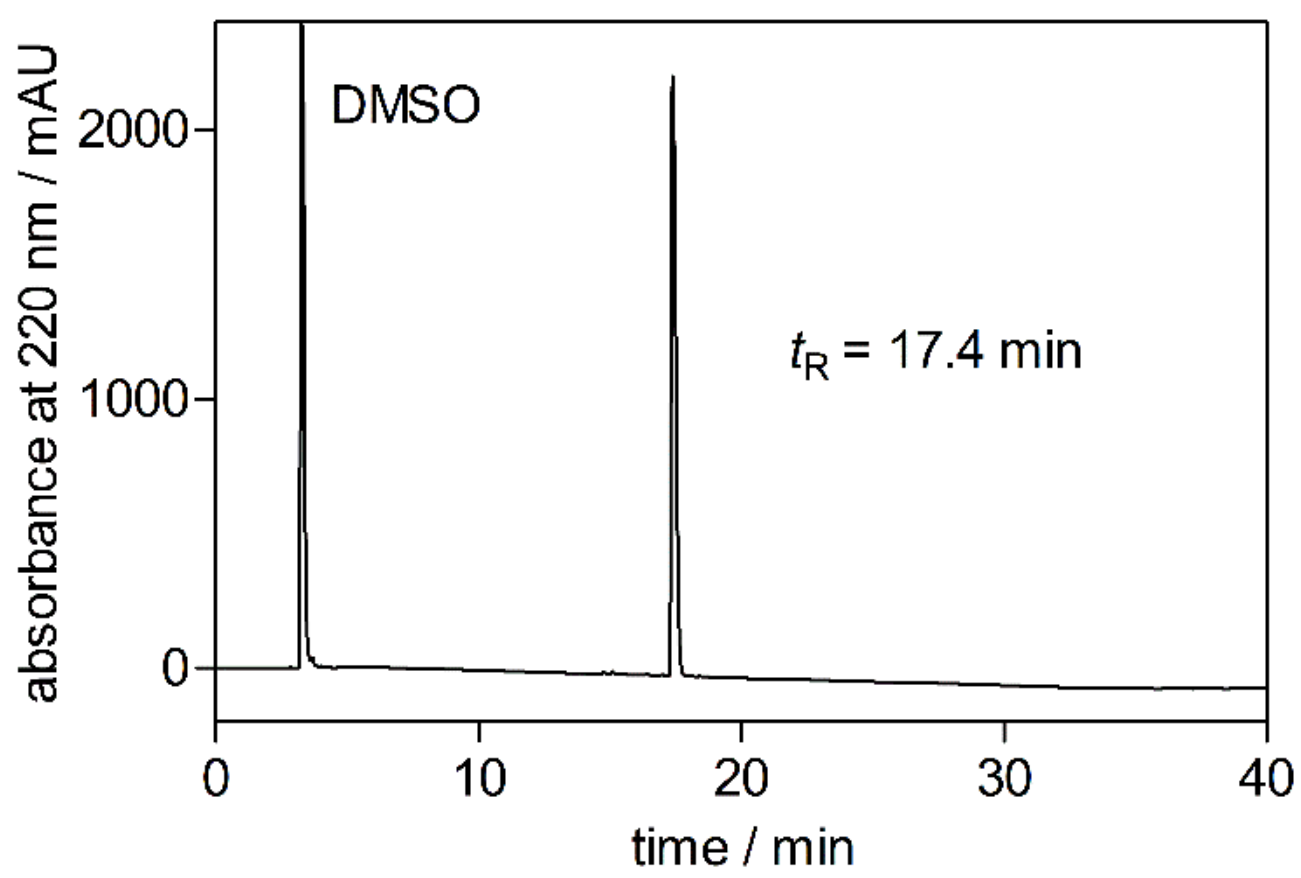


RP-HPLC analysis of compound **38**.

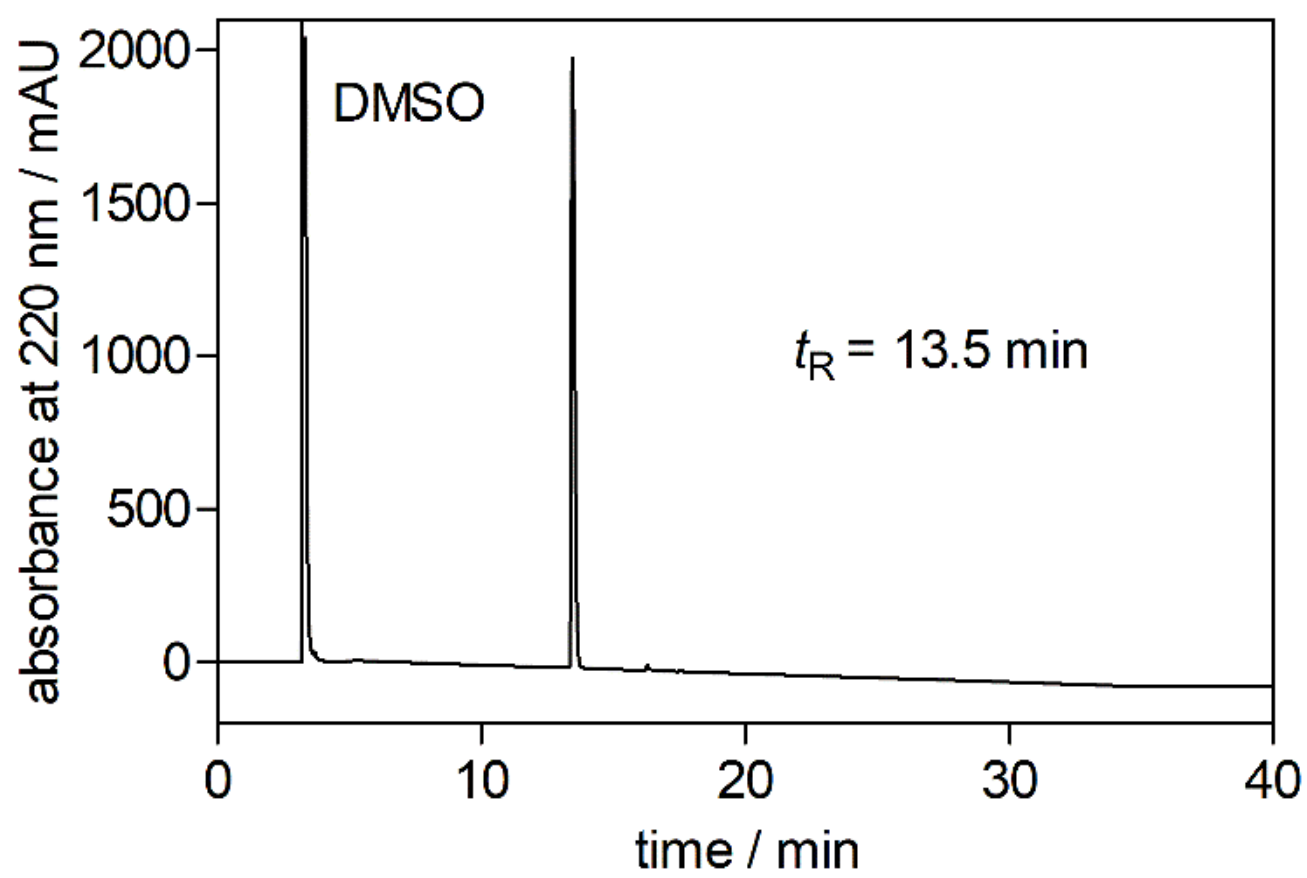


RP-HPLC analysis of compound **41**.

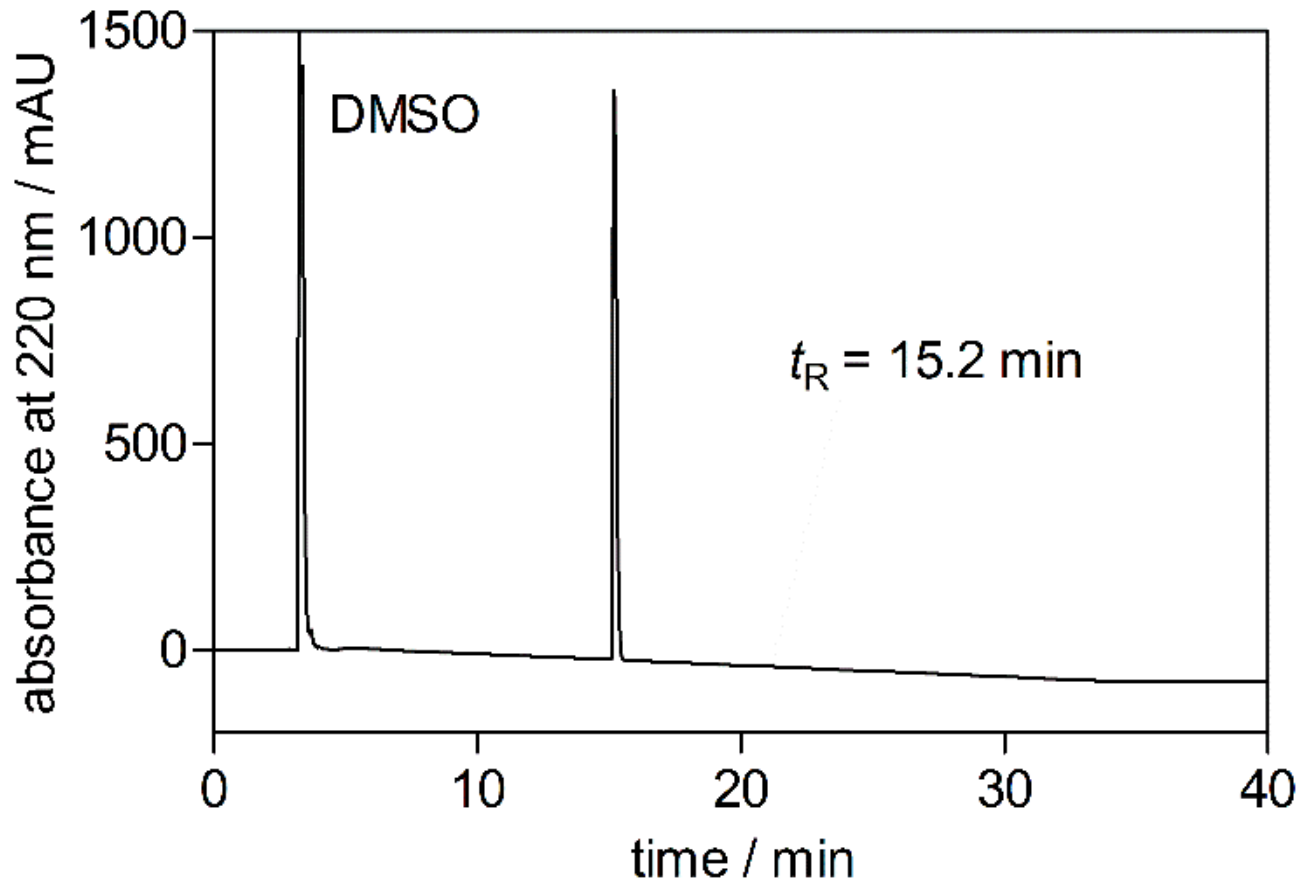


RP-HPLC analysis of compound **42**.

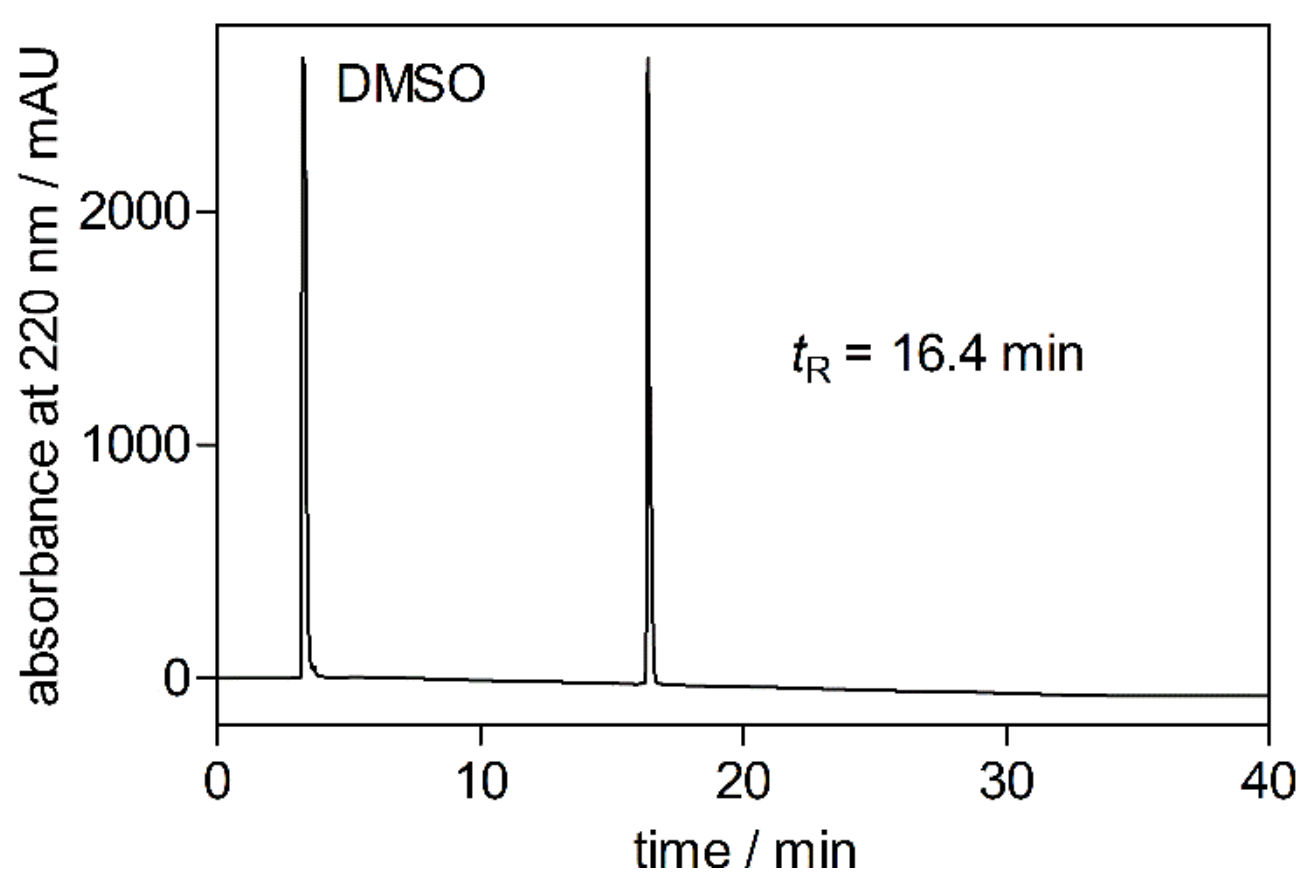


RP-HPLC analysis of compound **45**.

**3. $^{1}$H- and $^{13}$C-NMR spectra of compounds 3, 26-30, 32-34, 37, 38, 41, 42, and 45, and HSQC and HMBC spectrum of 42**

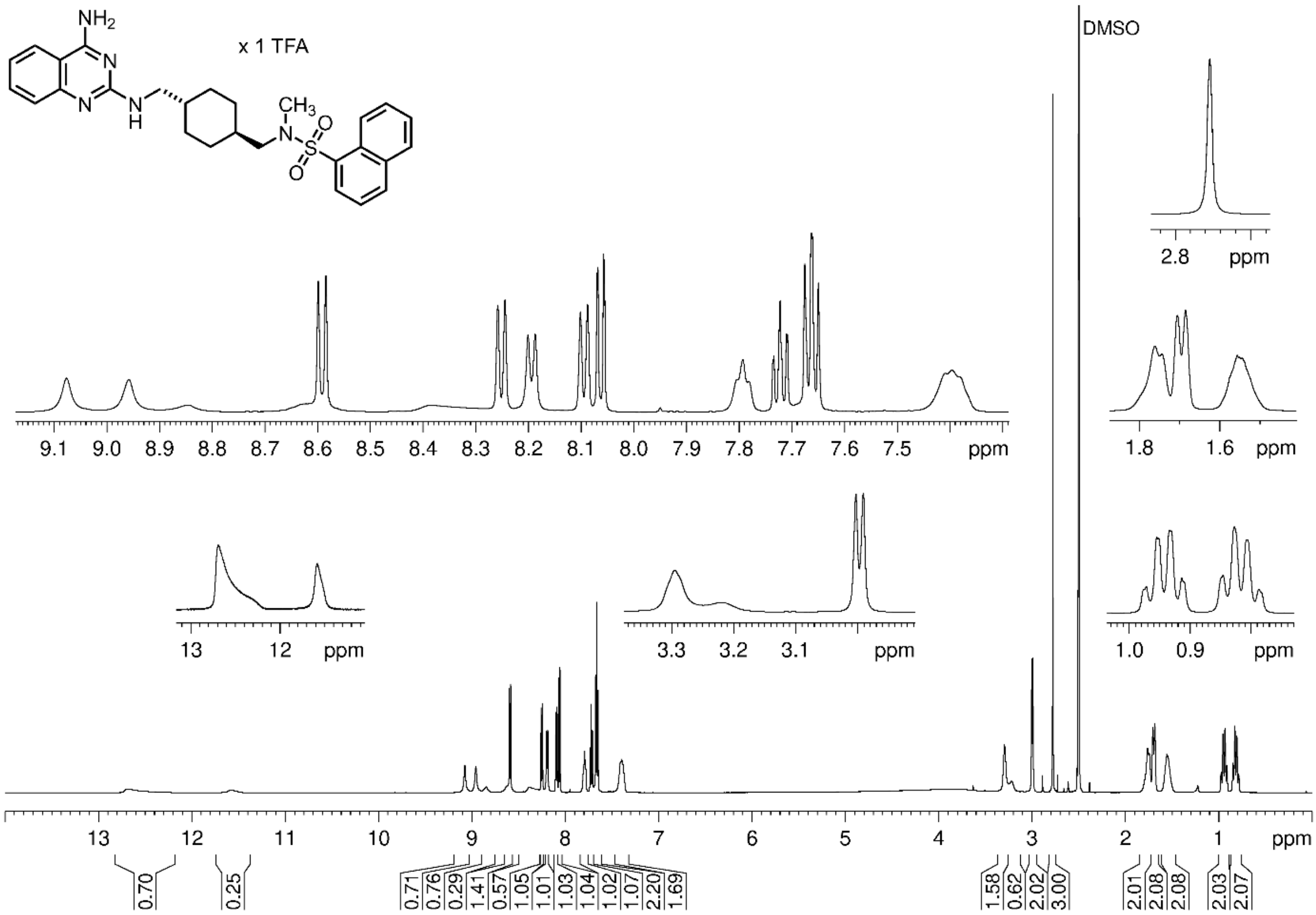


$^{1}$H-NMR spectrum (600 MHz, DMSO-$d_6$) of compound **3**

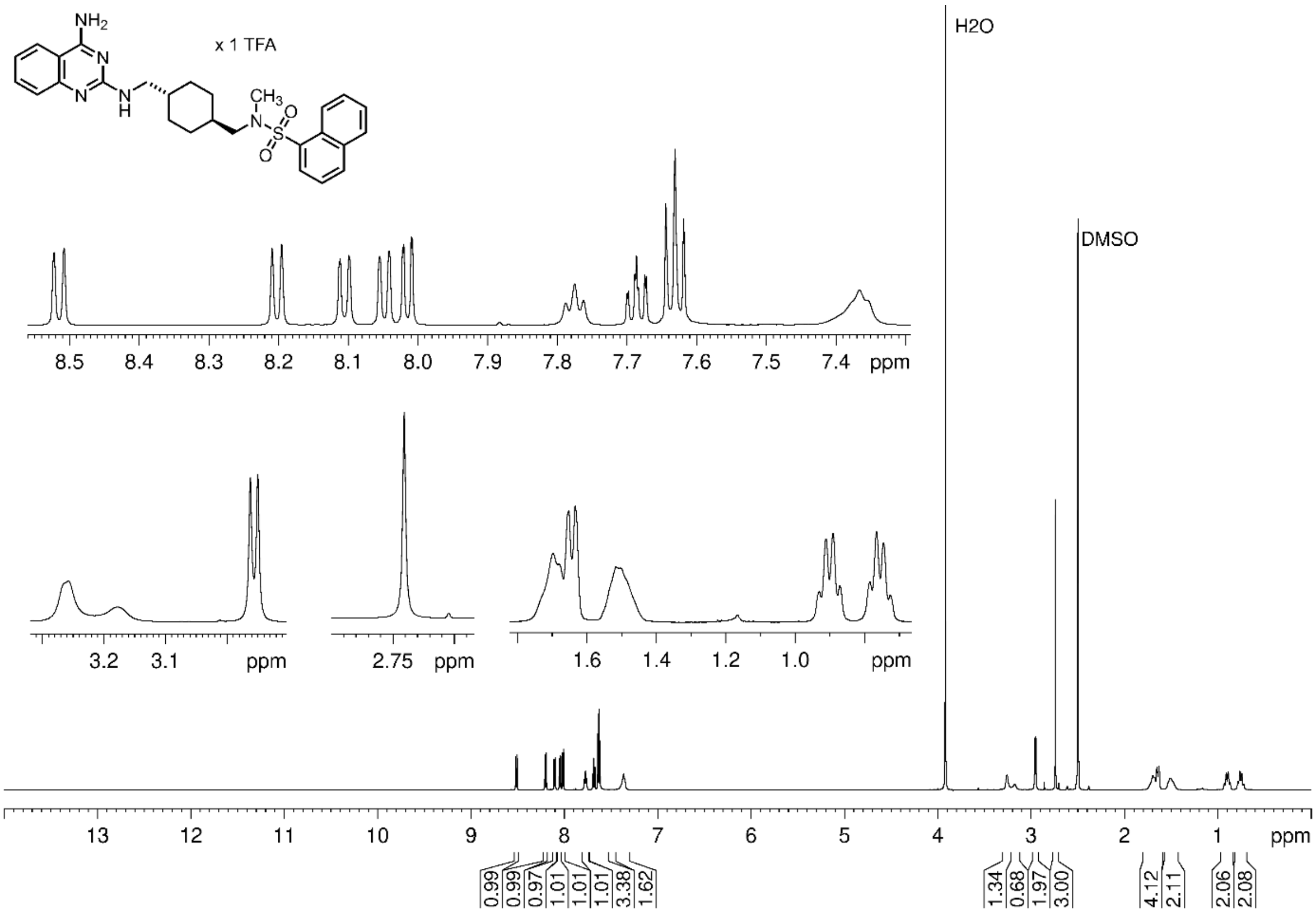


$^{1}$H-NMR spectrum (600 MHz, DMSO-$d_6$/$D_2O$ 9:1 v/v) of compound **3**

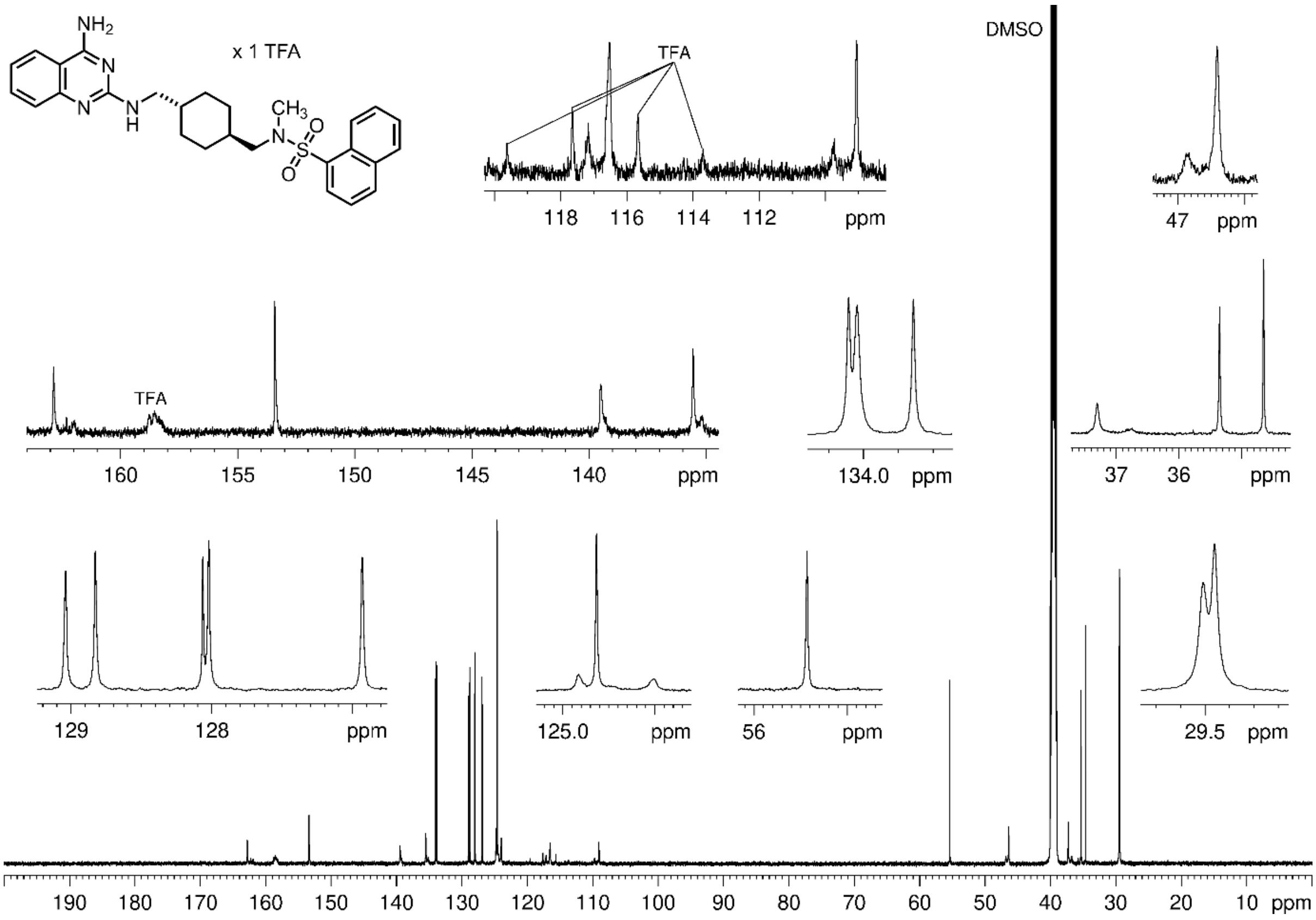


$^{13}$C-NMR spectrum (150 MHz, DMSO-*d*6) of compound **3**

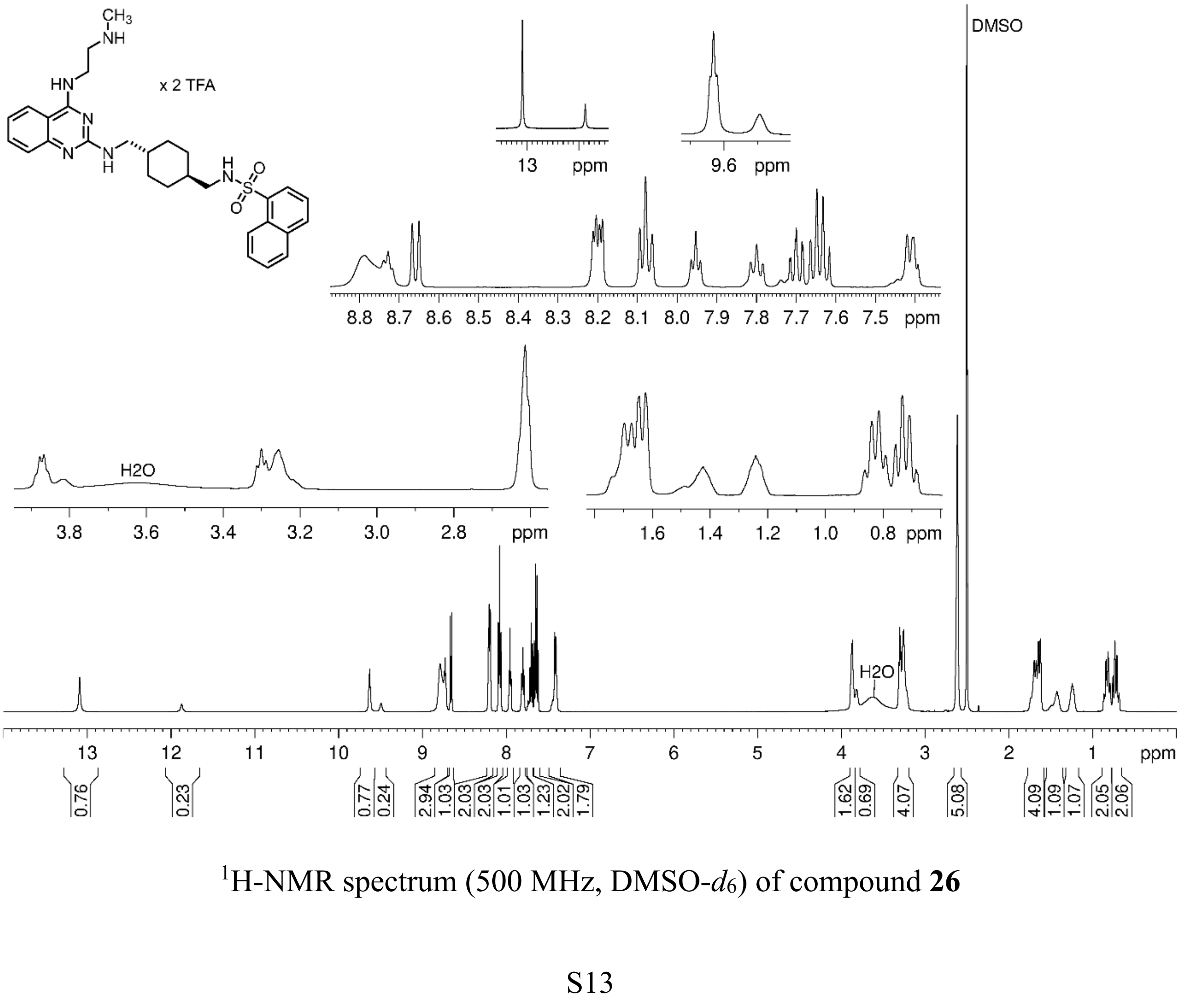


$^{1}$H-NMR spectrum (500 MHz, DMSO-*d*6) of compound **26**

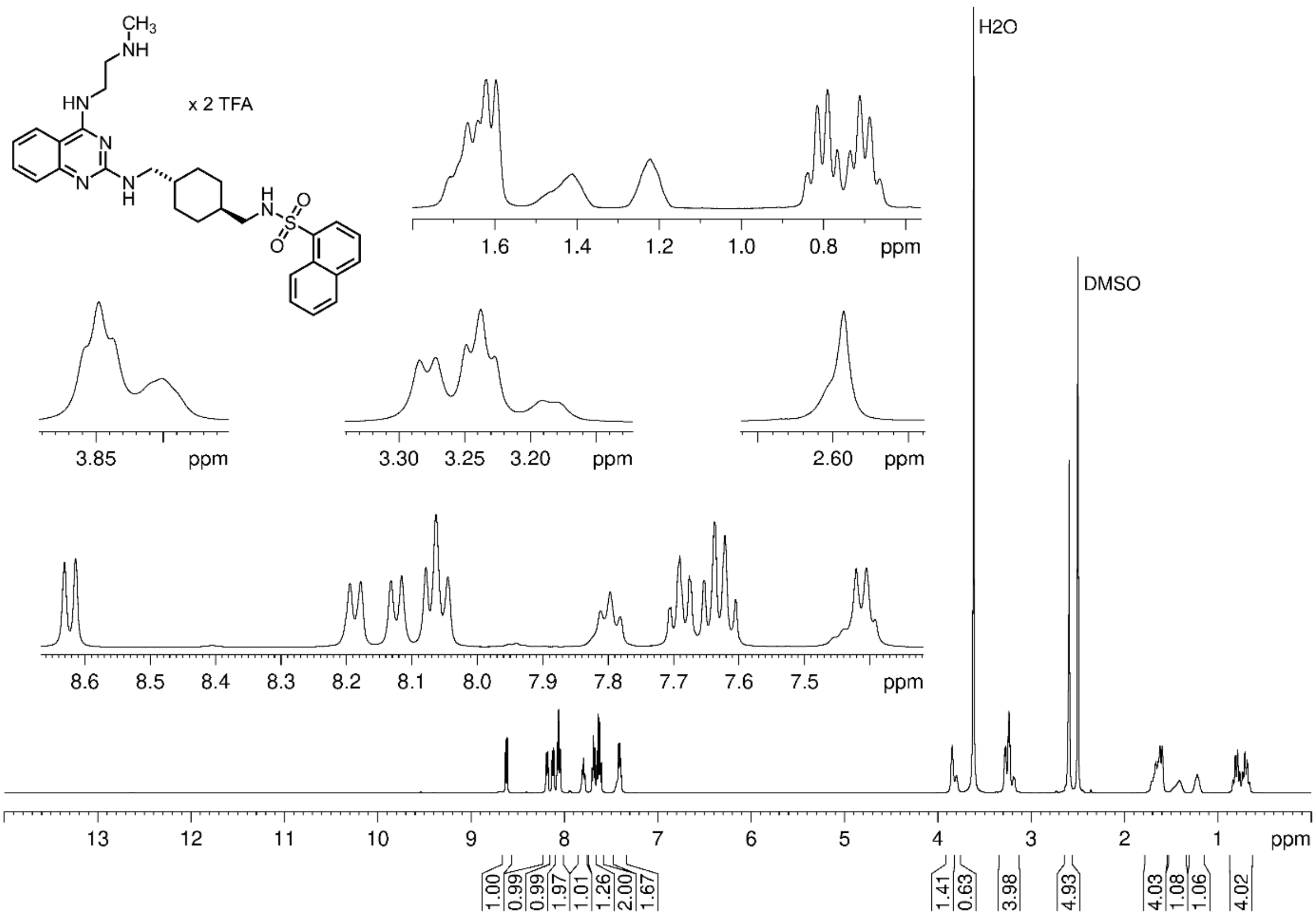


$^{1}$H-NMR spectrum (500 MHz, DMSO-$d_6$/$D_2O$ 9:1 v/v) of compound **26**

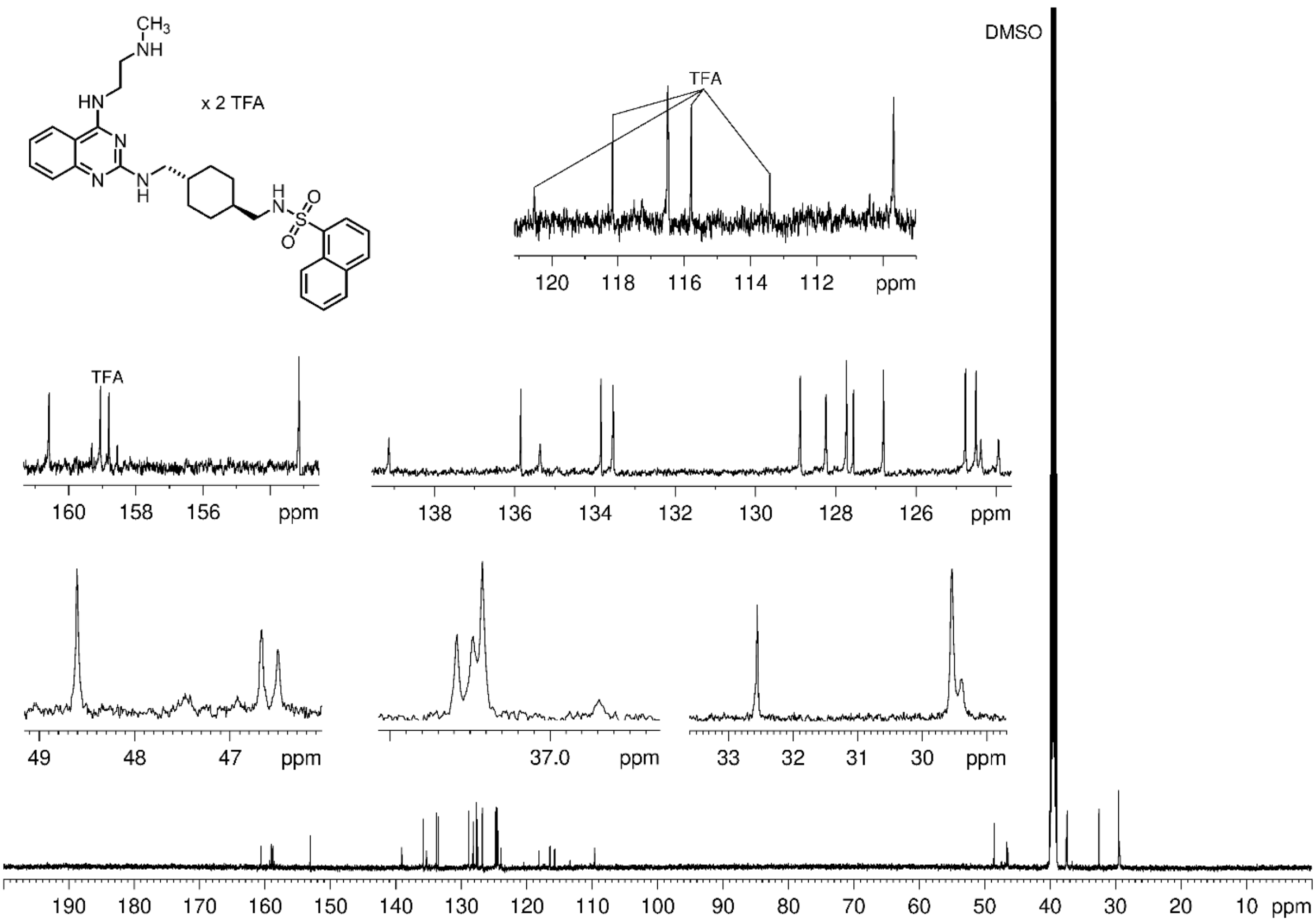


$^{13}$C-NMR spectrum (126 MHz, DMSO-$d_6$) of compound **26**

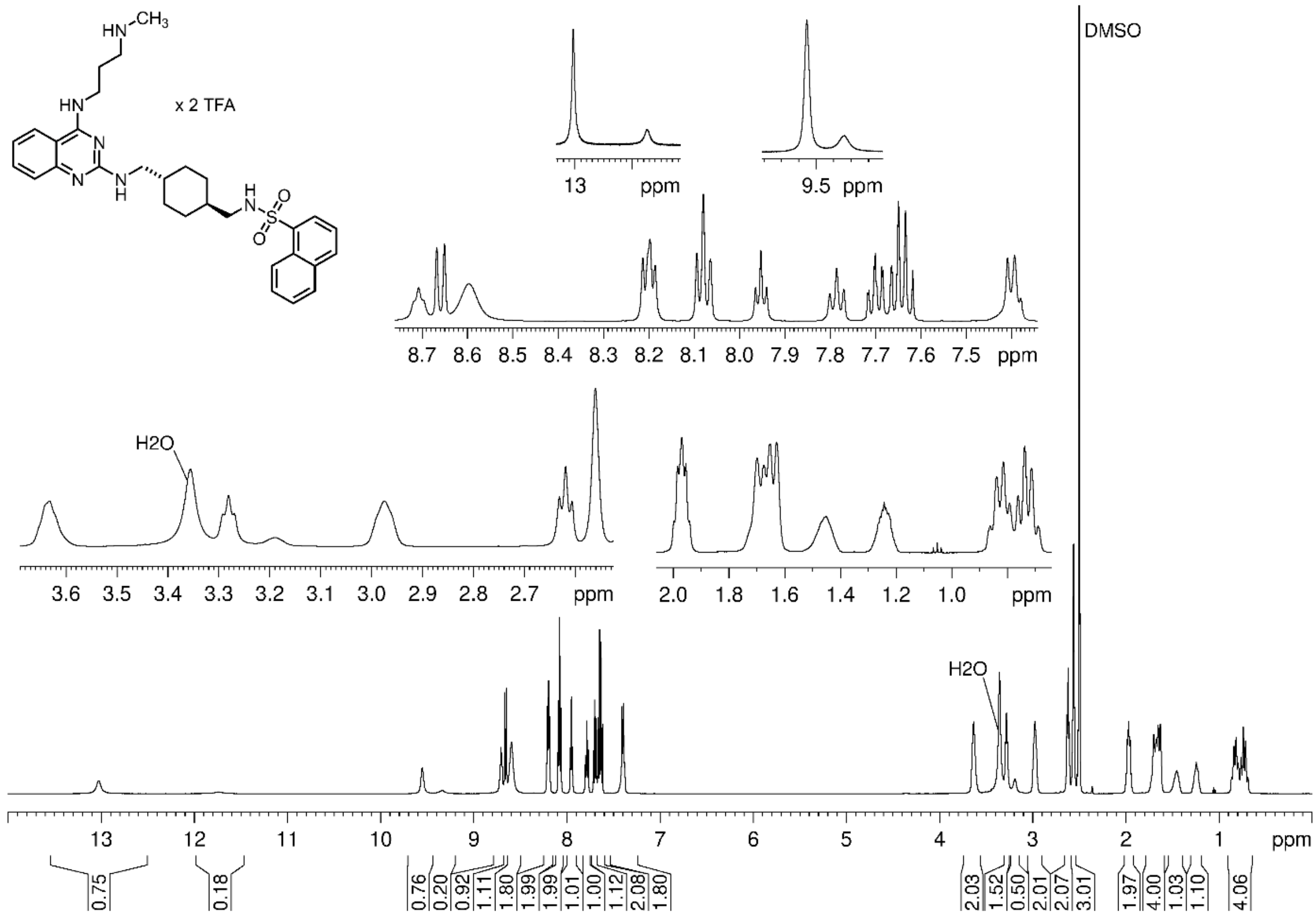


$^{1}$H-NMR spectrum (500 MHz, DMSO-$d_6$) of compound **27**

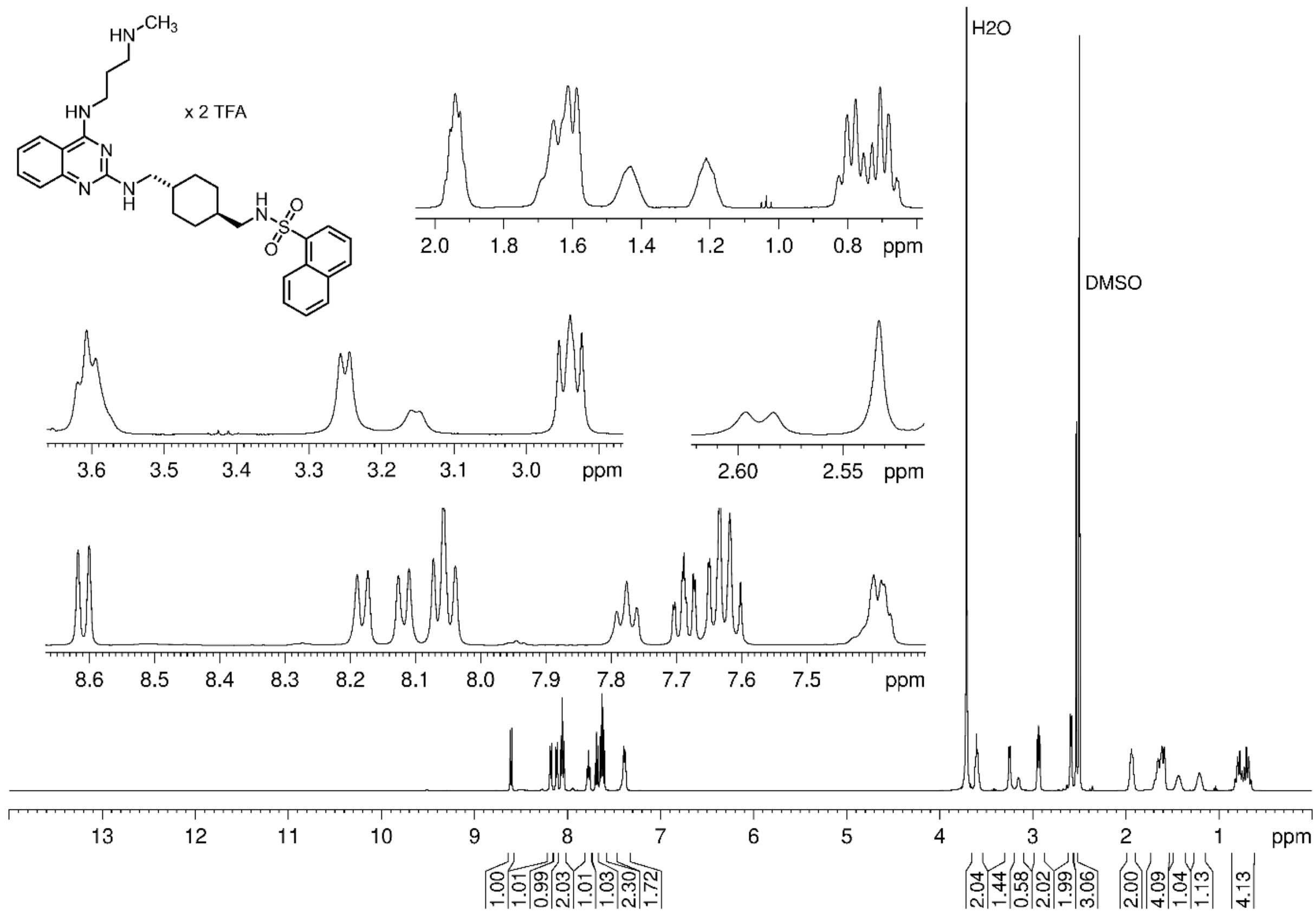


$^{1}$H-NMR spectrum (500 MHz, DMSO-$d_6$/$D_2O$ 9:1 v/v) of compound **27**

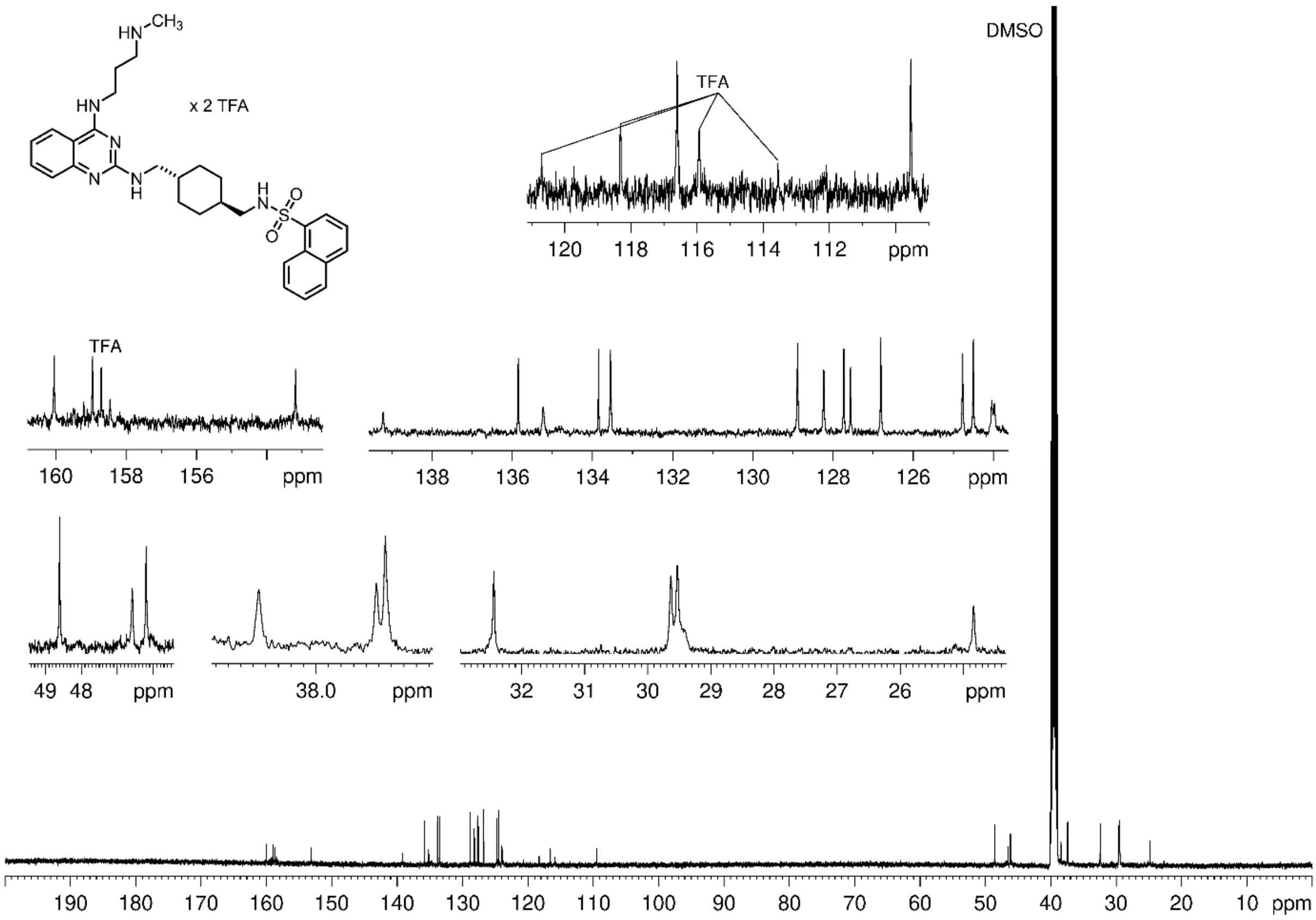


$^{13}$C-NMR spectrum (126 MHz, DMSO-$d_6$) of compound **27**

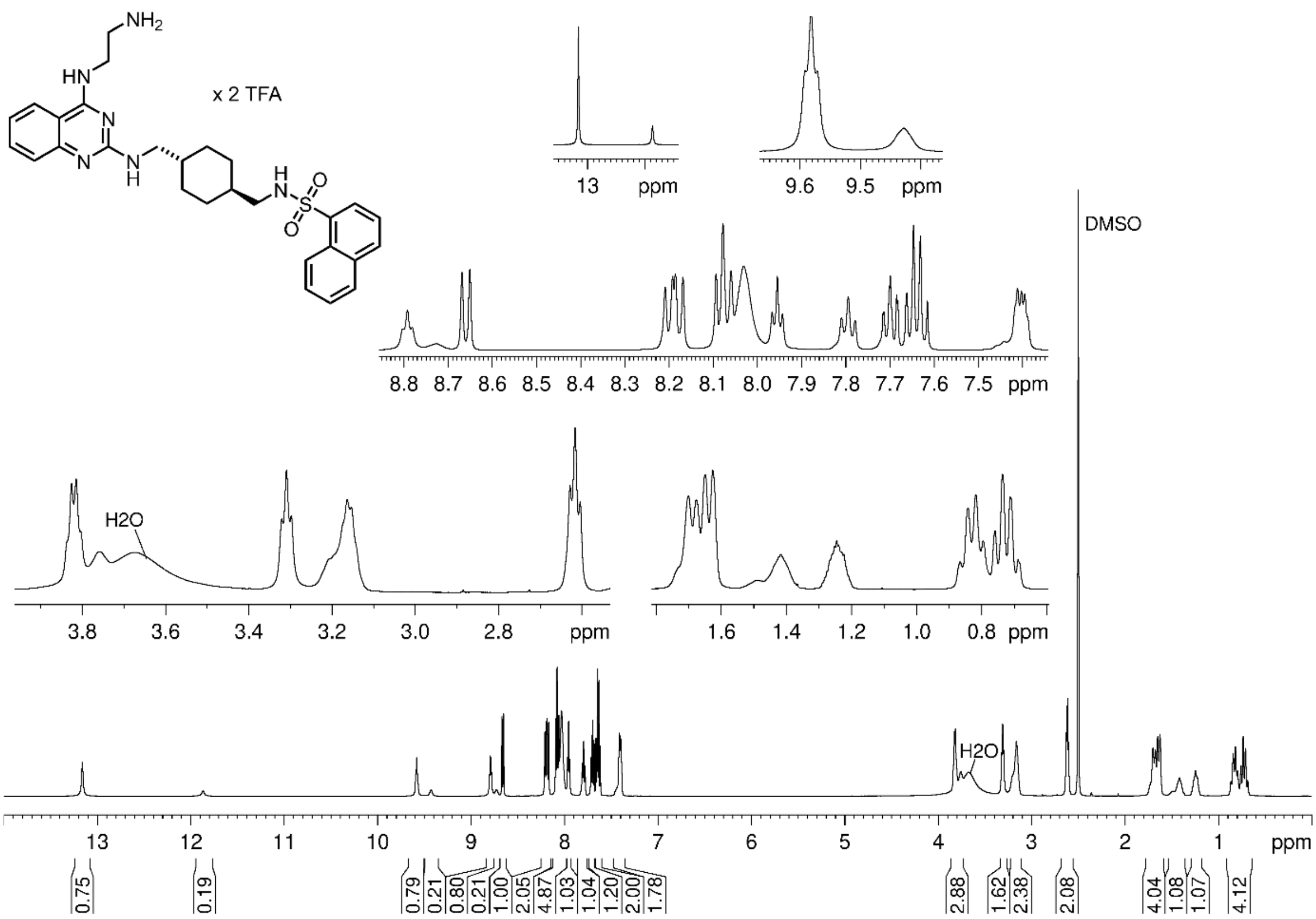


$^{1}$H-NMR spectrum (500 MHz, DMSO-$d_6$) of compound **28**

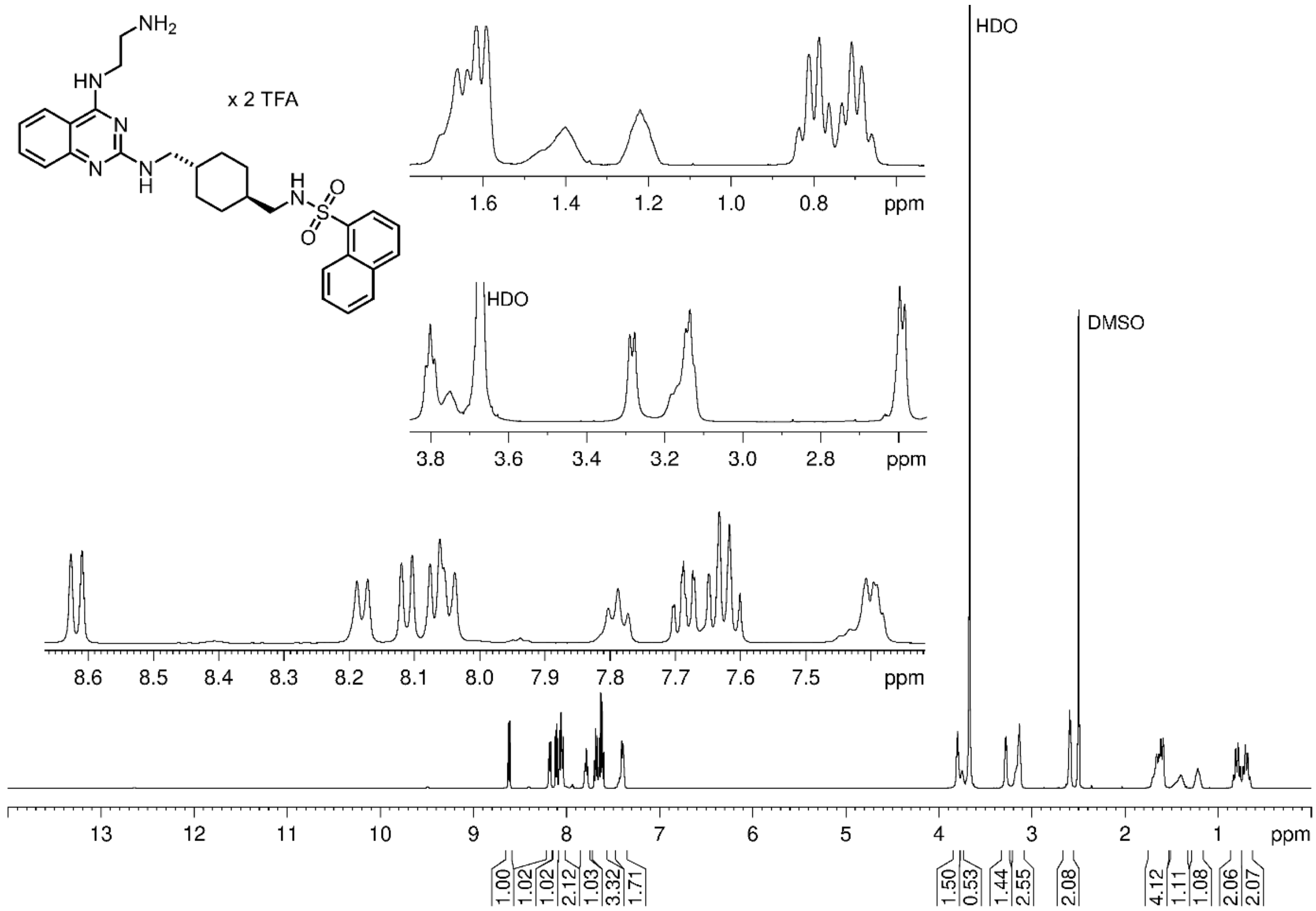


$^{1}$H-NMR spectrum (500 MHz, DMSO-$d_6$/$D_2O$ 9:1 v/v) of compound **28**

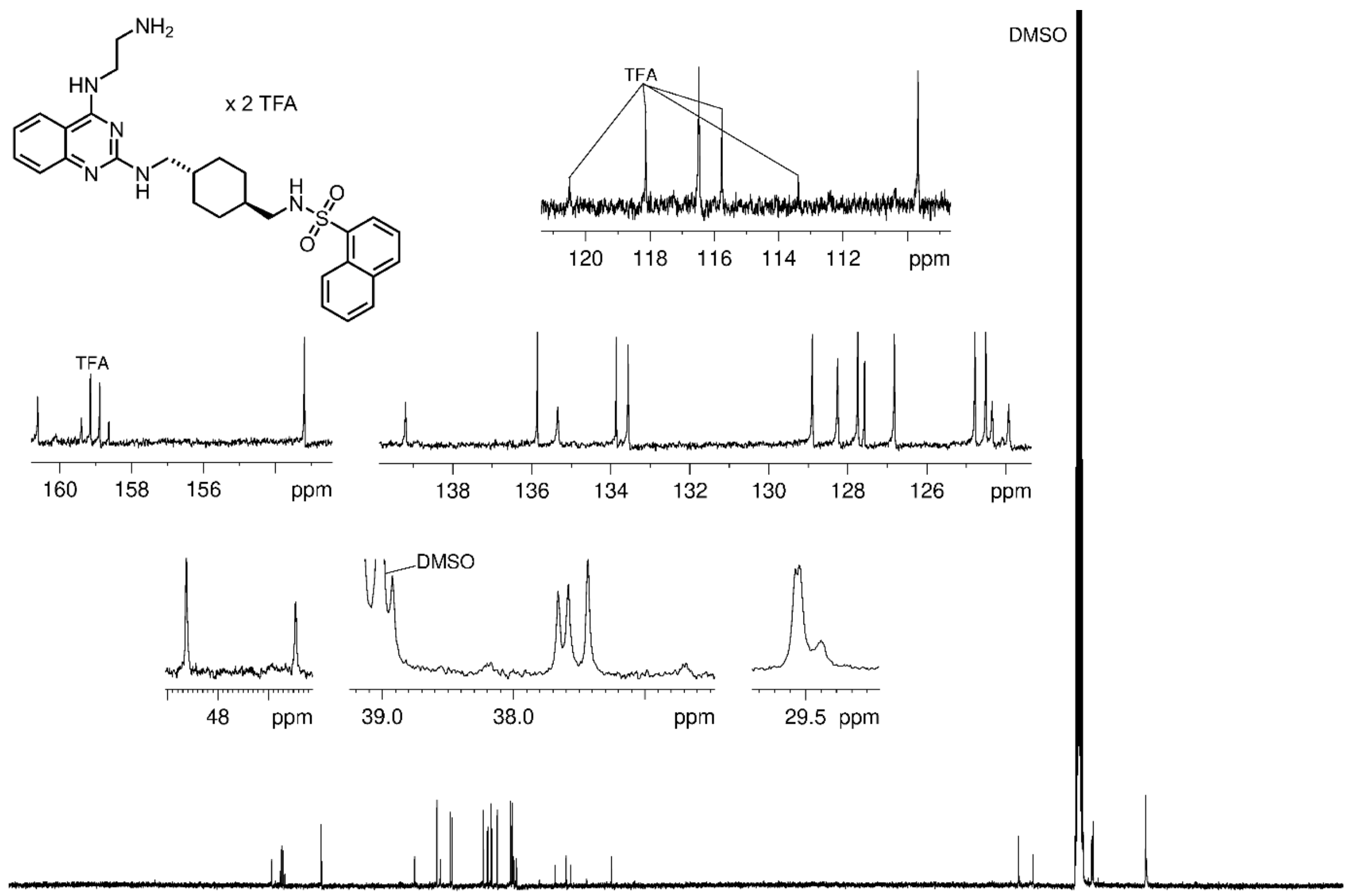


$^{13}$C-NMR spectrum (126 MHz, DMSO-$d_6$) of compound **28**

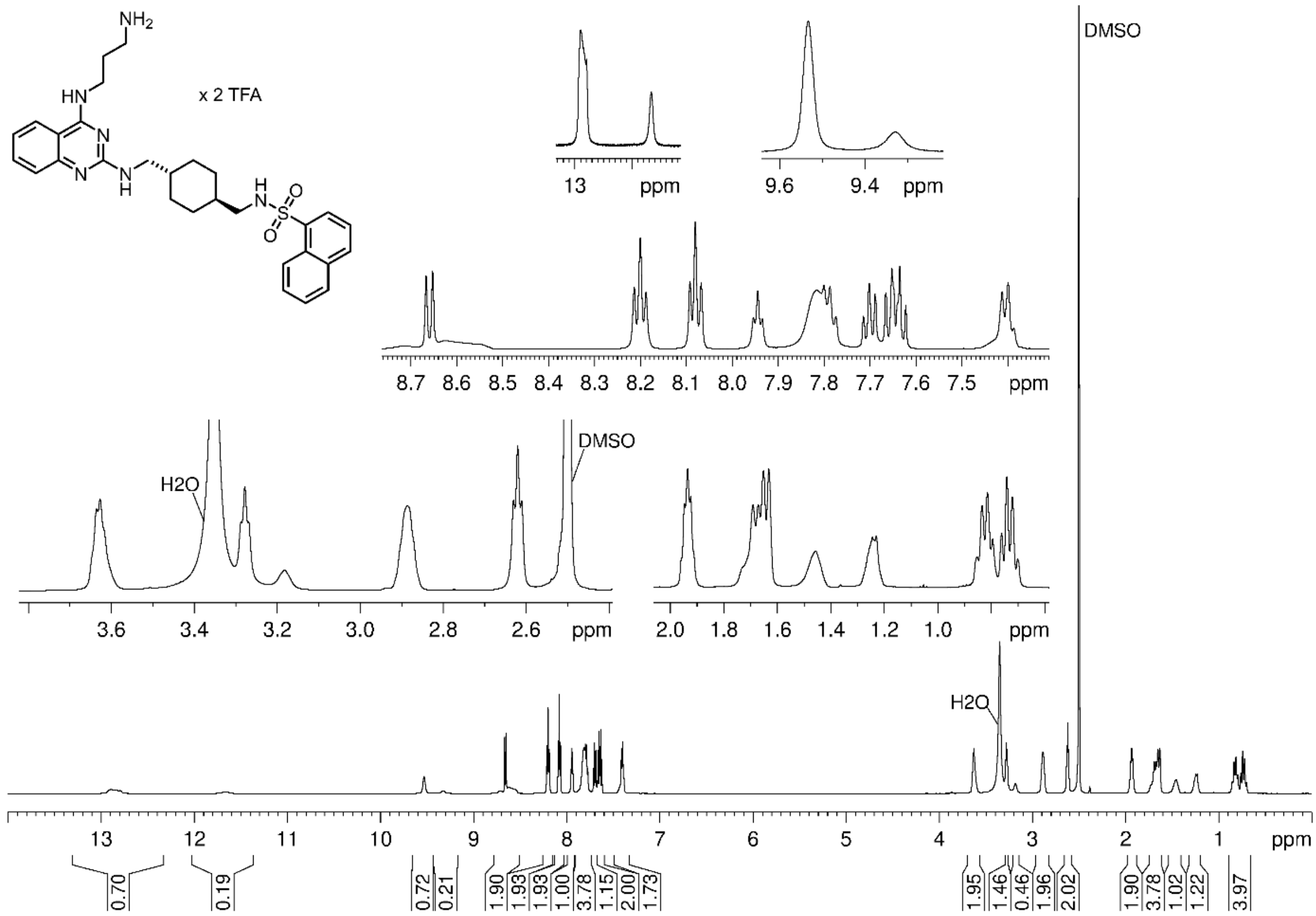


$^{1}$H-NMR spectrum (600 MHz, DMSO-$d_6$) of compound **29**

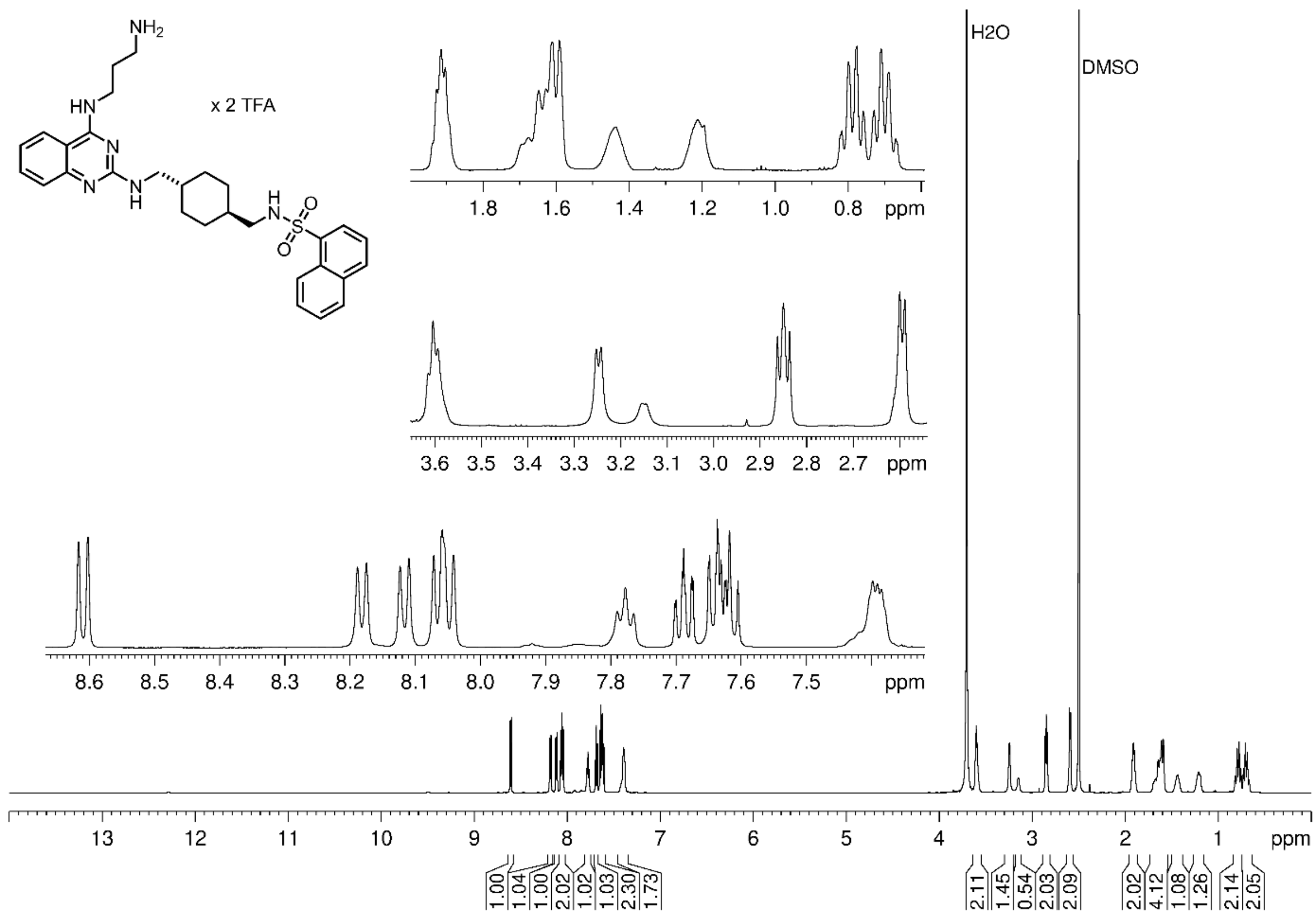


$^{1}$H-NMR spectrum (600 MHz, DMSO-$d_6$/$D_2O$ 9:1 v/v) of compound **29**

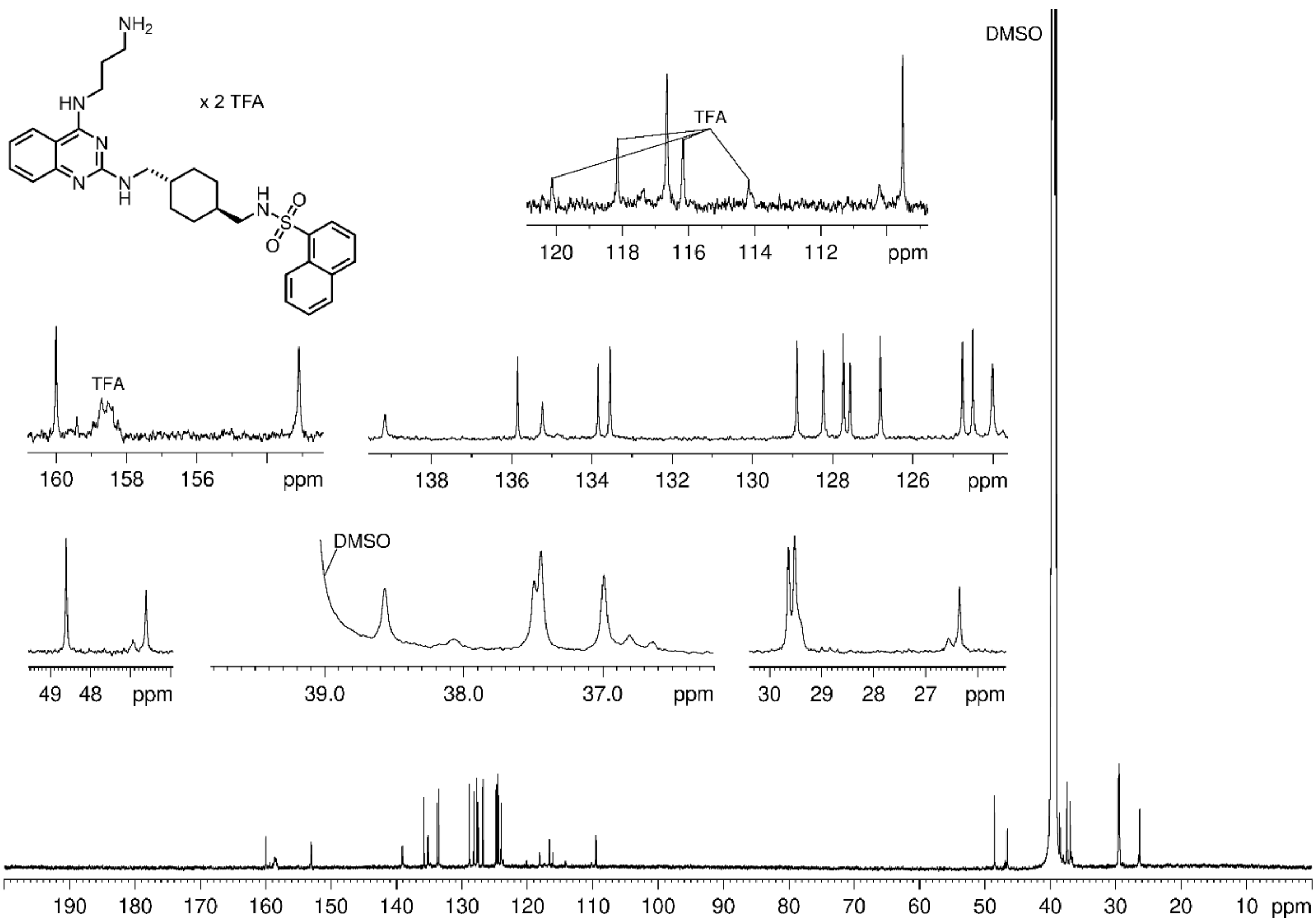


$^{13}$C-NMR spectrum (150 MHz, DMSO-$d_6$) of compound **29**

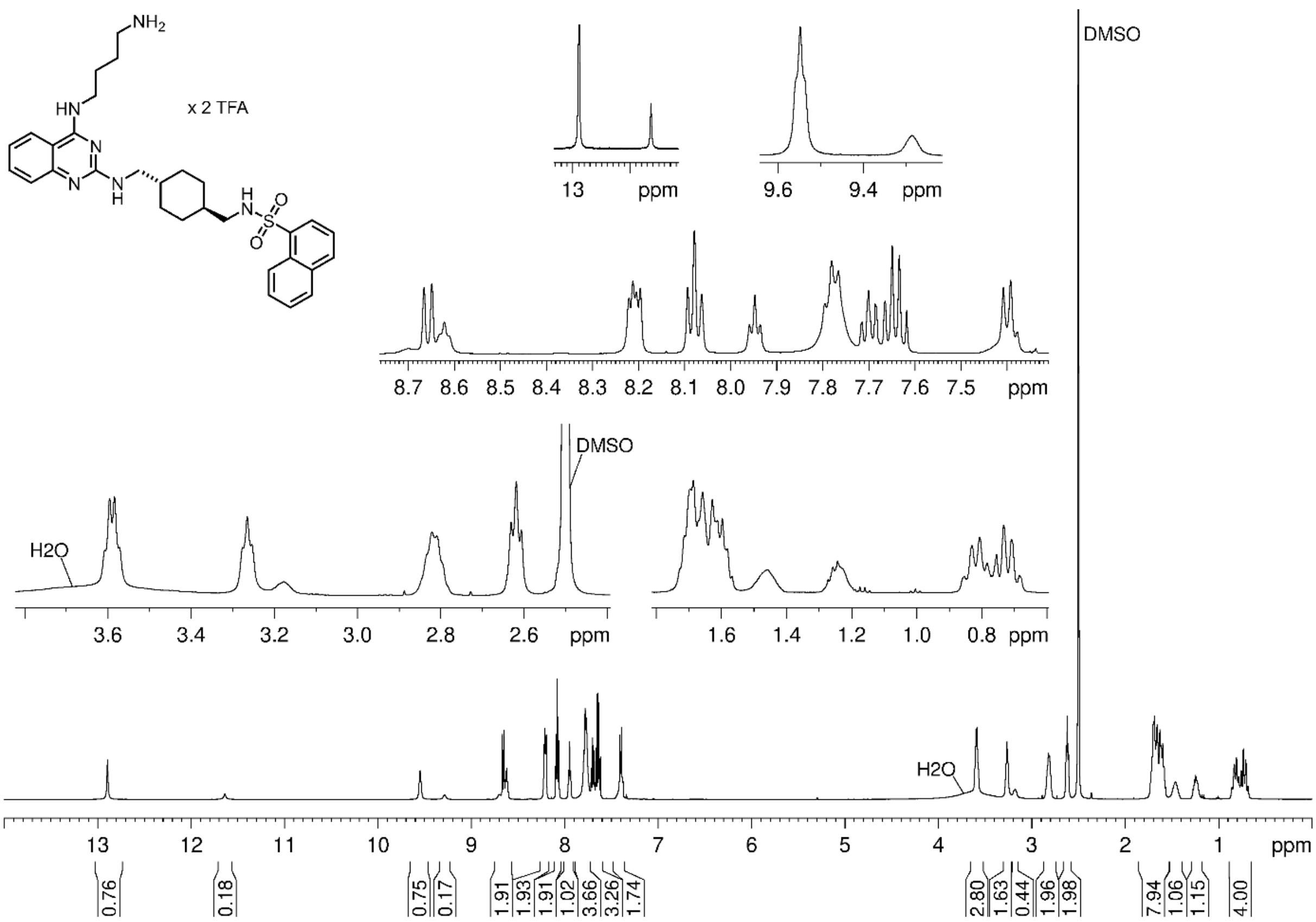


$^{1}$H-NMR spectrum (500 MHz, DMSO-$d_6$) of compound **30**

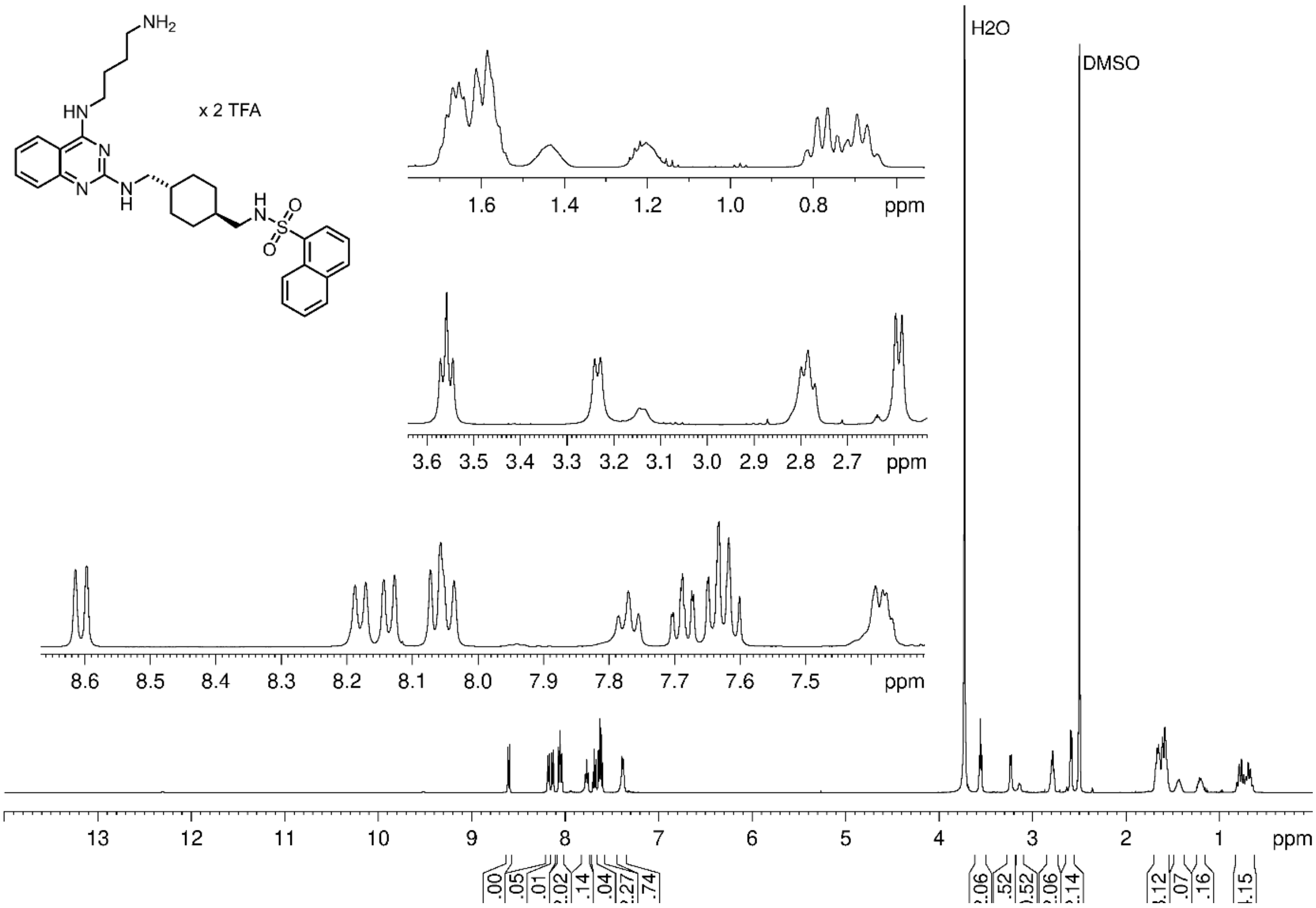

$^{1}$H-NMR spectrum (500 MHz, DMSO-$d_6$/$D_2O$ 9:1 v/v) of compound **30**

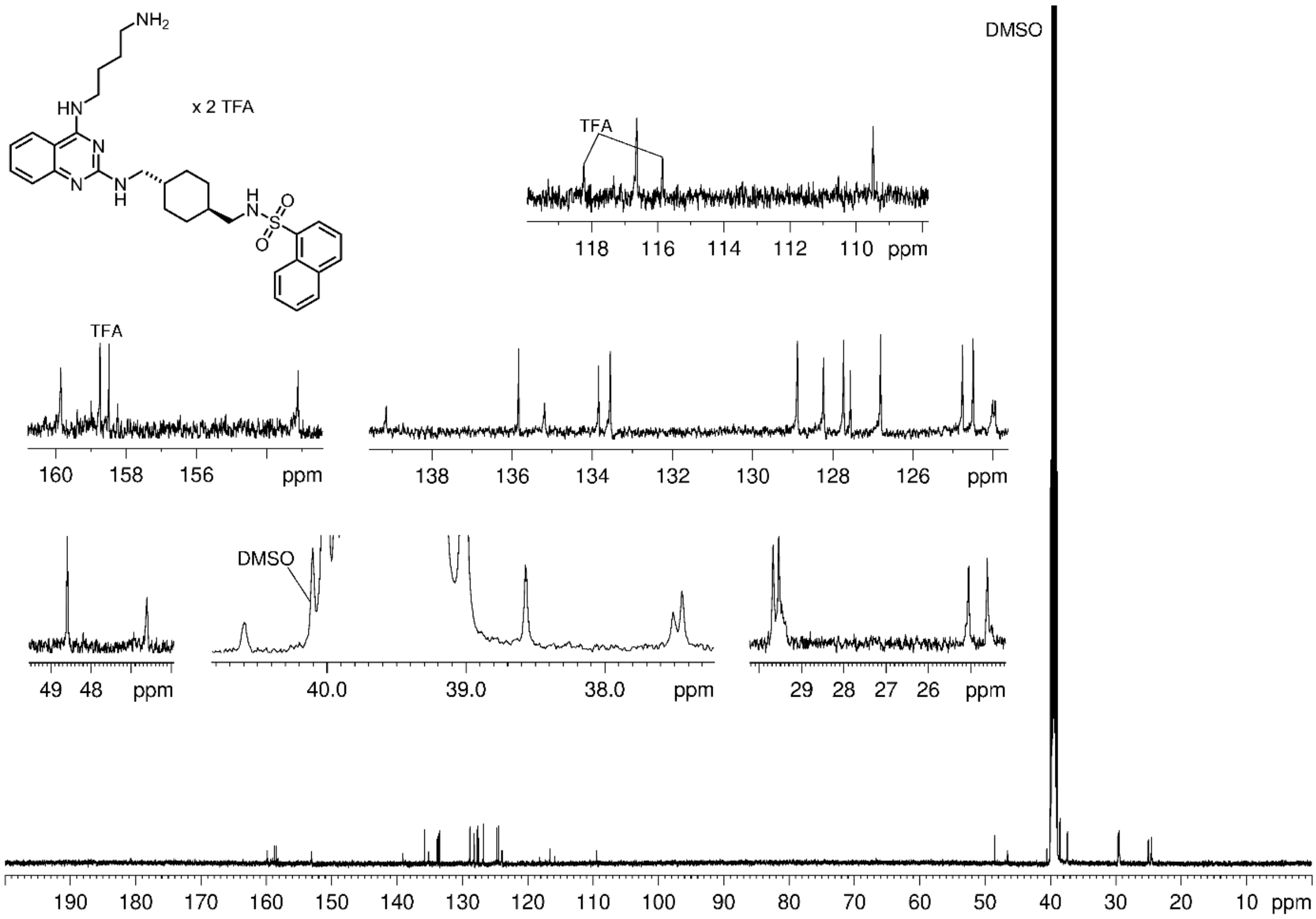

$^{13}$C-NMR spectrum (126 MHz, DMSO-$d_6$) of compound **30**

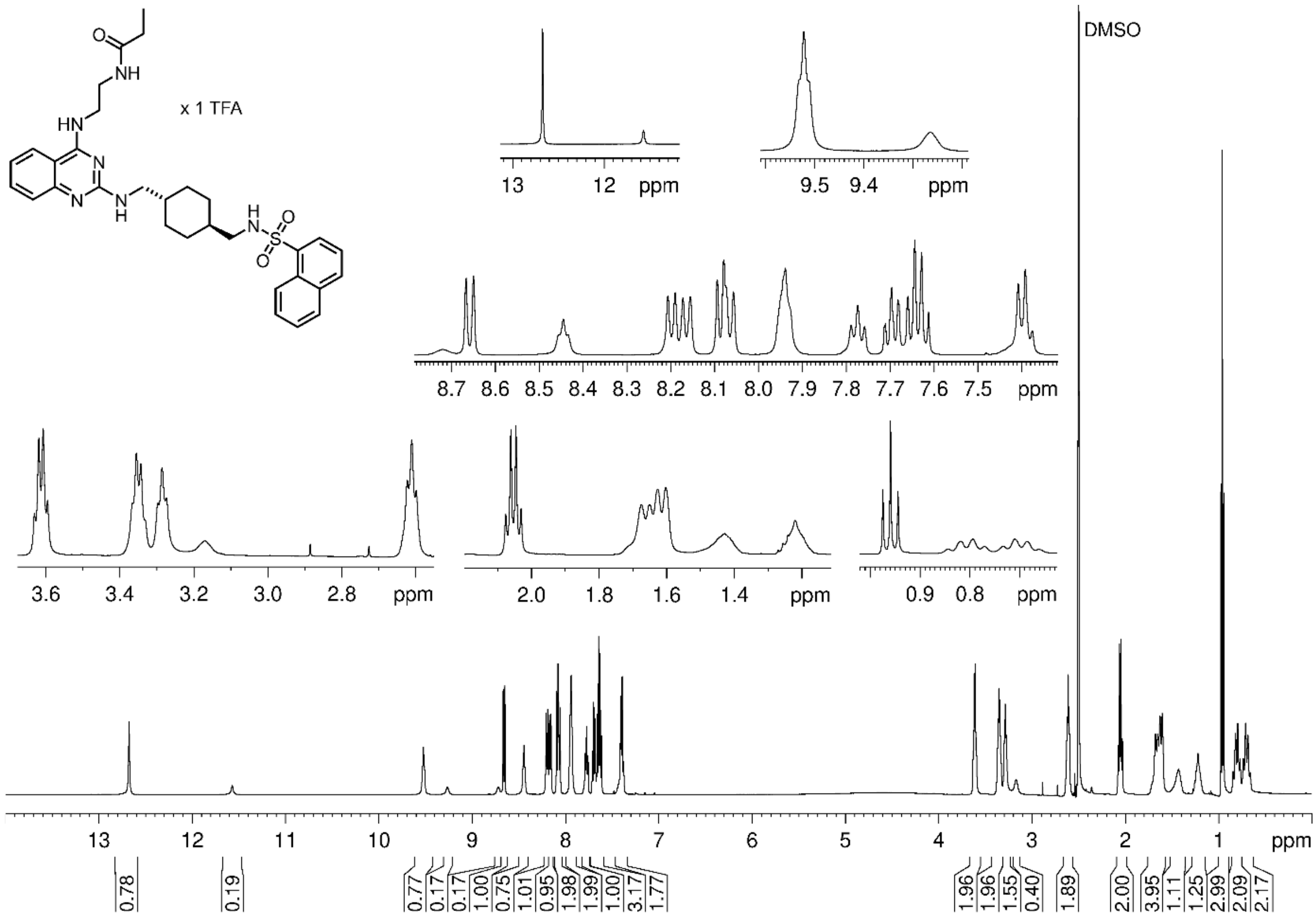


$^1$H-NMR spectrum (500 MHz, DMSO-$d_6$) of compound **32**

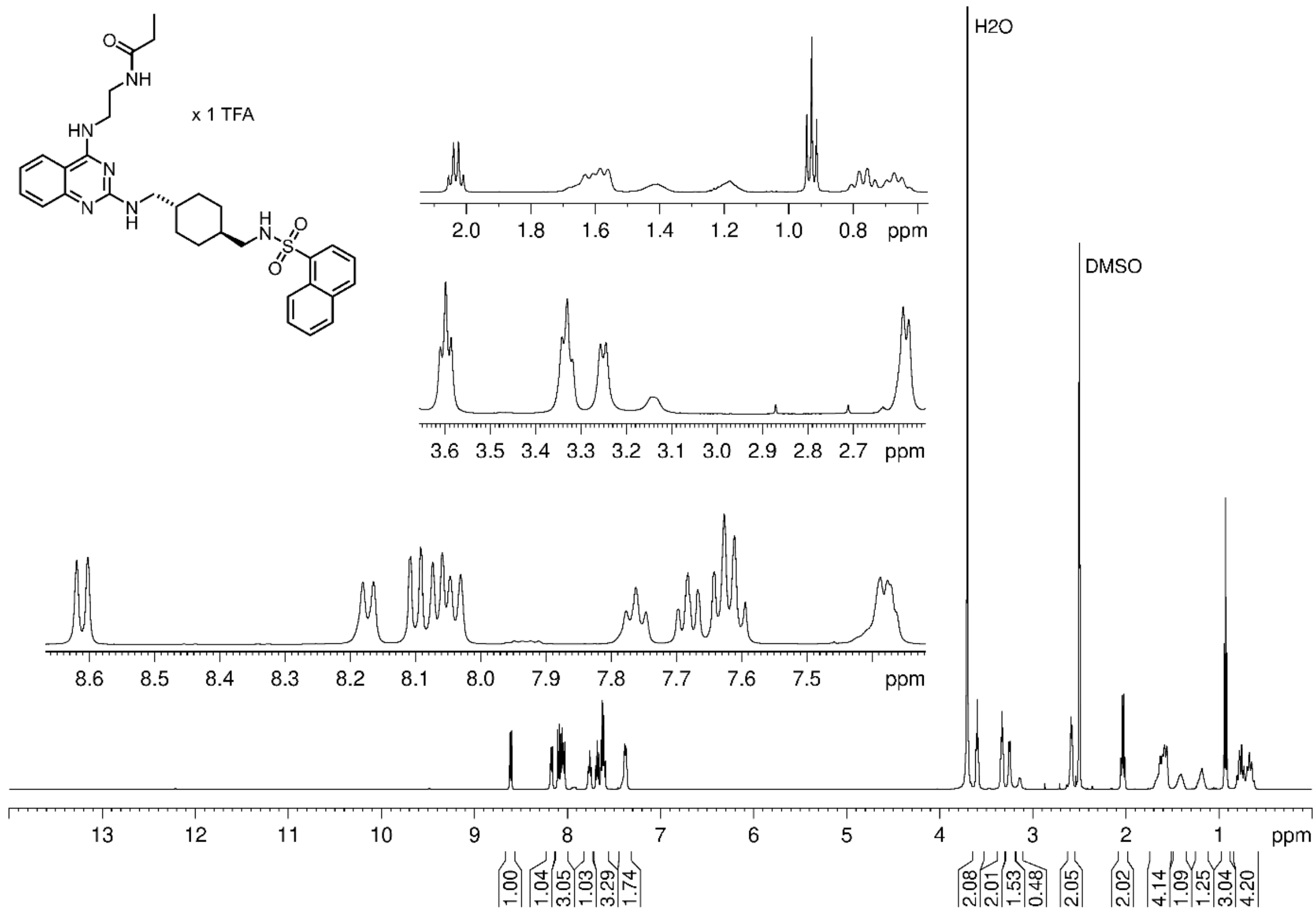


$^1$H-NMR spectrum (500 MHz, DMSO-$d_6$/$D_2O$ 9:1 v/v) of compound **32**

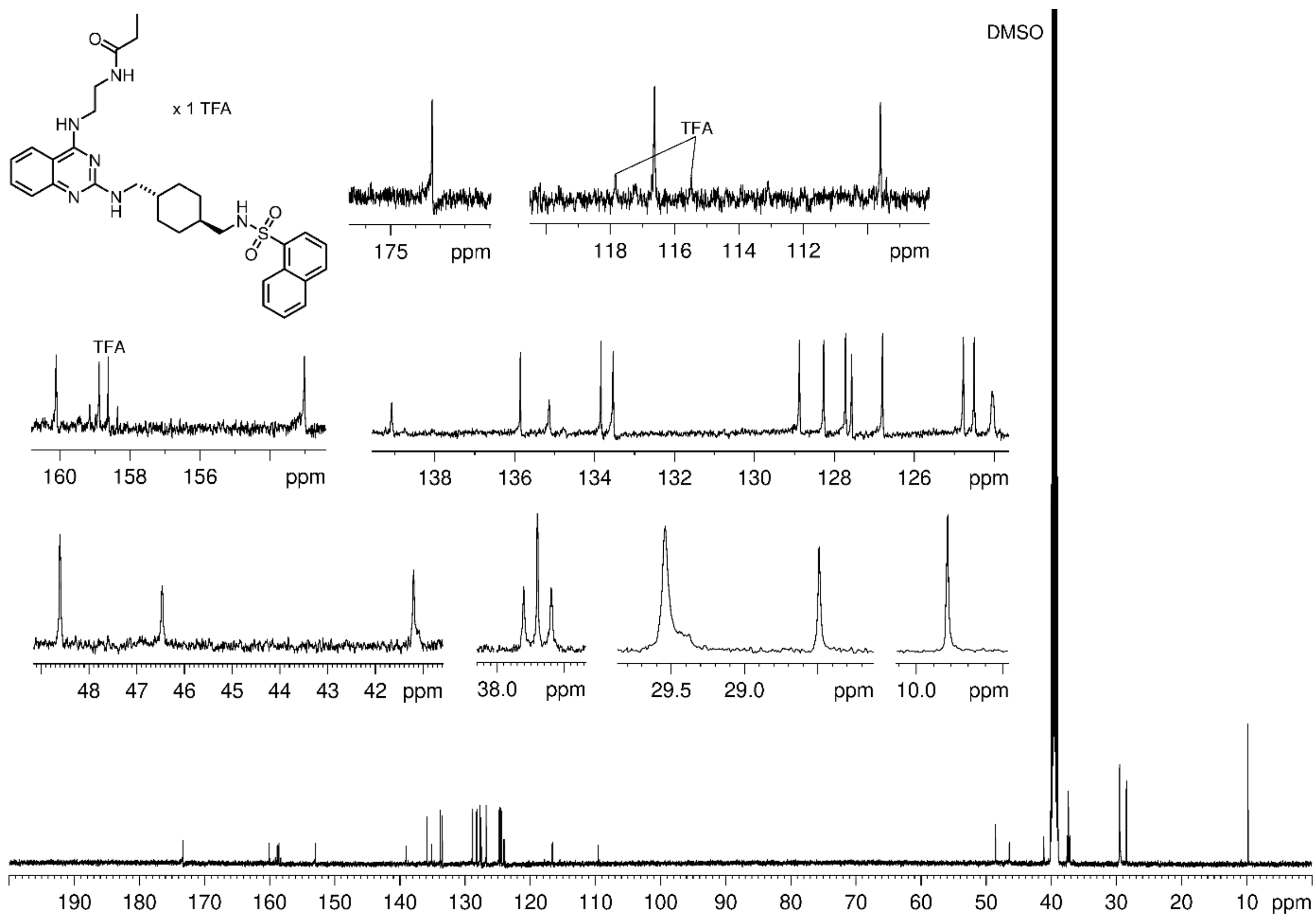

$^{13}$C-NMR spectrum (126 MHz, DMSO-$d_6$) of compound **32**

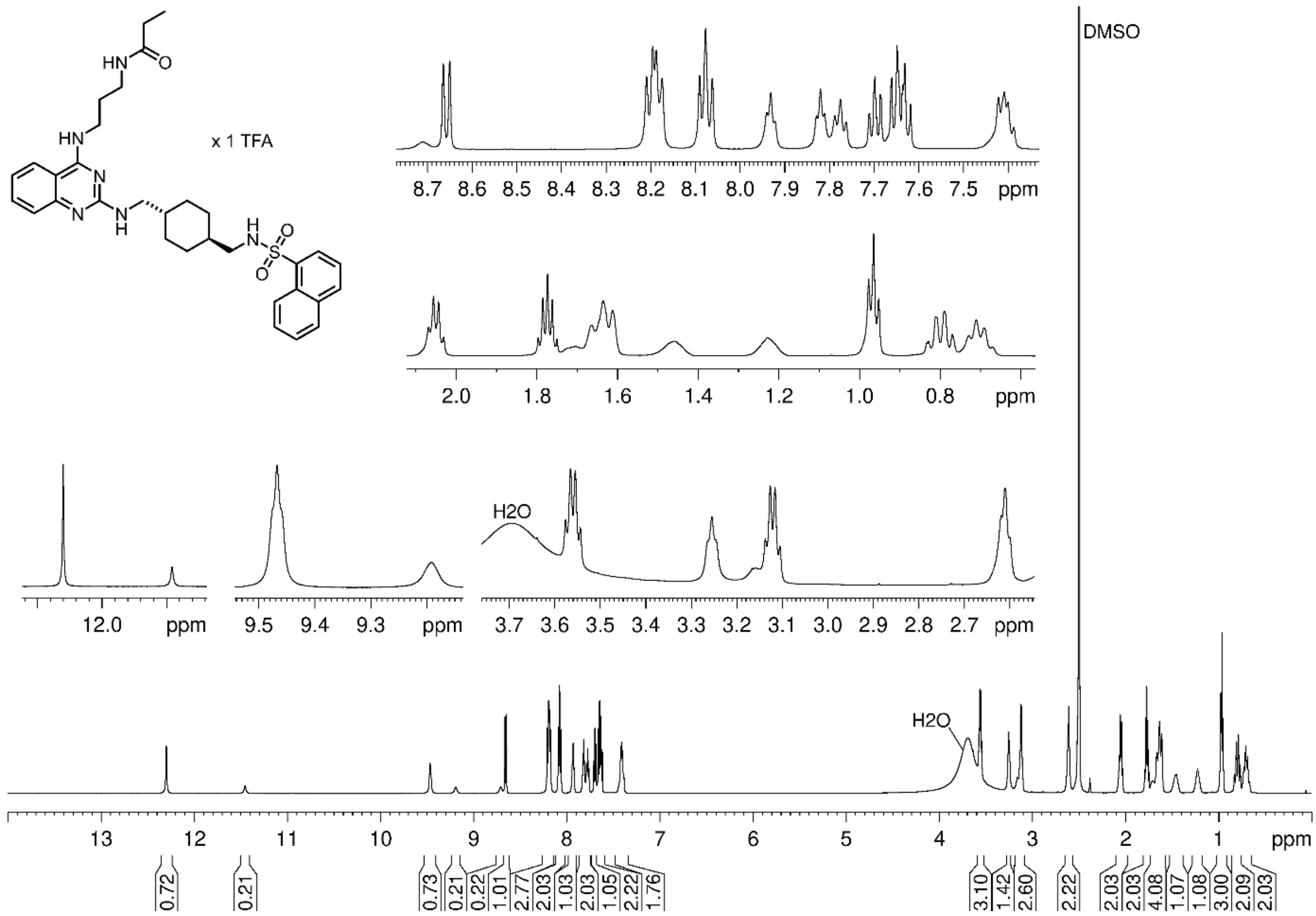

$^{1}$H-NMR spectrum (600 MHz, DMSO-$d_6$) of compound **33**

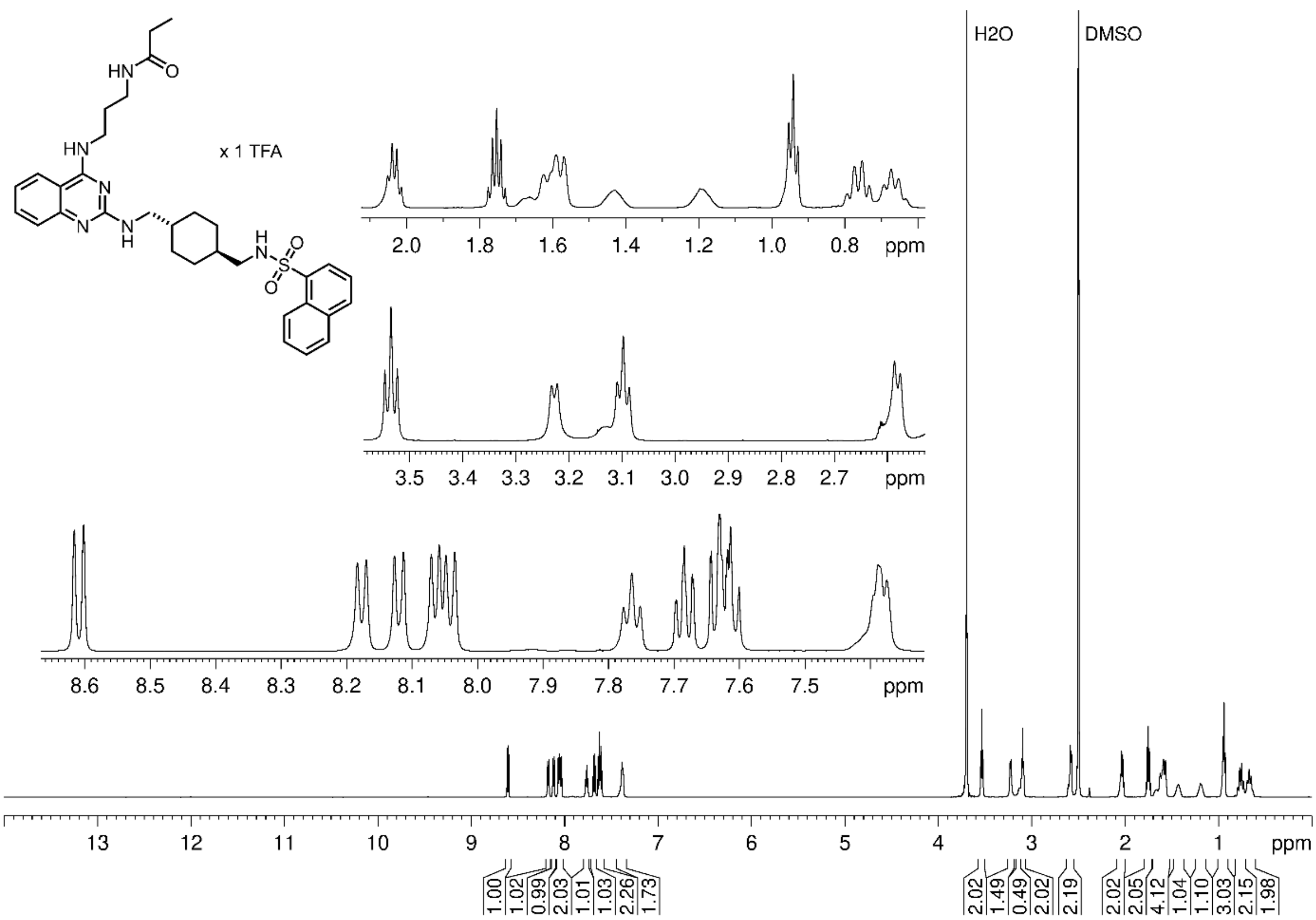


$^{1}$H-NMR spectrum (600 MHz, DMSO-$d_6$/$D_2O$ 9:1 v/v) of compound **33**

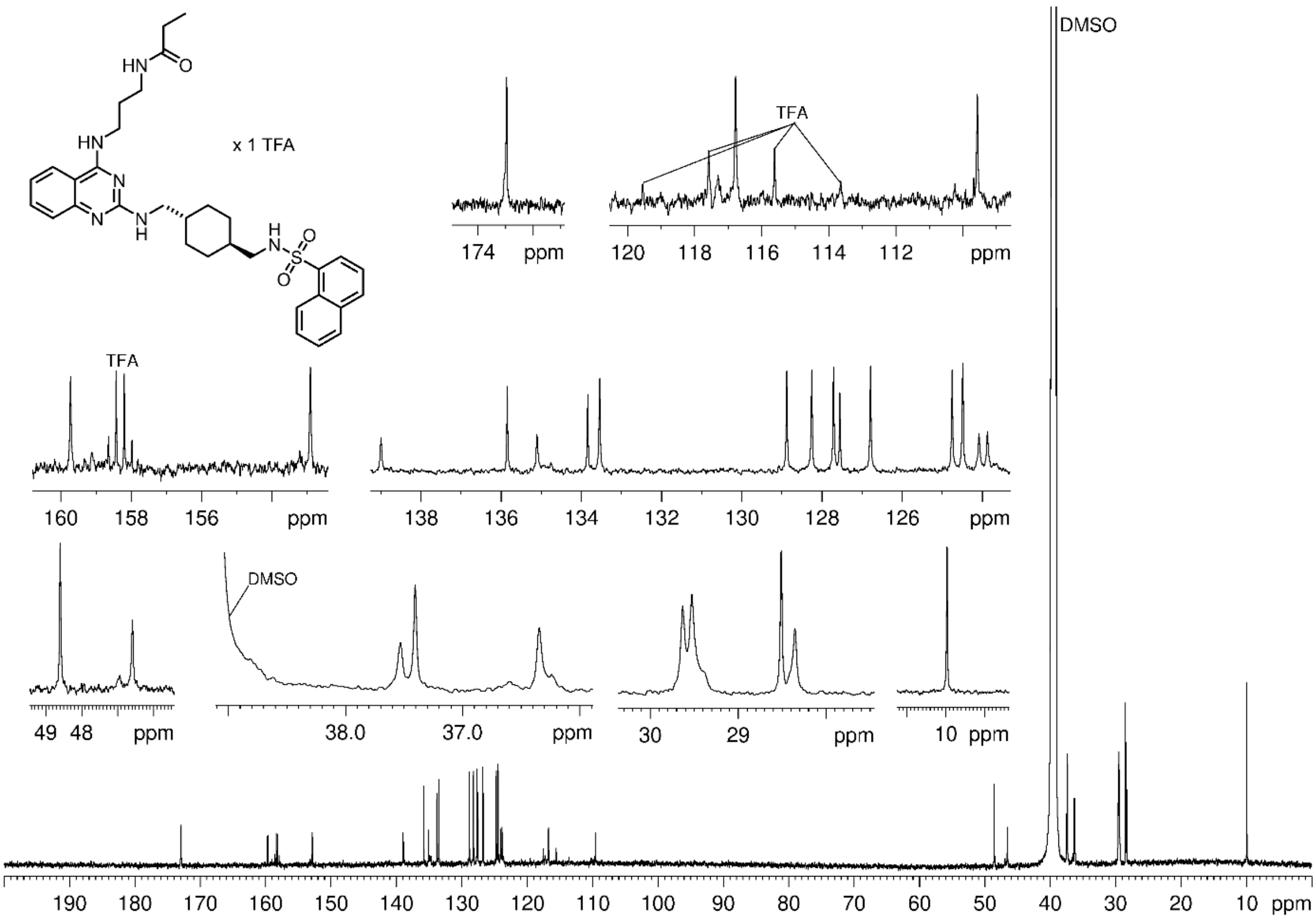


$^{13}$C-NMR spectrum (150 MHz, DMSO-$d_6$) of compound **33**

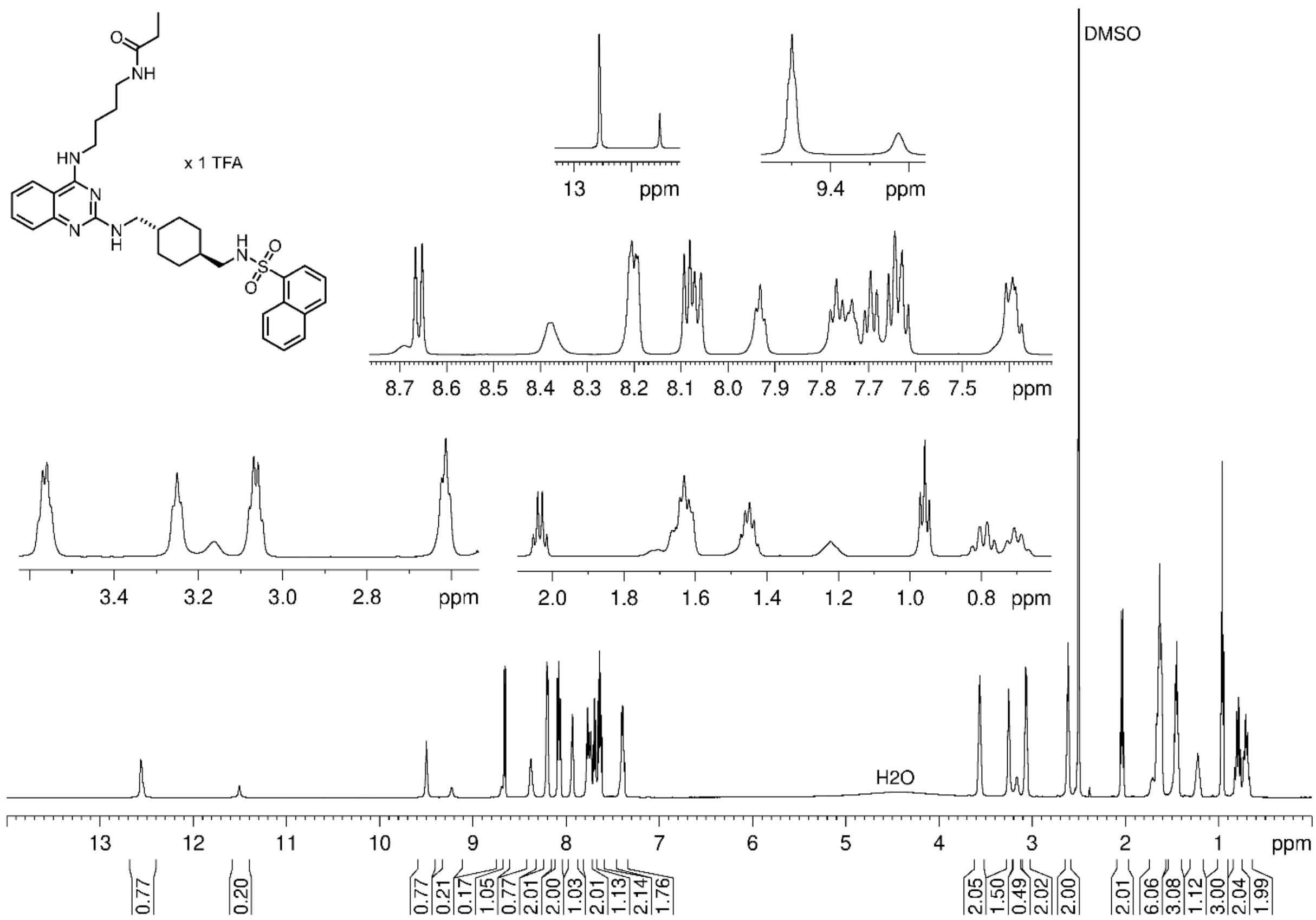

$^{1}$H-NMR spectrum (600 MHz, DMSO-$d_6$) of compound **34**

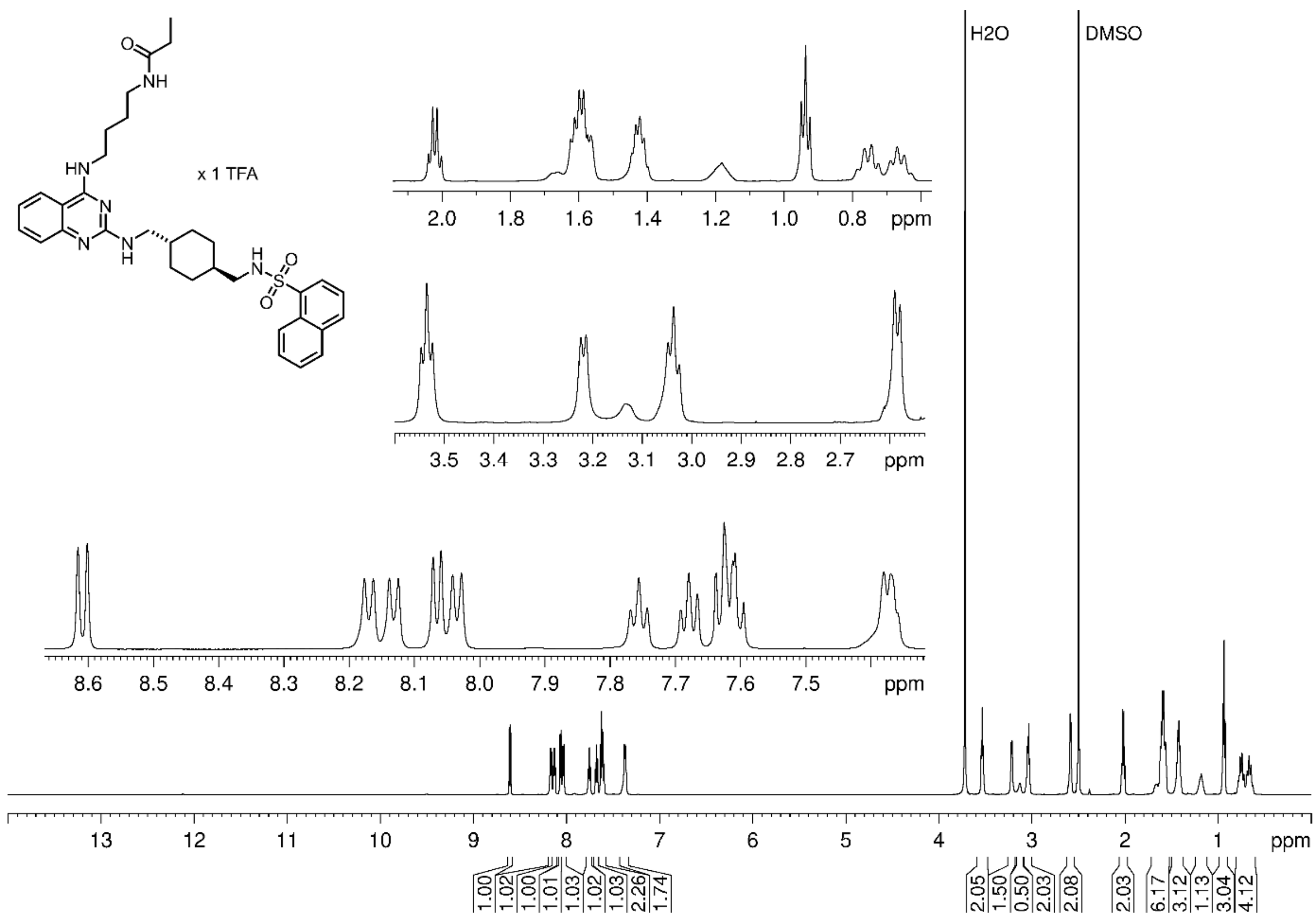

$^{1}$H-NMR spectrum (600 MHz, DMSO-$d_6$/$D_2O$ 9:1 v/v) of compound **34**

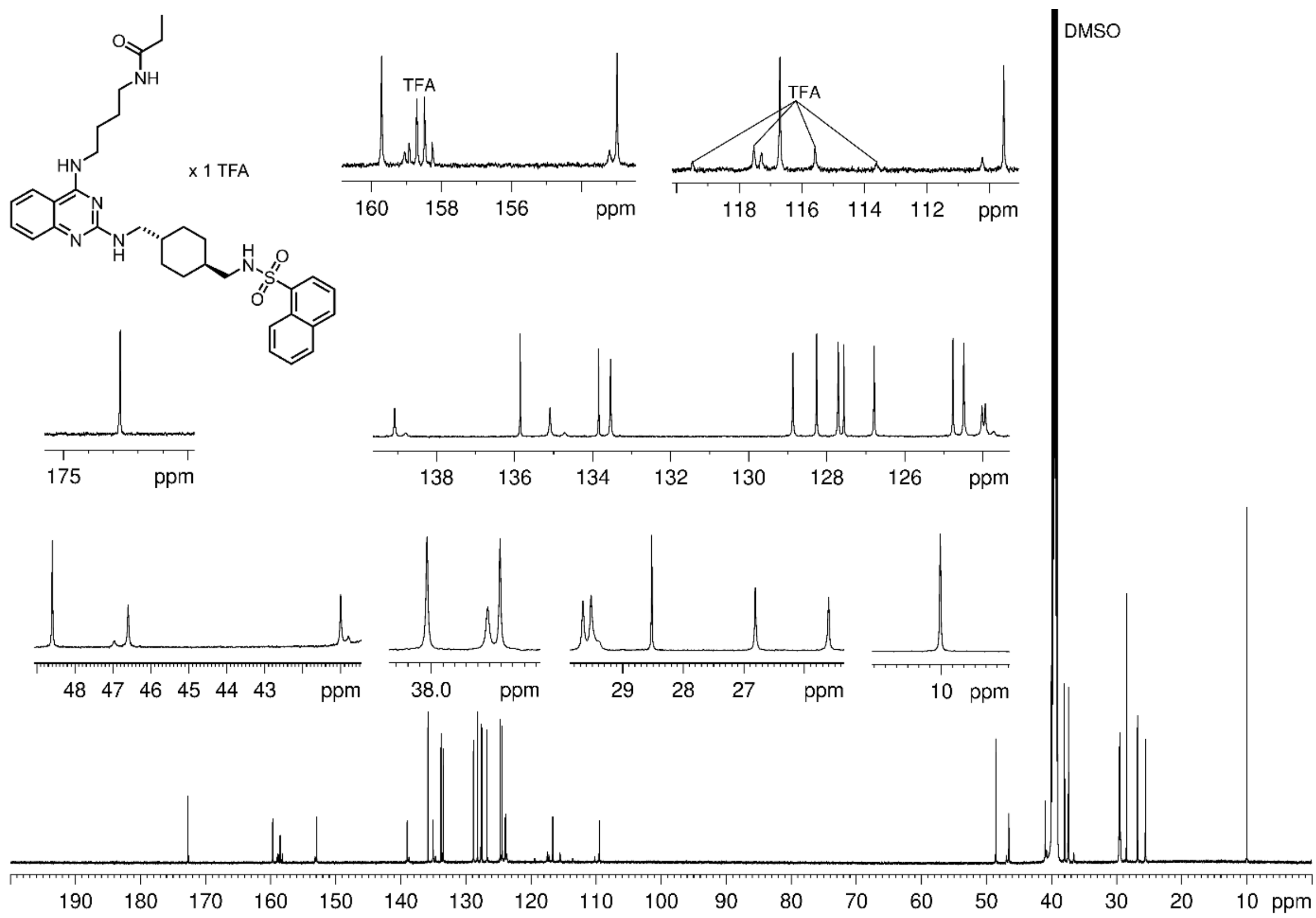


$^{13}$C-NMR spectrum (150 MHz, DMSO-$d_6$) of compound **34**

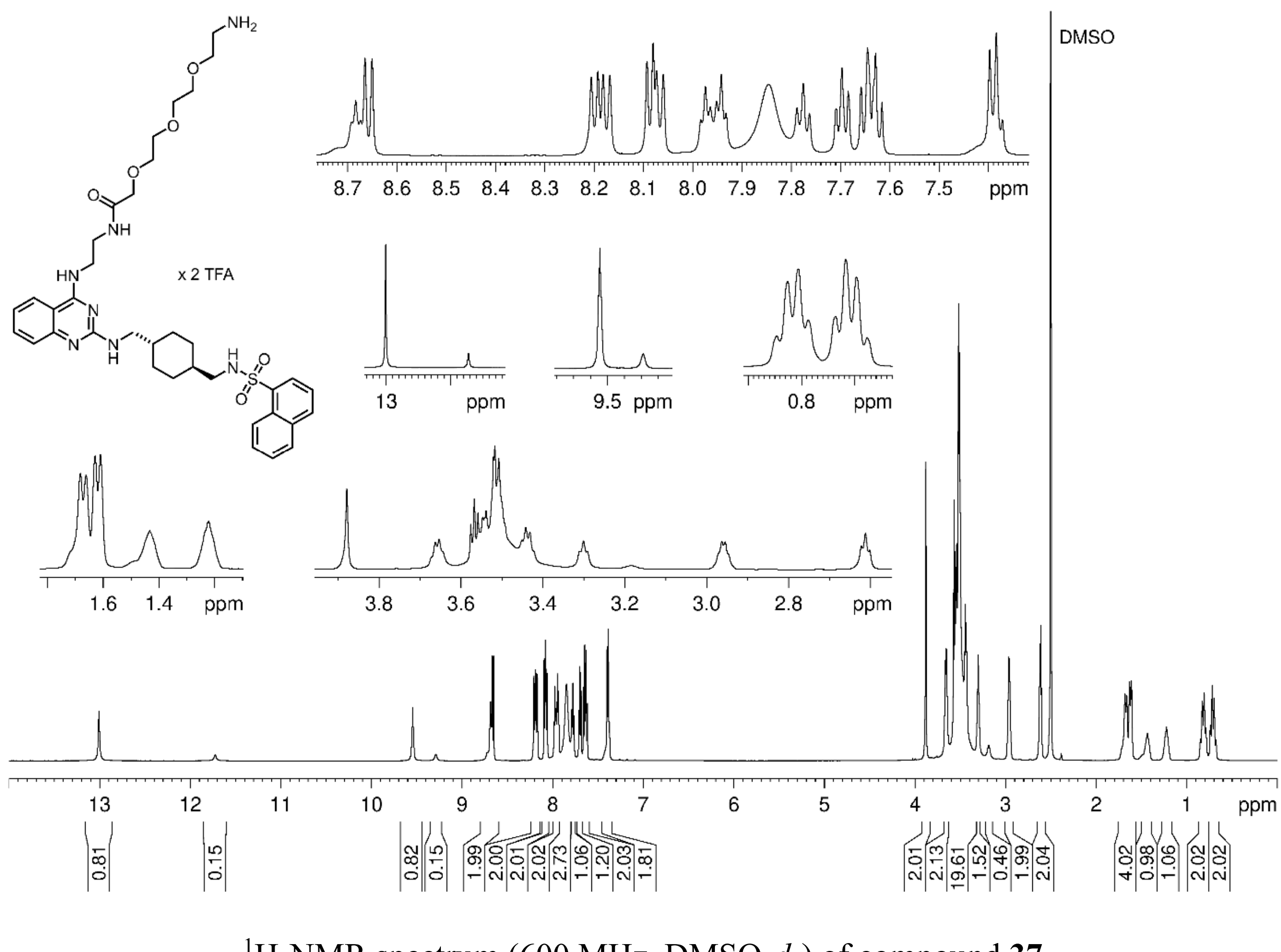


$^{1}$H-NMR spectrum (600 MHz, DMSO-$d_6$) of compound **37**

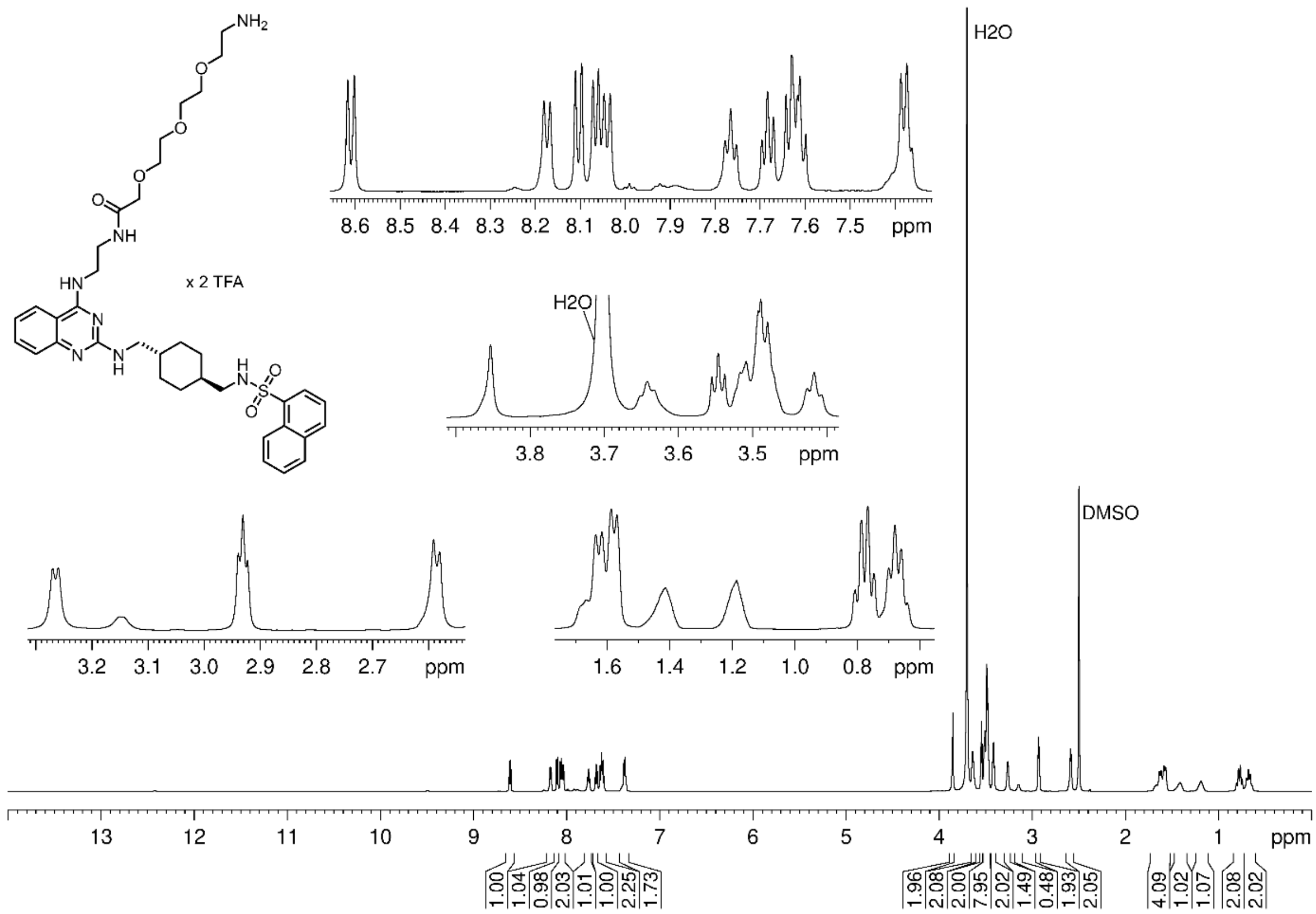


$^{1}$H-NMR spectrum (600 MHz, DMSO-$d_6$/$D_2O$ 9:1 v/v) of compound **37**

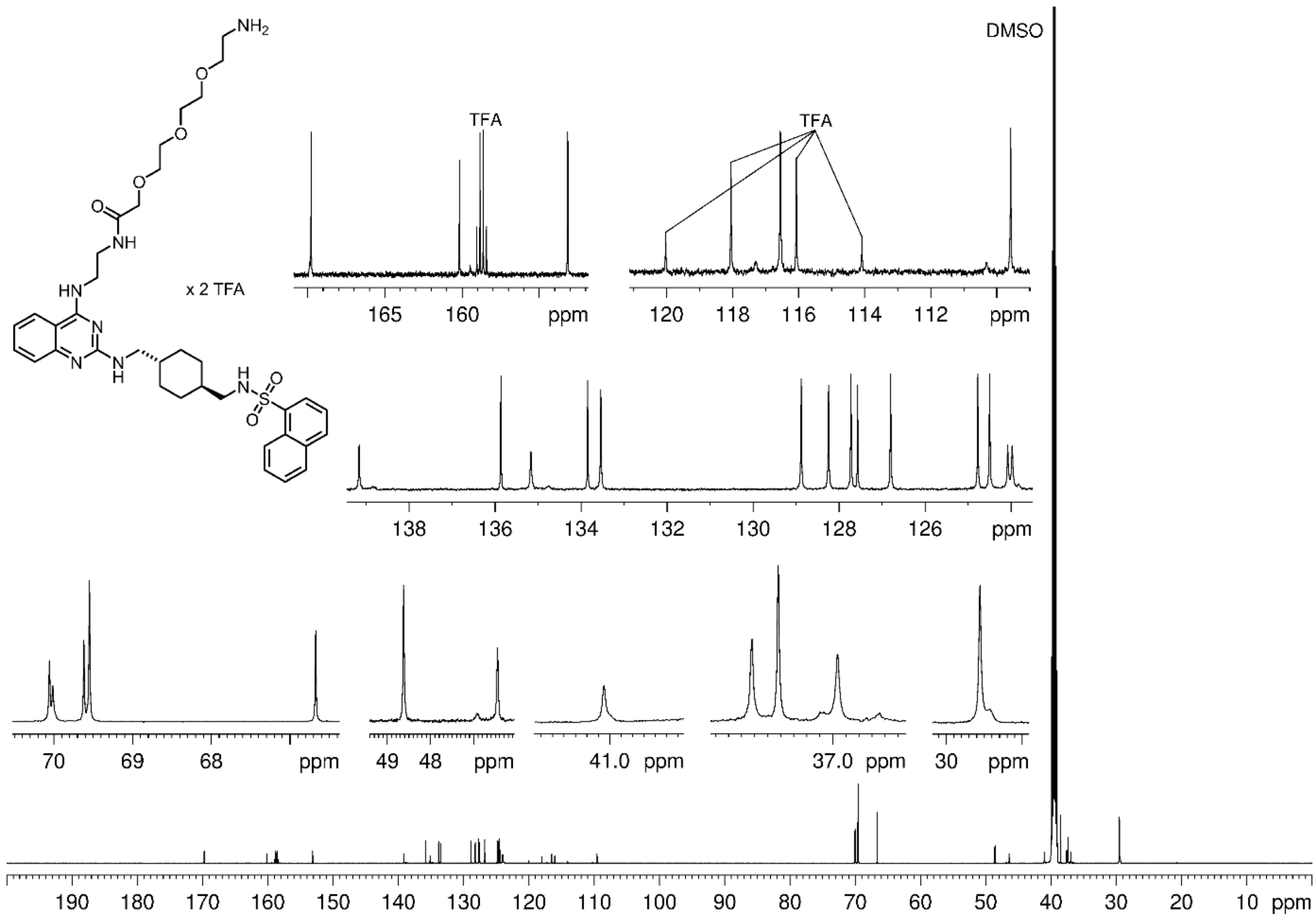


$^{13}$C-NMR spectrum (151 MHz, DMSO-$d_6$) of compound **37**

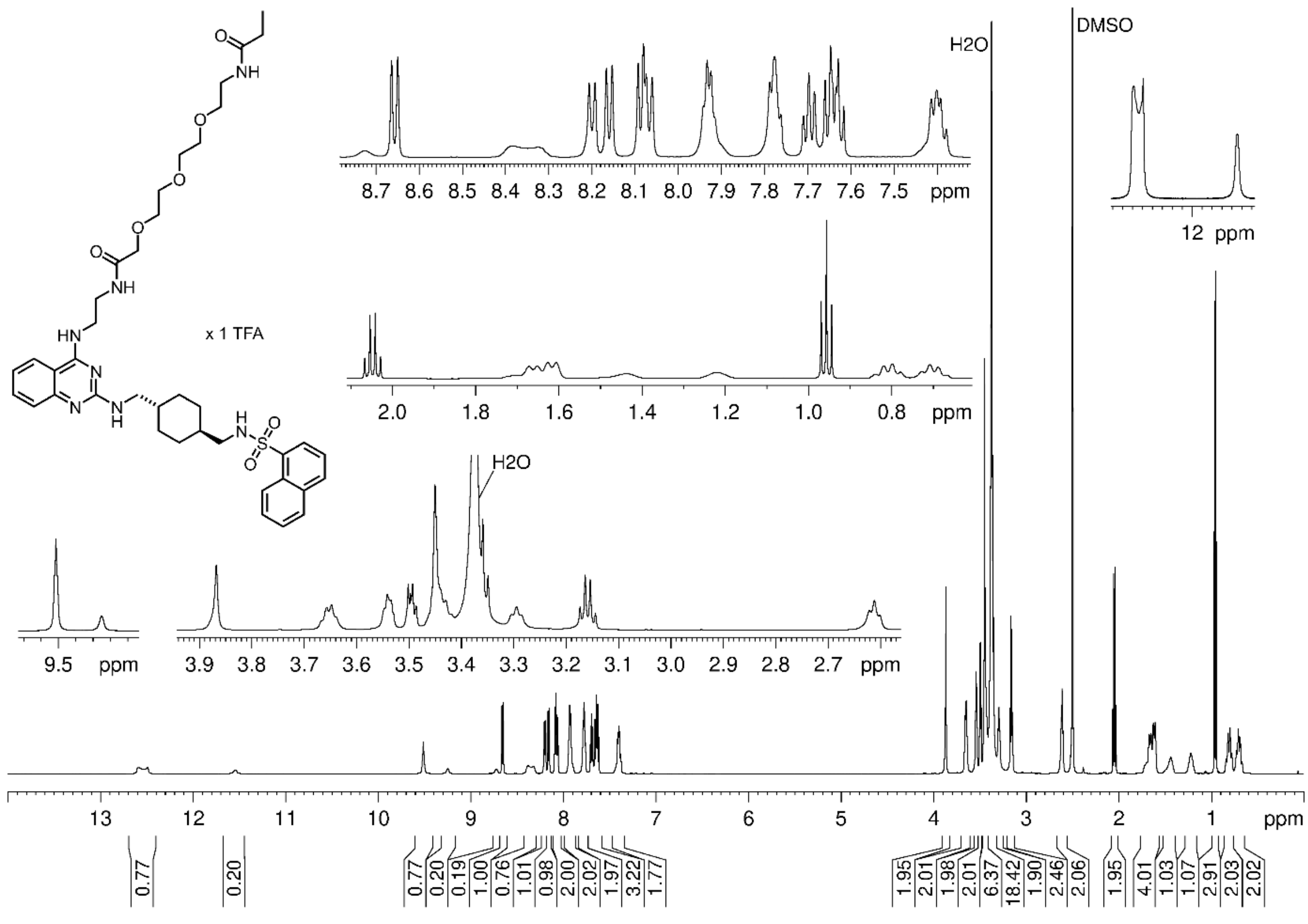


$^{1}$H-NMR spectrum (600 MHz, DMSO-$d_6$) of compound **38**

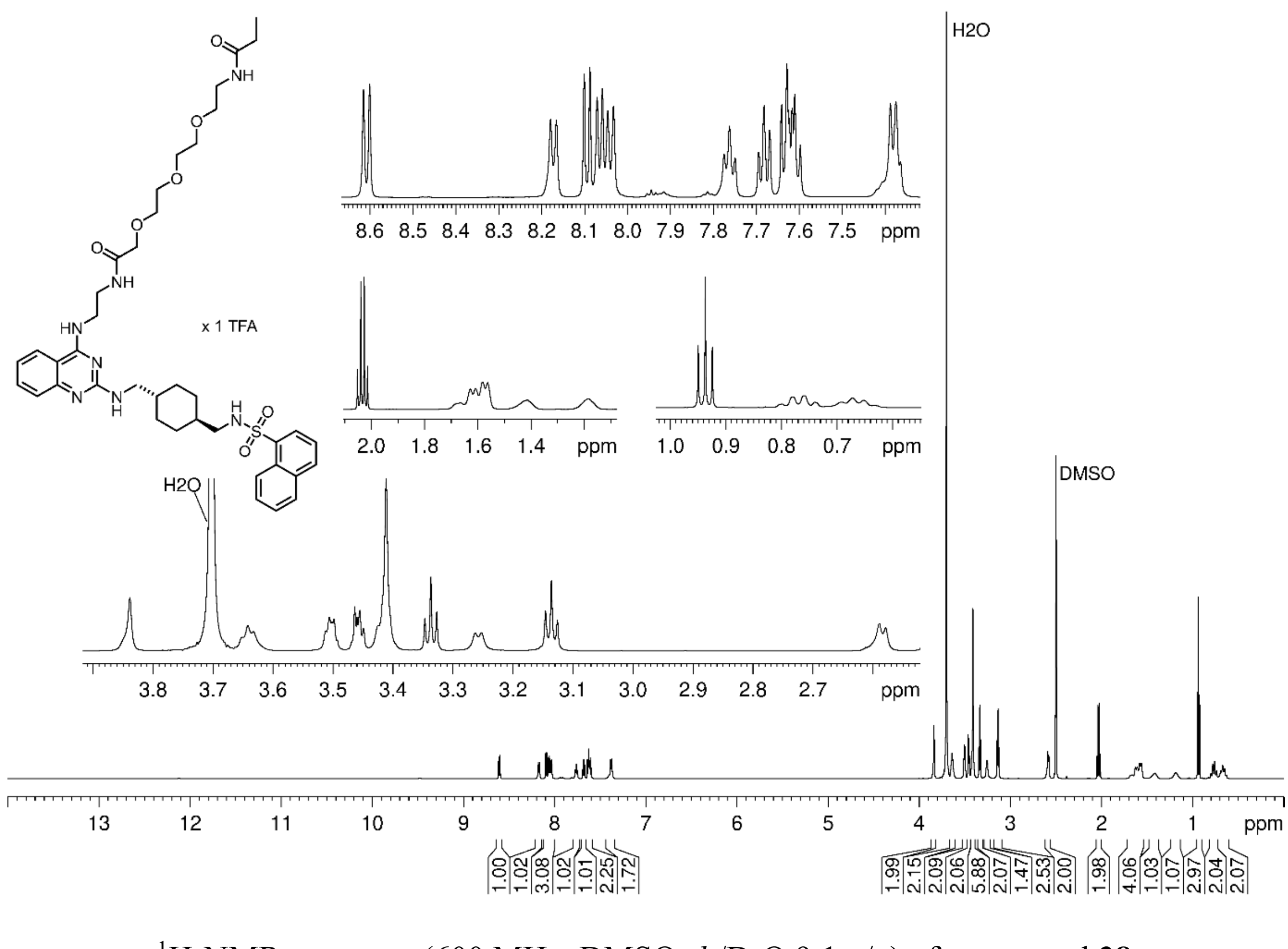


$^{1}$H-NMR spectrum (600 MHz, DMSO-$d_6$/$D_2O$ 9:1 v/v) of compound **38**

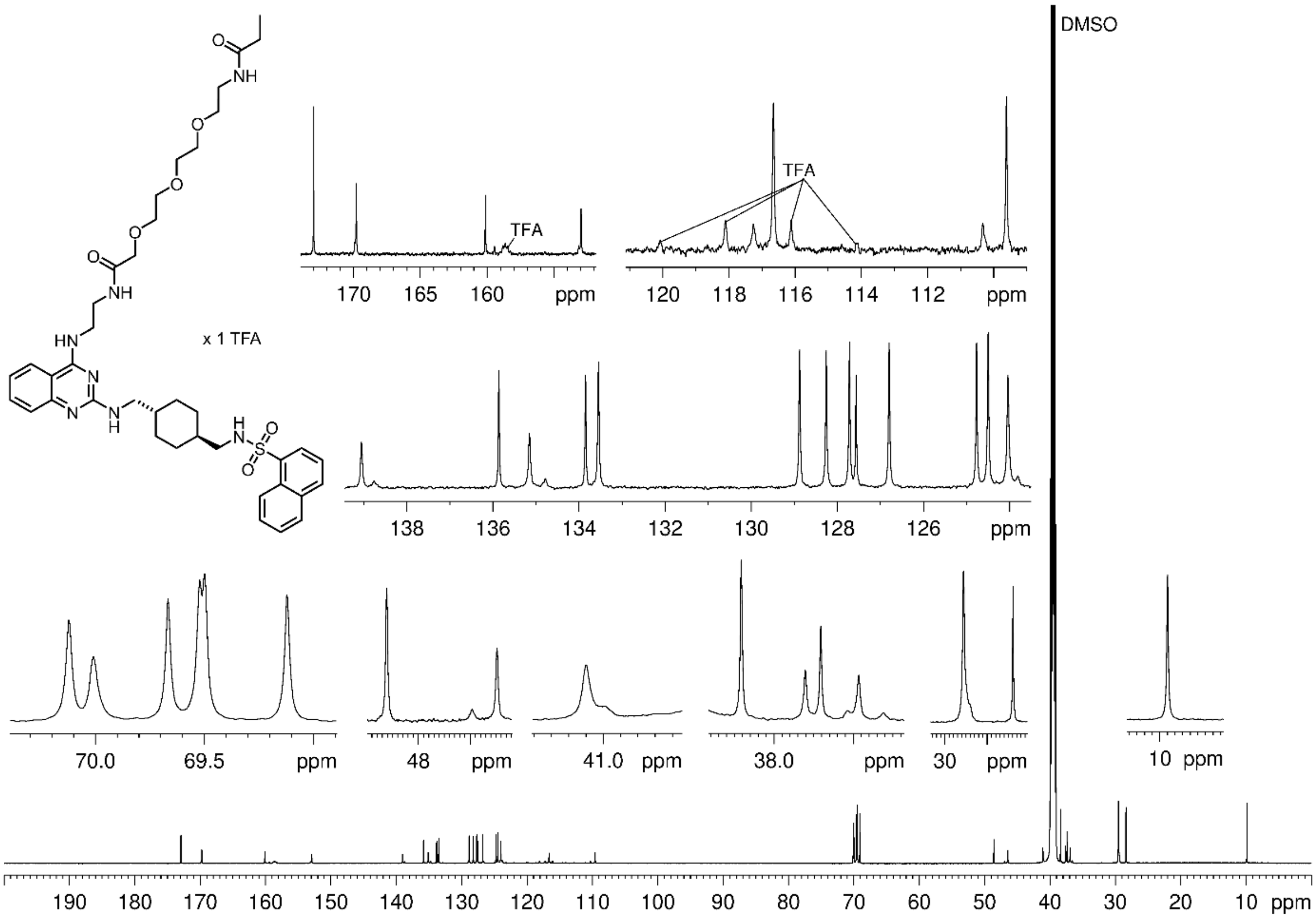


$^{13}$C-NMR spectrum (151 MHz, DMSO-$d_6$) of compound **38**

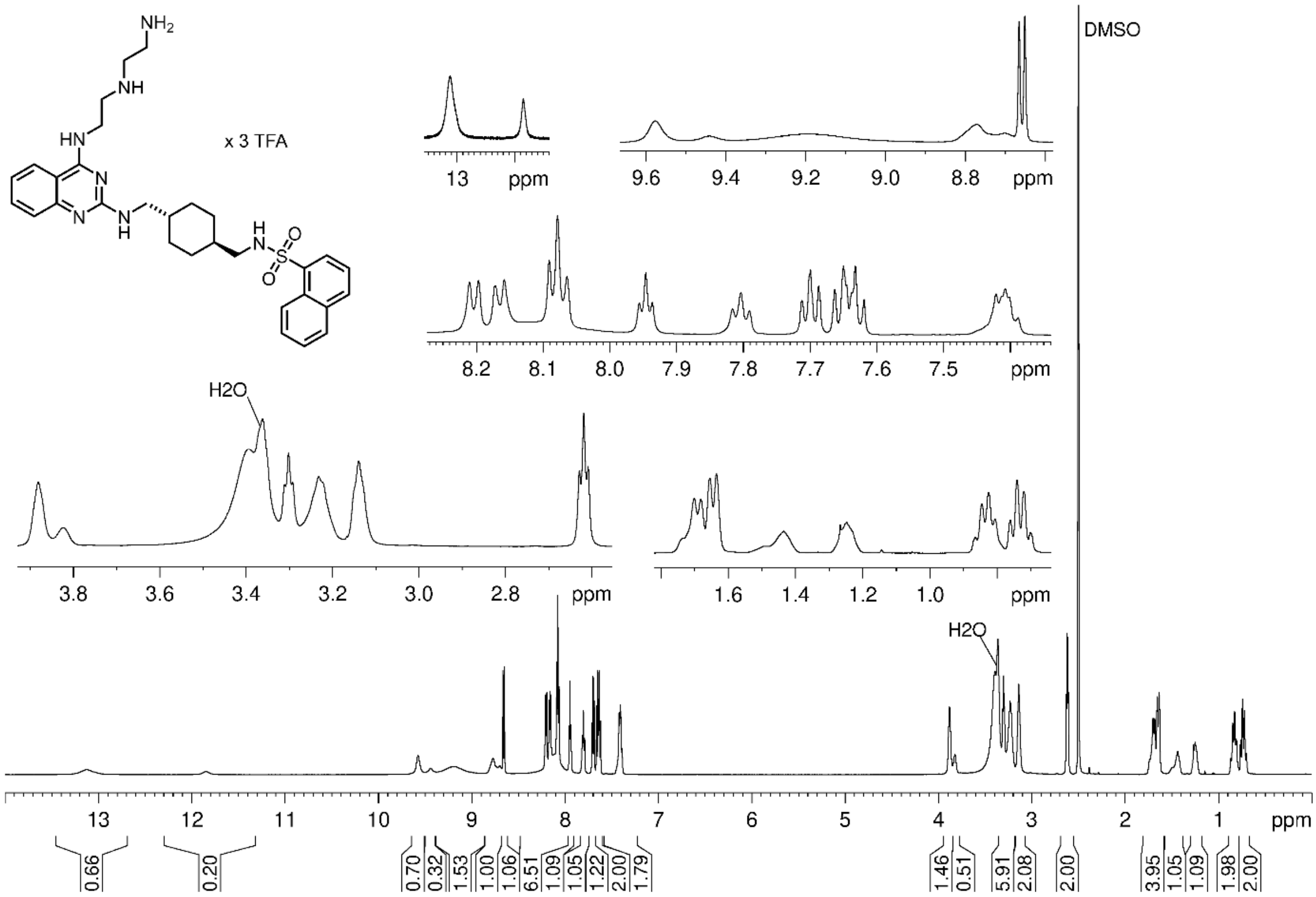


$^{1}$H-NMR spectrum (600 MHz, DMSO-$d_6$) of compound **41**

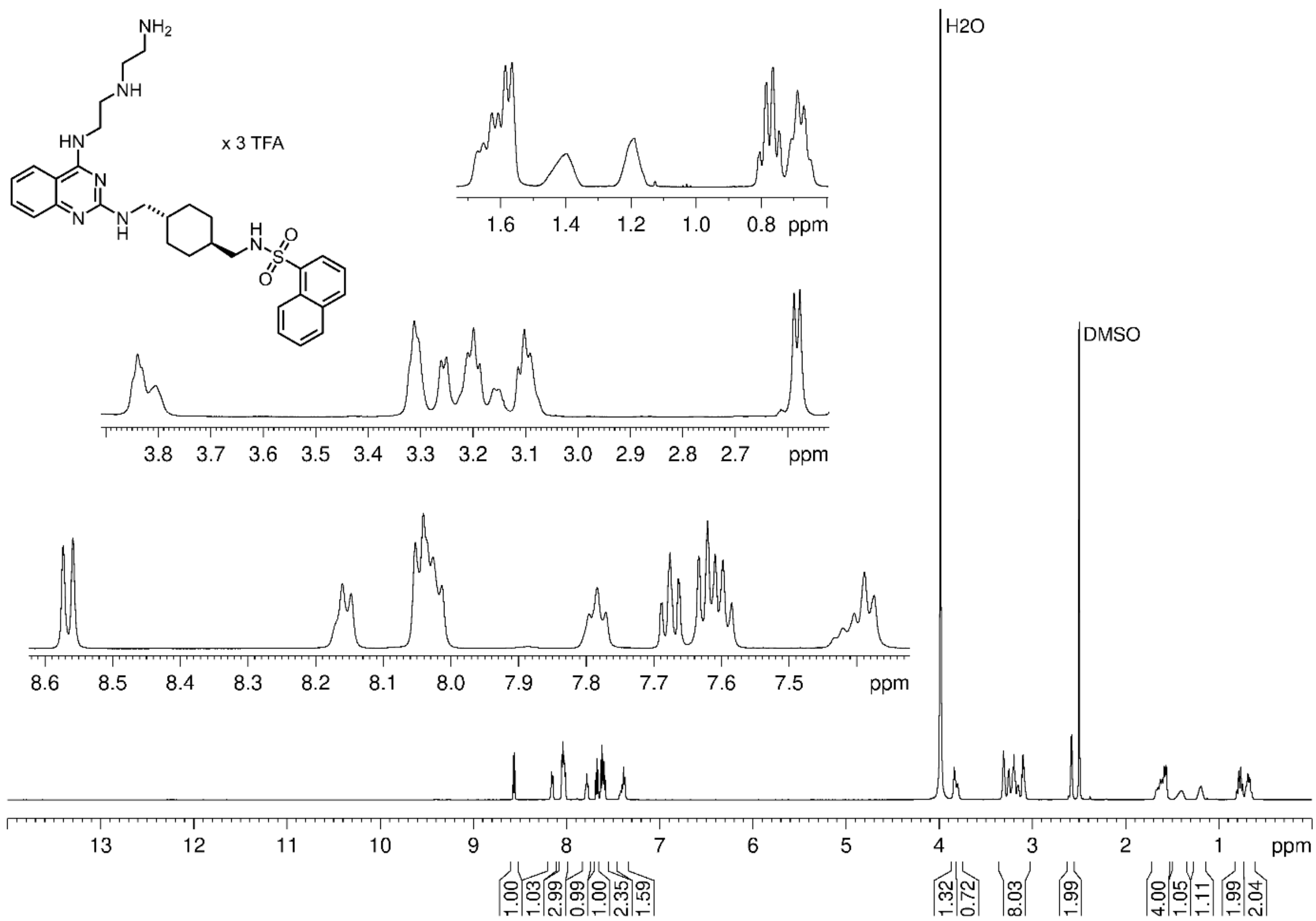


$^{1}$H-NMR spectrum (600 MHz, DMSO-$d_6$/$D_2O$ 9:1 v/v) of compound **41**

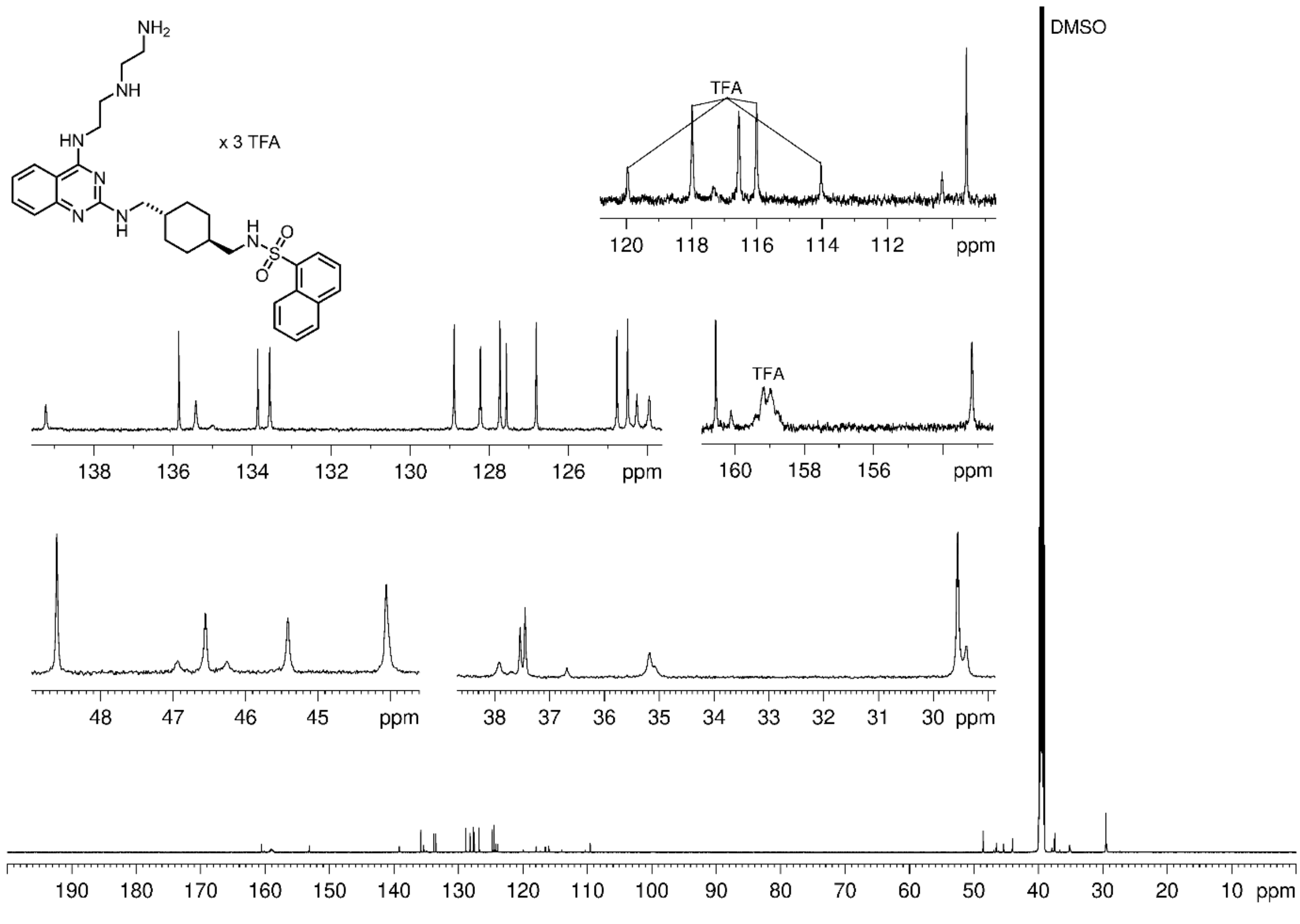


$^{13}$C-NMR spectrum (151 MHz, DMSO-$d_6$) of compound **41**

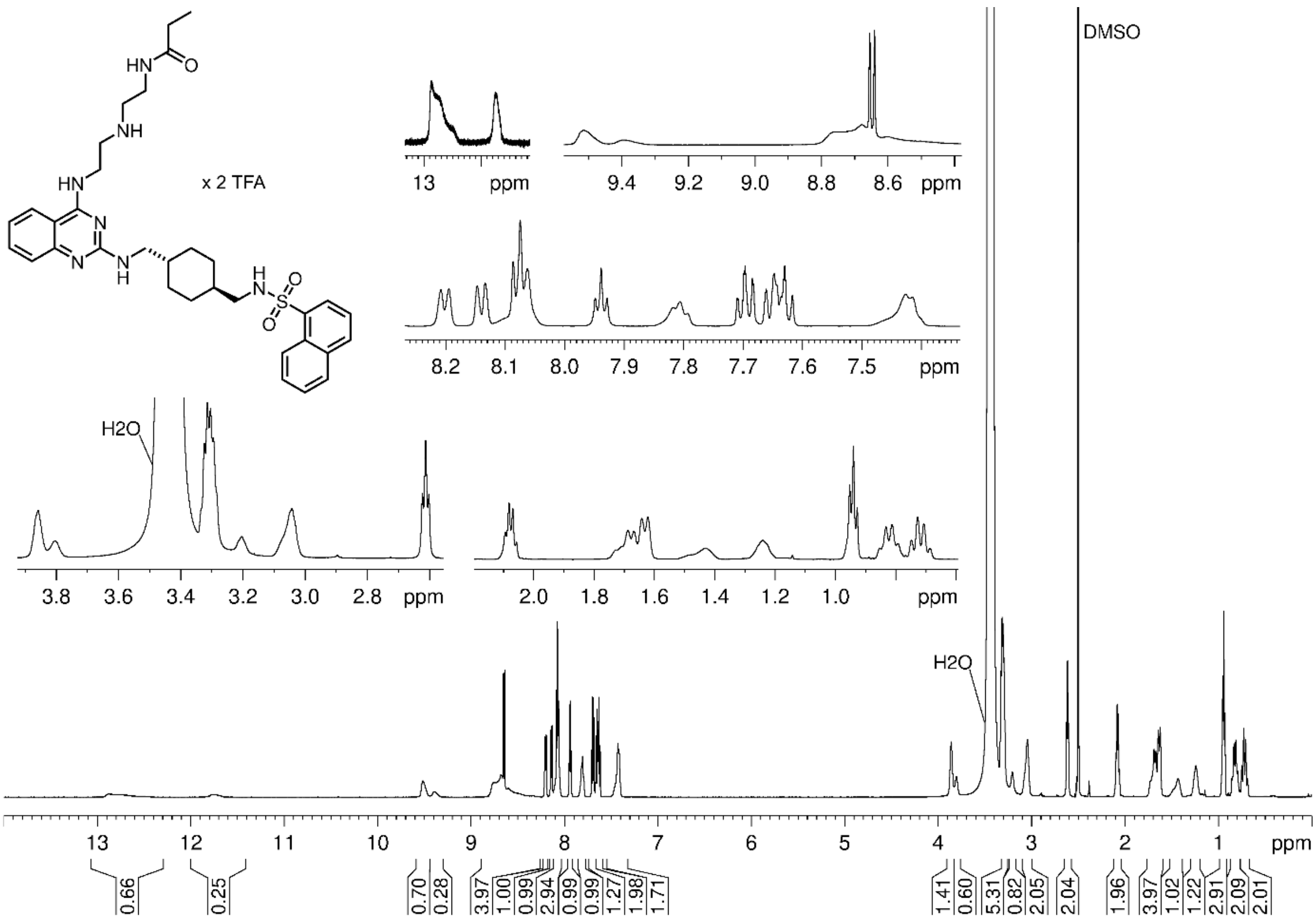


$^1$H-NMR spectrum (600 MHz, DMSO-$d_6$) of compound **42**

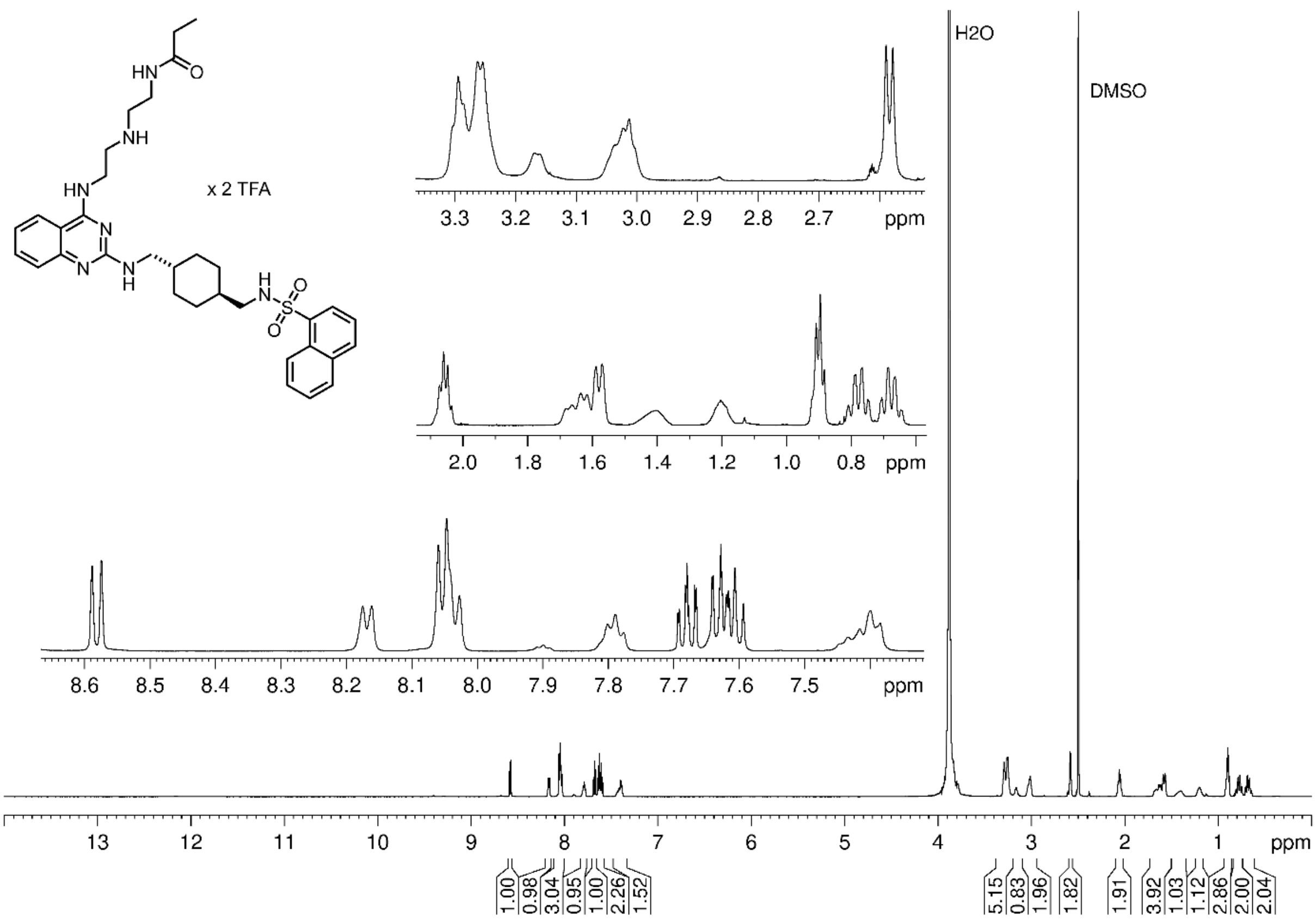


$^1$H-NMR spectrum (600 MHz, DMSO-$d_6$/$D_2O$ 9:1 v/v) of compound **42**

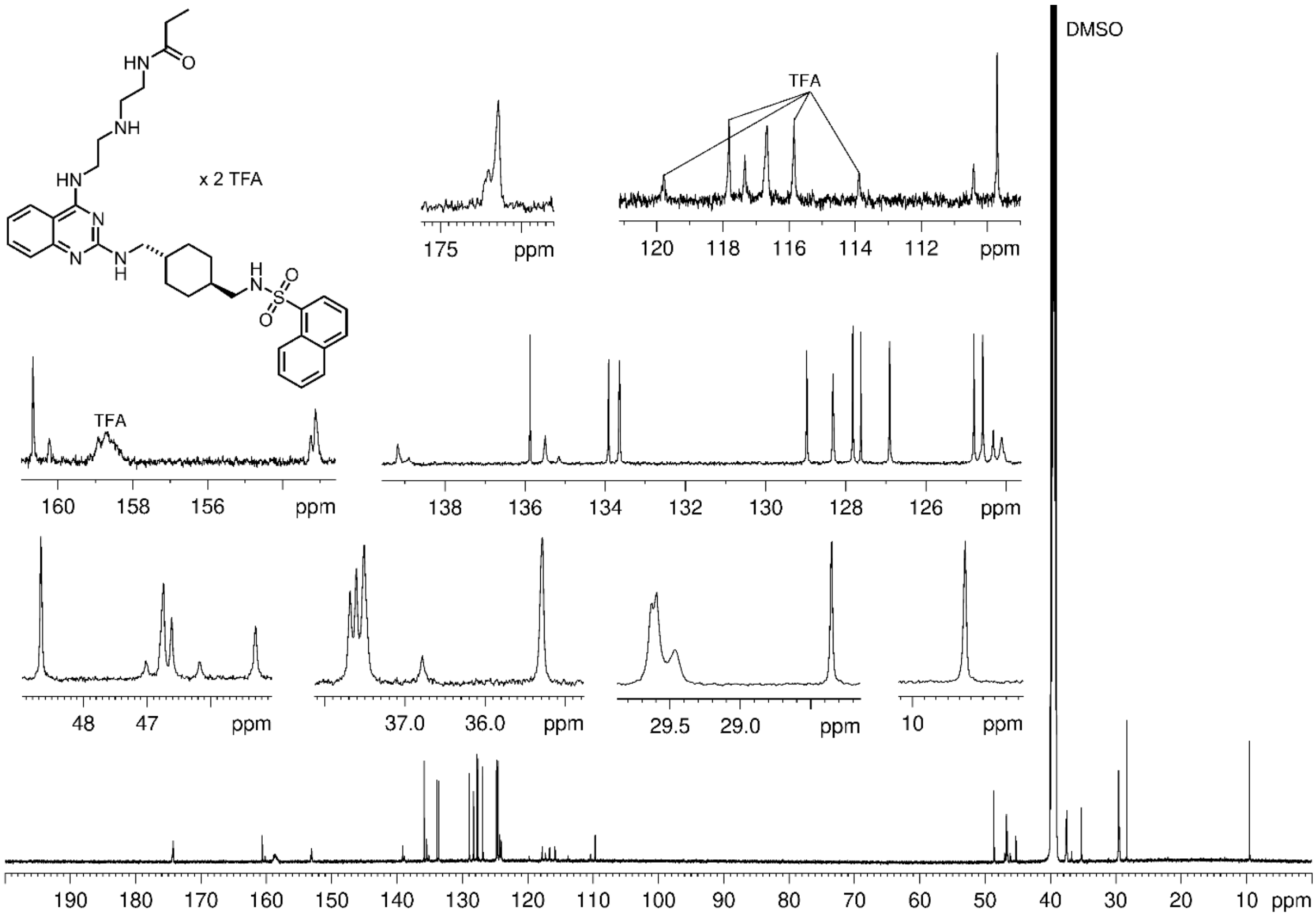


$^{13}$C-NMR spectrum (151 MHz, DMSO-$d_6$) of compound **42**

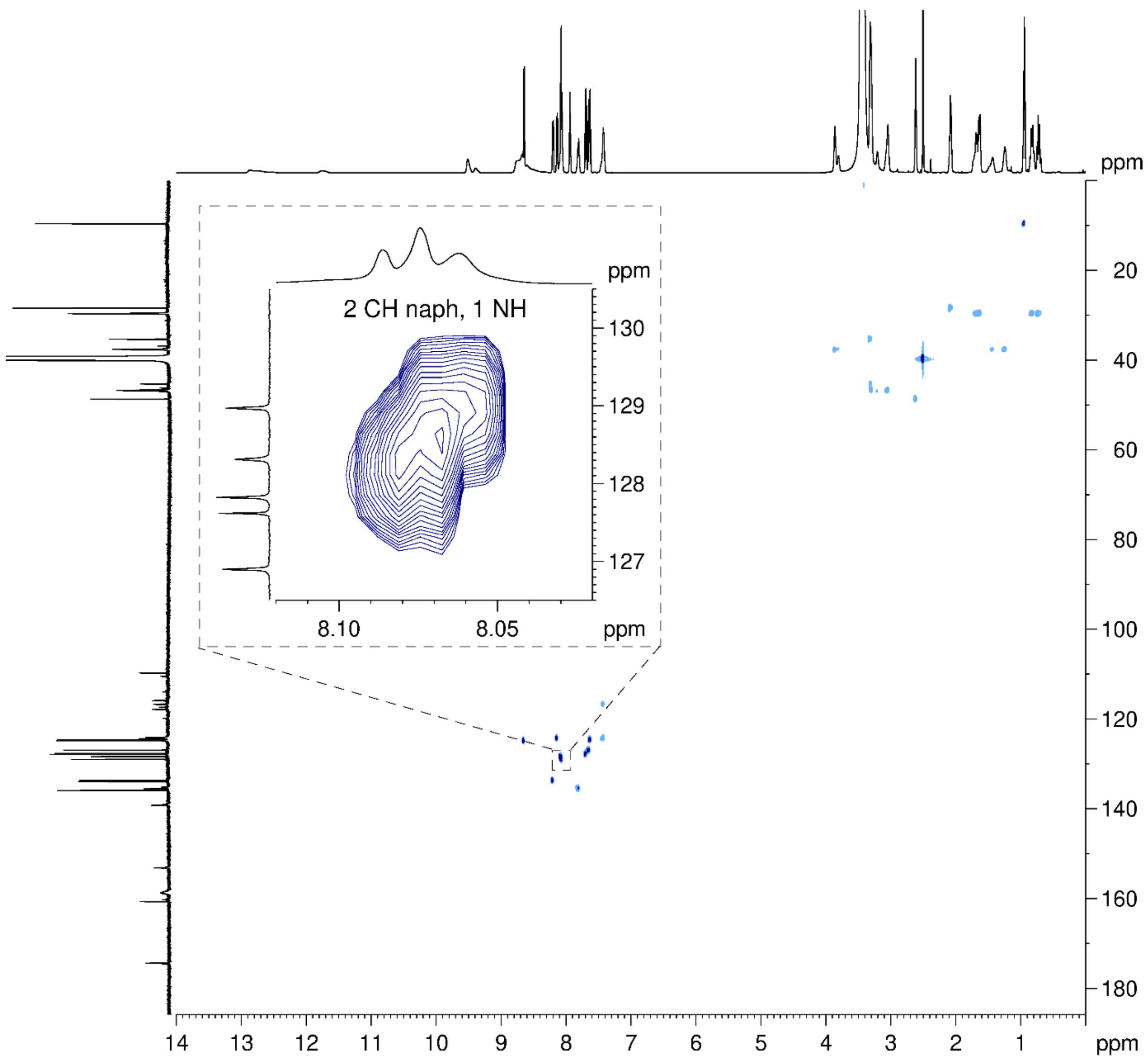


$^{1}H/^{13}C$ HSQC spectrum of compound **42**

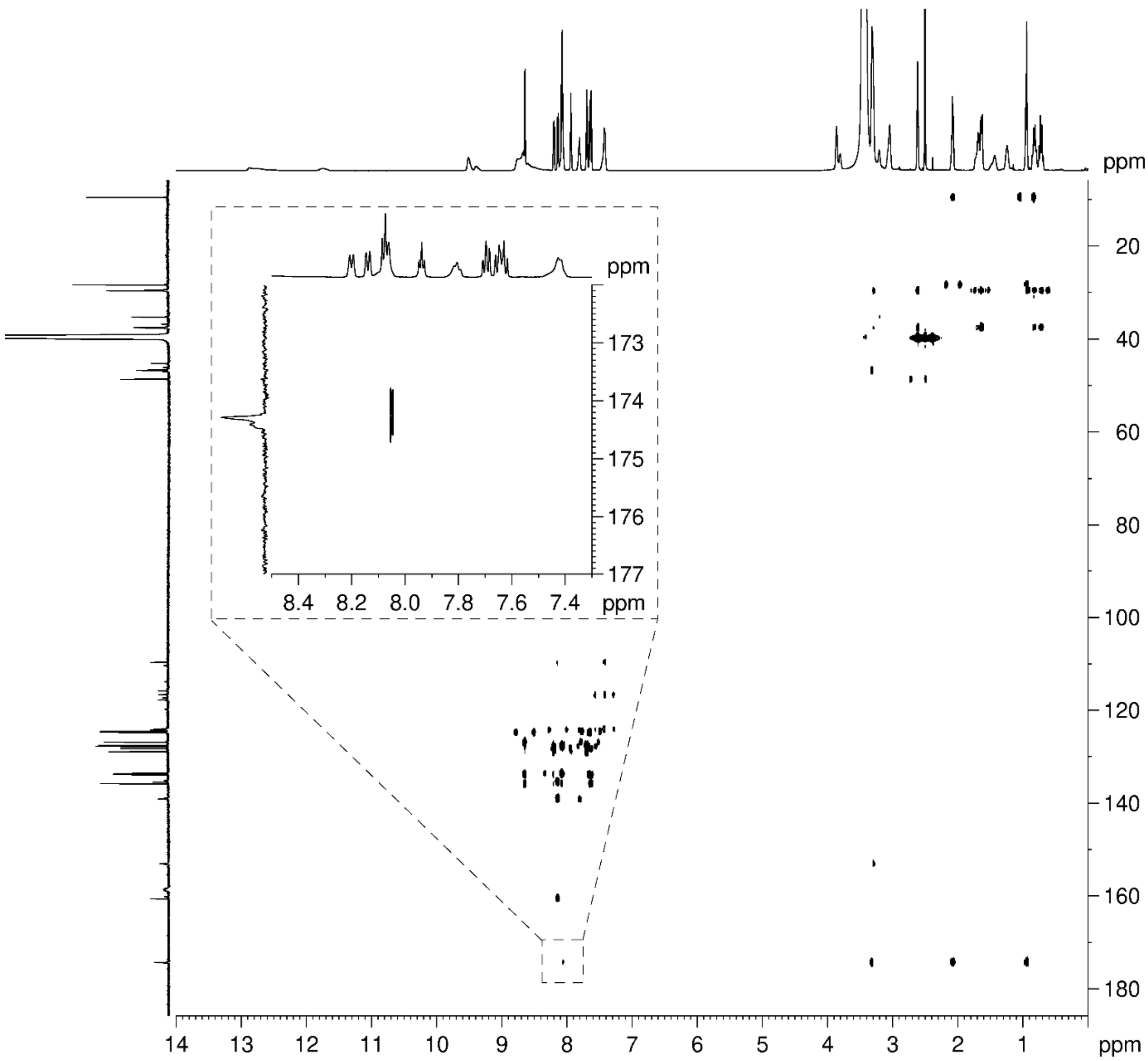


$^1H/^{13}C$ HMBC spectrum of compound **42**

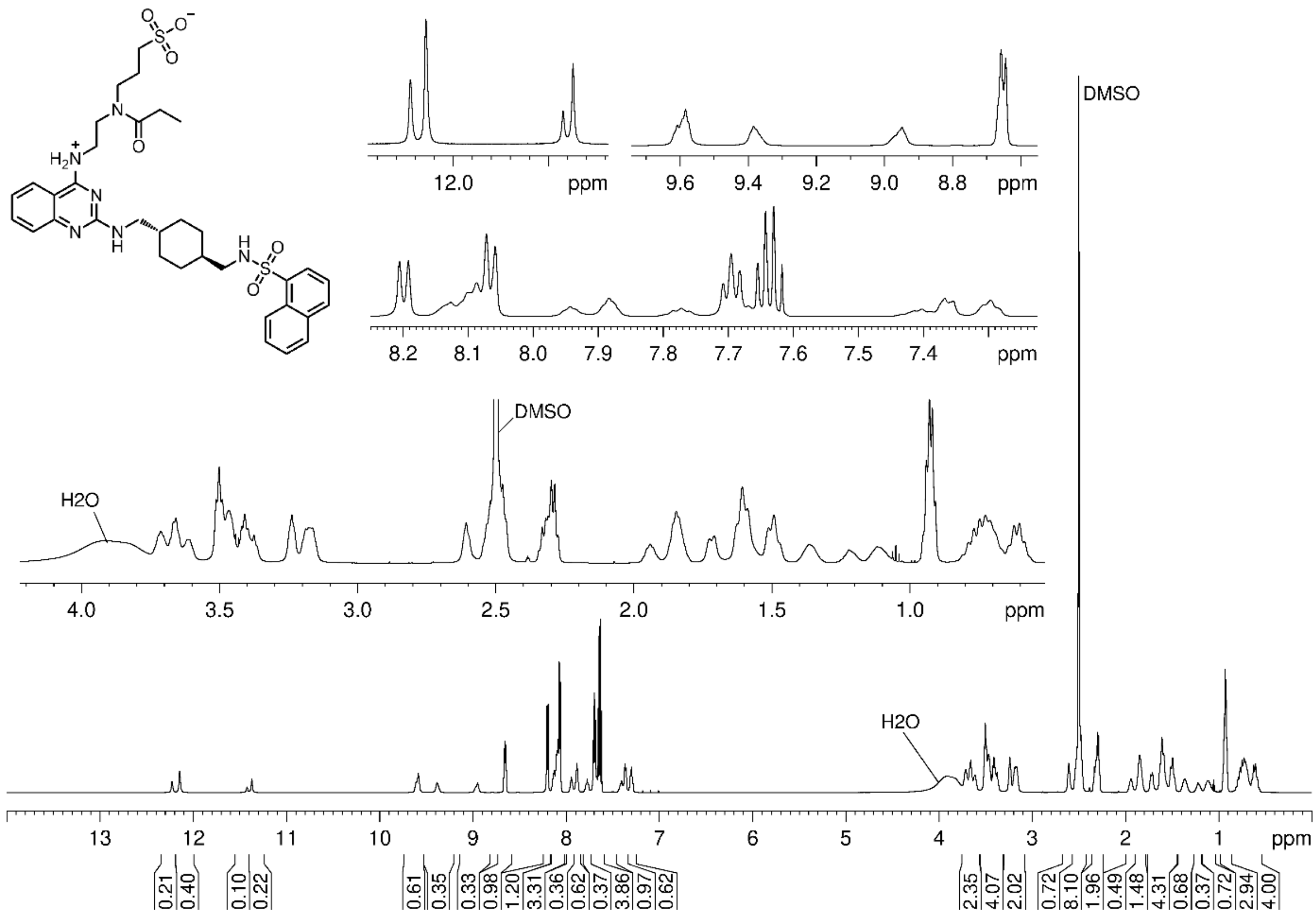


$^{1}$H-NMR spectrum (600 MHz, DMSO-$d_6$) of compound **45**

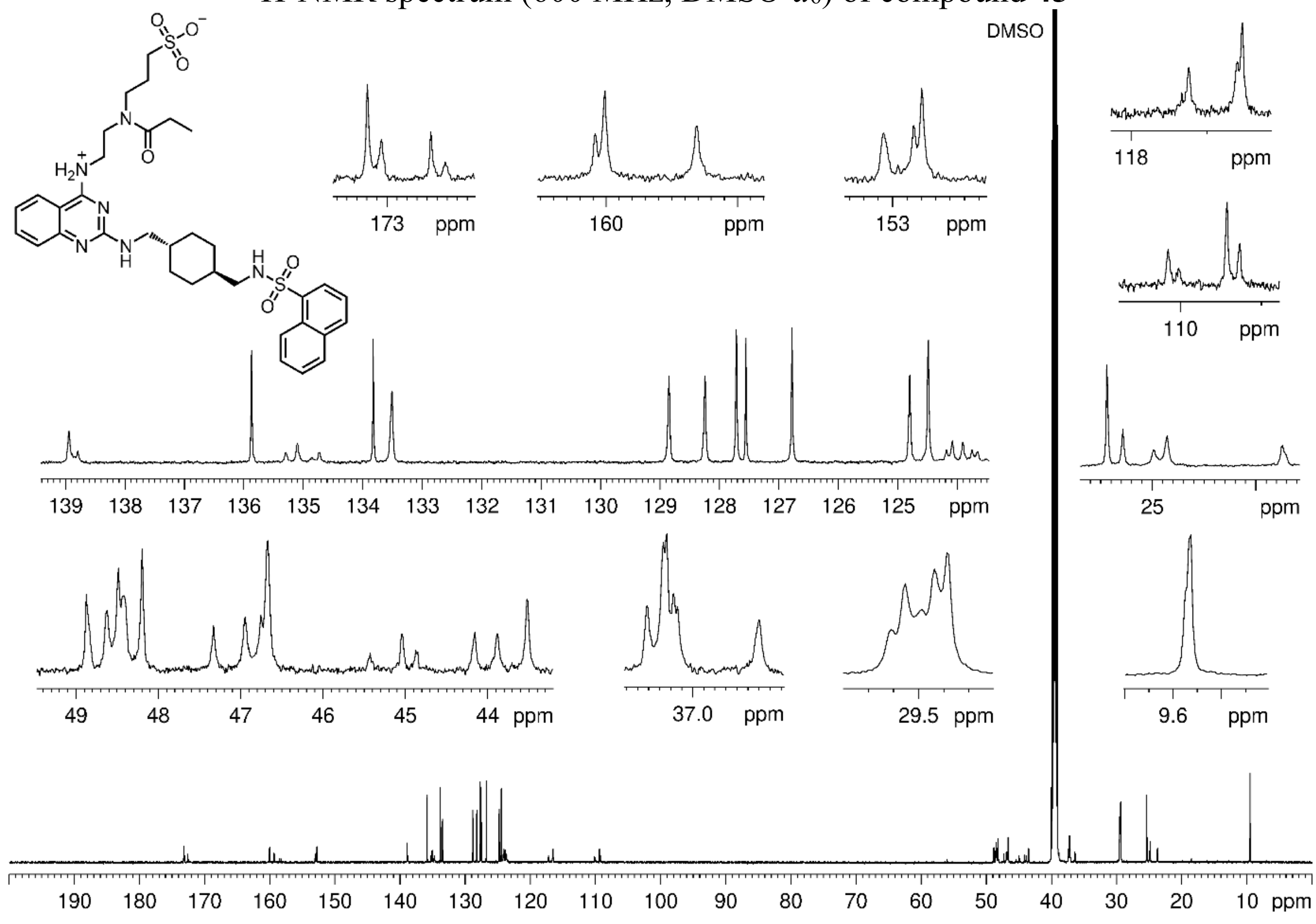


$^{13}$C-NMR spectrum (151 MHz, DMSO-$d_6$) of compound **45**

## 4. HRMS spectra of compounds 3, 26-30, 32-34, 37, 38, 41, 42, and 45

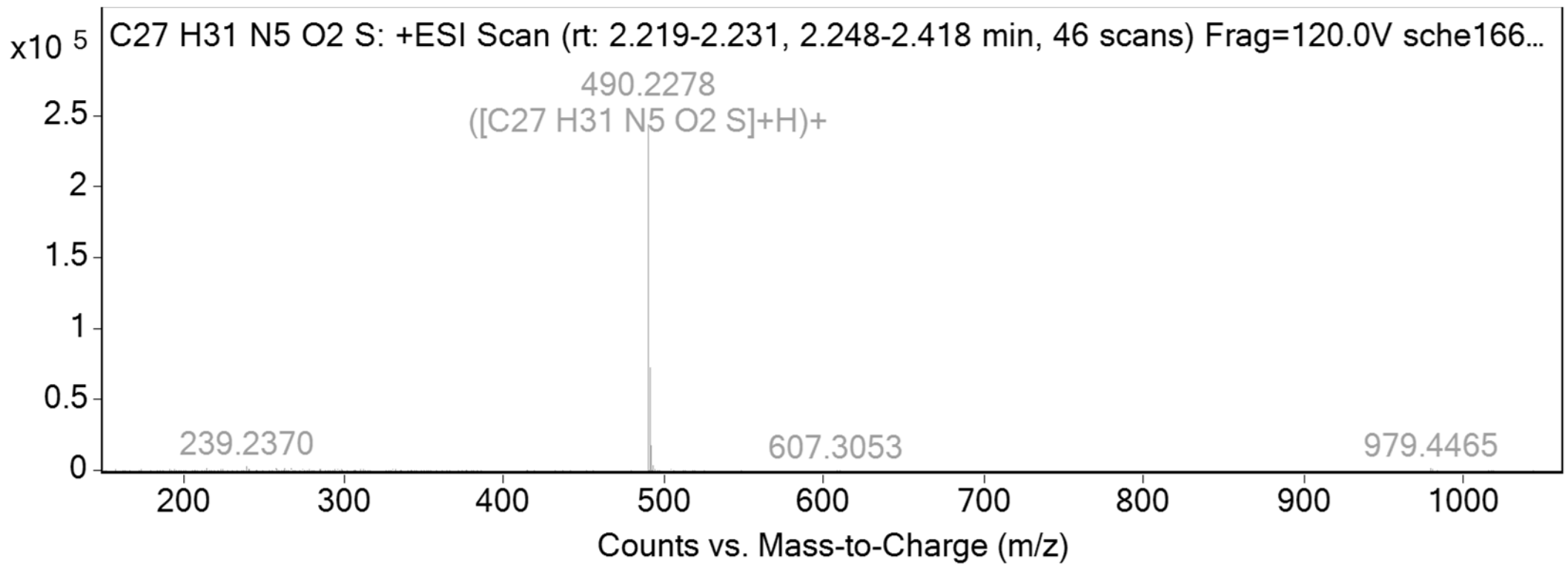


HRMS spectrum of compound **3**

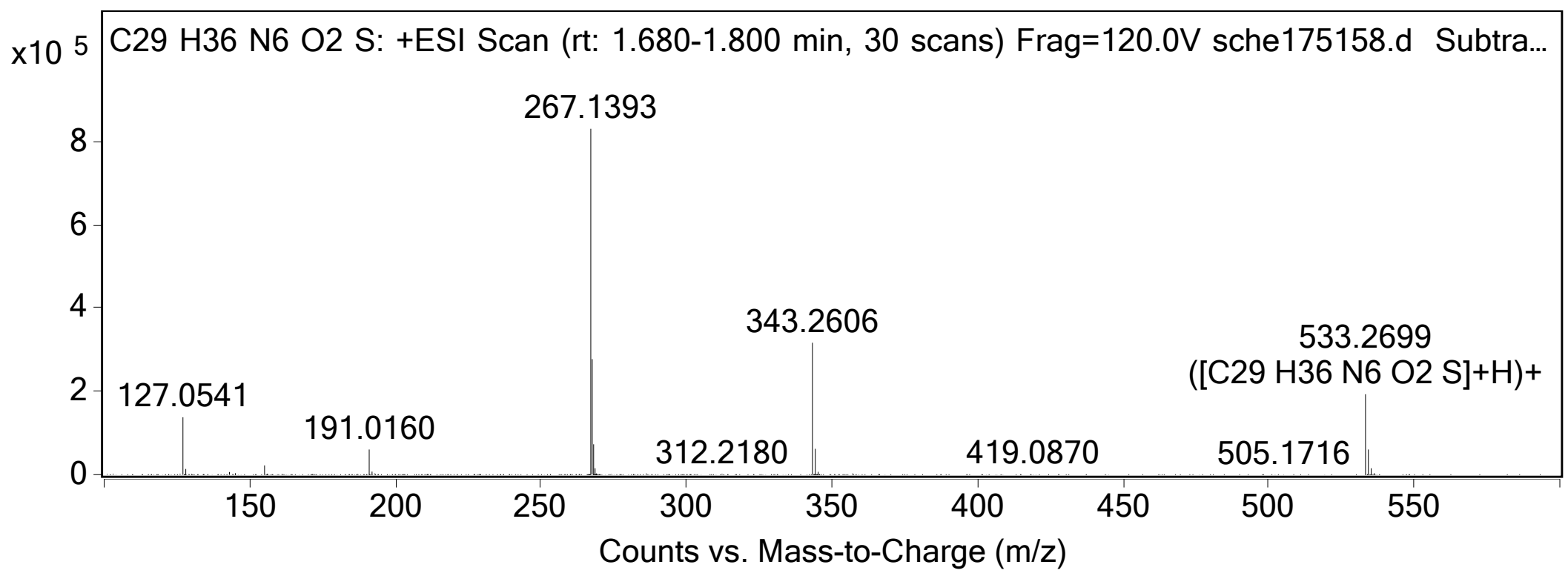


HRMS spectrum of compound **26**

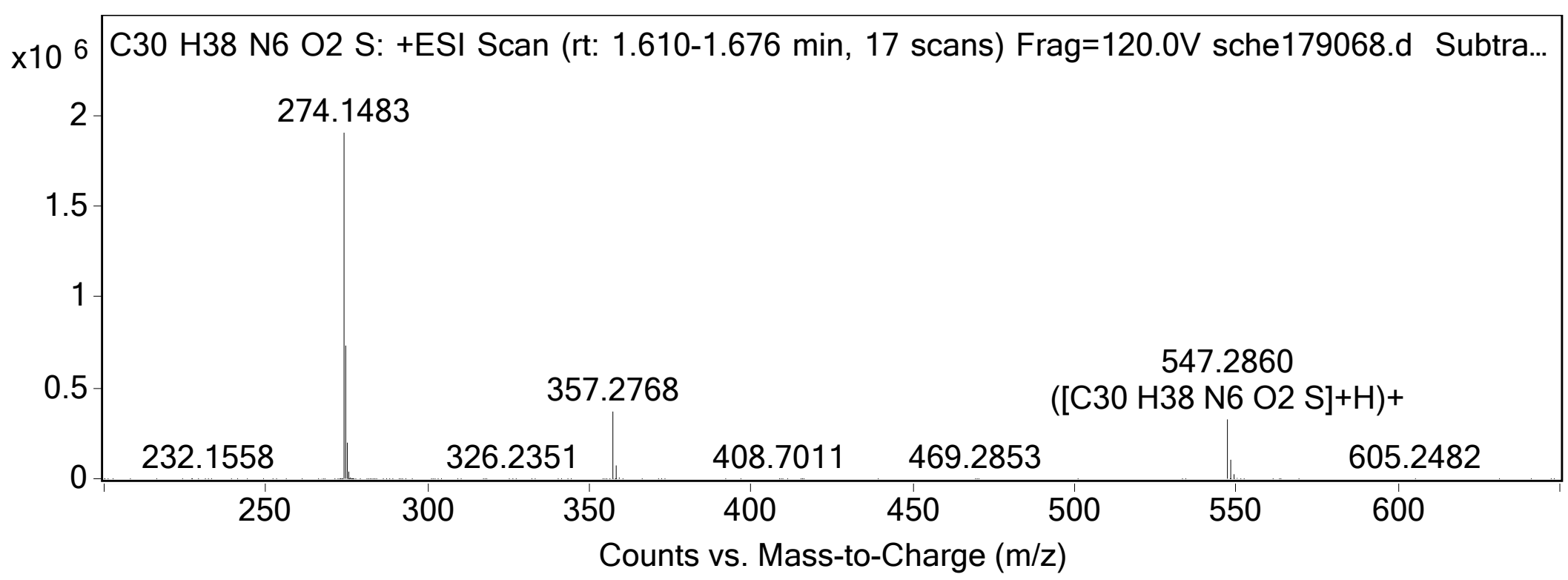


HRMS spectrum of compound **27**

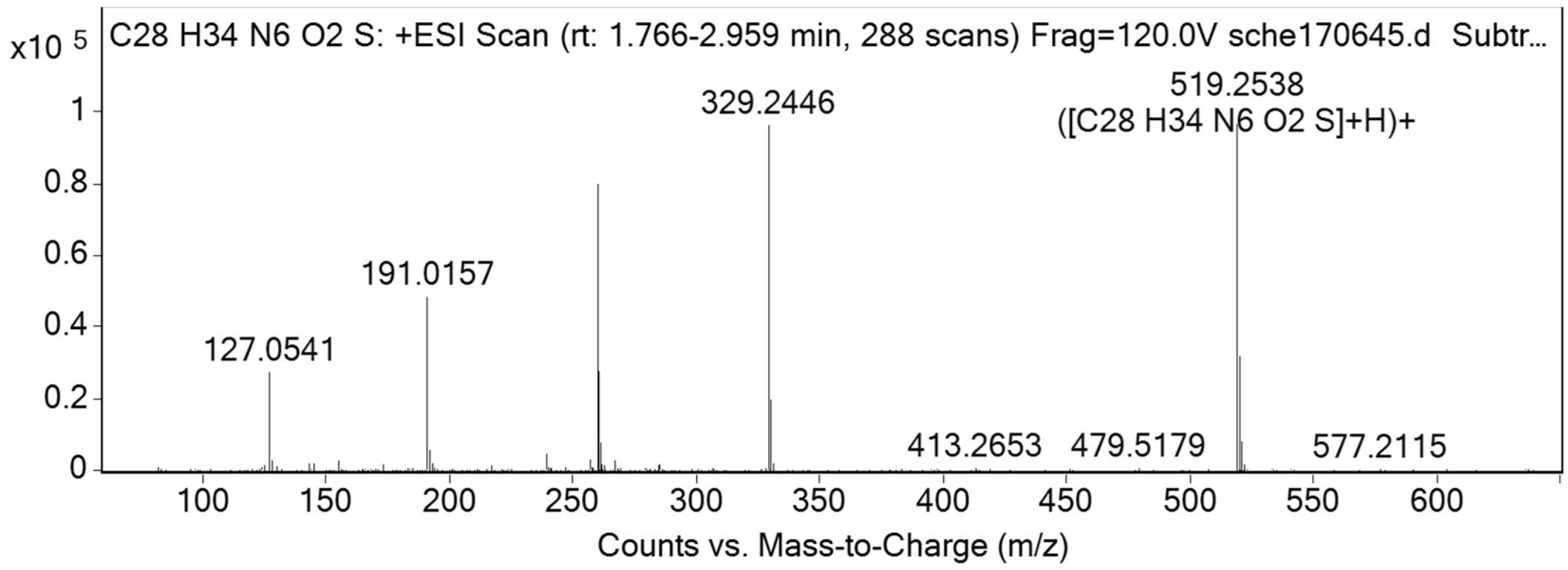


HRMS spectrum of compound **28**

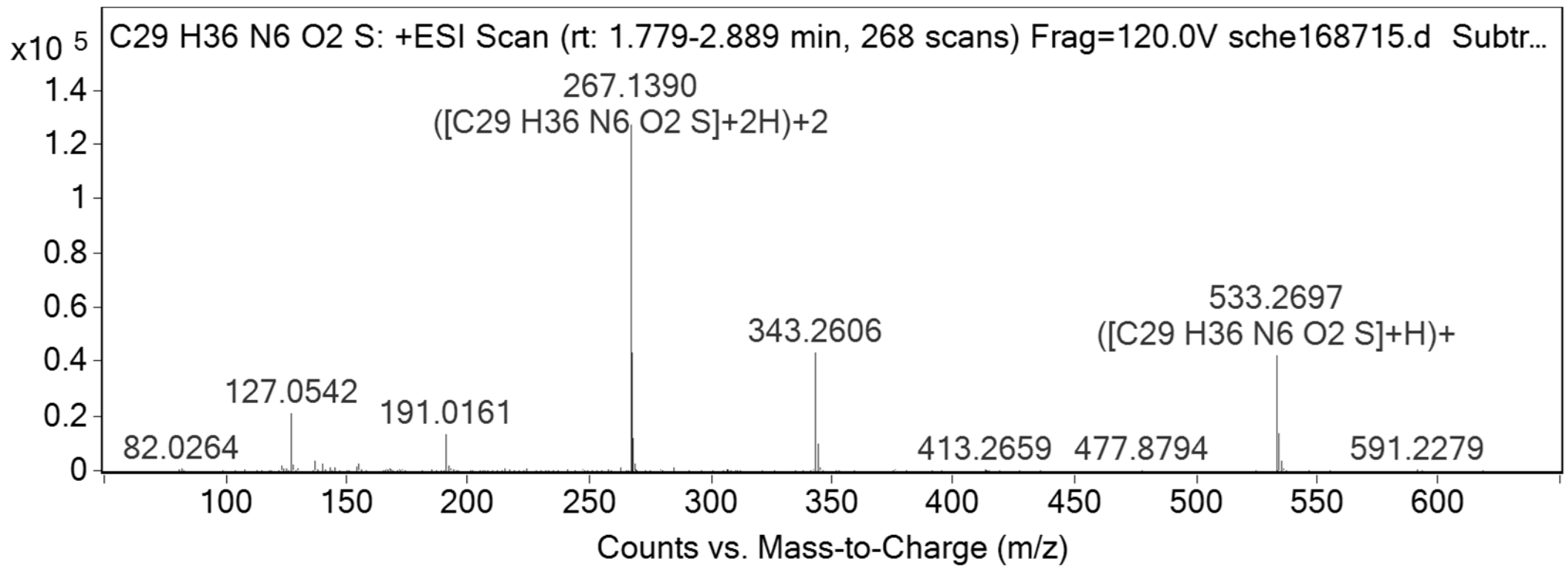


HRMS spectrum of compound **29**

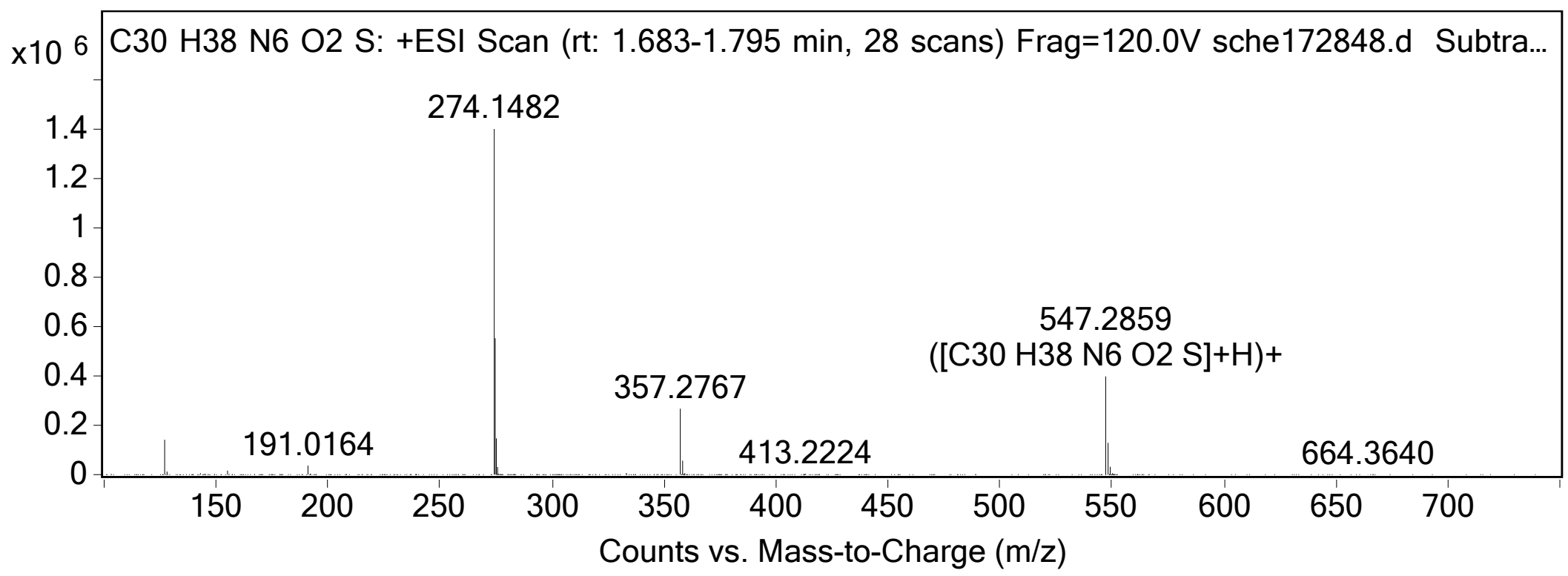


HRMS spectrum of compound **30**

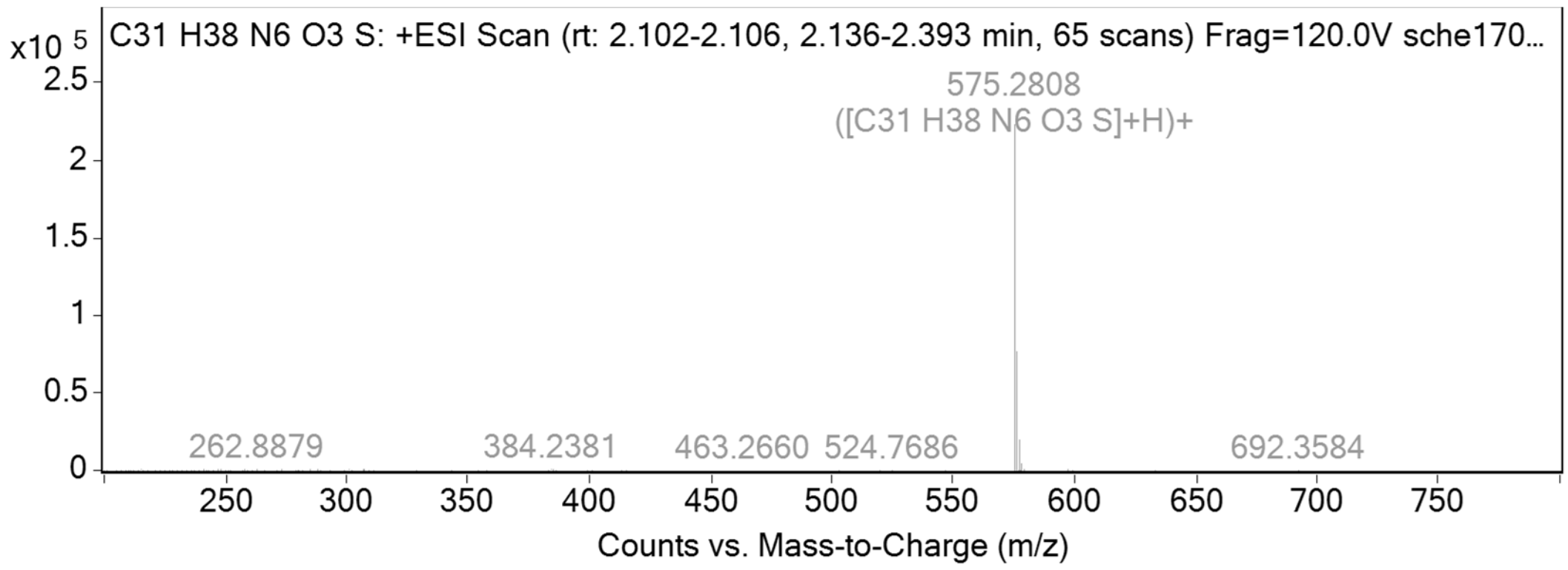


HRMS spectrum of compound **32**

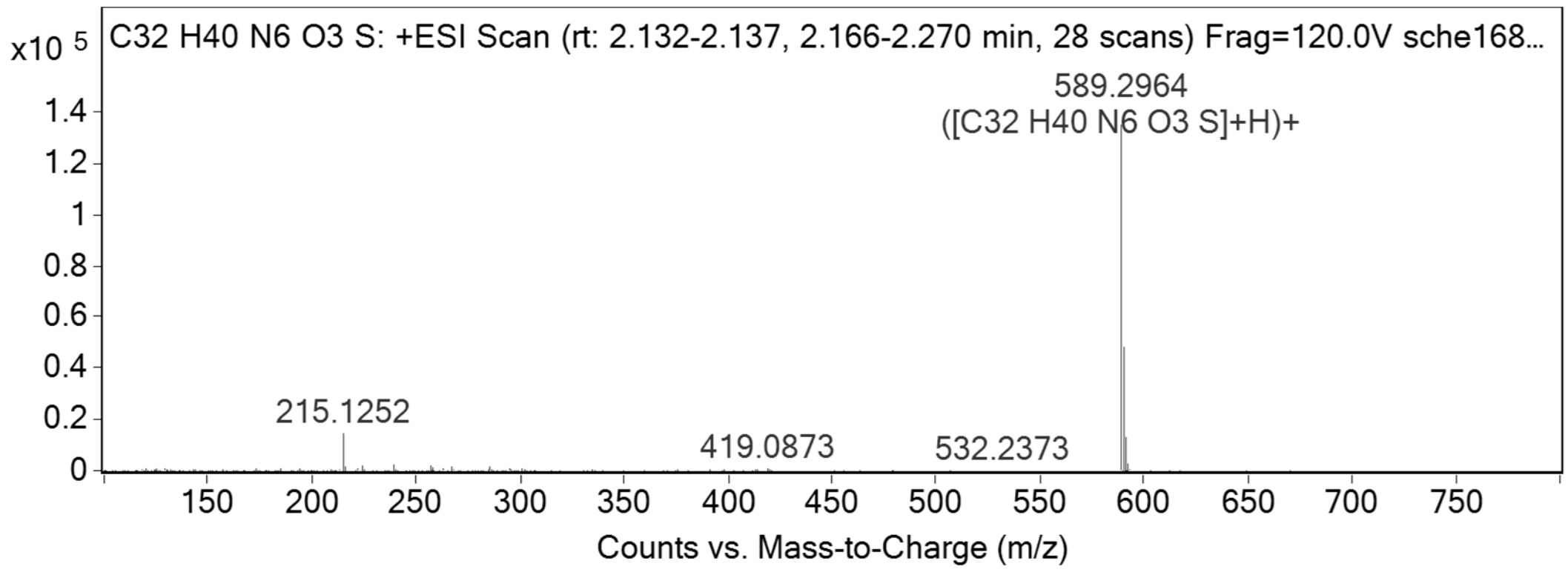


HRMS spectrum of compound **33**

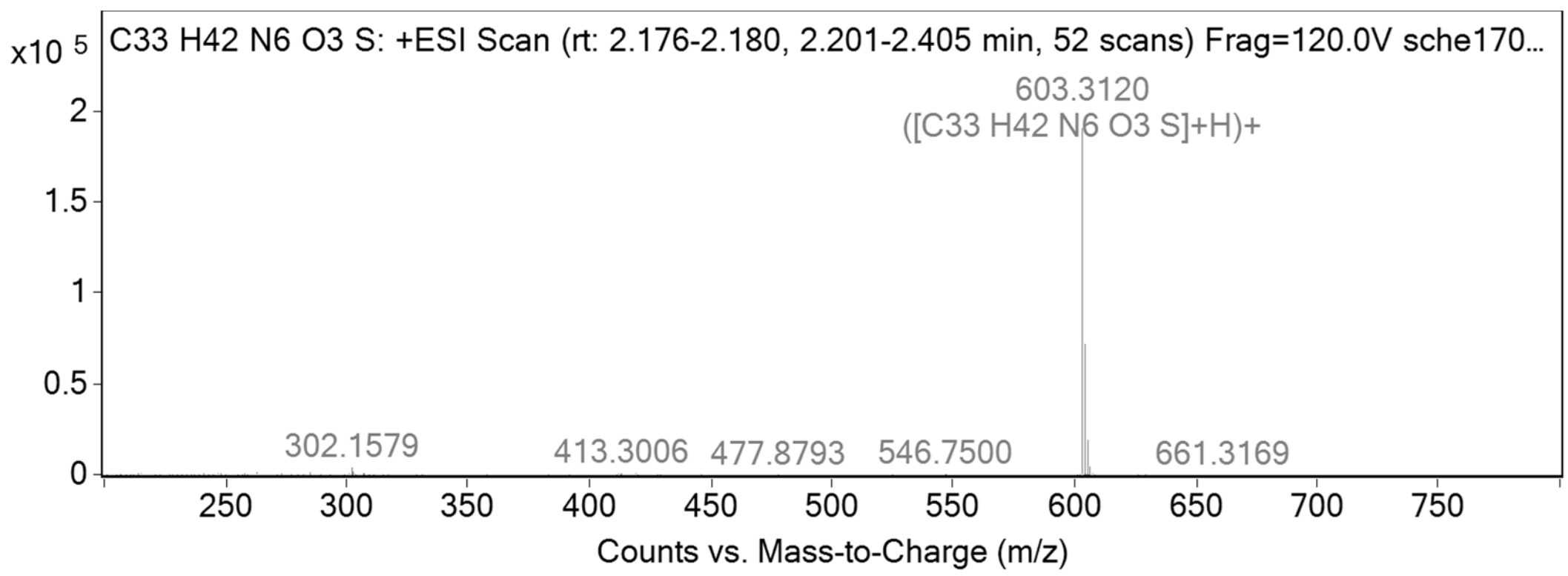


HRMS spectrum of compound **34**

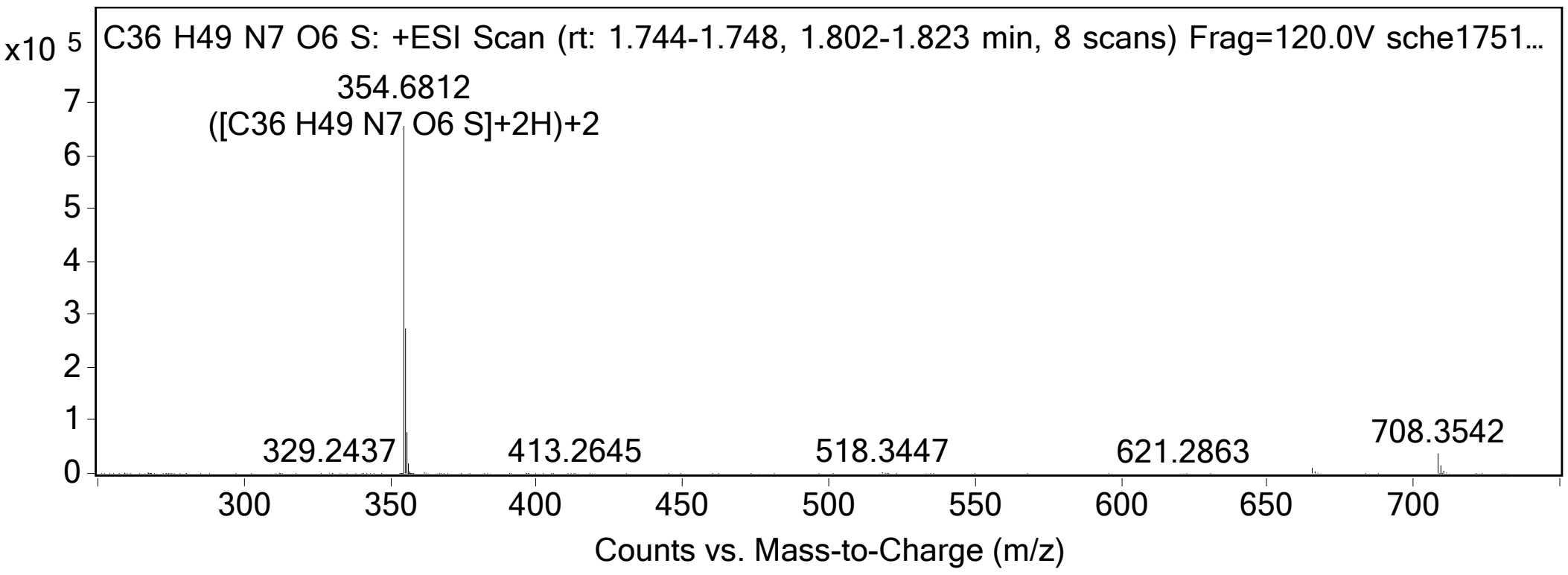


HRMS spectrum of compound **37**

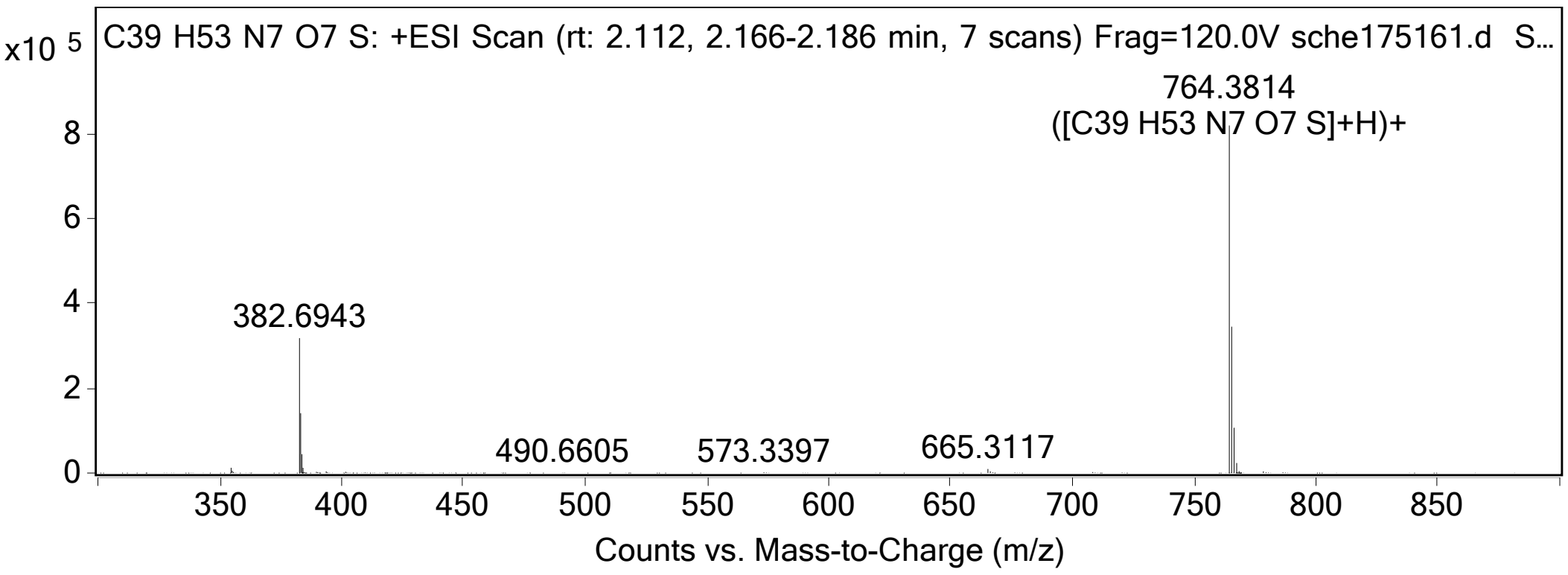


HRMS spectrum of compound **38**

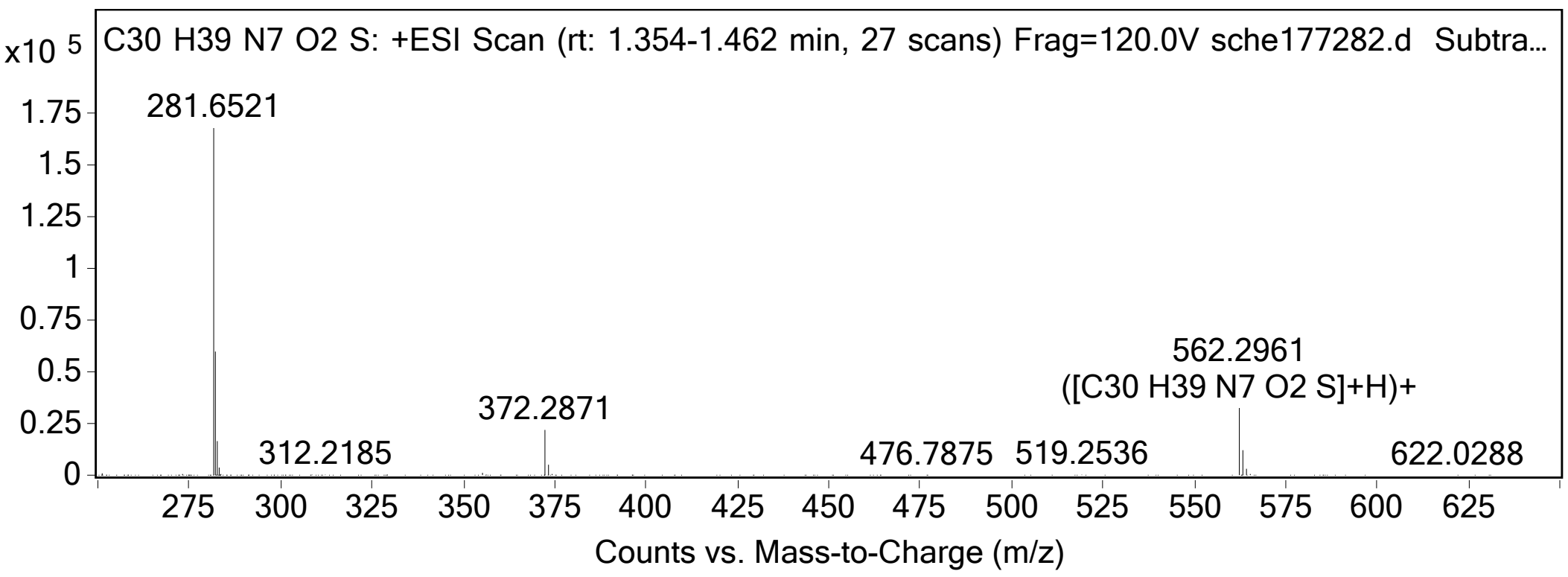


HRMS spectrum of compound **41**

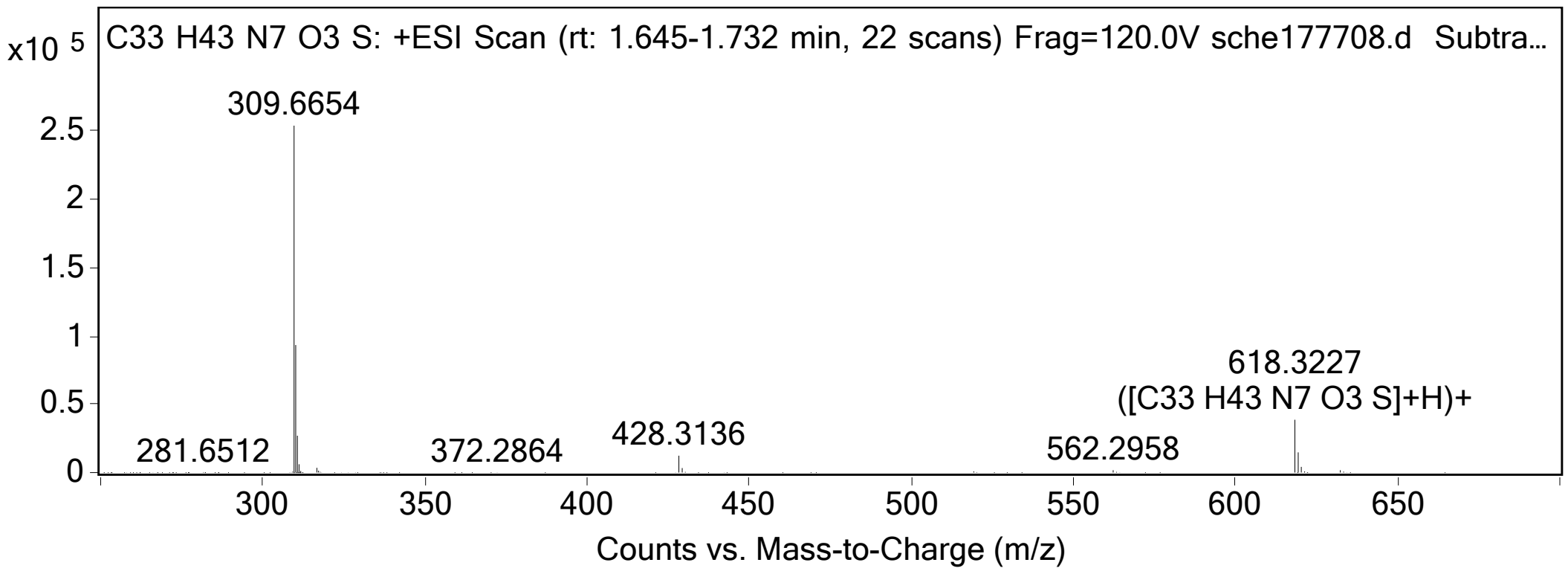


HRMS spectrum of compound **42**

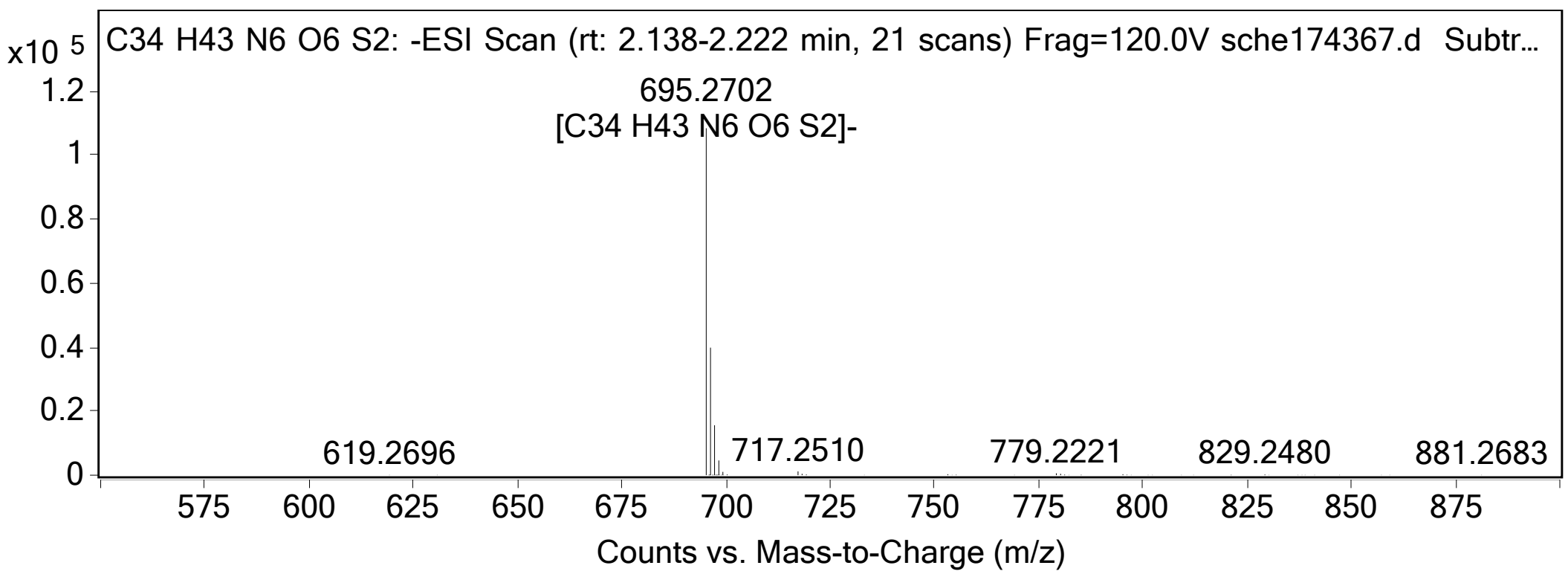


HRMS spectrum of compound **45**
