# Supplementary material for: Synthesis and Characterization of Tritium‐Labeled Neuropeptide Y Y5 Receptor Ligands Derived From CGP71683A
Source: Arch Pharm (Weinheim). 2026 Aug 17;359(8):e70314. doi: 10.1002/ardp.70314 (PMC13479786; doi:10.1002/ardp.70314)
Supplement: Supplementary file 2 [file ARDP-359-e70314-s001.doc]

**Supplemental Material: Novel Compounds and Biological Screening Results**

Tritiated Radioligands Derived from the Neuropeptide Y Y_5_ Receptor Antagonist CGP71683A

Franziska Schettler^1^, Pierre Koch^1^ and Max Keller^1^

^1^ Institute of Pharmacy, Faculty of Chemistry and Pharmacy, University of Regensburg, Regensburg, Germany

Correspondence:

Max Keller, Institute of Pharmacy, Faculty of Chemistry and Pharmacy, University of Regensburg, Universitätsstraße 31, D-93053 Regensburg, Germany

| **Compd. No.** | **InChI** | **Biological Activity** | | | | | |
| --- | --- | --- | --- | --- | --- | --- | --- |
|  |  | ***K*_i_, hY_1_R^a^** | ***K*_i_, hY_2_R^a^** | ***K*_i_, hY_4_R^a^** | ***K*_i_, hY_5_R^a^** | ***K*_d_, hY_5_R^b^** | ***K*_d_, hY_5_R^c^** |
| **26** | InChI=1S/C29H36N6O2S/c1-30-17-18-31-28-25-10-4-5-11-26(25)34-29(35-28)32-19-21-13-15-22(16-14-21)20-33-38(36,37)27-12-6-8-23-7-2-3-9-24(23)27/h2-12,21-22,30,33H,13-20H2,1H3,(H2,31,32,34,35)/t21-,22- | > 10 µM | > 1 µM | > 10 µM | 1.3 nM | - | - |
| **27** | InChI=1S/C30H38N6O2S/c1-31-18-7-19-32-29-26-11-4-5-12-27(26)35-30(36-29)33-20-22-14-16-23(17-15-22)21-34-39(37,38)28-13-6-9-24-8-2-3-10-25(24)28/h2-6,8-13,22-23,31,34H,7,14-21H2,1H3,(H2,32,33,35,36)/t22-,23- | - | - | - | 4.0 nM | - | - |
| **28** | InChI=1S/C28H34N6O2S/c29-16-17-30-27-24-9-3-4-10-25(24)33-28(34-27)31-18-20-12-14-21(15-13-20)19-32-37(35,36)26-11-5-7-22-6-1-2-8-23(22)26/h1-11,20-21,32H,12-19,29H2,(H2,30,31,33,34)/t20-,21- | > 10 µM | > 10 µM | > 10 µM | 2.5 nM | - | - |
| **29** | InChI=1S/C29H36N6O2S/c30-17-6-18-31-28-25-10-3-4-11-26(25)34-29(35-28)32-19-21-13-15-22(16-14-21)20-33-38(36,37)27-12-5-8-23-7-1-2-9-24(23)27/h1-5,7-12,21-22,33H,6,13-20,30H2,(H2,31,32,34,35)/t21-,22- | - | - | - | 2.6 nM | - | - |
| **30** | InChI=1S/C30H38N6O2S/c31-18-5-6-19-32-29-26-11-3-4-12-27(26)35-30(36-29)33-20-22-14-16-23(17-15-22)21-34-39(37,38)28-13-7-9-24-8-1-2-10-25(24)28/h1-4,7-13,22-23,34H,5-6,14-21,31H2,(H2,32,33,35,36)/t22-,23- | - | - | - | 23 nM | - | - |
| **32** | InChI=1S/C31H38N6O3S/c1-2-29(38)32-18-19-33-30-26-11-5-6-12-27(26)36-31(37-30)34-20-22-14-16-23(17-15-22)21-35-41(39,40)28-13-7-9-24-8-3-4-10-25(24)28/h3-13,22-23,35H,2,14-21H2,1H3,(H,32,38)(H2,33,34,36,37)/t22-,23- | > 10 µM | > 10 µM | > 10 µM | 1.9 nM | - | - |
| **33** | InChI=1S/C32H40N6O3S/c1-2-30(39)33-19-8-20-34-31-27-12-5-6-13-28(27)37-32(38-31)35-21-23-15-17-24(18-16-23)22-36-42(40,41)29-14-7-10-25-9-3-4-11-26(25)29/h3-7,9-14,23-24,36H,2,8,15-22H2,1H3,(H,33,39)(H2,34,35,37,38)/t23-,24- | - | - | - | 2.3 nM | - | - |
| **34** | InChI=1S/C33H42N6O3S/c1-2-31(40)34-20-7-8-21-35-32-28-13-5-6-14-29(28)38-33(39-32)36-22-24-16-18-25(19-17-24)23-37-43(41,42)30-15-9-11-26-10-3-4-12-27(26)30/h3-6,9-15,24-25,37H,2,7-8,16-23H2,1H3,(H,34,40)(H2,35,36,38,39)/t24-,25- | - | - | - | 2.2 nM | - | - |
| **37** | InChI=1S/C36H49N7O6S/c37-16-19-47-20-21-48-22-23-49-26-34(44)38-17-18-39-35-31-9-3-4-10-32(31)42-36(43-35)40-24-27-12-14-28(15-13-27)25-41-50(45,46)33-11-5-7-29-6-1-2-8-30(29)33/h1-11,27-28,41H,12-26,37H2,(H,38,44)(H2,39,40,42,43)/t27-,28- | > 10 µM | > 10 µM | > 10 µM | 7.8 nM | - | - |
| **38** | InChI=1S/C39H53N7O7S/c1-2-36(47)41-20-21-51-22-23-52-24-25-53-28-37(48)40-18-19-42-38-33-11-5-6-12-34(33)45-39(46-38)43-26-29-14-16-30(17-15-29)27-44-54(49,50)35-13-7-9-31-8-3-4-10-32(31)35/h3-13,29-30,44H,2,14-28H2,1H3,(H,40,48)(H,41,47)(H2,42,43,45,46)/t29-,30- | - | - | - | 117 nM | - | - |
| **41** | InChI=1S/C30H39N7O2S/c31-16-17-32-18-19-33-29-26-9-3-4-10-27(26)36-30(37-29)34-20-22-12-14-23(15-13-22)21-35-40(38,39)28-11-5-7-24-6-1-2-8-25(24)28/h1-11,22-23,32,35H,12-21,31H2,(H2,33,34,36,37)/t22-,23- | > 10 µM | > 10 µM | > 10 µM | 28.5 nM | - | - |
| **42** | InChI=1S/C33H43N7O3S/c1-2-31(41)35-20-18-34-19-21-36-32-28-11-5-6-12-29(28)39-33(40-32)37-22-24-14-16-25(17-15-24)23-38-44(42,43)30-13-7-9-26-8-3-4-10-27(26)30/h3-13,24-25,34,38H,2,14-23H2,1H3,(H,35,41)(H2,36,37,39,40)/t24-,25- | > 10 µM | > 10 µM | > 10 µM | 10.6 nM | - | - |
| **45** | InChI=1S/C34H44N6O6S2/c1-2-32(41)40(20-8-22-47(42,43)44)21-19-35-33-29-12-5-6-13-30(29)38-34(39-33)36-23-25-15-17-26(18-16-25)24-37-48(45,46)31-14-7-10-27-9-3-4-11-28(27)31/h3-7,9-14,25-26,37H,2,8,15-24H2,1H3,(H,42,43,44)(H2,35,36,38,39)/t25-,26- | > 10 µM | > 10 µM | > 10 µM | > 1 µM | - | - |
| [^3^H]**26** | InChI=1S/C29H36N6O2S/c1-30-17-18-31-28-25-10-4-5-11-26(25)34-29(35-28)32-19-21-13-15-22(16-14-21)20-33-38(36,37)27-12-6-8-23-7-2-3-9-24(23)27/h2-12,21-22,30,33H,13-20H2,1H3,(H2,31,32,34,35)/t21-,22-/i1T3 | - | - | - | - | 6.4 nM | 1.5 nM |
| [^3^H]**32** | InChI=1S/C31H38N6O3S/c1-2-29(38)32-18-19-33-30-26-11-5-6-12-27(26)36-31(37-30)34-20-22-14-16-23(17-15-22)21-35-41(39,40)28-13-7-9-24-8-3-4-10-25(24)28/h3-13,22-23,35H,2,14-21H2,1H3,(H,32,38)(H2,33,34,36,37)/t22-,23-/i1T3,2T2 | - | - | - | - | 4.5 nM | 1.6 nM |
| [^3^H]**42** | InChI=1S/C33H43N7O3S/c1-2-31(41)35-20-18-34-19-21-36-32-28-11-5-6-12-29(28)39-33(40-32)37-22-24-14-16-25(17-15-24)23-38-44(42,43)30-13-7-9-26-8-3-4-10-27(26)30/h3-13,24-25,34,38H,2,14-23H2,1H3,(H,35,41)(H2,36,37,39,40)/t24-,25-/i1T3,2T2 | - | - | - | - | 7.4 nM | 13 nM |

^a^ *K*_i_ value determined in a radioligand competition assay using intact cells expressing the neuropeptide Y receptor of interest.

^b^ *K*_d_ value determined in a radiochemical saturation binding assay at intact adherent HEC-1B-hY_5_R cells.

^c^ *K*_d_ value determined in a radiochemical saturation binding assay at intact suspended HEC-1B-hY_5_R cells.
